# Gut Virome of the World’s Highest-Elevation Lizard Species (*Phrynocephalus erythrurus* and *Phrynocephalus theobaldi*) Reveals Versatile Commensal Viruses

**DOI:** 10.1128/spectrum.01872-21

**Published:** 2022-02-23

**Authors:** Juan Lu, Shixing Yang, Chunmei Wang, Hao Wang, Ga Gong, Yuan Xi, Jiamin Pan, Xiaochun Wang, Jian Zeng, Ju Zhang, Peng Li, Quan Shen, Tongling Shan, Wen Zhang

**Affiliations:** a Department of Microbiology, School of Medicine, Jiangsu University, Zhenjiang, Jiangsu, China; b Shanghai Veterinary Research Institute, Chinese Academy of Agricultural Sciences, Shanghai, China; c The Affiliated Huai’an Hospital, Xuzhou Medical University, Huai’an, Jiangsu, China; d Animal Science College, Tibet Agriculture and Animal Husbandry University, Nyingchi, Tibet, China; e Jiangsu Key Laboratory for Biodiversity and Biotechnology, College of Life Sciences, Nanjing Normal University, Nanjing, Jiangsu, China; University of Prince Edward Island

**Keywords:** viral metagenomics, bacteriophages, gut virome, lizards, Qinghai-Tibet Plateau

## Abstract

The gut virome is a reservoir of diverse symbiotic and pathogenic viruses coevolving with their hosts, and yet limited research has explored the gut viromes of highland-dwelling rare species. Using viral metagenomic analysis, the viral communities of the Phrynocephalus lizards living in the Qinghai-Tibet Plateau were investigated. Phage-encoded functional genes and antibiotic resistance genes (ARGs) were analyzed. The viral communities of different lizard species were all predominated by bacteriophages, especially the *Caudovirales* order. The virome of Phrynocephalus erythrurus living around the Namtso Lake possessed a unique structure, with the greatest abundance of the *Parvoviridae* family and the highest number of exclusive viral species. Several vertebrate-infecting viruses were discovered, including caliciviruses, astroviruses, and parvoviruses. Phylogenetic analyses demonstrated that the virus hallmark genes of bacteriophages possessed high genetic diversity. After functional annotation, the majority of phage-associated functional genes were classified in the energy metabolism category. In addition, plenty of ARGs belonging to the multidrug category were discovered, and five ARGs were exclusive to the virome from Phrynocephalus theobaldi. This study provided the first insight into the structure and function of the virome in highland lizards, contributing to the protection of threatened lizard species. Also, our research is of exemplary significance for the gut virome research of lizard species and other cold-blooded and highland-dwelling animals, prompting a better understanding of the interspecific differences and transmission of commensal viruses.

**IMPORTANCE** The *Phrynocephalus* lizards inhabiting the Qinghai-Tibet Plateau (QTP) are considered to be the highest-altitude lizard species in the world, and they have been added to the IUCN list of threatened species. Living in the QTP with hypoxic, arid, natural conditions, the lizards presented a unique pattern of gut virome, which could provide both positive and negative effects, such as the enrichment of functional genes and the dissemination of antibiotic resistance genes (ARGs). This work provides the foundation for further research on the gut virome in these endangered lizard species and other cold-blooded and highland-dwelling animals, contributing to the maintenance of ecological balance on the plateau.

## INTRODUCTION

The habitat elevation of Phrynocephalus lizards reaches the altitude of the Qinghai-Tibet Plateau (QTP), which is known as the “roof of the world” and has an average altitude of over 4,500 m (1). The Phrynocephalus genus contains over 40 species of toad-headed lizards, some of which are widely distributed in the QTP (2), including the species Phrynocephalus erythrurus and Phrynocephalus theobaldi, which are considered to be the highest-altitude lizard species in the world (3). Living restricted to the QTP with its harsh climate, these species need to have the adaptive ability to survive in the hypoxic, arid, natural conditions (4). As global warming continues, the QTP is being affected as a vulnerable ecological environment sensitive to natural and anthropogenic disturbances (5). Overwhelmed by the increasing environmental pressure, the two endangered lizard species have both been listed in the International Union for Conservation of Nature’s *IUCN Red List of Threatened Species* (6, 7). Conducting research on the endangered lizard species is of vital ecological significance in maintaining the ecological balance on the plateau.

Previous studies revealed the genetic mechanisms for native animals to adapt to the extremely inhospitable highland, and additional functional genes related to energy metabolism were identified from animals living in the QTP, such as yaks (Bos grunniens) (8) and antelope (Pantholops hodgsonii) (9). Microbial genomes, known as the “second genome,” also play a vital role in the adaptation of highland-dwelling animals, and research on yak rumen and pika gut microbiomes highlights the adaptive contributions of microbial communities (10, 11). The gut microbiome of western fence lizards (Sceloporus occidentalis) also presents adaptive changes against different temperatures (12). Nevertheless, despite its being an indispensable part of the microbiome, consisting of eukaryotic and prokaryotic viruses from lizards, gut bacteria, and their food that have both pathogenic and beneficial effects, the gut virome in lizards is largely unexplored (13). On one hand, wild reptiles, including snakes, crocodiles, and lizards, could be potential reservoirs of pathogenic viruses that pose health risks to human and nonhuman animals, leading to the spread of zoonotic disease (14, 15). On the other hand, the transkingdom interactions between bacteriophages and their bacterial hosts in the intestine could contribute to host fitness by altering gene expression or mediating gene transduction (16). Given the complicated functions of the virome, the necessity to explore the composition of viral communities in highland lizards’ guts in order to protect these endangered species is great.

The biological functions of bacteriophages could extend to the dissemination of antibiotic resistance genes (ARGs) through horizontal gene transfer (HGT), which puts an increasing strain on public health (17). The abuse of antibiotics may bring about antibiotic-resistant bacteria and thus interfere with the treatment and control of infectious diseases (18), and phage transduction may accelerate the progress (19). Phage-associated ARGs have been discovered from various sources that are closely related to human activities, such as animals, including livestock and poultry (20), and aquatic environments, including wastewater and freshwater (21, 22). However, the ARGs of animals living in the desolate and underpopulated plateau have not yet been explored. Hence, there might be a unique pattern of phage-encoded ARGs in the two lizard species in the study.

In the present study, we investigated the viral communities of the P. erythrurus and P. theobaldi lizards living in the QTP and analyzed the genetic diversity of the main virus groups based on virus hallmark genes through phylogenetic analyses. Then, we explored the composition of phage-related functional genes and ARGs in the highland lizards’ gut viromes. The findings in this study could reveal the composition and function of the gut viromes in lizards inhabiting the plateau, contributing to the knowledge of the gut viromes in these endangered lizard species and other cold-blooded and highland-dwelling animals.

## RESULTS

### Overview of sequencing outcomes.

**FIG 1:**
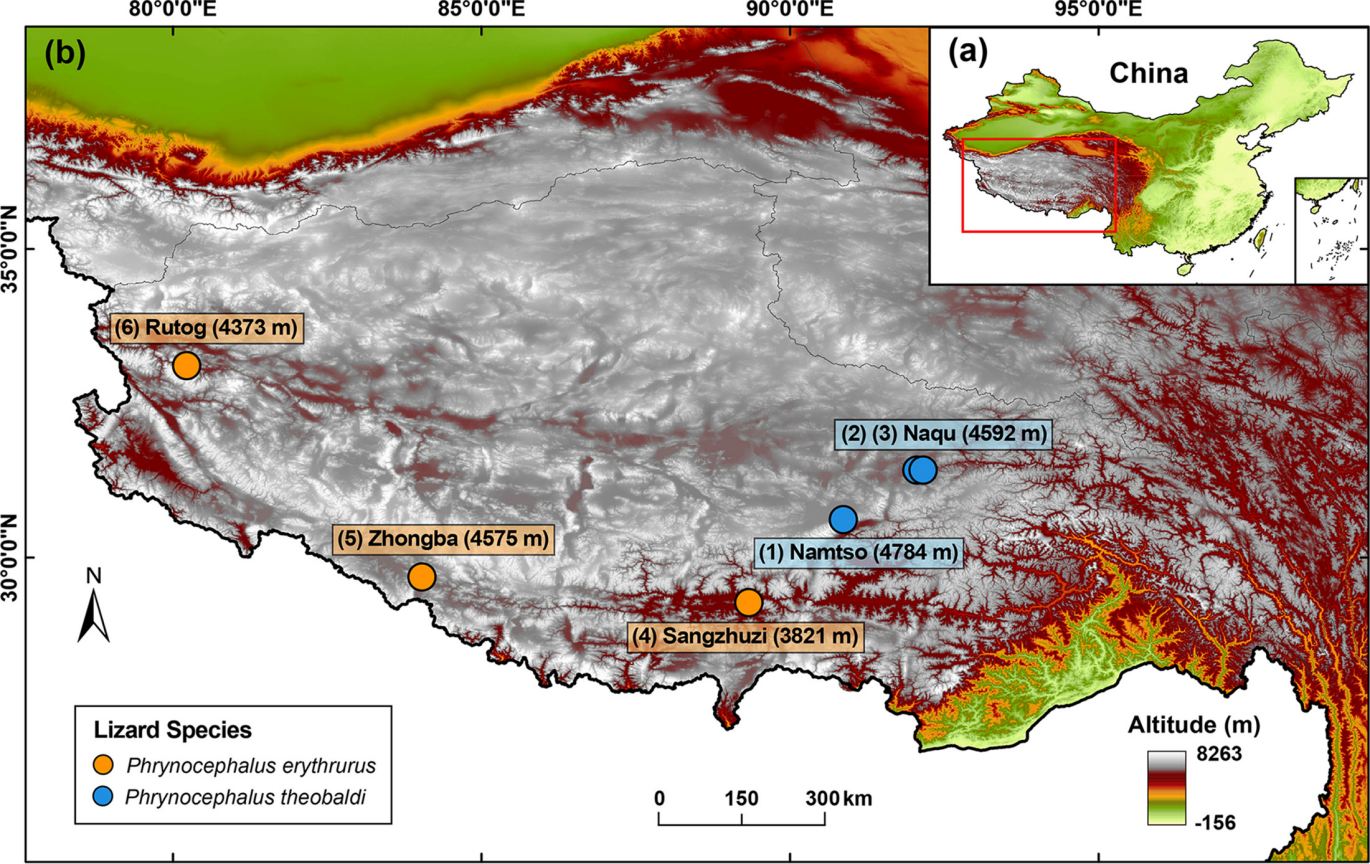
Map of the Qinghai-Tibet Plateau with sampling sites. (a) Location within China. (b) Magnified view of the plateau in the red box of panel a. The sampling sites are indicated by dots and labeled with city names and altitudes. Lizard species are indicated by the color coding shown in the key. The map was created by the authors.

To explore the gut virome of highland lizards, we carried out a complicated viral metagenomics investigation of both DNA and RNA viruses in 81 fecal samples from the lizards in the Qinghai-Tibetan Plateau (Fig. 1). These fecal samples were pooled into six sample pools according to the two lizard species (P. erythrurus and P. theobaldi) and five sampling sites (Namtso, Naqu, Sangzhuzi, Zhongba, and Rutog). After library construction and next-generation sequencing on the Illumina NovaSeq platform, the six libraries generated a total of 86,640,498 raw sequence reads with an average length of 247 bp and an average GC content of 45.3%. The sequence reads were binned according to barcode and assembled into larger contigs. In total, 322,225 viral contigs (average length of 554.3 bp) were obtained through *de novo* assembly within the six bins and alignment against the viral protein database using BLASTx. The percentage of raw reads mapped to the viral contigs in each library ranged from 39.26% to 59.81% (Table S1 in the supplemental material). As for sequences in the blank control, no detectable DNA existed in the control library during quality inspection. Equal volumes of the seven libraries were included in the run of Illumina NovaSeq 6000 platform sequencing, where the control library generated a small number of reads (*n* = 14,162). BLASTx searching based on the total reads in the control library revealed no viral sequences.

### Characterization of viral communities.

**FIG 2:**
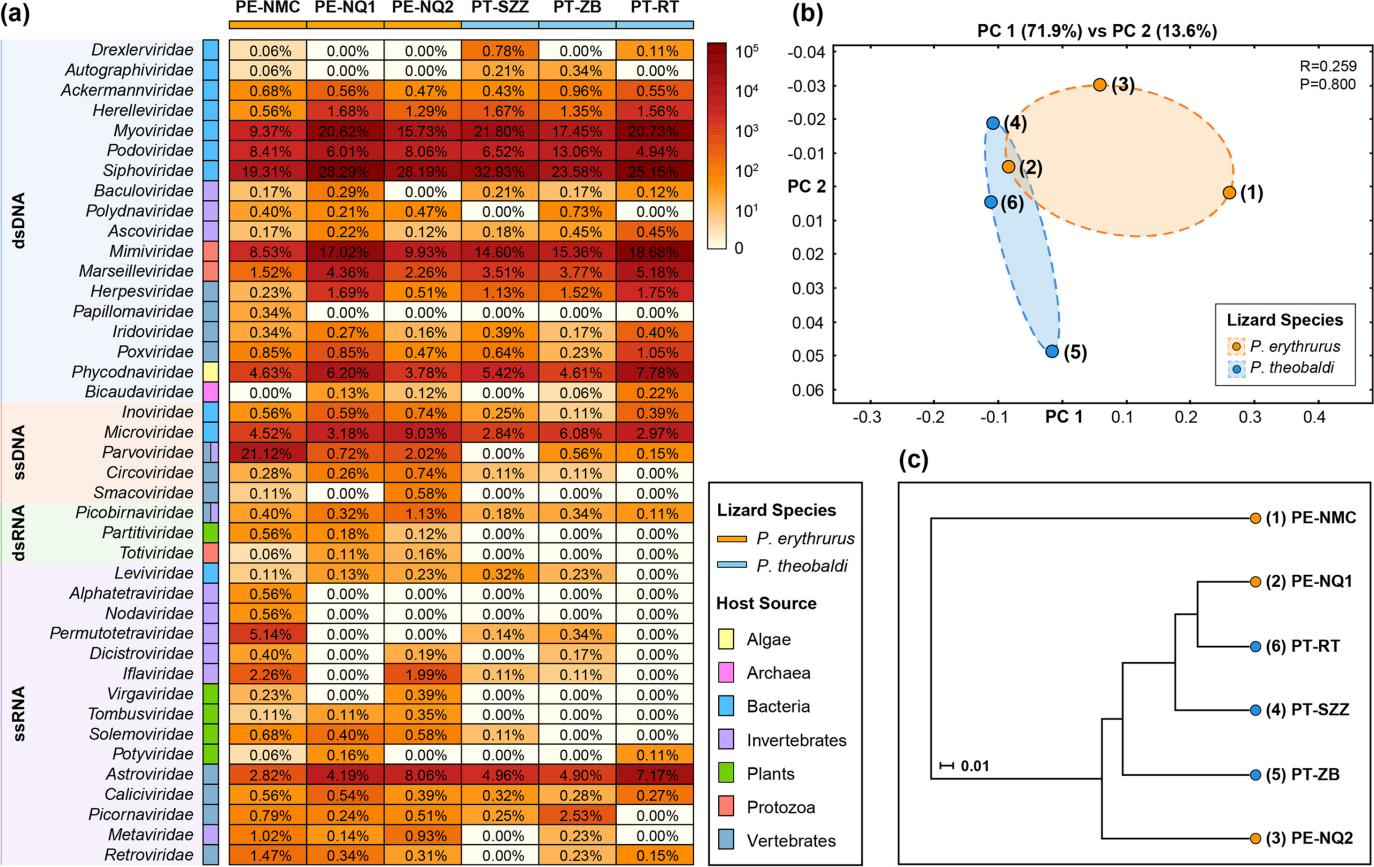
Viral taxonomy analyses at the family level. (a) Heatmap representing the read number of each viral family in exponential form. Host sources and lizard species are indicated with the color coding shown in the key. Viral genome composition is represented by different-colored rectangles, with taxon names indicated on the left. The percentages of each viral family in the six viromes are shown. The sample names are defined in the form of lizard species-sampling site. PE, Phrynocephalus erythrurus; PT, Phrynocephalus theobaldi; NMC, Namtso, NQ, Naqu; SZZ, Sangzhuzi; ZB, Zhongba; RT, Rutog. (b, c) PCoA plot (b) and UPGMA taxonomic tree (c) showing the similarity of viral community structures based on the Bray-Curtis ecological distance matrix. The *P* value was calculated using ANOSIM.

At the family level, viral contigs and singlet reads were classified into 41 viral families, including 18 double-stranded DNA (dsDNA) viral families, five single-stranded DNA (ssDNA) viral families, three double-stranded RNA (dsRNA) viral families, and 15 single-stranded RNA (ssRNA) viral families (Fig. 2a). The order *Caudovirales* accounted for the largest percentage of viral reads in the six libraries, mainly dominated by *Siphoviridae* (26.24% ± 4.26% [mean ± standard deviation]), *Myoviridae* (17.62% ± 4.24%), *Podoviridae* (7.83% ± 2.62%), and other bacteriophage families. Fewer reads were assigned to other dsDNA viral families, such as *Mimiviridae* (14.02% ± 3.64%) and *Marseilleviridae* (3.43% ± 1.237%), which infect amoebas, and *Phycodnaviridae* (5.40% ± 1.30%), which infect algae. The most abundant ssDNA viral family was *Microviridae* (4.77% ± 2.21%), whose members are bacteriophages, except in the virome from Namtso, which was predominated by *Parvoviridae* (21.12%). Relatively small numbers of reads were identified as being homologous to circular Rep-encoding single-stranded (CRESS) DNA viruses, including vertebrate-infecting *Circoviridae* (0.25% ± 0.24%) and *Smacoviridae* (0.12% ± 0.21%). The *Riboviria* realm was mainly dominated by the family *Astroviridae* (5.35% ± 1.77%), whose members infect vertebrates. Other RNA viral families were distributed sporadically in the six viromes, such as invertebrate-infecting *Permutotetraviridae* in the virome from Namtso (5.14%) and vertebrate-infecting *Picornaviridae* in the virome from Zhongba (2.53%). The comparison of viral communities between Phrynocephalus erythrurus and Phrynocephalus theobaldi through principal-coordinate analysis (PCoA) suggested that there was no statistically significant difference between the two lizard groups at the family level (*P* > 0.05) (Fig. 2b). Meanwhile, the unweighted pair group method with arithmetic mean (UPGMA) dendrogram presented a clear separation between the virome from Namtso and the other five viromes. The second and the sixth viromes from different lizard species were clustered together, and thus, they might share similar compositions and abundances (Fig. 2c).

**FIG 3:**
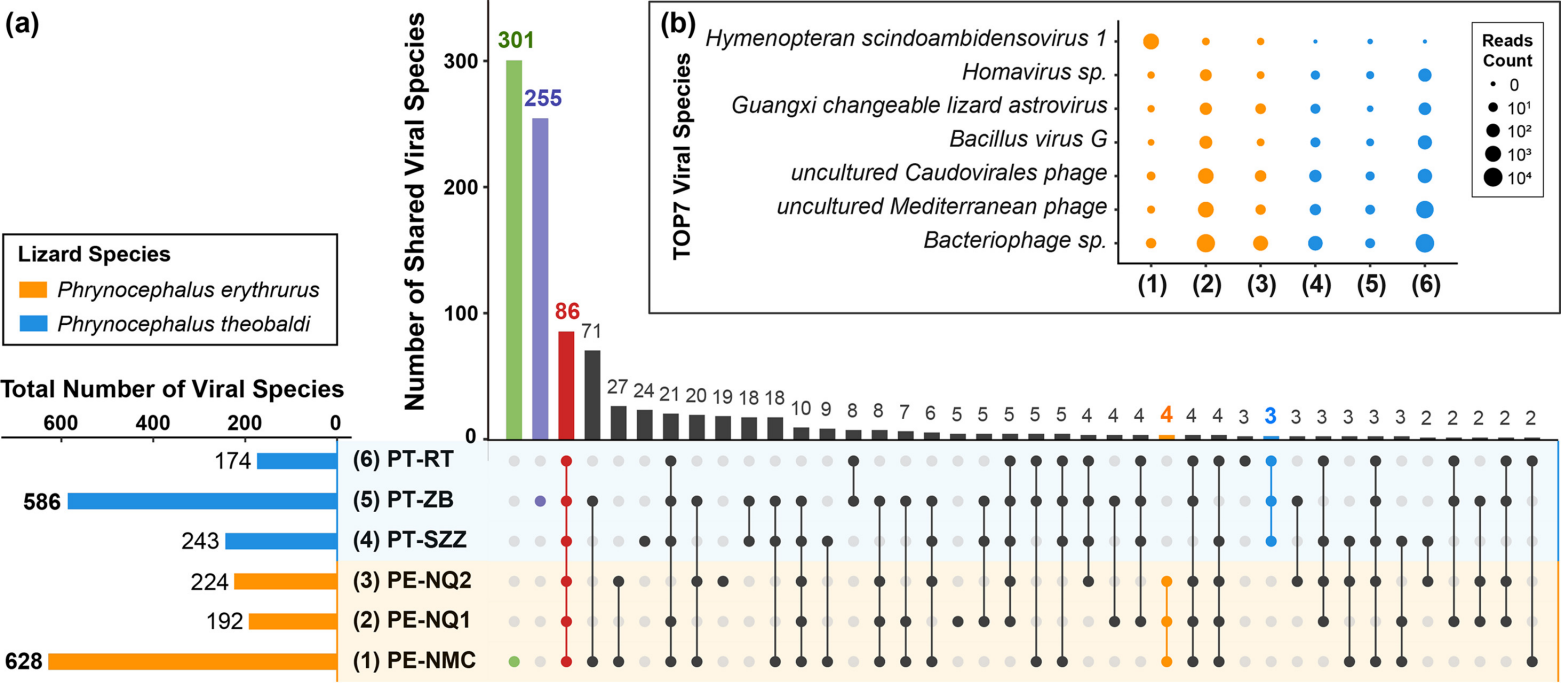
Viral taxonomy analyses at the species level. (a) UpSet plot depicting the numbers of shared viral species among the six viromes. Filled dots with interconnecting vertical lines represent the intersections, and unfilled light gray dots represent sets that do not belong to the intersections. The bars above represent the numbers of species within the intersections, and the bars to the left depict the total number of viral species in each virome set. (b) Bubble chart showing the top 7 most abundant viral species in the six viromes. Bubble size indicates the abundance of reads assigned to each species. In both panels, the lizard species are indicated with the color coding shown in the key in panel a.

At the species level, a total of 1,044 viral species were identified in the six viral communities. The virome from Namtso had the highest number of viral species, followed by the one from Zhongba. Meanwhile, the two viromes also contained considerable numbers of distinctive species, 301 and 255, respectively (Fig. 3a). The substantial numbers of exclusive viral species in the two viromes demonstrated their unique viral community signatures. However, a total of 86 viral species were shared in all viromes of the two lizard species, accounting for 13% to 49% of the total number of species identified in each of the six viromes. It is worth mentioning that not only did large proportions of viral species exist simultaneously in both lizard species, but also, few viral species were exclusive to either one of them, four and three species, respectively. These results revealed the slight influence of lizard species on viral community structure at the species level. For further analyses, the results for the top 7 most abundant viral species in the six viromes are depicted in Fig. 3b; these were dominated by viral species belonging to bacteriophages, such as uncultured *Caudovirales* phage and uncultured Mediterranean phage. In addition, species *Hymenopteran scindoambidensovirus 1* of the *Parvoviridae* family was particularly abundant in the virome from Namtso, indicating that the virome possibly possessed a unique viral community structure.

### Identification of virus hallmark gene sequences.

In this study, 465 viral sequences were generated after extension and annotation of contigs (Fig. S1; Table S2). These sequences belonged to the five abundant virus groups in the study and contained the virus hallmark genes (i.e., conserved domains) of each virus group (23, 24), including the genes encoding phage terminase large subunit (TerL) for *Caudovirales*, major capsid protein (MCP) for *Microviridae*, replication-associated protein (Rep) for CRESS DNA viruses, nonstructural protein 1 (NS1) for *Parvoviridae*, and RNA-dependent RNA polymerase (RdRp) domain for *Riboviria*. The BLASTx results showed that those sequences shared sequence identities with their best matches ranging from 23.04% to 100%, and their lengths ranged from 305 bp to 11,503 bp with an average of 2,294 bp. To phylogenetically analyze these viral sequences, 186 sequences among them with a complete coding sequence (CDS) of a virus hallmark gene were selected for further construction of phylogenetic trees.

### Phylogenetic analyses of the main virus groups.

**FIG 4:**
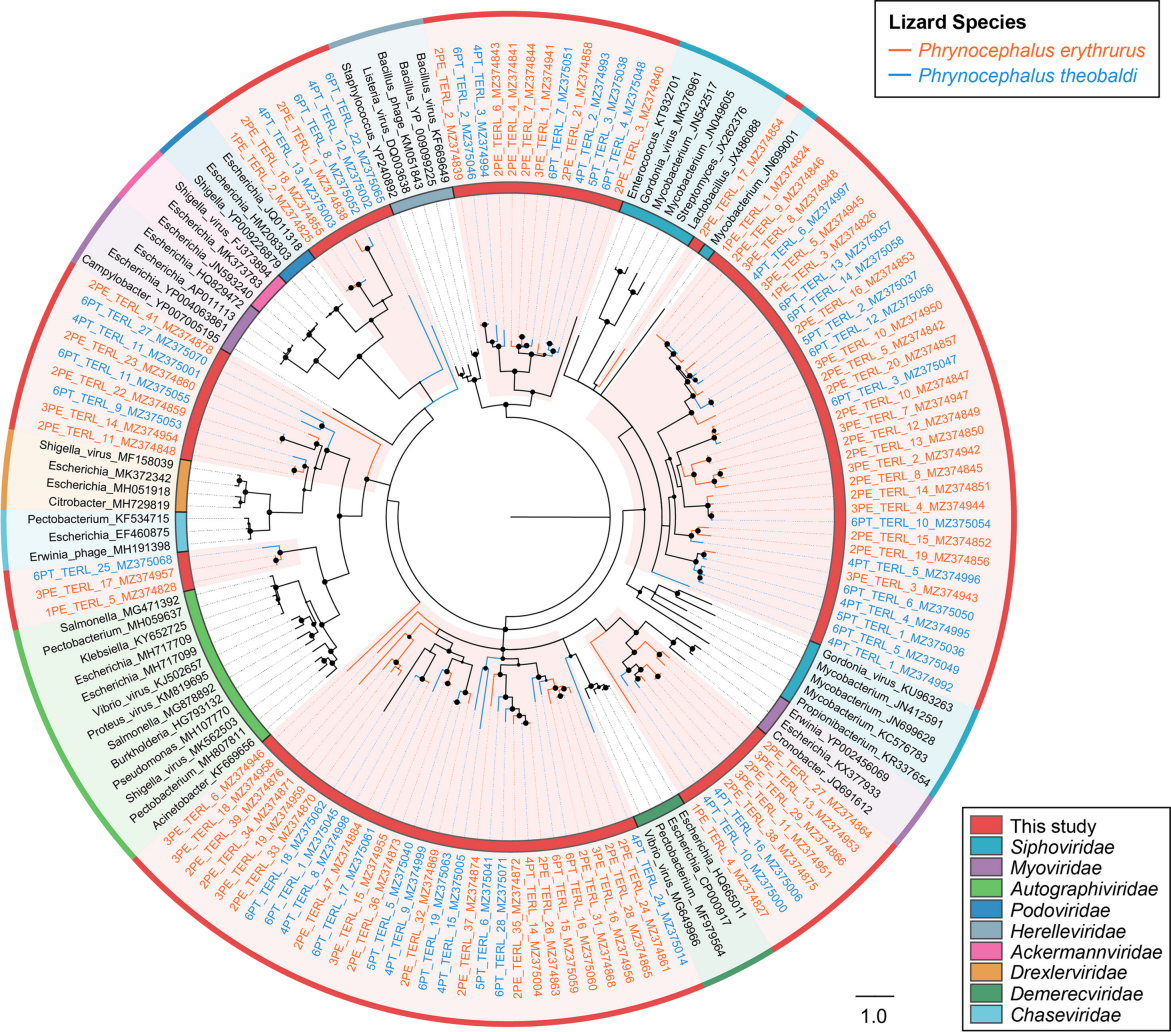
The phylogeny of *Caudovirales* identified in feces of lizards. Bayesian inference tree was established based on amino acid sequences of TerL of *Caudovirales*. Representative strains of all families in *Caudovirales* are included and marked with the color coding in the key on the bottom right. The viruses found in this study are indicated by red-filled sectors and orange or blue lines according to lizard species. The sizes of the black dots on the nodes are positively correlated with the corresponding bootstrap scores. The scale bar indicates the amino acid substitutions.

**FIG 5:**
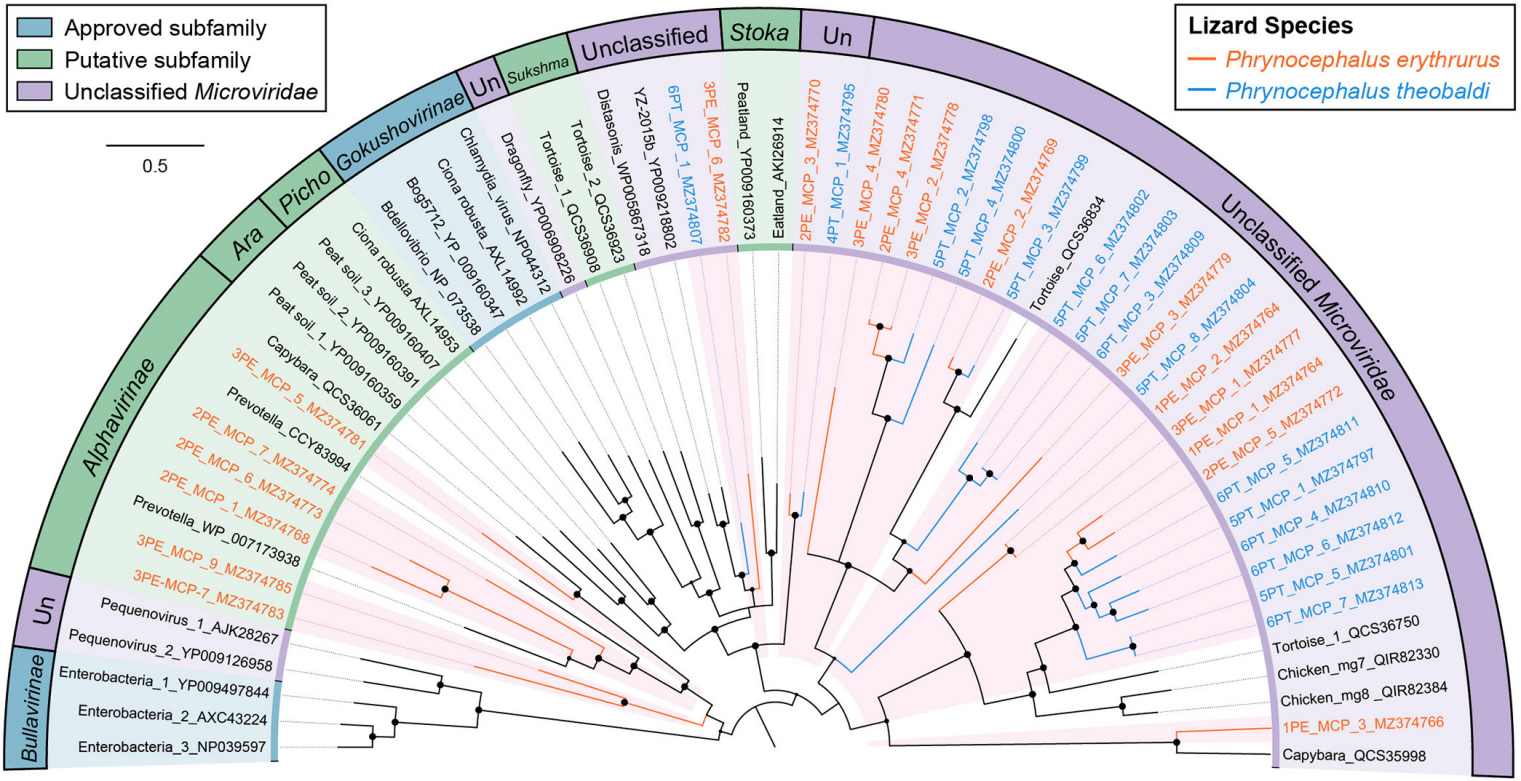
The phylogeny of *Microviridae* identified in feces of lizards. Bayesian inference tree established based on amino acid sequences of MCP of *Microviridae*. The viruses found in this study are marked with colored lines and red-filled sectors. Different viral groups are marked with color coding shown in the key on the upper left. The sizes of the black dots on the nodes are positively correlated with the corresponding bootstrap scores. The scale bar indicates the amino acid substitutions. *Ara*, *Aravirinae*; *Picho*, *Pichovirinae*; *Sukshma*, *Sukshmavirinae*; *Stoka*, *Stokavirinae*; Un, unclassified *Microviridae*.

The phylogenies of the main virus groups in the study were constructed based on the amino acid sequences encoded by complete virus hallmark genes and the corresponding reference protein sequences in the GenBank database. In total, 186 sequences with complete marker regions were selected for phylogenetic analyses. The order *Caudovirales* and the family *Microviridae* represent the largest categories of dsDNA and ssDNA bacteriophages, respectively. The phage large terminase subunit (TerL) region is highly conserved in its evolution in the *Caudovirales* order, and thus, the phylogenetic analysis of dsDNA phages was performed based on the 105 TerL protein sequences that were identified in this study (Fig. 4). The sequences from P. erythrurus distributed alternating with those from P. theobaldi. The tree structure revealed that the majority of phage TerL sequences were too divergent to be classified into known families in the *Caudovirales* order and were grouped into several distinct clusters among the representative clades. The divergence was supported by high bootstrap values, indicating the hidden diversity of tailed bacteriophages in the study. Meanwhile, the phylogenetic tree based on 33 MCPs of *Microviridae* also revealed the substantial genetic variety of ssDNA bacteriophages (Fig. 5). Specifically, none of them showed a close relationship to the approved subfamilies, including *Bullavirinae* and *Gokushovirinae*. Moreover, six of these sequences from P. erythrurus were phylogenetically grouped into the putative subfamily *Alphavirinae*, and the rest of them were classified into separate clades genetically distinct from those classified into subfamilies. These results revealed the undiscovered diversity of bacteriophages from lizards living in the plateau.

**FIG 6:**
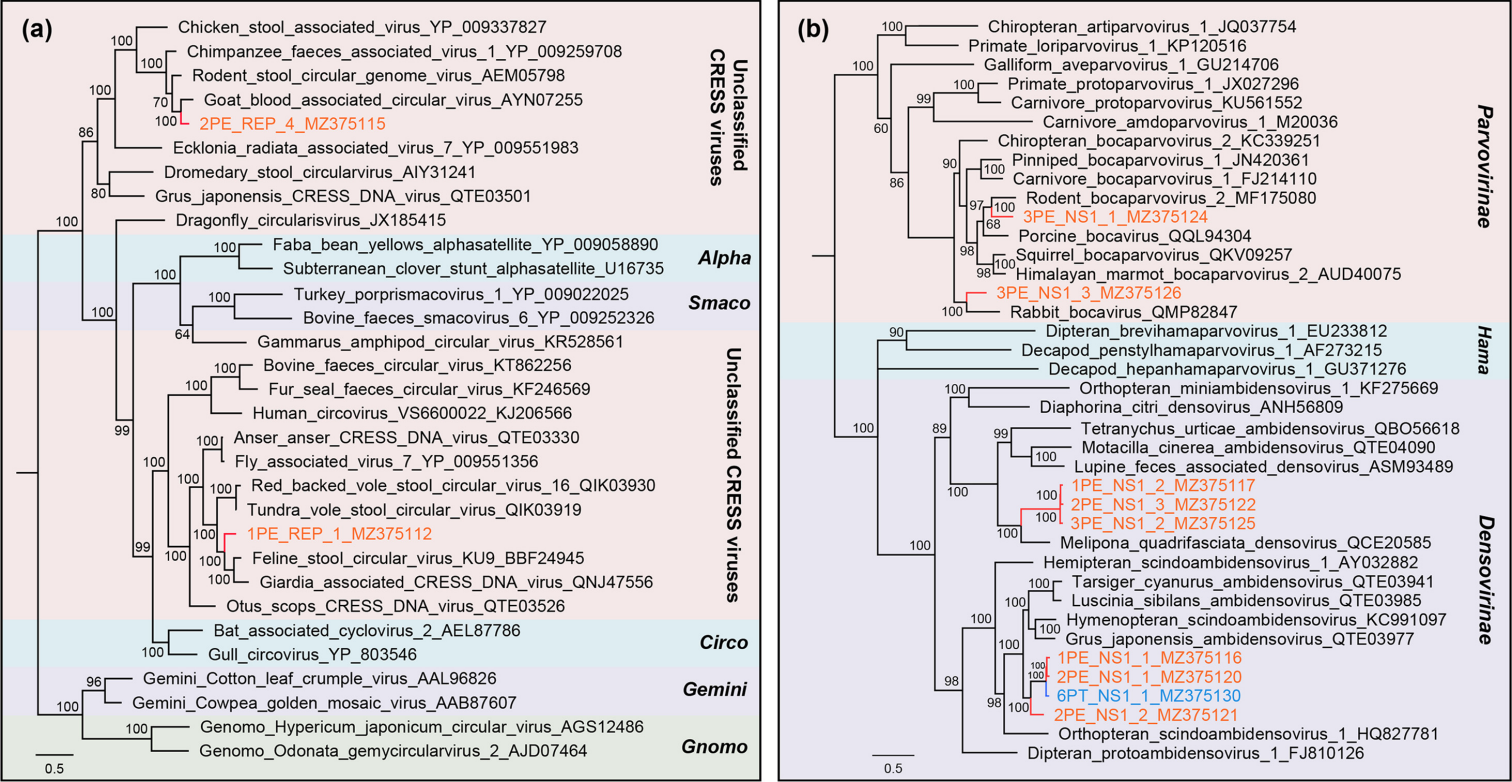
The phylogenies of CRESS DNA viruses and *Parvoviridae* identified in feces of lizards. (a) Bayesian inference tree established based on amino acid sequences of Rep protein of CRESS DNA viruses. (b) Bayesian inference tree established based on amino acid sequences of NS1 protein of *Parvoviridae*. The viruses found in this study are marked with colored lines and letters. Different taxonomic clusters were represented by rectangles filled with different colors, and taxon names are indicated on the right. Scale bars indicate the amino acid substitutions per site.

**FIG 7:**
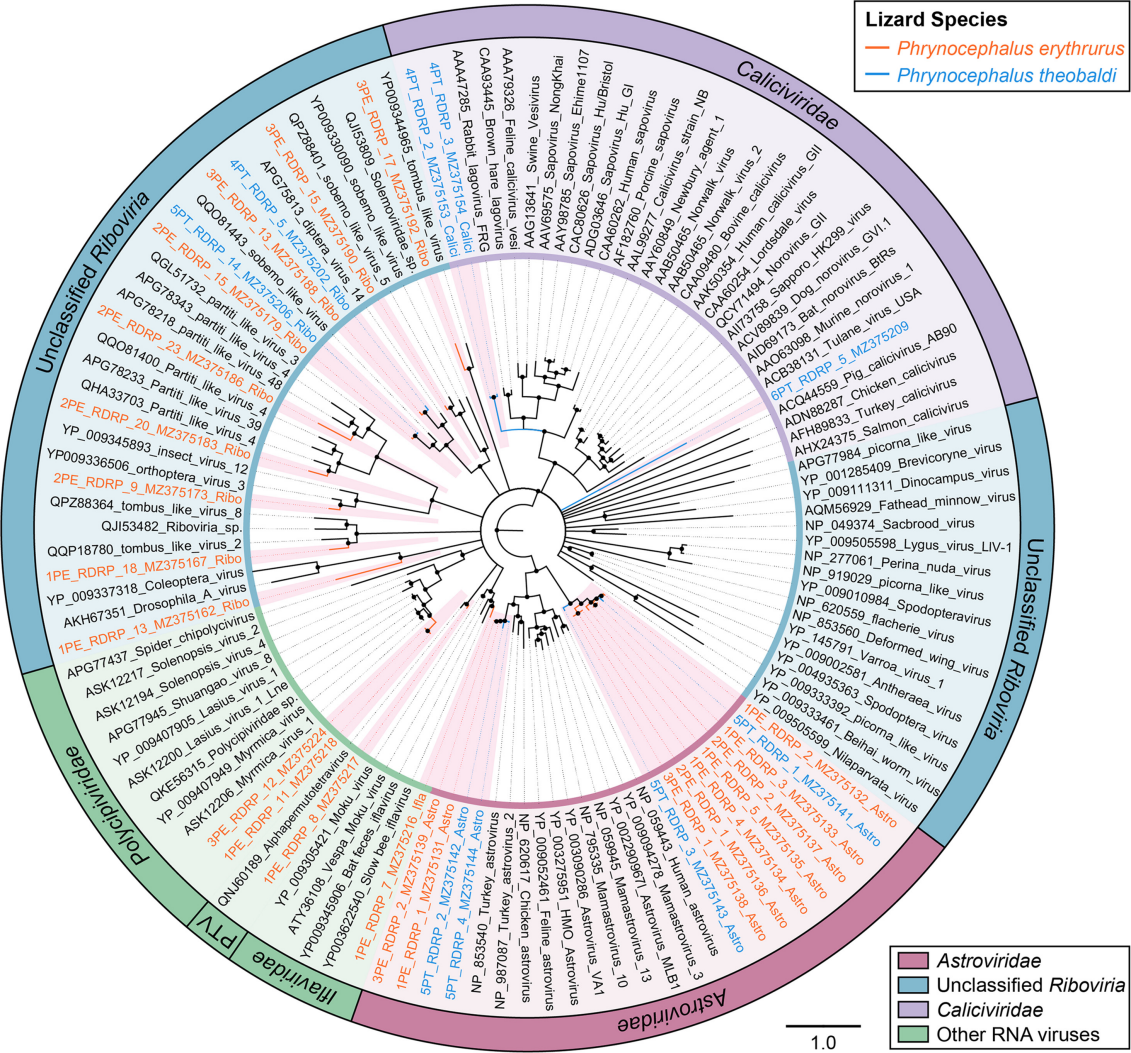
The phylogeny of RNA viruses identified in feces of lizards. Bayesian inference tree established based on amino acid sequences of RdRp protein of RNA viruses. The viruses found in this study are marked with colored lines and red-filled sectors. Different viral groups are marked with color coding shown in the key on the bottom right. The sizes of the black dots on the nodes are positively correlated with the corresponding bootstrap scores. The scale bar indicates the amino acid substitutions. PTV, *Permutotetraviridae*.

However, phylogenetic analyses of other ssDNA viruses and RNA viruses presented a different picture, in which most of the sequences were closely clustered with known viral strains. As presented in the phylogenetic tree of replication protein (Rep) sequences for CRESS DNA viruses, the two sequences from P. erythrurus were both phylogenetically clustered with known viral strains in the clade of unclassified CRESS DNA viruses, sharing >60% Rep protein sequence identity (Fig. 6a). Similarly, the tree based on NS1 for *Parvoviridae* also indicated that the nine sequences all showed a close relationship to previous viral species (Fig. 6b). Two of them were closely clustered with bocaparvoviruses that were identified from rodents and rabbits, and seven showed close relationships with ambidensoviruses from a wide range of invertebrates. Moreover, the dendrogram of RdRp proteins showed that the 37 sequences of RNA viruses were all clustered with known viral species, including caliciviruses and astroviruses, which infect vertebrates, and iflaviruses and alphapermutotetraviruses, which infect invertebrates (Fig. 7).

### Functional analysis of phage genes.

**FIG 8:**
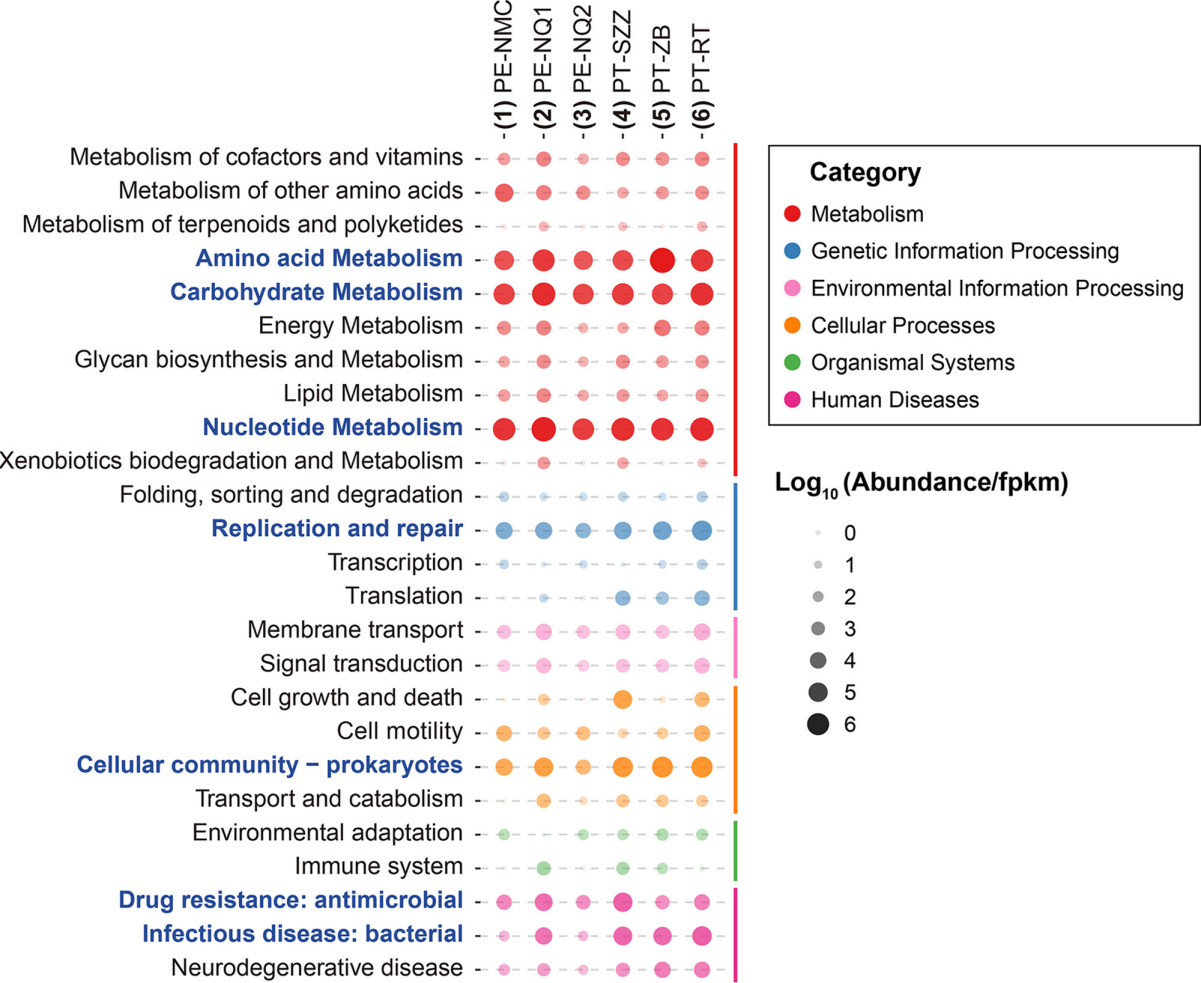
Abundances of functional genes in phage contigs from the six viromes. Bubble chart presenting the functional annotation results for putative proteins in phage contigs based on the KEGG pathway database. The sizes of the bubbles are positively correlated with the abundances of the genes. Pathway subcategories mentioned in the text are highlighted in blue font.

Considering that a large number of diverse bacteriophages were identified in the study and phages are capable of controlling and impacting bacterial hosts, we annotated the predicted open reading frames (ORFs) in phage contigs using the KEGG database to explore the latent functions of phages (Fig. 8; Table S3). The majority of ORFs were annotated with metabolism functions, such as amino acid, carbohydrate, and nucleotide metabolism. As for the genetic information processing category, functional genes related to replication and repair were the largest proportion. Moreover, it is worth mentioning that there were considerable numbers of genes directly correlated with the bacterial hosts, including those classified into prokaryote cellular community, antimicrobial drug resistance, and bacterial infectious disease categories, indicating the host-regulating function of bacteriophages in the lizard gut.

### Diversity and abundance of phage ARGs.

**FIG 9:**
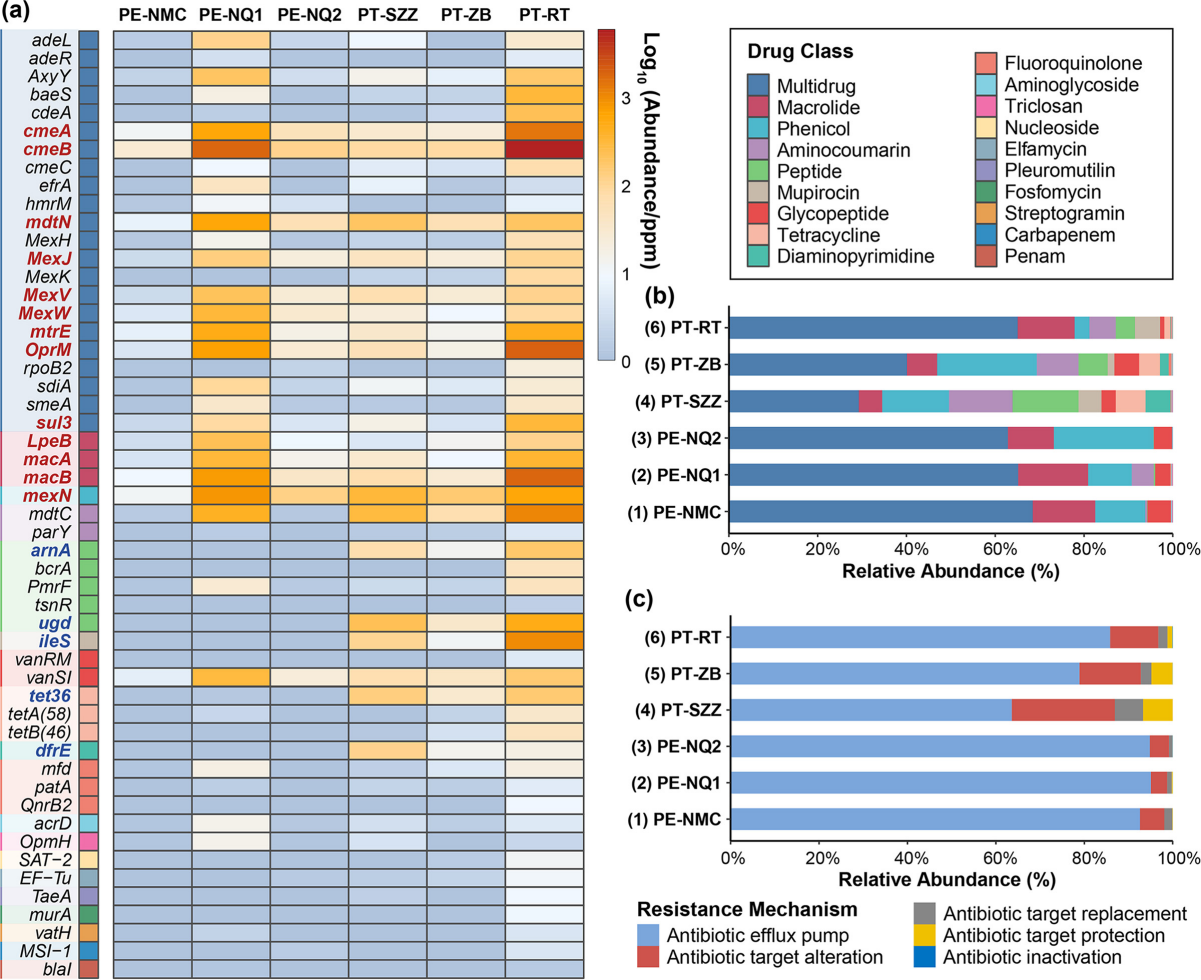
Abundances of ARGs in phage contigs from the six viromes. (a) Heatmap depicting the abundances of dominant ARGs in the six viromes. Drug classes are indicated with the color coding shown in the key on the upper right. The ARGs exclusive to an individual lizard species are marked in blue font, and the ARGs existing in all six viromes are marked in red font. (b) Bar plot showing the relative abundances of different drug classes of ARGs. (c) Bar plot showing the proportions of different resistance mechanisms of ARGs in the six viromes.

Bacteriophage-mediated transduction is an indispensable route for the dissemination of ARGs, and thus, phages might be a crucial reservoir of ARGs. To explore the functions of phages, the potential ARGs in phage contigs were identified using BLASTp against the Comprehensive Antibiotic Resistance Database (CARD). A total of 146 predicted ORFs were annotated as the corresponding 52 ARGs classified into 19 drug classes (Fig. 9a; Table S4). The predominant antibiotic drug class was multidrug (62.70 to 11,406.68 ppm), followed by macrolide (12.90 to 2,248.56 ppm) and phenicol (10.31 to 816.55 ppm) (Fig. 9b), and 13 ARGs were widely detected in all six viromes. The most abundant gene was *cmeB* (26.77 to 5,781.76 ppm), followed by *macB* (8.28 to 1,778.52 ppm), *oprM* (3.33 to 1,887.22 ppm), *cmeA* (10.87 to 1,368.48 ppm), etc. In addition, five ARGs were exclusive to the lizard species Phrynocephalus theobaldi, including *arnA* (11.95 to 116.15 ppm), *ugd* (31.87 to 498.60 ppm), *iles* (10.33 to 997.12 ppm), *tet36* (28.33 to 152.27 ppm), and *dfrE* (13.43 to 113.04 ppm), indicating a unique composition of ARGs in the lizard species (Fig. 9a). The relative abundances of resistance mechanisms in the six viromes are presented in Fig. 9c. Antibiotic efflux pumps formed the largest proportion of resistance mechanisms throughout the six viromes, ranging from 63.59% to 95.03%, followed by antibiotic target alteration (∼3.70% to 23.32%), and the other three mechanisms were all less than 10%.

## DISCUSSION

The virome is an integral part of the microbiome that can provide both positive and negative effects during coevolution with prokaryotic and eukaryotic hosts (13). Considering the inhospitable environment in the QTP and the rarity of highland lizards, this study was carried out to explore the viral community structure in two endangered lizard species and provide clues to reveal the underlying function of phages in the gut virome, in order to provide a foundation for protecting threatened species.

The viral communities in the six virome were all predominated by bacteriophages, especially the *Caudovirales* order, mainly including *Myoviridae*, *Podoviridae*, and *Siphoviridae* families. In addition, another bacteriophage family, *Microviridae*, also accounted for a definite proportion of each of the six viromes. These results demonstrated that phages accounted for the overwhelming proportion of the lizard gut virome, consistent with the gut viromes in honeybees, birds, and mammals (25, 26). Furthermore, the conserved TerL and MCP genes of the *Caudovirales* order and *Microviridae* family all presented high genetic diversity that clustered phylogenetically between clades of classified species and even into several novel clades. The scenario is similar to that of the bacteriophages in the human gut virome, which possess a predominant virome core containing highly diverse viral sequences from *Caudovirales* and *Microviridae* (27). Therefore, although bacteriophages are characterized by the simplest structures and the smallest sizes, phages possess the highest abundance and the greatest diversity in gut viromes.

The viral community structures of the two lizard species presented no significant differences. Gut microbiomes are subject to multiple factors, such as living conditions (28) and eating patterns (29), and environments play a greater role in shaping gut microbiomes than host genetic backgrounds (30). Given the bleak and inhospitable characteristics of the plateau, it came as no surprise that the species factor alone would make no difference. Although the two sample groups collected from the Naqu area did not cluster closely together in PCoA, there was no statistically significant difference between the two groups, and the differences could be explained by individual variations and environmental factors. In addition, it should be taken into consideration that the virome from P. erythrurus lizards living around the Namtso Lake possessed a unique viral community structure. It had the highest number of viral species and contained more viruses belonging to the *Iflaviridae*, *Permutotetraviridae*, and *Parvoviridae* families, which infect a wide range of invertebrates, because the lake area was not only a natural habitat for many insects and other prey of lizards but also a tourist attraction with numerous visitors. And the UPGMA dendrogram obviously separated this virome from the others, further highlighting the role of environmental factors in the regulation of viral communities.

Several vertebrate-infecting viruses were discovered in the study, including caliciviruses and astroviruses, which are potentially pathogenic for lizards. Astroviruses and caliciviruses both are nonenveloped, positive-sense, single-stranded RNA viruses and infect a wide range of vertebrates, causing viral gastroenteritis accompanied by various symptoms, including diarrhea, listlessness, nervousness, loss of appetite, etc. However, astroviruses mainly infect avian and mammalian animal species, and there are no related reports about lizard-infecting astroviruses (31). Although caliciviruses have been found in other reptiles, such as snakes, the infected snakes presented no correlated symptoms of gastroenteritis (32). Given that the lizard subjects in the study were also seemingly asymptomatic, the pathogenicity and transmissibility of those viruses remain to be further studied.

Bacteriophages are capable of mediating the transduction of genetic material in bacterial communities, affecting and controlling the phenotypes of their bacterial hosts (16). Considerable functional gene reserves have been discovered in bacteriophages, contributing to the evolution of their hosts, including metabolic genes and virulence genes (33). In this study, the majority of phage-associated functional genes were classified into the energy metabolism and DNA repair subcategories. Native animals living in high-altitude regions are forced to generate adaptive evolution in order to survive in low-temperature and hypoxic environments. Functional genes related to nutrient metabolism and DNA repair are also enriched in highland-dwelling humans and other mammals, providing potential genetic mechanisms of highland adaptation (8, 9, 34). However, the lack of lowland-dwelling species of lizards in this study hindered comparative research based on different altitudes, and thus, the phenomena could not simply be attributed to the factor of elevation. Further research would be needed to identify the adaptive responses to extreme environments.

Bacteriophages can transfer ARGs through transduction, facilitating the evolution of drug-resistant bacteria (17). Previous research demonstrates that bacteriophages in both densely populated and sparsely populated areas store numerous ARGs, which are associated with the level of anthropogenic activities (35, 36). In the present study, most of the ARGs were classified into the multidrug category, and few of them encoded β-lactam resistance, whereas β-lactam resistance genes have been identified as a predominant resistance type in phage viromes from multiple animals and environments, such as livestock (37), poultry (20), agricultural soil (38), and wastewater (21). Thus, it is probably because the Plateau is a desolate area with a low urbanization level and would not be affected by contamination with common antibiotics that these highland-dwelling lizard species might possess a different composition of ARGs. In addition, some ARGs were exclusive to the virome from P. theobaldi, revealing that those genes might be correlated with the lizard species. Previous research identified the ARGs in different chicken cecum microbiomes and found that the lincosamide-streptogramin-macrolide (LSM) resistance genes were more abundant in one chicken group (39). Thus, the uniqueness of ARGs in different species deserves further investigation.

In conclusion, this study first explored the structure and function of viral communities in two endangered lizard species, P. erythrurus and P. theobaldi, inhabiting the Qinghai-Tibet Plateau. The lizard gut viromes were predominated by a wide variety of bacteriophages, which included plenty of functional genes associated with energy metabolism and antibiotic resistance. Because of the rarity of the lizard species and the difficulty of sampling in the highland, sample sizes are inevitably limited in the study. Even so, our research contributed to a better understanding of viromes in highland lizard species, encouraging more research to focus on these vulnerable species and other cold-blooded and highland-dwelling animals in the future.

## MATERIALS AND METHODS

### Sample collection and preparation.

In July 2020, to investigate the viromes of two lizard species (Phrynocephalus erythrurus and Phrynocephalus theobaldi) living in the Qinghai-Tibetan Plateau, a total of 81 fecal samples from the two lizard species were collected from five sampling sites (Namtso, Naqu, Sangzhuzi, Zhongba, and Rutog) with an average altitude of over 4,000 m (Fig. 1; Table S1). All samples were collected using disposable materials and shipped on dry ice. Fecal samples were pooled in six sample pools according to lizard species and sampling sites. Samples were resuspended in 10 volumes of phosphate-buffered saline (PBS) and vigorously vortexed for 5 min and then freeze-thawed three times on dry ice. The supernatants were then collected after centrifugation (10 min at 15,000 × *g*) and stored at −80°C until use.

### Viral metagenomic library construction.

Five hundred microliters of each supernatant was filtered through a 0.45-μm filter (Millipore) to remove eukaryotic- and bacterial-cell-sized particles. The filtrates enriched in viral particles were treated with DNase and RNase to digest unprotected nucleic acid at 37°C for 60 min (40–42). Then, the remaining total nucleic acid was isolated using a QIAamp viral RNA minikit (Qiagen) according to the manufacturer’s protocol. For library construction, dsDNA was synthesized from viral RNA and DNA. For RNA viruses, a reverse transcription kit (SuperScript III reverse transcriptase) was used for reverse transcribing RNA into cDNA, and then the DNA polymerase I large fragment (Klenow fragment) was added to synthesize the second strand of cDNA (dsDNA). Specifically, 12 μL of nucleic acid extracts was added to the reaction mixture to synthesize dsDNA (total reaction mixture volume, 20 μL). For ssDNA viruses, ssDNA was converted to dsDNA using the Klenow reaction and all the dsDNA products were used to construct libraries. Overall, six libraries, along with a control library, were then constructed using a Nextera XT DNA sample preparation kit (Illumina), and the quality was inspected using agarose gel electrophoresis and the Agilent bioanalyzer 2100. All libraries were sequenced on an Illumina NovaSeq 6000 platform with 250-bp paired-end reads with dual barcoding for each sample pool (43). The total read number of each library is presented in Table S1.

### Bioinformatics analysis.

Paired-end reads of 250 bp generated by NovaSeq were debarcoded using vendor software from Illumina. An in-house analysis pipeline running on a 32-node Linux cluster was utilized to process the data. Reads were considered duplicates if bases 5 to 55 were identical, and only one randomly chosen copy of any pair of duplicates was kept. Low-sequencing-quality tails were trimmed using a Phred quality score of 10 as the threshold. Adaptors were trimmed using the default parameters of VecScreen, which is based on NCBI BLASTn with specialized parameters designed for adapter removal. The cleaned reads were *de novo* assembled within each barcode, and the chimeras among them were detected. Then, the reads were filtered by length using the ENSEMBLE assembler with the default parameters (44). Contigs and singlet reads were then matched against a customized viral proteome database using BLASTx with an E-value cutoff of <10^−5^. The virus BLASTx database was compiled using the NCBI virus reference proteome (ftp://ftp.ncbi.nih.gov/refseq/release/viral/) and viral protein sequences from NCBI nr FASTA files (based on annotation taxonomy in Virus Kingdom). Candidate viral hits were then compared to an in-house nonvirus nonredundant (NVNR) protein database with an E-value cutoff of <10^−5^ to remove false-positive viral hits. The NVNR database was compiled using nonviral protein sequences extracted from NCBI nr FASTA files (based on annotation taxonomy excluding Virus Kingdom). Contigs without significant BLASTx similarity to the viral proteome database were searched against viral protein families in the vFam database (45) using HMMER3 (46–48) to detect remote viral protein similarities.

### Viral community analysis.

Composition similarity analysis of the six viromes was performed using MEGAN software (MEtaGenome Analyzer, version 6.21.7) (49) under the compare option. The results were presented by constructing an unweighted pair group method with arithmetic mean (UPGMA) taxonomic tree and using canonical correspondence analysis (CCA) under the cluster analysis option and the Bray-Curtis ecological distance matrix with default parameters. Analysis of similarities (ANOSIM) was used to compare differences between groups in viral communities using the R version 4.0.4 package vegan (version 2.5-7; https://CRAN.R-project.org/package=vegan). The viral community structure and richness results were visualized in a heatmap and UpSet plot, which were generated using the R version 4.0.4 package pheatmap (version 1.0.12; https://CRAN.R-project.org/package=pheatmap) and UpSetR (version 1.4.0; https://CRAN.R-project.org/package=UpSetR), respectively.

### Viral sequence extension and annotation.

Viral contigs that might have been from the same genome but without overlaps were merged using the Low Sensitivity/Fastest parameter in Geneious software version 11.1.2 (50). The individual contigs were used as the reference for mapping to the raw reads of their original barcodes using the Low Sensitivity/Fastest parameter. Putative viral open reading frames (ORFs) were predicted by using Geneious version 11.1.2 with built-in parameters (minimum size, 300; genetic code, standard; start codons, ATG) (50) and were further checked through comparison to related viruses by BLASTp in NCBI. The annotations of these ORFs were based on comparisons to the Conserved Domain Database using RPS-BLAST with an E-value cutoff of <10^−5^. Those contigs annotated with virus hallmark genes of the main virus groups were selected, among which those identified as complete ORFs were included for further phylogenetic analyses (the virus hallmark genes used were MCP for *Microviridae*, NS1 for *Parvoviridae*, Rep for CRESS DNA viruses, TerL for *Caudovirales*, and RdRp for *Riboviria*). All sequences with virus hallmark genes are presented in scatterplots drawn using R package ggplot2 (version 3.2.1; https://ggplot2.tidyverse.org).

### Phylogenetic analysis.

Phylogenetic analyses were performed based on the predicted protein sequences of virus hallmark genes identified in this study and protein sequences of reference strains belonging to different groups of viruses that were downloaded from the NCBI GenBank database. Related protein sequences were aligned using MUSCLE in MEGA version 10.1.8 (51) with the default settings. Sites containing more than 50% gaps were temporarily removed from alignments. Bayesian inference trees were then constructed using MrBayes version 3.2.7 (52). The Markov chain was run for a maximum of 1 million generations, sampled every 50 generations, and the first 25% of Markov chain Monte Carlo (MCMC) samples were discarded as burn-in. Maximum-likelihood trees were also constructed to confirm all the Bayesian inference trees, using MEGA software version 10.1.8 (51).

### Phage ARG characterization and functional annotation.

Contigs and singlet reads classified as phage sequences in the previous taxonomy annotation based on BLASTx were included for further ARG characterization and functional annotation. The ORF predictions for the phage sequences were measured in VirSorter with default settings (53). Then, the ORFs were used as queries to search for ARGs and functional genes using BLASTp against the Comprehensive Antibiotic Resistance Database (CARD) (54) and Kyoto Encyclopedia of Genes and Genomes (KEGG) database with an E-value cutoff of <10^−5^. To quantify gene abundance, raw sequence reads were aligned to the ORFs using Bowtie 2 with default parameters (55). The numbers of read hits were then normalized to the library size and the coding length of each gene, manifested as parts per million (ppm) or fragments per kilobase per million (FPKM) (56, 57). The annotation information and the abundances of gene hits in each library are shown in Tables S3 and S4.

### Quality control.

As a blank control, sterile double-distilled water (ddH_2_O; Sagon, Shanghai, China) was prepared simultaneously and further processed under the same conditions. Particular attention was given to minimizing the risk of cross contamination and nucleic acid degradation. Aerosol filter pipet tips were used to avoid possible cross contamination among samples. All experimental materials (including microcentrifuge tubes, pipet tips, etc.) that directly contacted nucleic acid samples were RNase and DNase free, and all nucleic acid samples were dissolved in diethyl pyrocarbonate (DEPC)-treated water with RNase inhibitors added. All experimental processes were performed in a biological safety cabinet.

### Data availability.

The raw sequence read data analyzed in this study are available at the National Center for Biotechnology Information (NCBI) Sequence Read Archive database under accession numbers SRX11831358, SRX11831382, SRX11831397, SRX11831406, SRX11831413, SRX11831441, and SRX11870785 (control library). All viral sequences with virus hallmark genes identified in this study were deposited in the GenBank database under the accession numbers MZ374764, MZ375112, MZ375114, and MZ375229.
