# Supplementary material for: Gut Virome of the World’s Highest-Elevation Lizard Species (Phrynocephalus erythrurus and Phrynocephalus theobaldi) Reveals Versatile Commensal Viruses
Source: Microbiol Spectr. 2022 Feb 23;10(1):e01872-21. doi: 10.1128/spectrum.01872-21 (PMC8865479; doi:10.1128/spectrum.01872-21)
Supplement: SUPPLEMENTAL FILE 1 — Supplemental material. Download SPECTRUM01872-21_Supp_1_seq13.pdf, PDF file, 1.6 MB [file spectrum01872-21_supp_1_seq13.pdf]

Supplementary Information

Gut virome of the world's highest lizard species (*Phrynocephalus erythrurus* and *Phrynocephalus theobaldi*) reveals versatile commensal viruses

Juan Lu[1#], Shixing Yang[1#], Chunmei Wang[2#], Hao Wang[3#], Ga Gong[4#], Yuan Xi[1], Jiamin Pan[1], Xiaochun Wang[1],

Jian Zeng[1], Ju Zhang[1], Peng Li[5*], Quan Shen[1*], Tongling Shan[2*], Wen Zhang[1*]

[1]Department of Microbiology, School of Medicine, Jiangsu University, Zhenjiang, Jiangsu 212003, China

[2]Shanghai Veterinary Research Institute, Chinese Academy of Agricultural Sciences, Shanghai 200241, China

[3]Department of Clinical Laboratory, Huai'an Hospital, Xuzhou Medical University, Huai'an, Jiangsu 223002, China

[4]Animal Science College, Tibet Agriculture and Animal Husbandry University, Nyingchi, Tibet 860000, China

[5]Jiangsu Key Laboratory for Biodiversity and Biotechnology, College of Life Sciences, Nanjing Normal University, Nanjing 210023, Jiangsu, China

---

*Corresponding authors. Peng Li, lipeng@njnu.edu.cn; Quan Shen, shenquan@ujs.edu.cn; Tongling Shan, shantongling@shvri.ac.cn; Wen Zhang, z0216wen@yahoo.com.
[#]These authors contributed equally to this work.

Figure S1 Length and identity distribution of sequences with virus hallmark genes in the six viromes.

Table S1 Information of sampling sites and corresponding libraries.

Table S2 Information of viral sequences with virus hallmark genes identified in lizards.

Table S3 The results of functional annotation of phage-encoded genes based on KEGG database.

Table S4 The results of ARGs annotation of phage-encoded genes based on CARD database.

Figure S1 Length and identity distribution of sequences with virus hallmark genes in the six viromes. The horizontal axis above shows the library names and the right vertical axis shows the types of virus hallmark genes. The horizontal axis below indicates the sequence length and the left vertical axis indicates sequence identity based on comparison between the 465 viral sequences in this study and their best matches in BLASTx search, respectively. Red dots represent the sequences with complete CDS selected for further phylogenetic analysis, and other sequences are marked with gray dots.

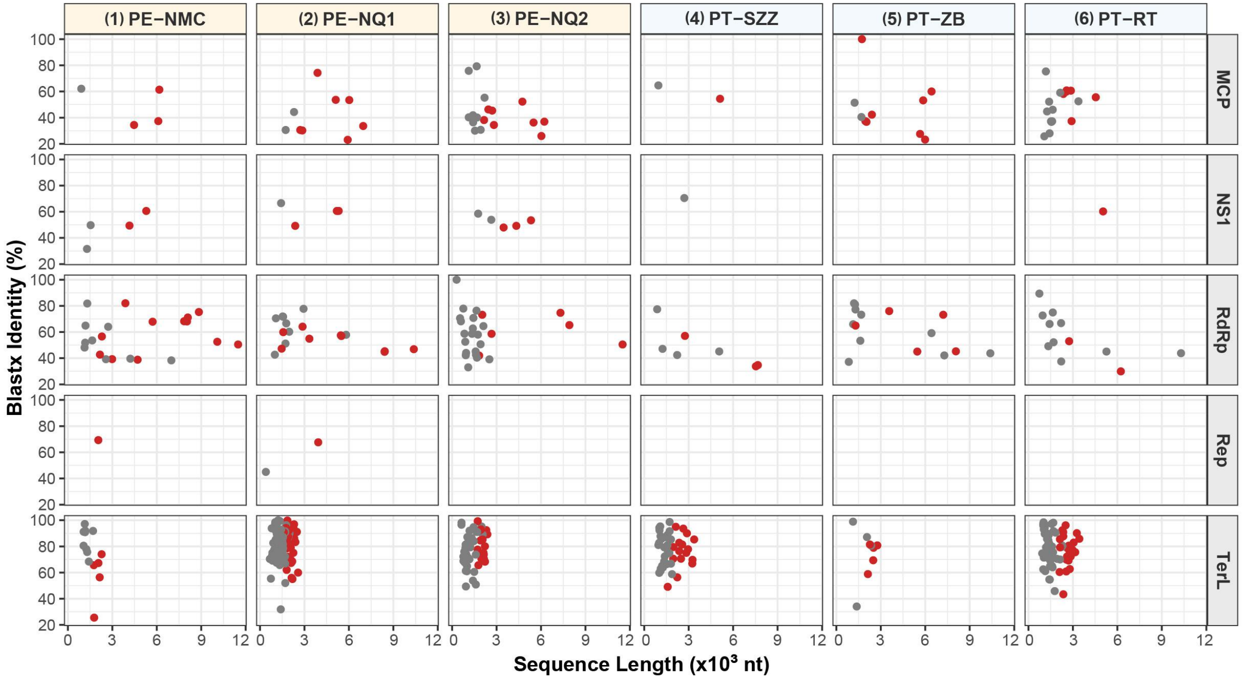

Table S1. Information of sampling sites and corresponding libraries.

Sample ID	Library ID	SRA Accession No.	Lizard Species	Sampling Site	Location	Altitude	Sampling Date	Sampling Number	Library Total Reads	Average Reads Length	GC%	No. of Viral Contigs	Min contigs length	Max contigs length	Average Contigs Length	Percentage of reads mapped to Viral Contigs
(1) PE-NMC	lizardfe001	SRX11831358	Phrynocephalus erythrurus	Namtso, Lhasa, Tibet, China	30.68 N, 90.87 E	4784 m	23-Jul-2020	17	21,625,256	248.0	42.9	24,275	200	15,121	495	42.33%
(2) PE-NQ1	lizardfe002	SRX11831382	Phrynocephalus erythrurus	Seni, Naqu, Tibet, China	31.51 N, 92.09 E	4592 m	25-Jul-2020	21	18,140,872	248.3	46.5	90,322	200	45,723	592	50.62%
(3) PE-NQ2	lizardfe003	SRX11831397	Phrynocephalus erythrurus	Seni, Naqu, Tibet, China	31.51 N, 92.09 E	4592 m	25-Jul-2020	9	14,760,460	243.0	42.5	30,854	200	34,792	532	59.81%
(4) PT-SZZ	lizardfe004	SRX11831406	Phrynocephalus theobaldi	Sangzhuzi, Shigatse,Tibet, China	29.32 N, 89.33 E	3821 m	31-Jul-2020	2	12,871,580	248.5	44.7	35,960	200	98,376	583	39.26%
(5) PT-ZB	lizardfe005	SRX11831413	Phrynocephalus theobaldi	Zhongba, Shigatse,Tibet, China	29.74 N, 84.04 E	4575 m	29-Jul-2020	10	6,777,440	242.2	46.3	25,682	200	79,725	514	39.31%
(6) PT-RT	lizardfe006	SRX11831441	Phrynocephalus theobaldi	Rutog, Shigatse,Tibet, China	33.12 N, 80.24 E	4373 m	30-Jul-2020	22	12,464,890	249.6	49.0	115,132	200	88,091	610	39.97%
Blank Control	Blank Control	SRX11870785	—	—	—	—	—	—	14,162	237.4	56.0	0	—	—	—	—

Table S2. Information of viral sequences with virus hallmark genes identified in lizards.

Virus strain name	GenBank No.	LibraryID	SRA Accession No.	Length	Virus Reads	Virus hallmark genes	Completeness of CDS	Classification	Blastx hits on known protein and viruses	Coverage (%)	E-value	Identity (%)	Accession no. of best match
1PE-MCP-1	MZ374764	lizardfe001	SRX11831358	6171	800	major capsid protein	complete	Microviridae	hypothetical protein [Bacteroides xylanisolvens]	99%	0	61.37%	WP_141409247.1
1PE-MCP-2	MZ374765	lizardfe001	SRX11831358	6101	28538	major capsid protein	complete	Microviridae	major capsid protein [Microvirus sp.]	99%	3E-148	37.35%	WP_195578815.1
1PE-MCP-3	MZ374766	lizardfe001	SRX11831358	4466	99	major capsid protein	complete	Microviridae	major capsid protein [Capybara microvirus Cap1_SP_83]	99%	4E-108	34.39%	QRV61933.1
1PE-MCP-4	MZ374767	lizardfe001	SRX11831358	907	18	major capsid protein	partial	Microviridae	major capsid protein [Microviridae sp.]	99%	4E-103	62.08%	AXF52770.1
1PE-NS1-1	MZ375116	lizardfe001	SRX11831358	5290	2086269	nonstructual protein 1	complete	Parvoviridae	NS1 [Ambidensovirus sp.]	99%	0	60.60%	QGZ11177.1
1PE-NS1-2	MZ375117	lizardfe001	SRX11831358	4158	460	nonstructual protein 1	complete	Parvoviridae	nonstructural protein [Melipona quadrifasciata densovirus]	97%	4E-166	49.42%	QCE20585.1
1PE-NS1-3	MZ375118	lizardfe001	SRX11831358	1537	105	nonstructual protein 1	partial	Parvoviridae	NS1 [Murine bocavirus]	95%	5E-143	49.76%	AIY27465.1
1PE-NS1-4	MZ375119	lizardfe001	SRX11831358	1294	19	nonstructual protein 1	partial	Parvoviridae	hypothetical protein [Parvoviridae sp.]	98%	1E-48	31.58%	AAM28943.1
1PE-RDRP-1	MZ375131	lizardfe001	SRX11831358	8840	23224	RNA dependent RNA polymerase	complete	Astroviridae	ORF1ab [Hainan oriental leaf-toed gecko astrovirus 1]	99%	0	75.29%	AVM87517.1
1PE-RDRP-2	MZ375132	lizardfe001	SRX11831358	8094	60853	RNA dependent RNA polymerase	complete	Astroviridae	ORF1ab [Guangxi changeable lizard astrovirus]	82%	2E-144	71.10%	AVM87190.1
1PE-RDRP-3	MZ375133	lizardfe001	SRX11831358	8038	141314	RNA dependent RNA polymerase	complete	Astroviridae	ORF1ab [Guangxi changeable lizard astrovirus]	86%	0	68.08%	AVM87190.1
1PE-RDRP-4	MZ375134	lizardfe001	SRX11831358	7845	37220	RNA dependent RNA polymerase	complete	Astroviridae	ORF1ab [Guangxi changeable lizard astrovirus]	85%	0	68.25%	AVM87190.1
1PE-RDRP-5	MZ375135	lizardfe001	SRX11831358	5719	88	RNA dependent RNA polymerase	complete	Astroviridae	ORF1ab [Guangxi changeable lizard astrovirus]	87%	0	67.89%	AVM87190.1
1PE-RDRP-7	MZ375216	lizardfe001	SRX11831358	10082	4408	RNA dependent RNA polymerase	complete	Iflaviridae	polyprotein [Moku virus]	89%	0	52.55%	YP_009305421.1
1PE-RDRP-8	MZ375217	lizardfe001	SRX11831358	3880	1018777	RNA dependent RNA polymerase	complete	Permutotetraviridae	RNA-dependent RNA polymerase [Alphapermutotetravirus sp.]	99%	0	82.00%	QNJ60189.1
1PE-RDRP-11	MZ375218	lizardfe001	SRX11831358	11486	14836	RNA dependent RNA polymerase	complete	Polycipiviridae	RNA-dependent RNA polymerase [Lasius neglectus virus 1]	99%	0	50.48%	YP_009407905.1
1PE-RDRP-12	MZ375161	lizardfe001	SRX11831358	6982	745	RNA dependent RNA polymerase	partial	unclassified Riboviria	hypothetical protein [Hubei coleoptera virus 2]	98%	0	38.33%	YP_009337178.1
1PE-RDRP-13	MZ375162	lizardfe001	SRX11831358	4706	1575896	RNA dependent RNA polymerase	complete	unclassified Riboviria	RdRp [Hubei permutotetra-like virus 6]	71%	2E-137	38.83%	YP_009337318.1
1PE-RDRP-14	MZ375163	lizardfe001	SRX11831358	2988	145	RNA dependent RNA polymerase	complete	unclassified Riboviria	RNA-dependent RNA polymerase [Merch tombus-like virus]	73%	4E-129	39.29%	QIJ70127.1
1PE-RDRP-15	MZ375164	lizardfe001	SRX11831358	2721	1024	RNA dependent RNA polymerase	partial	unclassified Riboviria	hypothetical protein 2 [Wenzhou sobemo-like virus 4]	100%	4E-124	64.02%	QJI53807.1
1PE-RDRP-16	MZ375165	lizardfe001	SRX11831358	2570	146	RNA dependent RNA polymerase	partial	unclassified Riboviria	RNA-dependent RNA polymerase [Hubei orthoptera virus 4]	95%	0	39.22%	APG76402.1
1PE-RDRP-17	MZ375166	lizardfe001	SRX11831358	2297	363	RNA dependent RNA polymerase	complete	unclassified Riboviria	RNA-dependent RNA polymerase [Gervais tombus-like virus]	99%	0	56.59%	QIJ70125.1
1PE-RDRP-18	MZ375167	lizardfe001	SRX11831358	2166	958	RNA dependent RNA polymerase	complete	unclassified Riboviria	RNA-dependent RNA polymerase [Soybean thrips tombus-like virus 2]	99%	2E-108	42.73%	QJI53482.1
1PE-RDRP-19	MZ375168	lizardfe001	SRX11831358	1644	73	RNA dependent RNA polymerase	partial	unclassified Riboviria	RdRp [Hubei partiti-like virus 11]	98%	0	53.58%	YP_009329875.1
1PE-RDRP-20	MZ375169	lizardfe001	SRX11831358	1310	50	RNA dependent RNA polymerase	partial	unclassified Riboviria	hypothetical protein 2 [Hubei sobemo-like virus 48]	100%	0	81.79%	YP_009330080.1
1PE-RDRP-21	MZ375170	lizardfe001	SRX11831358	1191	95	RNA dependent RNA polymerase	partial	unclassified Riboviria	RdRp [Hubei partiti-like virus 48]	97%	0	64.91%	APG78218.1
1PE-RDRP-22	MZ375171	lizardfe001	SRX11831358	1168	28	RNA dependent RNA polymerase	partial	unclassified Riboviria	RdRp [Hubei partiti-like virus 39]	98%	1E-105	51.86%	APG78233.1
1PE-RDRP-23	MZ375172	lizardfe001	SRX11831358	1128	812	RNA dependent RNA polymerase	partial	unclassified Riboviria	hypothetical protein 3 [Hubei tombus-like virus 40]	99%	4E-112	48.12%	YP_009336561.1
1PE-RDRP-25	MZ375219	lizardfe001	SRX11831358	4217	386	RNA dependent RNA polymerase	partial	Tombusviridae	RNA-dependent RNA polymerase [Merch tombus-like virus]	83%	5E-154	39.61%	QIJ70127.1
1PE-REP-1	MZ375112	lizardfe001	SRX11831358	2055	700	replication-associated protein	complete	unclassified CRESS DNA virus	replication-associated protein [Giardia-associated CRESS DNA virus 3]	93%	1E-152	69.31%	QNJ47554.1
1PE-TERL-1	MZ374824	lizardfe001	SRX11831358	2279	11	terminase large subunit	complete	uncultured Caudovirales phage	terminase [Oscillibacter sp.]	99%	0	74.01%	MBD5169250.1
1PE-TERL-2	MZ374825	lizardfe001	SRX11831358	2149	18	terminase large subunit	complete	uncultured Caudovirales phage	hypothetical protein [Clostridia bacterium]	94%	0	56.37%	MBR6688796.1
1PE-TERL-3	MZ374826	lizardfe001	SRX11831358	2063	76	terminase large subunit	complete	uncultured Caudovirales phage	terminase [Ruminococcus sp. OM08-9BH]	95%	0	67.29%	WP_118064159.1
1PE-TERL-4	MZ374827	lizardfe001	SRX11831358	1776	119	terminase large subunit	complete	uncultured Caudovirales phage	hypothetical protein [Thermosipho sp. (in: Bacteria)]	56%	0.00000006	25.55%	MBO8161331.1
1PE-TERL-5	MZ374828	lizardfe001	SRX11831358	1755	13	terminase large subunit	complete	uncultured Caudovirales phage	hypothetical protein EII22_08935 [Coriobacteriales bacterium OH1046]	97%	0	65.63%	RVU97048.1
1PE-TERL-6	MZ374829	lizardfe001	SRX11831358	1699	13	terminase large subunit	partial	uncultured Caudovirales phage	terminase large subunit [Parabacteroides sp. AF19-14]	99%	0	91.83%	WP_122379962.1
1PE-TERL-7	MZ374830	lizardfe001	SRX11831358	1417	38	terminase large subunit	partial	uncultured Caudovirales phage	hypothetical protein [Slackia faecicanis]	98%	0	68.42%	WP_123197916.1
1PE-TERL-8	MZ374831	lizardfe001	SRX11831358	1305	10	terminase large subunit	partial	uncultured Caudovirales phage	terminase [Firmicutes bacterium]	100%	0	75.70%	PWM71933.1
1PE-TERL-9	MZ374832	lizardfe001	SRX11831358	1248	44	terminase large subunit	partial	uncultured Caudovirales phage	terminase large subunit [[Clostridium] methoxybenzovorans]	98%	0	77.88%	WP_024348198.1
1PE-TERL-10	MZ374833	lizardfe001	SRX11831358	1175	12	terminase large subunit	partial	uncultured Caudovirales phage	terminase large subunit [Bacteroides uniformis]	99%	0	92.00%	WP_117959321.1
1PE-TERL-11	MZ374834	lizardfe001	SRX11831358	1164	12	terminase large subunit	partial	uncultured Caudovirales phage	terminase large subunit [Blautia sp. OF03-15BH]	98%	0	90.88%	WP_117768406.1
1PE-TERL-12	MZ374835	lizardfe001	SRX11831358	1142	51	terminase large subunit	partial	uncultured Caudovirales phage	MULTISPECIES: terminase [unclassified Anaerotruncus]	99%	0	97.15%	WP_120484317.1
1PE-TERL-13	MZ374836	lizardfe001	SRX11831358	1066	12	terminase large subunit	partial	uncultured Caudovirales phage	terminase large subunit [Bacteroides uniformis]	99%	0	91.19%	WP_117959321.1
1PE-TERL-14	MZ374837	lizardfe001	SRX11831358	1062	14	terminase large subunit	partial	uncultured Caudovirales phage	terminase large subunit [Mogibacterium sp. BX12]	94%	0	80.54%	WP_187304421.1
2PE-MCP-1	MZ374768	lizardfe002	SRX11831382	6964	916	major capsid protein	complete	Microviridae	major capsid protein [Microvirus sp.]	99%	2E-86	33.59%	MBO5632558.1
2PE-MCP-2	MZ374769	lizardfe002	SRX11831382	6024	1093	major capsid protein	complete	Microviridae	major capsid protein [Tortoise microvirus 13]	99%	0	53.43%	QCS36834.1
2PE-MCP-3	MZ374770	lizardfe002	SRX11831382	5113	5787	major capsid protein	complete	Microviridae	major capsid protein [Tortoise microvirus 49]	99%	0	53.61%	QCS37072.1

Virus strain name	GenBank No.	LibraryID	SRA Accession No.	Length	Virus Reads	Virus hallmark genes	Completeness of CDS	Classification	Blastx hits on known protein and viruses	Coverage (%)	E-value	Identity (%)	Accession no. of best match
2PE-MCP-4	MZ374771	lizardfe002	SRX11831382	5919	416	major capsid protein	complete	Microviridae	major capsid protein [Microviridae sp.]	96%	2E-16	23.04%	AXH73451.1
2PE-MCP-5	MZ374772	lizardfe002	SRX11831382	3884	3506	major capsid protein	complete	Microviridae	major capsid protein [Chicken microvirus mg8_45]	78%	0	74.25%	SCH20855.1
2PE-MCP-6	MZ374773	lizardfe002	SRX11831382	2854	219	major capsid protein	complete	Microviridae	major capsid protein [Microviridae sp.]	99%	6E-72	30.20%	AXH75515.1
2PE-MCP-7	MZ374774	lizardfe002	SRX11831382	2719	159	major capsid protein	complete	Microviridae	major capsid protein [Microviridae sp.]	97%	4E-71	30.54%	AXH74252.1
2PE-MCP-8	MZ374775	lizardfe002	SRX11831382	2298	53	major capsid protein	partial	Microviridae	major capsid protein [Microviridae sp.]	93%	1E-131	44.30%	SCH20855.1
2PE-MCP-9	MZ374776	lizardfe002	SRX11831382	1734	43	major capsid protein	partial	Microviridae	major capsid protein [Microvirus sp.]	97%	5E-45	30.56%	QCS36051.1
2PE-NS1-1	MZ375120	lizardfe002	SRX11831382	5187	36920	nonstructual protein 1	complete	Parvoviridae	NS1 [Ambidensovirus sp.]	99%	0	60.60%	QGZ11177.1
2PE-NS1-2	MZ375121	lizardfe002	SRX11831382	5309	7865	nonstructual protein 1	complete	Parvoviridae	MAG nonstructural protein 1 [Grus japonensis ambidensovirus]	98%	0	60.64%	QTE03977.1
2PE-NS1-3	MZ375122	lizardfe002	SRX11831382	2379	324	nonstructual protein 1	complete	Parvoviridae	nonstructural protein [Melipona quadrifasciata densovirus]	97%	1E-165	49.22%	QCE20585.1
2PE-NS1-4	MZ375123	lizardfe002	SRX11831382	1425	20	nonstructual protein 1	partial	Parvoviridae	nonstructural protein [Parvoviridae sp.]	88%	2E-156	66.57%	QKE54967.1
2PE-RDRP-1	MZ375136	lizardfe002	SRX11831382	8417	299916	RNA dependent RNA polymerase	complete	Astroviridae	ORF1ab [Guangxi changeable lizard astrovirus]	90%	0	45.29%	AVM87190.1
2PE-RDRP-2	MZ375137	lizardfe002	SRX11831382	8407	104561	RNA dependent RNA polymerase	complete	Astroviridae	ORF1ab [Guangxi changeable lizard astrovirus]	87%	0	44.88%	AVM87190.1
2PE-RDRP-9	MZ375173	lizardfe002	SRX11831382	10380	143523	RNA dependent RNA polymerase	complete	unclassified Riboviria	hypothetical protein 1 [Hubei orthoptera virus 3]	98%	0	46.88%	YP_009336506.1
2PE-RDRP-10	MZ375174	lizardfe002	SRX11831382	5812	1985	RNA dependent RNA polymerase	partial	unclassified Riboviria	nonstructural polyprotein [Dicistroviridae sp.]	98%	0	57.89%	QJI52079.1
2PE-RDRP-11	MZ375175	lizardfe002	SRX11831382	5493	335	RNA dependent RNA polymerase	complete	unclassified Riboviria	RNA-dependent RNA polymerase [Gervais tombus-like virus]	98%	0	56.94%	QIJ70125.1
2PE-RDRP-12	MZ375176	lizardfe002	SRX11831382	5456	426	RNA dependent RNA polymerase	complete	unclassified Riboviria	RNA-dependent RNA polymerase [Gervais tombus-like virus]	97%	0	57.44%	QIJ70125.1
2PE-RDRP-13	MZ375177	lizardfe002	SRX11831382	3333	683	RNA dependent RNA polymerase	complete	unclassified Riboviria	RNA-dependent RNA polymerase [Gervais tombus-like virus]	99%	0	54.89%	QIJ70125.1
2PE-RDRP-14	MZ375178	lizardfe002	SRX11831382	2940	238	RNA dependent RNA polymerase	partial	unclassified Riboviria	RNA-dependent RNA polymerase [Soybean thrips sobemo-like virus 5]	92%	0	77.75%	QPZ88401.1
2PE-RDRP-15	MZ375179	lizardfe002	SRX11831382	2873	299	RNA dependent RNA polymerase	complete	unclassified Riboviria	hypothetical protein 2 [Hubei sobemo-like virus 42]	98%	0	64.10%	YP_009330107.1
2PE-RDRP-16	MZ375180	lizardfe002	SRX11831382	1973	5640	RNA dependent RNA polymerase	partial	unclassified Riboviria	hypothetical protein 2 [Hubei diptera virus 14]	99%	2E-108	60.23%	YP_009337875.1
2PE-RDRP-18	MZ375181	lizardfe002	SRX11831382	1774	49	RNA dependent RNA polymerase	partial	unclassified Riboviria	RdRp [Hubei partiti-like virus 27]	99%	0	66.60%	APG78241.1
2PE-RDRP-19	MZ375182	lizardfe002	SRX11831382	1721	102	RNA dependent RNA polymerase	partial	unclassified Riboviria	hypothetical protein 3 [Hubei tombus-like virus 14]	94%	2E-165	51.19%	YP_009336965.1
2PE-RDRP-20	MZ375183	lizardfe002	SRX11831382	1571	152294	RNA dependent RNA polymerase	complete	unclassified Riboviria	RdRp [Atrato Partiti-like virus 4]	96%	0	59.91%	QHA33703.1
2PE-RDRP-21	MZ375184	lizardfe002	SRX11831382	1562	461	RNA dependent RNA polymerase	partial	unclassified Riboviria	RNA-dependent RNA polymerase [Soybean thrips sobemo-like virus 5]	91%	0	71.70%	QPZ88401.1
2PE-RDRP-22	MZ375185	lizardfe002	SRX11831382	1530	269	RNA dependent RNA polymerase	partial	unclassified Riboviria	RNA-dependent RNA polymerase [Soybean thrips sobemo-like virus 5]	99%	0	71.87%	QPZ88401.1
2PE-RDRP-23	MZ375186	lizardfe002	SRX11831382	1463	258	RNA dependent RNA polymerase	complete	unclassified Riboviria	RdRp [Hubei partiti-like virus 48]	90%	1E-120	47.28%	APG78218.1
2PE-RDRP-25	MZ375187	lizardfe002	SRX11831382	1004	24	RNA dependent RNA polymerase	partial	unclassified Riboviria	hypothetical protein 2 [Jingmen tombus-like virus 2]	91%	5E-48	42.67%	YP_009344965.1
2PE-RDRP-27	MZ375220	lizardfe002	SRX11831382	1075	34	RNA dependent RNA polymerase	partial	Tombusviridae	RNA dependent RNA polymerase [Yam spherical virus]	99%	1E-125	70.47%	YP_008828157.1
2PE-REP-2	MZ375114	lizardfe002	SRX11831382	398	35	replication-associated protein	partial	Circoviridae	replication-associated protein [CRESS virus sp.]	96%	1E-14	45.00%	QSL97429.1
2PE-REP-4	MZ375115	lizardfe002	SRX11831382	3930	259	replication-associated protein	complete	unclassified CRESS DNA virus	REP [Rodent stool-associated circular genome virus]	99%	7E-151	67.64%	AEM05798.1
2PE-TERL-1	MZ374838	lizardfe002	SRX11831382	2572	75	terminase large subunit	complete	uncultured Caudovirales phage	terminase large subunit [IAS virus]	97%	0	59.97%	YP_009981724.1
2PE-TERL-2	MZ374839	lizardfe002	SRX11831382	2491	108	terminase large subunit	complete	uncultured Caudovirales phage	phage terminase large subunit family protein [Anaeromassilibacillus senegalensis]	99%	0	91.12%	WP_050697989.1
2PE-TERL-3	MZ374840	lizardfe002	SRX11831382	2390	166	terminase large subunit	complete	uncultured Caudovirales phage	phage terminase large subunit family protein [Klebsiella pneumoniae]	99%	0	83.33%	WP_200795005.1
2PE-TERL-4	MZ374841	lizardfe002	SRX11831382	2361	101	terminase large subunit	complete	uncultured Caudovirales phage	phage terminase large subunit family protein [Eisenbergiella tayi]	99%	0	90.23%	WP_069429757.1
2PE-TERL-5	MZ374842	lizardfe002	SRX11831382	2313	336	terminase large subunit	complete	uncultured Caudovirales phage	terminase [Lacrimispora amygdalina]	99%	0	86.11%	WP_144363778.1
2PE-TERL-6	MZ374843	lizardfe002	SRX11831382	2301	65	terminase large subunit	complete	uncultured Caudovirales phage	phage terminase large subunit family protein [Enterocloster bolteae]	94%	0	96.92%	WP_160277521.1
2PE-TERL-7	MZ374844	lizardfe002	SRX11831382	2271	15	terminase large subunit	complete	uncultured Caudovirales phage	phage terminase large subunit family protein [[Clostridium] symbiosum]	99%	0	89.23%	WP_202185501.1
2PE-TERL-8	MZ374845	lizardfe002	SRX11831382	2261	289	terminase large subunit	complete	uncultured Caudovirales phage	terminase large subunit [Blautia producta]	99%	0	93.93%	WP_171287229.1
2PE-TERL-9	MZ374846	lizardfe002	SRX11831382	2253	20	terminase large subunit	complete	uncultured Caudovirales phage	terminase [Oscillibacter sp.]	99%	0	75.09%	MBD5169250.1
2PE-TERL-10	MZ374847	lizardfe002	SRX11831382	2252	92	terminase large subunit	complete	uncultured Caudovirales phage	terminase large subunit [Parabacteroides sp. AF19-14]	99%	0	92.21%	WP_122379962.1
2PE-TERL-11	MZ374848	lizardfe002	SRX11831382	2251	516	terminase large subunit	complete	uncultured Caudovirales phage	phage terminase large subunit [Bacteroides thetaiotaomicron]	99%	0	93.21%	WP_054960548.1
2PE-TERL-12	MZ374849	lizardfe002	SRX11831382	2240	133	terminase large subunit	complete	uncultured Caudovirales phage	MULTISPECIES: terminase large subunit [Bacteroides]	99%	0	93.52%	WP_008999297.1
2PE-TERL-13	MZ374850	lizardfe002	SRX11831382	2222	20	terminase large subunit	complete	uncultured Caudovirales phage	terminase large subunit [Bacteroides uniformis]	99%	0	92.06%	WP_117959321.1
2PE-TERL-14	MZ374851	lizardfe002	SRX11831382	2205	38	terminase large subunit	complete	uncultured Caudovirales phage	terminase large subunit [[Clostridium] symbiosum]	99%	0	92.54%	WP_195321274.1
2PE-TERL-15	MZ374852	lizardfe002	SRX11831382	2193	80	terminase large subunit	complete	uncultured Caudovirales phage	terminase large subunit [Clostridia bacterium]	99%	0	55.07%	MBQ2775158.1
2PE-TERL-16	MZ374853	lizardfe002	SRX11831382	2176	120	terminase large subunit	complete	uncultured Caudovirales phage	terminase [Eubacterium maltosivorans]	98%	0	68.57%	WP_074618602.1
2PE-TERL-17	MZ374854	lizardfe002	SRX11831382	2157	2101	terminase large subunit	complete	uncultured Caudovirales phage	terminase large subunit [Enterobacter hormaechei]	99%	0	95.63%	WP_058688077.1
2PE-TERL-18	MZ374855	lizardfe002	SRX11831382	2134	13	terminase large subunit	complete	uncultured Caudovirales phage	hypothetical protein [Clostridia bacterium]	94%	0	55.98%	MBR6688796.1

Virus strain name	GenBank No.	LibraryID	SRA Accession No.	Length	Virus Reads	Virus hallmark genes	Completeness of CDS	Classification	Blastx hits on known protein and viruses	Coverage (%)	E-value	Identity (%)	Accession no. of best match
2PE-TERL-19	MZ374856	lizardfe002	SRX11831382	2105	86	terminase large subunit	complete	uncultured Caudovirales phage	terminase large subunit [Atopobiaceae bacterium]	99%	0	67.36%	MBR3312640.1
2PE-TERL-20	MZ374857	lizardfe002	SRX11831382	2099	1240	terminase large subunit	complete	uncultured Caudovirales phage	terminase [Anaerotruncus colihominis]	99%	0	95.18%	WP_087300728.1
2PE-TERL-21	MZ374858	lizardfe002	SRX11831382	2079	188	terminase large subunit	complete	uncultured Caudovirales phage	phage terminase large subunit family protein [Clostridiaceae bacterium]	99%	0	79.06%	NBH77717.1
2PE-TERL-22	MZ374859	lizardfe002	SRX11831382	2069	138	terminase large subunit	complete	uncultured Caudovirales phage	terminase family protein [Bacteroidales bacterium]	99%	0	69.94%	MBR3286951.1
2PE-TERL-23	MZ374860	lizardfe002	SRX11831382	2065	94	terminase large subunit	complete	uncultured Caudovirales phage	phage terminase large subunit [Bacteroides clarus]	99%	0	84.19%	WP_196053082.1
2PE-TERL-24	MZ374861	lizardfe002	SRX11831382	2014	204	terminase large subunit	complete	uncultured Caudovirales phage	PBSX family phage terminase large subunit [Clostridium sp. HMb25]	99%	0	83.77%	PKB53660.1
2PE-TERL-25	MZ374862	lizardfe002	SRX11831382	1989	13	terminase large subunit	partial	uncultured Caudovirales phage	terminase large subunit [Lachnospiraceae bacterium]	98%	0	89.97%	MBD5550227.1
2PE-TERL-26	MZ374863	lizardfe002	SRX11831382	1985	529	terminase large subunit	complete	uncultured Caudovirales phage	PBSX family phage terminase large subunit [Enterocloster citroniae]	99%	0	96.51%	WP_117451920.1
2PE-TERL-27	MZ374864	lizardfe002	SRX11831382	1961	121	terminase large subunit	complete	uncultured Caudovirales phage	Terminase-like family protein [Clostridiales bacterium]	99%	0	82.08%	PWL46840.1
2PE-TERL-28	MZ374865	lizardfe002	SRX11831382	1948	89	terminase large subunit	complete	uncultured Caudovirales phage	PBSX family phage terminase large subunit [Clostridium sp. FS41]	99%	0	92.13%	WP_045093877.1
2PE-TERL-29	MZ374866	lizardfe002	SRX11831382	1947	25	terminase large subunit	complete	uncultured Caudovirales phage	phage terminase large subunit [Oscillibacter sp.]	97%	0	71.34%	MBQ5928030.1
2PE-TERL-30	MZ374867	lizardfe002	SRX11831382	1923	603	terminase large subunit	partial	uncultured Caudovirales phage	phage terminase large subunit family protein [bacterium 1XD42-76]	99%	0	86.99%	NBJ83417.1
2PE-TERL-31	MZ374868	lizardfe002	SRX11831382	1906	21	terminase large subunit	complete	uncultured Caudovirales phage	MULTISPECIES: PBSX family phage terminase large subunit [Eubacteriales]	98%	0	87.27%	WP_118756516.1
2PE-TERL-32	MZ374869	lizardfe002	SRX11831382	1886	95	terminase large subunit	complete	uncultured Caudovirales phage	PBSX family phage terminase large subunit [Clostridia bacterium]	98%	0	72.06%	MBO5743457.1
2PE-TERL-33	MZ374870	lizardfe002	SRX11831382	1868	231	terminase large subunit	complete	uncultured Caudovirales phage	PBSX family phage terminase large subunit [Ruminococcus sp. D55t1_190419_H1]	99%	0	81.55%	WP_195477907.1
2PE-TERL-34	MZ374871	lizardfe002	SRX11831382	1860	52	terminase large subunit	complete	uncultured Caudovirales phage	PBSX family phage terminase large subunit [Anaerostipes sp. NSJ-7]	99%	0	96.84%	WP_186992475.1
2PE-TERL-35	MZ374872	lizardfe002	SRX11831382	1850	340	terminase large subunit	complete	uncultured Caudovirales phage	PBSX family phage terminase large subunit [Erysipelotrichaceae bacterium 66202529]	99%	0	99.78%	QSI24531.1
2PE-TERL-36	MZ374873	lizardfe002	SRX11831382	1838	10	terminase large subunit	complete	uncultured Caudovirales phage	MULTISPECIES: PBSX family phage terminase large subunit [Eubacteriales]	99%	0	86.07%	WP_015527567.1
2PE-TERL-37	MZ374874	lizardfe002	SRX11831382	1817	272	terminase large subunit	complete	uncultured Caudovirales phage	PBSX family phage terminase large subunit [Enterocloster lavalensis]	99%	0	92.03%	WP_107436570.1
2PE-TERL-38	MZ374875	lizardfe002	SRX11831382	1814	65	terminase large subunit	complete	uncultured Caudovirales phage	phage terminase large subunit [Anaerotruncus rubiinfantis]	99%	0	78.51%	WP_066460208.1
2PE-TERL-39	MZ374876	lizardfe002	SRX11831382	1802	46	terminase large subunit	complete	uncultured Caudovirales phage	phage terminase large subunit [Clostridia bacterium]	94%	0	62.03%	MBQ8689465.1
2PE-TERL-40	MZ374877	lizardfe002	SRX11831382	1769	12	terminase large subunit	partial	uncultured Caudovirales phage	MULTISPECIES: terminase large subunit [Eubacteriales]	99%	0	96.27%	WP_002591266.1
2PE-TERL-41	MZ374878	lizardfe002	SRX11831382	1755	46	terminase large subunit	complete	uncultured Caudovirales phage	hypothetical protein [Bacteroides intestinalis]	99%	0	94.12%	WP_061433606.1
2PE-TERL-42	MZ374879	lizardfe002	SRX11831382	1710	54	terminase large subunit	partial	uncultured Caudovirales phage	hypothetical protein [Oscillospiraceae bacterium BX18]	98%	5E-161	52.01%	MBC8582039.1
2PE-TERL-43	MZ374880	lizardfe002	SRX11831382	1704	180	terminase large subunit	partial	uncultured Caudovirales phage	terminase [Lachnospiraceae bacterium]	99%	0	90.87%	NBH27241.1
2PE-TERL-44	MZ374881	lizardfe002	SRX11831382	1703	62	terminase large subunit	partial	uncultured Caudovirales phage	Phage terminase large subunit (GpA) [Acetatifactor muris]	99%	0	91.47%	SOY30065.1
2PE-TERL-45	MZ374882	lizardfe002	SRX11831382	1695	107	terminase large subunit	partial	uncultured Caudovirales phage	phage terminase large subunit family protein [bacterium 1XD42-76]	99%	0	67.00%	NBJ83417.1
2PE-TERL-46	MZ374883	lizardfe002	SRX11831382	1688	50	terminase large subunit	partial	uncultured Caudovirales phage	terminase [Erysipelatoclostridium ramosum]	99%	0	72.19%	WP_117580262.1
2PE-TERL-47	MZ374884	lizardfe002	SRX11831382	1613	64	terminase large subunit	complete	uncultured Caudovirales phage	PBSX family phage terminase large subunit [Parabacteroides distasonis]	99%	0	90.76%	WP_011966215.1
2PE-TERL-48	MZ374885	lizardfe002	SRX11831382	1564	16	terminase large subunit	partial	uncultured Caudovirales phage	phage terminase large subunit family protein [Pseudoflavonifractor sp. 524-17]	99%	0	78.59%	NCE63876.1
2PE-TERL-49	MZ374886	lizardfe002	SRX11831382	1560	91	terminase large subunit	partial	uncultured Caudovirales phage	phage terminase, large subunit, PBSX family [Lacrimispora sphenoides]	99%	0	87.17%	SET79753.1
2PE-TERL-50	MZ374887	lizardfe002	SRX11831382	1551	25	terminase large subunit	partial	uncultured Caudovirales phage	phage terminase large subunit [Odoribacter splanchnicus]	96%	0	81.72%	WP_087381242.1
2PE-TERL-51	MZ374888	lizardfe002	SRX11831382	1532	66	terminase large subunit	partial	uncultured Caudovirales phage	terminase [Bacteroides uniformis]	99%	0	74.01%	WP_202192604.1
2PE-TERL-52	MZ374889	lizardfe002	SRX11831382	1525	35	terminase large subunit	partial	uncultured Caudovirales phage	Phage terminase-like protein, large subunit [uncultured Blautia sp.]	99%	0	76.26%	VEJ95862.1
2PE-TERL-53	MZ374890	lizardfe002	SRX11831382	1510	46	terminase large subunit	partial	uncultured Caudovirales phage	MULTISPECIES: phage terminase large subunit [Anaerotruncus]	99%	0	84.63%	WP_160202290.1
2PE-TERL-54	MZ374891	lizardfe002	SRX11831382	1503	67	terminase large subunit	partial	uncultured Caudovirales phage	PBSX family phage terminase large subunit [Lachnospiraceae bacterium]	99%	0	70.75%	MBQ8413503.1
2PE-TERL-55	MZ374892	lizardfe002	SRX11831382	1487	22	terminase large subunit	partial	uncultured Caudovirales phage	MULTISPECIES: phage terminase large subunit family protein [Eubacteriales]	95%	0	78.69%	WP_008396678.1
2PE-TERL-56	MZ374893	lizardfe002	SRX11831382	1439	16	terminase large subunit	partial	uncultured Caudovirales phage	terminase large subunit [Clostridiaceae bacterium]	99%	0	72.82%	NLU52370.1
2PE-TERL-57	MZ374894	lizardfe002	SRX11831382	1427	47	terminase large subunit	partial	uncultured Caudovirales phage	phage terminase large subunit [Bacteroides ovatus]	99%	0	92.94%	KAA4071616.1
2PE-TERL-58	MZ374895	lizardfe002	SRX11831382	1404	36	terminase large subunit	partial	uncultured Caudovirales phage	terminase family protein [Prevotella sp.]	94%	2E-62	31.89%	MBP5257152.1
2PE-TERL-59	MZ374896	lizardfe002	SRX11831382	1402	47	terminase large subunit	partial	uncultured Caudovirales phage	MULTISPECIES: terminase large subunit [Eggerthella]	99%	0	79.90%	WP_114526811.1
2PE-TERL-60	MZ374897	lizardfe002	SRX11831382	1388	34	terminase large subunit	partial	uncultured Caudovirales phage	terminase large subunit [Bacteroides sp. KFT8]	99%	0	89.01%	WP_099146696.1
2PE-TERL-61	MZ374898	lizardfe002	SRX11831382	1376	73	terminase large subunit	partial	uncultured Caudovirales phage	terminase large subunit [Bacteroides thetaiotaomicron]	98%	0	72.25%	KAB4839554.1
2PE-TERL-62	MZ374899	lizardfe002	SRX11831382	1371	23	terminase large subunit	partial	uncultured Caudovirales phage	PBSX family phage terminase large subunit [Oscillibacter ruminantium]	99%	0	76.28%	WP_051131713.1
2PE-TERL-63	MZ374900	lizardfe002	SRX11831382	1341	15	terminase large subunit	partial	uncultured Caudovirales phage	terminase [Oscillibacter sp.]	98%	0	77.42%	MBD5169250.1
2PE-TERL-64	MZ374901	lizardfe002	SRX11831382	1339	34	terminase large subunit	partial	uncultured Caudovirales phage	MULTISPECIES: terminase [unclassified Anaerotruncus]	100%	0	99.41%	WP_120484317.1
2PE-TERL-65	MZ374902	lizardfe002	SRX11831382	1334	47	terminase large subunit	partial	uncultured Caudovirales phage	hypothetical protein EKK62_00085 [Acidimicrobiia bacterium]	97%	9E-171	65.72%	RTL09810.1
2PE-TERL-66	MZ374903	lizardfe002	SRX11831382	1329	36	terminase large subunit	partial	uncultured Caudovirales phage	PBSX family phage terminase large subunit [Dorea longicatena]	99%	0	83.71%	WP_161158730.1

Virus strain name	GenBank No.	LibraryID	SRA Accession No.	Length	Virus Reads	Virus hallmark genes	Completeness of CDS	Classification	Blastx hits on known protein and viruses	Coverage (%)	E-value	Identity (%)	Accession no. of best match
2PE-TERL-67	MZ374904	lizardfe002	SRX11831382	1305	25	terminase large subunit	partial	uncultured Caudovirales phage	PBSX family phage terminase large subunit [Clostridium sp. FS41]	98%	0	72.88%	WP_045093877.1
2PE-TERL-68	MZ374905	lizardfe002	SRX11831382	1287	25	terminase large subunit	partial	uncultured Caudovirales phage	hypothetical protein [Bacteroides thetaiotaomicron]	98%	0	84.20%	WP_061472845.1
2PE-TERL-69	MZ374906	lizardfe002	SRX11831382	1244	173	terminase large subunit	partial	uncultured Caudovirales phage	terminase [Lachnospiraceae bacterium]	100%	0	91.74%	NBH27241.1
2PE-TERL-70	MZ374907	lizardfe002	SRX11831382	1243	64	terminase large subunit	partial	uncultured Caudovirales phage	PBSX family phage terminase large subunit [Anaerosacchriphilus polymeriproducens]	93%	0	70.14%	WP_115483093.1
2PE-TERL-71	MZ374908	lizardfe002	SRX11831382	1230	41	terminase large subunit	partial	uncultured Caudovirales phage	putative phage terminase, large subunit [Clostridium sp. HGF2]	100%	0	100.00%	EFR39717.1
2PE-TERL-72	MZ374909	lizardfe002	SRX11831382	1178	24	terminase large subunit	partial	uncultured Caudovirales phage	terminase large subunit [Enterocloster citroniae]	99%	0	97.96%	MBE7725875.1
2PE-TERL-73	MZ374910	lizardfe002	SRX11831382	1174	50	terminase large subunit	partial	uncultured Caudovirales phage	MULTISPECIES: phage terminase large subunit [unclassified Pseudoflavonifractor]	99%	0	84.85%	WP_155152106.1
2PE-TERL-74	MZ374911	lizardfe002	SRX11831382	1173	30	terminase large subunit	partial	uncultured Caudovirales phage	terminase [Erysipelotrichaceae bacterium 66202529]	99%	2E-161	72.08%	QSI27780.1
2PE-TERL-75	MZ374912	lizardfe002	SRX11831382	1171	29	terminase large subunit	partial	uncultured Caudovirales phage	phage terminase large subunit [Butyricicoccus pullicaecorum]	99%	0	73.59%	WP_087370162.1
2PE-TERL-76	MZ374913	lizardfe002	SRX11831382	1154	36	terminase large subunit	partial	uncultured Caudovirales phage	PBSX family phage terminase large subunit [Intestinibacillus massiliensis]	99%	0	91.26%	WP_143436024.1
2PE-TERL-77	MZ374914	lizardfe002	SRX11831382	1150	19	terminase large subunit	partial	uncultured Caudovirales phage	terminase [Desulfosporosinus sp. BRH_c37]	99%	0	77.28%	KUO72701.1
2PE-TERL-78	MZ374915	lizardfe002	SRX11831382	1136	93	terminase large subunit	partial	uncultured Caudovirales phage	MULTISPECIES: phage terminase large subunit [Bacteroidales]	99%	4E-177	89.39%	WP_155226838.1
2PE-TERL-79	MZ374916	lizardfe002	SRX11831382	1135	22	terminase large subunit	partial	uncultured Caudovirales phage	phage terminase large subunit [Odoribacter splanchnicus]	99%	3E-148	75.00%	WP_087381242.1
2PE-TERL-80	MZ374917	lizardfe002	SRX11831382	1133	38	terminase large subunit	partial	uncultured Caudovirales phage	TPA: terminase large subunit [Lachnospiraceae bacterium]	98%	0	91.04%	HAB61739.1
2PE-TERL-81	MZ374918	lizardfe002	SRX11831382	1126	39	terminase large subunit	partial	uncultured Caudovirales phage	PBSX family phage terminase large subunit [Beduini massiliensis]	99%	4E-142	72.14%	WP_041137858.1
2PE-TERL-82	MZ374919	lizardfe002	SRX11831382	1123	11	terminase large subunit	partial	uncultured Caudovirales phage	terminase large subunit [Butyricicoccus pullicaecorum]	99%	0	77.48%	WP_087372136.1
2PE-TERL-83	MZ374920	lizardfe002	SRX11831382	1118	12	terminase large subunit	partial	uncultured Caudovirales phage	hypothetical protein [Slackia faecicanis]	98%	8E-160	67.55%	WP_123197916.1
2PE-TERL-84	MZ374921	lizardfe002	SRX11831382	1094	43	terminase large subunit	partial	uncultured Caudovirales phage	phage terminase large subunit [Bacteroides ovatus]	97%	0	96.33%	WP_175353440.1
2PE-TERL-85	MZ374922	lizardfe002	SRX11831382	1062	30	terminase large subunit	partial	uncultured Caudovirales phage	phage terminase large subunit [Bacteroides salyersiae]	99%	0	92.07%	WP_149986531.1
2PE-TERL-86	MZ374923	lizardfe002	SRX11831382	1061	41	terminase large subunit	partial	uncultured Caudovirales phage	phage terminase large subunit family protein [Clostridiaceae bacterium]	99%	6E-175	85.25%	NBH77956.1
2PE-TERL-87	MZ374924	lizardfe002	SRX11831382	1043	29	terminase large subunit	partial	uncultured Caudovirales phage	PBSX family phage terminase large subunit [Anaeromassilibacillus senegalensis]	99%	0	96.06%	WP_082236500.1
2PE-TERL-88	MZ374925	lizardfe002	SRX11831382	1031	13	terminase large subunit	partial	uncultured Caudovirales phage	TPA: terminase [Escherichia coli]	99%	0	98.50%	HAJ5146084.1
2PE-TERL-89	MZ374926	lizardfe002	SRX11831382	1022	33	terminase large subunit	partial	uncultured Caudovirales phage	PBSX family phage terminase large subunit [Enterocloster bolteae]	99%	0	97.33%	WP_118024319.1
2PE-TERL-90	MZ374927	lizardfe002	SRX11831382	1018	29	terminase large subunit	partial	uncultured Caudovirales phage	TPA: terminase large subunit [Firmicutes bacterium]	99%	0	78.47%	HBE76350.1
2PE-TERL-91	MZ374928	lizardfe002	SRX11831382	1009	55	terminase large subunit	partial	uncultured Caudovirales phage	phage terminase, large subunit, PBSX family [Bacteroides uniformis]	99%	1E-160	94.32%	KXT33443.1
2PE-TERL-92	MZ374929	lizardfe002	SRX11831382	1007	37	terminase large subunit	partial	uncultured Caudovirales phage	terminase large subunit [Bacteroides fragilis]	98%	1E-152	95.53%	WP_122128079.1
2PE-TERL-93	MZ374930	lizardfe002	SRX11831382	962	27	terminase large subunit	partial	uncultured Caudovirales phage	terminase [Clostridiales bacterium 36_14]	99%	4E-133	77.49%	OKZ74881.1
2PE-TERL-94	MZ374931	lizardfe002	SRX11831382	929	33	terminase large subunit	partial	uncultured Caudovirales phage	terminase [Bacteroides uniformis]	98%	4E-119	73.82%	WP_202192604.1
2PE-TERL-95	MZ374932	lizardfe002	SRX11831382	897	28	terminase large subunit	partial	uncultured Caudovirales phage	terminase [Enterocloster clostridioformis]	99%	1E-108	82.63%	WP_057571348.1
2PE-TERL-96	MZ374933	lizardfe002	SRX11831382	890	49	terminase large subunit	partial	uncultured Caudovirales phage	TPA: terminase [Lachnospiraceae bacterium]	99%	9E-89	73.56%	HCX91512.1
2PE-TERL-97	MZ374934	lizardfe002	SRX11831382	885	15	terminase large subunit	partial	uncultured Caudovirales phage	phage terminase large subunit [Anaerotruncus sp. AF02-27]	100%	6E-102	74.62%	WP_118480894.1
2PE-TERL-98	MZ374935	lizardfe002	SRX11831382	846	23	terminase large subunit	partial	uncultured Caudovirales phage	hypothetical protein [Olsenella sp. HMSC062G07]	100%	5E-90	69.11%	WP_083307154.1
2PE-TERL-99	MZ374936	lizardfe002	SRX11831382	838	34	terminase large subunit	partial	uncultured Caudovirales phage	phage terminase large subunit family protein [Clostridiaceae bacterium]	98%	2E-97	75.13%	NBH77956.1
2PE-TERL-100	MZ374937	lizardfe002	SRX11831382	817	22	terminase large subunit	partial	uncultured Caudovirales phage	PBSX family phage terminase large subunit [Beduini massiliensis]	97%	1E-94	72.83%	WP_041137858.1
2PE-TERL-101	MZ374938	lizardfe002	SRX11831382	793	35	terminase large subunit	partial	uncultured Caudovirales phage	phage terminase large subunit [Eisenbergiella sp. OF01-20]	99%	2E-131	93.81%	WP_118680996.1
2PE-TERL-102	MZ374939	lizardfe002	SRX11831382	738	15	terminase large subunit	partial	uncultured Caudovirales phage	terminase [Eubacterium callanderi]	99%	3E-55	55.35%	WP_180494070.1
2PE-TERL-103	MZ374940	lizardfe002	SRX11831382	666	34	terminase large subunit	partial	uncultured Caudovirales phage	PBSX family phage terminase large subunit [Massilibacillus massiliensis]	96%	1E-53	70.40%	WP_199883998.1
3PE-MCP-1	MZ374777	lizardfe003	SRX11831397	6224	2419	major capsid protein	complete	Microviridae	major capsid protein [Microvirus sp.]	92%	3E-141	36.92%	WP_195578815.1
3PE-MCP-2	MZ374778	lizardfe003	SRX11831397	6026	2039	major capsid protein	complete	Microviridae	major capsid protein [Microviridae sp.]	40%	4E-16	25.96%	AXH77152.1
3PE-MCP-3	MZ374779	lizardfe003	SRX11831397	5496	245	major capsid protein	complete	Microviridae	major capsid protein [Microviridae sp.]	97%	5E-96	36.25%	WP_195525717.1
3PE-MCP-4	MZ374780	lizardfe003	SRX11831397	4744	234	major capsid protein	complete	Microviridae	major capsid protein [Microviridae sp.]	99%	2E-167	52.22%	AXF52770.1
3PE-MCP-5	MZ374781	lizardfe003	SRX11831397	2823	57	major capsid protein	complete	Microviridae	major capsid protein [Capybara microvirus Cap1_SP_109]	99%	2E-103	34.39%	QCS36061.1
3PE-MCP-6	MZ374782	lizardfe003	SRX11831397	2705	50	major capsid protein	complete	Microviridae	major capsid protein [Microviridae sp.]	99%	3E-155	45.37%	AXH73645.1
3PE-MCP-7	MZ374783	lizardfe003	SRX11831397	2450	125	major capsid protein	complete	Microviridae	putative VP1 [Microviridae sp.]	98%	1E-138	46.26%	AYQ58205.1
3PE-MCP-8	MZ374784	lizardfe003	SRX11831397	2192	1874	major capsid protein	partial	Microviridae	major capsid protein [Chicken microvirus mg8_45]	99%	0	55.26%	WP_195578815.1
3PE-MCP-9	MZ374785	lizardfe003	SRX11831397	2159	53	major capsid protein	complete	Microviridae	major capsid protein [Microviridae sp.]	99%	1E-102	38.15%	AXF52319.1
3PE-MCP-10	MZ374786	lizardfe003	SRX11831397	1929	37	major capsid protein	partial	Microviridae	major capsid protein [Microviridae sp.]	98%	5E-41	30.70%	MBO5632558.1
3PE-MCP-11	MZ374787	lizardfe003	SRX11831397	1684	27	major capsid protein	partial	Microviridae	major capsid protein [Microviridae sp.]	99%	7E-102	40.11%	AXH74252.1

Virus strain name	GenBank No.	LibraryID	SRA Accession No.	Length	Virus Reads	Virus hallmark genes	Completeness of CDS	Classification	Blastx hits on known protein and viruses	Coverage (%)	E-value	Identity (%)	Accession no. of best match
3PE-MCP-12	MZ374788	lizardfe003	SRX11831397	1661	40	major capsid protein	partial	Microviridae	VP1 [Gokushovirus WZ-2015a]	99%	0	79.25%	ALS03599.1
3PE-MCP-13	MZ374789	lizardfe003	SRX11831397	1562	27	major capsid protein	partial	Microviridae	major capsid protein [Capybara microvirus Cap1_SP_83]	99%	2E-71	39.80%	QCS35998.1
3PE-MCP-14	MZ374790	lizardfe003	SRX11831397	1545	22	major capsid protein	partial	Microviridae	major capsid protein [Capybara microvirus Cap1_SP_107]	99%	2E-48	30.04%	QCS36051.1
3PE-MCP-16	MZ374791	lizardfe003	SRX11831397	1421	20	major capsid protein	partial	Microviridae	major capsid protein [Microviridae sp.]	99%	4E-77	36.40%	AXH74252.1
3PE-MCP-17	MZ374792	lizardfe003	SRX11831397	1410	18	major capsid protein	partial	Microviridae	major capsid protein [Capybara microvirus Cap1_SP_83]	99%	5E-89	41.85%	QCS35998.1
3PE-MCP-18	MZ374793	lizardfe003	SRX11831397	1126	15	major capsid protein	partial	Microviridae	major capsid protein [Microvirus sp.]	99%	2E-57	40.23%	QRV61846.1
3PE-MCP-19	MZ374794	lizardfe003	SRX11831397	1124	42283	major capsid protein	partial	Microviridae	major capsid protein [Chicken microvirus mg8_45]	99%	6E-167	75.78%	RHK08760.1
3PE-NS1-1	MZ375124	lizardfe003	SRX11831397	5325	788	nonstructual protein 1	complete	Parvoviridae	NS1 [Murine bocavirus]	87%	0	53.46%	AWB14590.1
3PE-NS1-2	MZ375125	lizardfe003	SRX11831397	4334	5942	nonstructual protein 1	complete	Parvoviridae	nonstructural protein [Melipona quadrifasciata densovirus]	95%	8E-165	49.22%	QCE20585.1
3PE-NS1-3	MZ375126	lizardfe003	SRX11831397	3477	119	nonstructual protein 1	complete	Parvoviridae	NS1 [Lupine bocavirus]	98%	0	47.97%	YP_009551693.1
3PE-NS1-4	MZ375127	lizardfe003	SRX11831397	2644	211	nonstructual protein 1	partial	Parvoviridae	NS1 [Lupine bocavirus]	97%	0	53.87%	YP_009551693.1
3PE-NS1-5	MZ375128	lizardfe003	SRX11831397	1758	1681	nonstructual protein 1	partial	Parvoviridae	NS1 [Ambidensovirus sp.]	99%	0	58.52%	QGZ11177.1
3PE-RDRP-1	MZ375138	lizardfe003	SRX11831397	7915	80177	RNA dependent RNA polymerase	complete	Astroviridae	ORF1ab [Guangxi changeable lizard astrovirus]	81%	0	65.30%	AVM87190.1
3PE-RDRP-2	MZ375139	lizardfe003	SRX11831397	7312	110923	RNA dependent RNA polymerase	complete	Astroviridae	ORF1ab [Hainan oriental leaf-toed gecko astrovirus 1]	99%	0	74.67%	AVM87517.1
3PE-RDRP-3	MZ375151	lizardfe003	SRX11831397	947	14	RNA dependent RNA polymerase	partial	Caliciviridae	polyprotein [Hare calicivirus Australia-2]	99%	7E-64	44.04%	QCC62368.1
3PE-RDRP-4	MZ375152	lizardfe003	SRX11831397	891	11	RNA dependent RNA polymerase	partial	Caliciviridae	RNA-dependent RNA polymerase [European brown hare syndrome virus]	96%	2E-85	52.53%	NP_786902.1
3PE-RDRP-5	MZ375221	lizardfe003	SRX11831397	541	823	RNA dependent RNA polymerase	partial	Iflaviridae	RDRP [Slow bee paralysis virus]	99%	8E-81	70.56%	YP_009305421.1
3PE-RDRP-6	MZ375222	lizardfe003	SRX11831397	1439	217	RNA dependent RNA polymerase	partial	Partitiviridae	RdRp [Atrato Partiti-like virus 2]	100%	0	70.76%	QHA33902.1
3PE-RDRP-7	MZ375223	lizardfe003	SRX11831397	924	32	RNA dependent RNA polymerase	partial	Picobirnaviridae	RNA-dependent RNA polymerase [Picobirnavirus sp.]	93%	3E-41	42.27%	AOW41972.1
3PE-RDRP-12	MZ375224	lizardfe003	SRX11831397	11503	2161	RNA dependent RNA polymerase	complete	Polycipiviridae	RNA-dependent RNA polymerase [Lasius neglectus virus 1]	99%	0	50.48%	YP_009407905.1
3PE-RDRP-13	MZ375188	lizardfe003	SRX11831397	2663	28656	RNA dependent RNA polymerase	complete	unclassified Riboviria	hypothetical protein 2 [Hubei diptera virus 14]	98%	3E-157	58.62%	YP_009337875.1
3PE-RDRP-14	MZ375189	lizardfe003	SRX11831397	2517	156	RNA dependent RNA polymerase	partial	unclassified Riboviria	RdRp [Hubei permutotetra-like virus 6]	59%	2E-81	39.13%	YP_009337318.1
3PE-RDRP-15	MZ375190	lizardfe003	SRX11831397	2034	2484	RNA dependent RNA polymerase	complete	unclassified Riboviria	RNA-dependent RNA polymerase [Soybean thrips sobemo-like virus 5]	99%	0	73.18%	QPZ88401.1
3PE-RDRP-16	MZ375191	lizardfe003	SRX11831397	1923	67	RNA dependent RNA polymerase	partial	unclassified Riboviria	hypothetical protein [Hubei coleoptera virus 2]	98%	0	50.65%	YP_009337178.1
3PE-RDRP-17	MZ375192	lizardfe003	SRX11831397	1827	104	RNA dependent RNA polymerase	complete	unclassified Riboviria	hypothetical protein 2 [Jingmen tombus-like virus 2]	97%	1E-126	42.00%	YP_009344965.1
3PE-RDRP-18	MZ375193	lizardfe003	SRX11831397	1741	71	RNA dependent RNA polymerase	partial	unclassified Riboviria	RNA-dependent RNA polymerase [Gervais tombus-like virus]	100%	3E-171	58.00%	QIJ70125.1
3PE-RDRP-19	MZ375194	lizardfe003	SRX11831397	1678	134	RNA dependent RNA polymerase	partial	unclassified Riboviria	hypothetical protein 2 [Hubei tombus-like virus 36]	92%	3E-96	40.41%	QDH89718.1
3PE-RDRP-20	MZ375195	lizardfe003	SRX11831397	1647	116	RNA dependent RNA polymerase	partial	unclassified Riboviria	RNA-dependent RNA polymerase [Soybean thrips sobemo-like virus 5]	91%	0	76.34%	QPZ88401.1
3PE-RDRP-21	MZ375196	lizardfe003	SRX11831397	1607	32	RNA dependent RNA polymerase	partial	unclassified Riboviria	nonstructural polyprotein [Weivirus-like virus sp.]	41%	4E-43	42.74%	QJI53767.1
3PE-RDRP-22	MZ375197	lizardfe003	SRX11831397	1591	24	RNA dependent RNA polymerase	partial	unclassified Riboviria	hypothetical protein 2 [Jingmen tombus-like virus 2]	98%	2E-124	45.13%	YP_009344965.1
3PE-RDRP-23	MZ375198	lizardfe003	SRX11831397	1402	42	RNA dependent RNA polymerase	partial	unclassified Riboviria	RNA-dependent RNA polymerase [Gervais tombus-like virus]	100%	5E-168	62.73%	QIJ70125.1
3PE-RDRP-24	MZ375199	lizardfe003	SRX11831397	1345	3976	RNA dependent RNA polymerase	partial	unclassified Riboviria	hypothetical protein 2 [Hubei diptera virus 14]	94%	1E-156	58.62%	YP_009337875.1
3PE-RDRP-25	MZ375200	lizardfe003	SRX11831397	835	16	RNA dependent RNA polymerase	partial	unclassified Riboviria	hypothetical protein 3 [Hubei sobemo-like virus 13]	99%	3E-109	58.70%	YP_009330098.1
3PE-RDRP-27	MZ375201	lizardfe003	SRX11831397	596	14	RNA dependent RNA polymerase	partial	unclassified Riboviria	hypothetical protein [Blue fish point virus]	99%	8E-90	68.18%	AYP67542.1
3PE-RDRP-29	MZ375225	lizardfe003	SRX11831397	2114	148	RNA dependent RNA polymerase	partial	Solemoviridae	RNA-dependent RNA polymerase [Papaya lethal yellowing virus]	91%	0	64.54%	BBD88560.1
3PE-RDRP-31	MZ375226	lizardfe003	SRX11831397	759	22	RNA dependent RNA polymerase	partial	Solemoviridae	polyprotein P2ab [Lucerne transient streak virus]	93%	7E-130	77.92%	QQP18742.1
3PE-RDRP-36	MZ375227	lizardfe003	SRX11831397	1089	42	RNA dependent RNA polymerase	partial	Totiviridae	RNA-dependent RNA polymerase [Leishmania RNA virus 1]	95%	7E-38	33.01%	APU54698.1
3PE-RDRP-37	MZ375228	lizardfe003	SRX11831397	305	19	RNA dependent RNA polymerase	partial	Virgaviridae	hypothetical protein [Pepper mild mottle virus]	99%	1E-62	100.00%	APG77805.1
3PE-TERL-1	MZ374941	lizardfe003	SRX11831397	2381	22	terminase large subunit	complete	uncultured Caudovirales phage	phage terminase large subunit family protein [[Clostridium] symbiosum]	99%	0	89.23%	WP_202185501.1
3PE-TERL-2	MZ374942	lizardfe003	SRX11831397	2306	237	terminase large subunit	complete	uncultured Caudovirales phage	terminase large subunit [Alistipes sp.]	99%	0	92.43%	MBQ4533116.1
3PE-TERL-3	MZ374943	lizardfe003	SRX11831397	2220	40	terminase large subunit	complete	uncultured Caudovirales phage	terminase large subunit [Clostridiaceae bacterium]	99%	0	68.31%	NLU52370.1
3PE-TERL-4	MZ374944	lizardfe003	SRX11831397	2215	101	terminase large subunit	complete	uncultured Caudovirales phage	terminase large subunit [Firmicutes bacterium]	99%	0	80.07%	NMB00765.1
3PE-TERL-5	MZ374945	lizardfe003	SRX11831397	2148	23	terminase large subunit	complete	uncultured Caudovirales phage	terminase large subunit [Butyricicoccus porcorum]	98%	0	70.52%	WP_087020645.1
3PE-TERL-6	MZ374946	lizardfe003	SRX11831397	2139	86	terminase large subunit	complete	uncultured Caudovirales phage	PBSX family phage terminase large subunit [Anaerostipes caccae]	96%	0	73.75%	WP_083809569.1
3PE-TERL-7	MZ374947	lizardfe003	SRX11831397	2129	26	terminase large subunit	complete	uncultured Caudovirales phage	terminase large subunit [Parabacteroides sp. AF19-14]	99%	0	92.21%	WP_122379962.1
3PE-TERL-8	MZ374948	lizardfe003	SRX11831397	2121	16	terminase large subunit	complete	uncultured Caudovirales phage	terminase [Oscillibacter sp.]	99%	0	75.09%	MBD5169250.1
3PE-TERL-9	MZ374949	lizardfe003	SRX11831397	2063	23	terminase large subunit	partial	uncultured Caudovirales phage	terminase large subunit [Lachnospiraceae bacterium]	98%	0	89.08%	MBD5550227.1
3PE-TERL-10	MZ374950	lizardfe003	SRX11831397	2060	221	terminase large subunit	complete	uncultured Caudovirales phage	terminase large subunit [Blautia sp. OF03-15BH]	99%	0	85.30%	WP_117768406.1

Virus strain name	GenBank No.	LibraryID	SRA Accession No.	Length	Virus Reads	Virus hallmark genes	Completeness of CDS	Classification	Blastx hits on known protein and viruses	Coverage (%)	E-value	Identity (%)	Accession no. of best match
3PE-TERL-11	MZ374951	lizardfe003	SRX11831397	2029	20	terminase large subunit	complete	uncultured Caudovirales phage	phage terminase large subunit [Oscillibacter sp.]	97%	0	71.34%	MBQ5928030.1
3PE-TERL-12	MZ374952	lizardfe003	SRX11831397	1987	42	terminase large subunit	partial	uncultured Caudovirales phage	MULTISPECIES: terminase large subunit [Eubacteriales]	99%	0	95.22%	WP_002591266.1
3PE-TERL-13	MZ374953	lizardfe003	SRX11831397	1979	75	terminase large subunit	complete	uncultured Caudovirales phage	Terminase-like family protein [Lachnospiraceae bacterium AM48-27BH]	98%	0	70.59%	RHQ17436.1
3PE-TERL-14	MZ374954	lizardfe003	SRX11831397	1957	13	terminase large subunit	complete	uncultured Caudovirales phage	phage terminase large subunit [Bacteroides thetaiotaomicron]	99%	0	93.00%	WP_054960548.1
3PE-TERL-15	MZ374955	lizardfe003	SRX11831397	1911	63	terminase large subunit	complete	uncultured Caudovirales phage	PBSX family phage terminase large subunit [Clostridium sp. AM27-31LB]	99%	0	84.53%	WP_118745318.1
3PE-TERL-16	MZ374956	lizardfe003	SRX11831397	1856	26	terminase large subunit	complete	uncultured Caudovirales phage	PBSX family phage terminase large subunit [Clostridium sp. FS41]	99%	0	92.13%	WP_045093877.1
3PE-TERL-17	MZ374957	lizardfe003	SRX11831397	1776	23	terminase large subunit	complete	uncultured Caudovirales phage	hypothetical protein EII22_08935 [Coriobacteriales bacterium OH1046]	97%	0	65.63%	RVU97048.1
3PE-TERL-18	MZ374958	lizardfe003	SRX11831397	1730	21	terminase large subunit	complete	uncultured Caudovirales phage	putative terminase [Escherichia phage K1H]	99%	0	99.28%	YP_009168834.1
3PE-TERL-19	MZ374959	lizardfe003	SRX11831397	1722	53	terminase large subunit	complete	uncultured Caudovirales phage	PBSX family phage terminase large subunit [Collinsella tanakaei]	98%	0	77.58%	PWM20997.1
3PE-TERL-20	MZ374960	lizardfe003	SRX11831397	1692	13	terminase large subunit	partial	uncultured Caudovirales phage	terminase large subunit [[Clostridium] symbiosum]	99%	0	91.03%	WP_195321274.1
3PE-TERL-21	MZ374961	lizardfe003	SRX11831397	1650	29	terminase large subunit	partial	uncultured Caudovirales phage	phage terminase large subunit [Eisenbergiella sp. OF01-20]	99%	0	91.13%	WP_118680996.1
3PE-TERL-22	MZ374962	lizardfe003	SRX11831397	1602	42	terminase large subunit	partial	uncultured Caudovirales phage	hypothetical protein [Spirochaetia bacterium]	89%	1E-137	50.86%	NCB00837.1
3PE-TERL-23	MZ374963	lizardfe003	SRX11831397	1600	25	terminase large subunit	partial	uncultured Caudovirales phage	terminase [Oscillibacter sp.]	99%	0	73.74%	MBD5169250.1
3PE-TERL-24	MZ374964	lizardfe003	SRX11831397	1494	22	terminase large subunit	partial	uncultured Caudovirales phage	hypothetical protein [Clostridia bacterium]	92%	2E-172	60.52%	MBR6688796.1
3PE-TERL-25	MZ374965	lizardfe003	SRX11831397	1462	37	terminase large subunit	partial	uncultured Caudovirales phage	phage terminase large subunit family protein [Intestinimonas butyriciproducens]	99%	0	84.02%	WP_204738349.1
3PE-TERL-26	MZ374966	lizardfe003	SRX11831397	1451	12	terminase large subunit	partial	uncultured Caudovirales phage	hypothetical protein [Bacteroides intestinalis]	97%	0	94.70%	WP_061433606.1
3PE-TERL-27	MZ374967	lizardfe003	SRX11831397	1439	24	terminase large subunit	partial	uncultured Caudovirales phage	phage terminase large subunit [Alistipes finegoldii]	99%	0	93.54%	KAA3157596.1
3PE-TERL-28	MZ374968	lizardfe003	SRX11831397	1409	47	terminase large subunit	partial	uncultured Caudovirales phage	terminase family protein [Bacteroidales bacterium]	99%	1E-156	53.85%	MBQ2398421.1
3PE-TERL-29	MZ374969	lizardfe003	SRX11831397	1319	13	terminase large subunit	partial	uncultured Caudovirales phage	PBSX family phage terminase large subunit [Enterocloster clostridioformis]	99%	0	86.46%	WP_044918678.1
3PE-TERL-30	MZ374970	lizardfe003	SRX11831397	1281	44	terminase large subunit	partial	uncultured Caudovirales phage	PBSX family phage terminase large subunit [Alistipes communis]	99%	0	83.53%	WP_195454277.1
3PE-TERL-31	MZ374971	lizardfe003	SRX11831397	1227	21	terminase large subunit	partial	uncultured Caudovirales phage	PBSX family phage terminase large subunit [Collinsella tanakaei]	98%	0	79.25%	PWM20997.1
3PE-TERL-32	MZ374972	lizardfe003	SRX11831397	1214	14	terminase large subunit	partial	uncultured Caudovirales phage	terminase large subunit [Firmicutes bacterium]	97%	0	81.06%	NMB00765.1
3PE-TERL-33	MZ374973	lizardfe003	SRX11831397	1179	27	terminase large subunit	partial	uncultured Caudovirales phage	PBSX family phage terminase large subunit [Dielma fastidiosa]	99%	0	91.99%	WP_118454122.1
3PE-TERL-34	MZ374974	lizardfe003	SRX11831397	1171	47	terminase large subunit	partial	uncultured Caudovirales phage	terminase family protein [Bacteroidales bacterium]	99%	0	70.17%	MBR6903426.1
3PE-TERL-35	MZ374975	lizardfe003	SRX11831397	1087	62	terminase large subunit	partial	uncultured Caudovirales phage	hypothetical protein [Clostridiales bacterium BX7]	99%	2E-136	71.28%	MBC8537276.1
3PE-TERL-36	MZ374976	lizardfe003	SRX11831397	1047	12	terminase large subunit	partial	uncultured Caudovirales phage	MULTISPECIES: phage terminase large subunit family protein [Lachnospiraceae]	100%	0	80.23%	WP_179960935.1
3PE-TERL-37	MZ374977	lizardfe003	SRX11831397	1031	24	terminase large subunit	partial	uncultured Caudovirales phage	phage terminase large subunit family protein [Pseudoflavonifractor sp. 524-17]	99%	0	87.17%	NCE63876.1
3PE-TERL-38	MZ374978	lizardfe003	SRX11831397	1024	16	terminase large subunit	partial	uncultured Caudovirales phage	terminase large subunit [Mogibacterium sp. BX12]	99%	0	82.35%	WP_187304421.1
3PE-TERL-39	MZ374979	lizardfe003	SRX11831397	1007	17	terminase large subunit	partial	uncultured Caudovirales phage	TPA: terminase large subunit [Desulfitobacterium dehalogenans]	95%	1E-148	64.91%	HHY28080.1
3PE-TERL-40	MZ374980	lizardfe003	SRX11831397	1001	32	terminase large subunit	partial	uncultured Caudovirales phage	phage terminase large subunit [Anaerotruncus sp. X29]	99%	2E-97	61.02%	WP_161910330.1
3PE-TERL-41	MZ374981	lizardfe003	SRX11831397	958	15	terminase large subunit	partial	uncultured Caudovirales phage	hypothetical protein [Slackia faecicanis]	80%	3E-104	74.02%	WP_123197916.1
3PE-TERL-42	MZ374982	lizardfe003	SRX11831397	933	135	terminase large subunit	partial	uncultured Caudovirales phage	phage terminase large subunit [Clostridia bacterium]	91%	5E-74	49.38%	MBQ8689465.1
3PE-TERL-43	MZ374983	lizardfe003	SRX11831397	928	11	terminase large subunit	partial	uncultured Caudovirales phage	terminase large subunit [Clostridium sp. BSD9I1]	99%	6E-108	61.32%	WP_186431101.1
3PE-TERL-44	MZ374984	lizardfe003	SRX11831397	915	12	terminase large subunit	partial	uncultured Caudovirales phage	phage terminase large subunit [Odoribacter splanchnicus]	98%	8E-135	81.39%	WP_087381242.1
3PE-TERL-45	MZ374985	lizardfe003	SRX11831397	899	23	terminase large subunit	partial	uncultured Caudovirales phage	PBSX family phage terminase large subunit [Limosilactobacillus fermentum]	94%	7E-125	78.67%	PWM28080.1
3PE-TERL-46	MZ374986	lizardfe003	SRX11831397	857	12	terminase large subunit	partial	uncultured Caudovirales phage	terminase [[Clostridium] innocuum]	85%	7E-80	67.71%	WP_065531785.1
3PE-TERL-47	MZ374987	lizardfe003	SRX11831397	825	18	terminase large subunit	partial	uncultured Caudovirales phage	hypothetical protein D7X25_33475 [bacterium 1XD42-8]	100%	5E-153	76.00%	RKJ34574.1
3PE-TERL-48	MZ374988	lizardfe003	SRX11831397	768	12	terminase large subunit	partial	uncultured Caudovirales phage	terminase [Oscillibacter sp. MM59]	99%	6E-83	68.85%	BCK82989.1
3PE-TERL-49	MZ374989	lizardfe003	SRX11831397	761	19	terminase large subunit	partial	uncultured Caudovirales phage	phage terminase large subunit [Prevotella sp.]	99%	2E-134	72.20%	MBP5714384.1
3PE-TERL-50	MZ374990	lizardfe003	SRX11831397	638	20	terminase large subunit	partial	uncultured Caudovirales phage	terminase [Alistipes onderdonkii]	99%	2E-99	98.14%	KAA2766038.1
3PE-TERL-51	MZ374991	lizardfe003	SRX11831397	633	16	terminase large subunit	partial	uncultured Caudovirales phage	PBSX family phage terminase large subunit [Hungatella hathewayi]	99%	2E-71	96.55%	WP_006775529.1
4PT-MCP-1	MZ374795	lizardfe004	SRX11831406	5118	71911	major capsid protein	complete	Microviridae	major capsid protein [Tortoise microvirus 49]	99%	0	54.42%	QCS37072.1
4PT-MCP-2	MZ374796	lizardfe004	SRX11831406	957	97	major capsid protein	partial	Microviridae	major capsid protein [Tortoise microvirus 13]	99%	9E-112	64.64%	QCS36834.1
4PT-NS1-1	MZ375129	lizardfe004	SRX11831406	2706	73	nonstructual protein 1	partial	Parvoviridae	NS1 [Ambidensovirus sp.]	99%	4E-89	70.49%	QGZ11177.1
4PT-RDRP-1	MZ375140	lizardfe004	SRX11831406	5062	628953	RNA dependent RNA polymerase	partial	Astroviridae	ORF1ab [Guangxi changeable lizard astrovirus]	94%	0	45.15%	AVM87190.1
4PT-RDRP-2	MZ375153	lizardfe004	SRX11831406	7668	5727	RNA dependent RNA polymerase	complete	Caliciviridae	polyprotein [Guangdong greater green snake calicivirus]	80%	0	34.71%	AVM87221.1
4PT-RDRP-3	MZ375154	lizardfe004	SRX11831406	7535	1785	RNA dependent RNA polymerase	complete	Caliciviridae	polyprotein [Guangdong greater green snake calicivirus]	82%	0	33.78%	AVM87221.1
4PT-RDRP-4	MZ375229	lizardfe004	SRX11831406	876	12	RNA dependent RNA polymerase	partial	Picobirnaviridae	RNA-dependent RNA polymerase [Picobirnavirus sp.]	100%	9E-161	77.40%	QAA77624.1

Virus strain name	GenBank No.	LibraryID	SRA Accession No.	Length	Virus Reads	Virus hallmark genes	Completeness of CDS	Classification	Blastx hits on known protein and viruses	Coverage (%)	E-value	Identity (%)	Accession no. of best match
4PT-RDRP-5	MZ375202	lizardfe004	SRX11831406	2750	3235	RNA dependent RNA polymerase	complete	unclassified Riboviria	hypothetical protein 2 [Hubei diptera virus 14]	98%	1E-154	57.03%	APG75813.1
4PT-RDRP-6	MZ375203	lizardfe004	SRX11831406	2225	47	RNA dependent RNA polymerase	partial	unclassified Riboviria	RdRp [Hubei permutotetra-like virus 6]	90%	1E-139	42.42%	YP_009337318.1
4PT-RDRP-7	MZ375204	lizardfe004	SRX11831406	1238	104	RNA dependent RNA polymerase	partial	unclassified Riboviria	RdRp [Atrato Sobemo-like virus 2]	96%	3E-105	47.14%	QHA33888.1
4PT-TERL-1	MZ374992	lizardfe004	SRX11831406	3371	76	terminase large subunit	complete	uncultured Caudovirales phage	terminase large subunit [Enterocloster citroniae]	99%	0	85.32%	WP_195416351.1
4PT-TERL-2	MZ374993	lizardfe004	SRX11831406	3261	110	terminase large subunit	complete	uncultured Caudovirales phage	phage terminase large subunit family protein [bacterium 1XD42-76]	99%	0	69.74%	NBJ83417.1
4PT-TERL-3	MZ374994	lizardfe004	SRX11831406	3246	1074	terminase large subunit	complete	uncultured Caudovirales phage	phage terminase large subunit family protein [Pectinatus frisingensis]	94%	0	66.92%	WP_196590584.1
4PT-TERL-4	MZ374995	lizardfe004	SRX11831406	2957	185	terminase large subunit	complete	uncultured Caudovirales phage	terminase large subunit [Dorea sp. 5-2]	99%	0	77.85%	WP_016221223.1
4PT-TERL-5	MZ374996	lizardfe004	SRX11831406	2883	175	terminase large subunit	complete	uncultured Caudovirales phage	terminase large subunit [Lachnospiraceae bacterium]	99%	0	90.02%	NBH97180.1
4PT-TERL-6	MZ374997	lizardfe004	SRX11831406	2840	192	terminase large subunit	complete	uncultured Caudovirales phage	terminase [Oscillibacter sp.]	99%	0	75.09%	MBD5169250.1
4PT-TERL-8	MZ374998	lizardfe004	SRX11831406	2638	54	terminase large subunit	complete	uncultured Caudovirales phage	PBSX family phage terminase large subunit [Bacteroides finegoldii]	99%	0	93.60%	WP_195545061.1
4PT-TERL-9	MZ374999	lizardfe004	SRX11831406	2562	7680	terminase large subunit	complete	uncultured Caudovirales phage	PBSX family phage terminase large subunit [Emergencia timonensis]	98%	0	81.46%	WP_067537062.1
4PT-TERL-10	MZ375000	lizardfe004	SRX11831406	2482	99	terminase large subunit	complete	uncultured Caudovirales phage	terminase family protein [Dielma fastidiosa]	91%	0	70.60%	WP_022939325.1
4PT-TERL-11	MZ375001	lizardfe004	SRX11831406	2361	137	terminase large subunit	complete	uncultured Caudovirales phage	phage terminase large subunit [Robinsoniella sp. KNHs210]	99%	0	82.80%	WP_044290374.1
4PT-TERL-12	MZ375002	lizardfe004	SRX11831406	2359	96	terminase large subunit	complete	uncultured Caudovirales phage	phage terminase large subunit [Oscillospiraceae bacterium]	88%	0	76.45%	MBQ9167712.1
4PT-TERL-13	MZ375003	lizardfe004	SRX11831406	2232	173	terminase large subunit	complete	uncultured Caudovirales phage	hypothetical protein [Clostridia bacterium]	94%	0	56.37%	MBR6688796.1
4PT-TERL-14	MZ375004	lizardfe004	SRX11831406	2130	189	terminase large subunit	complete	uncultured Caudovirales phage	PBSX family phage terminase large subunit [Hungatella hathewayi]	99%	0	94.99%	WP_117622975.1
4PT-TERL-15	MZ375005	lizardfe004	SRX11831406	1964	17	terminase large subunit	complete	uncultured Caudovirales phage	terminase [Hungatella hathewayi]	99%	0	79.47%	WP_055651533.1
4PT-TERL-16	MZ375006	lizardfe004	SRX11831406	1959	34	terminase large subunit	complete	uncultured Caudovirales phage	phage terminase large subunit [Anaerotruncus rubiinfantis]	97%	0	70.60%	WP_066460208.1
4PT-TERL-17	MZ375007	lizardfe004	SRX11831406	1881	43	terminase large subunit	partial	uncultured Caudovirales phage	Phage terminase, large subunit GpA [Tepidibacter formicigenes DSM 15518]	93%	0	58.79%	SHJ74667.1
4PT-TERL-18	MZ375008	lizardfe004	SRX11831406	1806	34	terminase large subunit	partial	uncultured Caudovirales phage	phage terminase large subunit [Anaerotruncus sp. X29]	98%	0	66.81%	WP_161910330.1
4PT-TERL-19	MZ375009	lizardfe004	SRX11831406	1805	42	terminase large subunit	partial	uncultured Caudovirales phage	terminase large subunit [Clostridiales bacterium]	99%	0	85.34%	MBD5086687.1
4PT-TERL-20	MZ375010	lizardfe004	SRX11831406	1717	34	terminase large subunit	partial	uncultured Caudovirales phage	terminase large subunit [[Clostridium] populeti]	100%	0	98.66%	WP_207649493.1
4PT-TERL-21	MZ375011	lizardfe004	SRX11831406	1716	52	terminase large subunit	partial	uncultured Caudovirales phage	phage terminase large subunit [Bacteroides salyersiae]	99%	0	91.62%	WP_149986531.1
4PT-TERL-22	MZ375012	lizardfe004	SRX11831406	1695	43	terminase large subunit	partial	uncultured Caudovirales phage	terminase large subunit [Caproiciproducens galactitolivorans]	99%	0	74.86%	WP_135661135.1
4PT-TERL-23	MZ375013	lizardfe004	SRX11831406	1599	43	terminase large subunit	partial	uncultured Caudovirales phage	phage terminase GpA [Firmicutes bacterium CAG:124]	100%	6E-175	75.55%	CCY40045.1
4PT-TERL-24	MZ375014	lizardfe004	SRX11831406	1588	61	terminase large subunit	complete	uncultured Caudovirales phage	hypothetical protein [Spirochaetia bacterium]	90%	4E-137	49.10%	NCB00837.1
4PT-TERL-25	MZ375015	lizardfe004	SRX11831406	1576	18	terminase large subunit	partial	uncultured Caudovirales phage	terminase large subunit [Blautia sp. OF03-15BH]	99%	0	88.89%	WP_117768406.1
4PT-TERL-26	MZ375016	lizardfe004	SRX11831406	1571	25	terminase large subunit	partial	uncultured Caudovirales phage	terminase [Desulfovibrionaceae bacterium]	99%	0	80.97%	PWL61191.1
4PT-TERL-27	MZ375017	lizardfe004	SRX11831406	1518	48	terminase large subunit	partial	uncultured Caudovirales phage	phage terminase large subunit [Agathobaculum desmolans]	88%	2E-132	66.44%	WP_031475790.1
4PT-TERL-28	MZ375018	lizardfe004	SRX11831406	1502	28	terminase large subunit	partial	uncultured Caudovirales phage	terminase large subunit [Firmicutes bacterium]	95%	0	79.58%	NMB00765.1
4PT-TERL-29	MZ375019	lizardfe004	SRX11831406	1477	21	terminase large subunit	partial	uncultured Caudovirales phage	hypothetical protein BHV75_13770 [Bacteroides oleiciplenus]	99%	0	86.97%	OKZ08861.1
4PT-TERL-30	MZ375020	lizardfe004	SRX11831406	1389	17	terminase large subunit	partial	uncultured Caudovirales phage	terminase family protein [Bacteroidales bacterium]	99%	0	72.38%	MBR3286951.1
4PT-TERL-31	MZ375021	lizardfe004	SRX11831406	1363	42	terminase large subunit	partial	uncultured Caudovirales phage	terminase [Catonella massiliensis]	99%	5E-156	68.30%	WP_208430409.1
4PT-TERL-32	MZ375022	lizardfe004	SRX11831406	1351	18	terminase large subunit	partial	uncultured Caudovirales phage	phage terminase large subunit family protein [Anaerotruncus colihominis]	88%	2E-119	79.30%	WP_207731275.1
4PT-TERL-33	MZ375023	lizardfe004	SRX11831406	1347	13	terminase large subunit	partial	uncultured Caudovirales phage	phage terminase large subunit [Prevotella nanceiensis]	96%	2E-177	68.51%	MBF1421922.1
4PT-TERL-35	MZ375024	lizardfe004	SRX11831406	1257	39	terminase large subunit	partial	uncultured Caudovirales phage	phage terminase, large subunit, PBSX family [Sporomusa termitida]	99%	2E-153	63.84%	QDR80223.1
4PT-TERL-36	MZ375025	lizardfe004	SRX11831406	1210	14	terminase large subunit	partial	uncultured Caudovirales phage	terminase large subunit [Adlercreutzia caecimuris]	97%	2E-78	65.17%	WP_136434908.1
4PT-TERL-38	MZ375026	lizardfe004	SRX11831406	1158	23	terminase large subunit	partial	uncultured Caudovirales phage	PBSX family phage terminase large subunit [Ruminococcus sp.]	99%	0	85.68%	MBD5145043.1
4PT-TERL-39	MZ375027	lizardfe004	SRX11831406	1115	12	terminase large subunit	partial	uncultured Caudovirales phage	phage terminase large subunit [Dysgonomonas sp. GY75]	94%	2E-168	62.97%	WP_194225733.1
4PT-TERL-40	MZ375028	lizardfe004	SRX11831406	1103	11	terminase large subunit	partial	uncultured Caudovirales phage	PBSX family phage terminase large subunit [Hungatella hathewayi]	99%	2E-138	87.85%	RGY92527.1
4PT-TERL-41	MZ375029	lizardfe004	SRX11831406	1101	15	terminase large subunit	partial	uncultured Caudovirales phage	terminase large subunit [Eggerthella lenta]	99%	6E-159	61.16%	WP_035585337.1
4PT-TERL-42	MZ375030	lizardfe004	SRX11831406	1083	15	terminase large subunit	partial	uncultured Caudovirales phage	phage terminase large subunit [Bacteroides ovatus]	99%	0	95.29%	KAB1331208.1
4PT-TERL-43	MZ375031	lizardfe004	SRX11831406	1033	8	terminase large subunit	partial	uncultured Caudovirales phage	terminase large subunit [Bacteroides thetaiotaomicron]	96%	0	92.33%	RHI48344.1
4PT-TERL-44	MZ375032	lizardfe004	SRX11831406	1019	16	terminase large subunit	partial	uncultured Caudovirales phage	PBSX family phage terminase large subunit [Hungatella sp. L12]	97%	0	94.17%	WP_207729206.1
4PT-TERL-45	MZ375033	lizardfe004	SRX11831406	1018	23	terminase large subunit	partial	uncultured Caudovirales phage	phage terminase large subunit [Anaerotruncus sp. X29]	98%	3E-124	59.80%	WP_161910330.1
4PT-TERL-46	MZ375034	lizardfe004	SRX11831406	1002	17	terminase large subunit	partial	uncultured Caudovirales phage	terminase [Lacrimispora saccharolytica]	99%	0	82.61%	WP_013271461.1
4PT-TERL-48	MZ375035	lizardfe004	SRX11831406	958	13	terminase large subunit	partial	uncultured Caudovirales phage	terminase [[Clostridium] innocuum]	99%	1E-129	81.06%	WP_055200468.1
5PT-MCP-1	MZ374797	lizardfe005	SRX11831413	6427	4217	major capsid protein	complete	Microviridae	major capsid protein [Chicken microvirus mg8_45]	99%	0	60.00%	WP_118219241.1

Virus strain name	GenBank No.	LibraryID	SRA Accession No.	Length	Virus Reads	Virus hallmark genes	Completeness of CDS	Classification	Blastx hits on known protein and viruses	Coverage (%)	E-value	Identity (%)	Accession no. of best match
5PT-MCP-2	MZ374798	lizardfe005	SRX11831413	5990	1194	major capsid protein	complete	Microviridae	major capsid protein [Microvirus sp.]	98%	8E-17	23.22%	AXH73451.1
5PT-MCP-3	MZ374799	lizardfe005	SRX11831413	5860	595	major capsid protein	complete	Microviridae	major capsid protein [Microviridae sp.]	99%	0	53.21%	AXH75286.1
5PT-MCP-4	MZ374800	lizardfe005	SRX11831413	5650	913	major capsid protein	complete	Microviridae	major capsid protein [Tortoise microvirus 22]	42%	7E-18	27.56%	MBO5801551.1
5PT-MCP-5	MZ374801	lizardfe005	SRX11831413	2401	6577	major capsid protein	complete	Microviridae	major capsid protein [Microvirus sp.]	99%	0	42.26%	QRV61816.1
5PT-MCP-6	MZ374802	lizardfe005	SRX11831413	2035	47	major capsid protein	complete	Microviridae	major capsid protein [Tortoise microvirus 22]	98%	1E-90	36.79%	QCS36898.1
5PT-MCP-7	MZ374803	lizardfe005	SRX11831413	1966	182	major capsid protein	complete	Microviridae	major capsid protein [Tortoise microvirus 22]	98%	9E-112	37.32%	QCS36898.1
5PT-MCP-8	MZ374804	lizardfe005	SRX11831413	1730	22	major capsid protein	complete	Microviridae	major capsid protein [Tortoise microvirus 33]	65%	0	100.00%	WP_188393631.1
5PT-MCP-9	MZ374805	lizardfe005	SRX11831413	1694	50	major capsid protein	partial	Microviridae	major capsid protein [Tortoise microvirus 33]	97%	2E-94	40.47%	QCS36953.1
5PT-MCP-10	MZ374806	lizardfe005	SRX11831413	1235	160	major capsid protein	partial	Microviridae	major capsid protein [Microviridae sp.]	99%	1E-97	51.40%	AXQ66152.1
5PT-RDRP-1	MZ375141	lizardfe005	SRX11831413	8068	112628	RNA dependent RNA polymerase	complete	Astroviridae	ORF1ab [Guangxi changeable lizard astrovirus]	89%	0	45.25%	AVM87190.1
5PT-RDRP-2	MZ375142	lizardfe005	SRX11831413	7215	67865	RNA dependent RNA polymerase	complete	Astroviridae	ORF1ab [Hainan oriental leaf-toed gecko astrovirus 1]	99%	0	73.18%	AVM87517.1
5PT-RDRP-3	MZ375143	lizardfe005	SRX11831413	5463	3677	RNA dependent RNA polymerase	complete	Astroviridae	ORF1ab [Guangxi changeable lizard astrovirus]	89%	0	45.07%	AVM87190.1
5PT-RDRP-4	MZ375144	lizardfe005	SRX11831413	3569	21561	RNA dependent RNA polymerase	complete	Astroviridae	ORF1ab [Hainan oriental leaf-toed gecko astrovirus 1]	99%	0	76.00%	AVM87517.1
5PT-RDRP-5	MZ375145	lizardfe005	SRX11831413	1683	2798	RNA dependent RNA polymerase	partial	Astroviridae	ORF1ab [Hainan oriental leaf-toed gecko astrovirus 1]	99%	0	73.24%	AVM87518.1
5PT-RDRP-6	MZ375146	lizardfe005	SRX11831413	1137	35	RNA dependent RNA polymerase	partial	Astroviridae	ORF1ab [Guangxi changeable lizard astrovirus]	74%	3E-83	65.96%	AVM87190.1
5PT-RDRP-7	MZ375155	lizardfe005	SRX11831413	1293	44	RNA dependent RNA polymerase	partial	Picornaviridae	replicase [Cherry virus Trakiya]	100%	0	77.24%	YP_009551963.1
5PT-RDRP-8	MZ375156	lizardfe005	SRX11831413	1262	30	RNA dependent RNA polymerase	partial	Picornaviridae	replicase [Cherry virus Trakiya]	98%	0	81.11%	YP_009551963.1
5PT-RDRP-9	MZ375157	lizardfe005	SRX11831413	10391	4793	RNA dependent RNA polymerase	partial	Picornaviridae	hypothetical protein [Picornavirales sp.]	86%	0	43.74%	QJI53588.1
5PT-RDRP-10	MZ375158	lizardfe005	SRX11831413	7287	883	RNA dependent RNA polymerase	partial	Picornaviridae	polyprotein [livupivirus A1]	95%	0	42.12%	APM87483.1
5PT-RDRP-11	MZ375159	lizardfe005	SRX11831413	6423	5563	RNA dependent RNA polymerase	partial	Picornaviridae	polyprotein [Guangxi changeable lizard picornavirus 2]	99%	0	59.16%	AVM87456.1
5PT-RDRP-13	MZ375205	lizardfe005	SRX11831413	1621	39	RNA dependent RNA polymerase	partial	unclassified Riboviria	RdRp [Hubei partiti-like virus 10]	98%	0	53.33%	APG78227.1
5PT-RDRP-14	MZ375206	lizardfe005	SRX11831413	1299	629	RNA dependent RNA polymerase	complete	unclassified Riboviria	hypothetical protein 2 [Hubei sobemo-like virus 42]	98%	6E-175	64.89%	YP_009330107.1
5PT-RDRP-15	MZ375207	lizardfe005	SRX11831413	1196	17	RNA dependent RNA polymerase	partial	unclassified Riboviria	RNA-dependent RNA polymerase [Alphapermutotetravirus sp.]	99%	5E-174	81.96%	QNJ60189.1
5PT-RDRP-16	MZ375208	lizardfe005	SRX11831413	843	11	RNA dependent RNA polymerase	partial	unclassified Riboviria	RNA-dependent RNA polymerase [Soybean thrips tombus-like virus 7]	84%	6E-20	37.21%	QPZ88361.1
5PT-TERL-1	MZ375036	lizardfe005	SRX11831413	2767	239	terminase large subunit	complete	uncultured Caudovirales phage	terminase large subunit [Dorea sp. 5-2]	99%	0	80.78%	WP_016221223.1
5PT-TERL-2	MZ375037	lizardfe005	SRX11831413	2510	53	terminase large subunit	complete	uncultured Caudovirales phage	terminase [Hydrogeniiclostidium mannosilyticum]	99%	0	78.96%	WP_112333774.1
5PT-TERL-3	MZ375038	lizardfe005	SRX11831413	2491	12	terminase large subunit	complete	uncultured Caudovirales phage	phage terminase large subunit family protein [bacterium 1XD42-76]	99%	0	69.42%	NBJ83417.1
5PT-TERL-4	MZ375039	lizardfe005	SRX11831413	2351	65	terminase large subunit	partial	uncultured Caudovirales phage	phage terminase large subunit family protein [Pseudoflavonifractor sp. 524-171]	81%	0	80.62%	NCE63876.1
5PT-TERL-5	MZ375040	lizardfe005	SRX11831413	2267	245	terminase large subunit	complete	uncultured Caudovirales phage	PBSX family phage terminase large subunit [Emergencia timonensis]	98%	0	81.46%	WP_067537062.1
5PT-TERL-6	MZ375041	lizardfe005	SRX11831413	2133	32	terminase large subunit	complete	uncultured Caudovirales phage	PBSX family phage terminase large subunit [Clostridia bacterium]	98%	0	58.85%	NCB52458.1
5PT-TERL-7	MZ375042	lizardfe005	SRX11831413	2068	22	terminase large subunit	partial	uncultured Caudovirales phage	phage terminase large subunit family protein [bacterium 1XD42-76]	100%	0	87.14%	NBJ83417.1
5PT-TERL-8	MZ375043	lizardfe005	SRX11831413	1376	34	terminase large subunit	partial	uncultured Caudovirales phage	terminase family protein [Bacteroides stercoris]	94%	3E-63	34.07%	WP_117984748.1
5PT-TERL-9	MZ375044	lizardfe005	SRX11831413	1127	9	terminase large subunit	partial	uncultured Caudovirales phage	putative terminase [Escherichia phage K1H]	100%	0	98.91%	YP_009168834.1
6PT-MCP-1	MZ374807	lizardfe006	SRX11831441	4545	16496	major capsid protein	complete	Microviridae	major capsid protein [Microviridae sp.]	99%	0	55.58%	AXL14917.1
6PT-MCP-2	MZ374808	lizardfe006	SRX11831441	3375	604	major capsid protein	partial	Microviridae	major capsid protein [Microviridae sp.]	99%	0	52.48%	AXF52699.1
6PT-MCP-3	MZ374809	lizardfe006	SRX11831441	2912	2426	major capsid protein	complete	Microviridae	major capsid protein [Tortoise microvirus 22]	98%	9E-112	37.32%	QCS36898.1
6PT-MCP-4	MZ374810	lizardfe006	SRX11831441	2865	11651	major capsid protein	complete	Microviridae	major capsid protein [Tortoise microvirus 1]	99%	0	60.62%	WP_118219241.1
6PT-MCP-5	MZ374811	lizardfe006	SRX11831441	2575	2649	major capsid protein	complete	Microviridae	major capsid protein [Tortoise microvirus 97]	99%	0	60.81%	SCH20855.1
6PT-MCP-6	MZ374812	lizardfe006	SRX11831441	2556	6036	major capsid protein	complete	Microviridae	major capsid protein [Chicken microvirus mg7_6]	99%	0	59.87%	WP_118219241.1
6PT-MCP-7	MZ374813	lizardfe006	SRX11831441	2358	13838	major capsid protein	complete	Microviridae	major capsid protein [Microvirus sp.]	99%	0	58.11%	SCH20855.1
6PT-MCP-8	MZ374814	lizardfe006	SRX11831441	1427	47	major capsid protein	partial	Microviridae	major capsid protein [Microvirus sp.]	93%	2E-18	28.01%	AXH73118.1
6PT-MCP-9	MZ374815	lizardfe006	SRX11831441	2145	23720	major capsid protein	partial	Microviridae	major capsid protein [Tortoise microvirus 1]	99%	0	59.06%	SCH20855.1
6PT-MCP-11	MZ374816	lizardfe006	SRX11831441	1639	9984	major capsid protein	partial	Microviridae	major capsid protein [Chicken microvirus mg8_45]	99%	3E-100	46.04%	WP_195532903.1
6PT-MCP-12	MZ374817	lizardfe006	SRX11831441	1601	1150	major capsid protein	partial	Microviridae	major capsid protein [Tortoise microvirus 22]	99%	3E-68	37.21%	QCS36898.1
6PT-MCP-13	MZ374818	lizardfe006	SRX11831441	1554	1769	major capsid protein	partial	Microviridae	major capsid protein [Tortoise microvirus 22]	95%	7E-88	36.76%	QCS36898.1
6PT-MCP-14	MZ374819	lizardfe006	SRX11831441	1523	769	major capsid protein	partial	Microviridae	major capsid protein [Tortoise microvirus 22]	99%	2E-68	37.18%	QCS36898.1
6PT-MCP-15	MZ374820	lizardfe006	SRX11831441	1399	169	major capsid protein	partial	Microviridae	major capsid protein [Chicken microvirus mg8_45]	99%	2E-139	52.19%	SCH20855.1
6PT-MCP-16	MZ374821	lizardfe006	SRX11831441	1257	9829	major capsid protein	partial	Microviridae	major capsid protein [Tortoise microvirus 1]	100%	4E-95	44.72%	WP_195532903.1

Virus strain name	GenBank No.	LibraryID	SRA Accession No.	Length	Virus Reads	Virus hallmark genes	Completeness of CDS	Classification	Blastx hits on known protein and viruses	Coverage (%)	E-value	Identity (%)	Accession no. of best match
6PT-MCP-17	MZ374822	lizardfe006	SRX11831441	1178	8486	major capsid protein	partial	Microviridae	major capsid protein [Chicken microvirus mg8_45]	99%	0	75.32%	SCH20855.1
6PT-MCP-18	MZ374823	lizardfe006	SRX11831441	1073	14	major capsid protein	partial	Microviridae	major capsid protein [Microviridae sp.]	98%	2E-12	25.71%	AXF52770.1
6PT-NS1-1	MZ375130	lizardfe006	SRX11831441	5037	191	nonstructual protein 1	complete	Parvoviridae	NS1 [Ambidensovirus sp.]	99%	0	60.25%	QGZ11177.1
6PT-RDRP-1	MZ375147	lizardfe006	SRX11831441	5265	480936	RNA dependent RNA polymerase	partial	Astroviridae	ORF1ab [Guangxi changeable lizard astrovirus]	87%	0	45.07%	AVM87190.1
6PT-RDRP-2	MZ375148	lizardfe006	SRX11831441	2203	7781	RNA dependent RNA polymerase	partial	Astroviridae	ORF1ab [Guangxi changeable lizard astrovirus]	74%	0	66.81%	AVM87190.1
6PT-RDRP-3	MZ375149	lizardfe006	SRX11831441	1644	128173	RNA dependent RNA polymerase	partial	Astroviridae	ORF1b [Hainan gekko similignum astrovirus]	99%	0	74.94%	AVM87517.1
6PT-RDRP-4	MZ375150	lizardfe006	SRX11831441	959	35291	RNA dependent RNA polymerase	partial	Astroviridae	ORF1b [Hainan gekko similignum astrovirus]	99%	5E-166	72.64%	AVM87518.1
6PT-RDRP-5	MZ375209	lizardfe006	SRX11831441	6235	1414	RNA dependent RNA polymerase	complete	unclassified Riboviria	hypothetical protein 1 [Beihai sipunculid worm virus 5]	53%	1E-82	29.93%	YP_009333461.1
6PT-RDRP-6	MZ375160	lizardfe006	SRX11831441	10295	6091	RNA dependent RNA polymerase	partial	Picornaviridae	hypothetical protein [Picornavirales sp.]	86%	0	43.82%	QJI53588.1
6PT-RDRP-10	MZ375210	lizardfe006	SRX11831441	1428	1427	RNA dependent RNA polymerase	partial	unclassified Riboviria	hypothetical protein [Sanxia water strider virus 9]	99%	0	66.17%	YP_009337438.1
6PT-RDRP-11	MZ375211	lizardfe006	SRX11831441	1343	480	RNA dependent RNA polymerase	partial	unclassified Riboviria	RdRp [Hubei partiti-like virus 48]	96%	3E-129	49.18%	APG78218.1
6PT-RDRP-12	MZ375212	lizardfe006	SRX11831441	739	11	RNA dependent RNA polymerase	partial	unclassified Riboviria	hypothetical protein 2 [Hubei unio douglasiae virus 2]	99%	1E-114	89.39%	YP_009336653.1
6PT-RDRP-13	MZ375213	lizardfe006	SRX11831441	2746	1274	RNA dependent RNA polymerase	complete	unclassified Riboviria	hypothetical protein 2 [Solemoviridae sp.]	99%	3E-104	52.96%	YP_009330005.1
6PT-RDRP-14	MZ375214	lizardfe006	SRX11831441	2213	4648	RNA dependent RNA polymerase	partial	unclassified Riboviria	hypothetical protein 2 [Hubei tombus-like virus 13]	99%	4E-110	37.50%	YP_009337096.1
6PT-RDRP-15	MZ375215	lizardfe006	SRX11831441	1696	92	RNA dependent RNA polymerase	partial	unclassified Riboviria	hypothetical protein 2 [Hubei tombus-like virus 23]	96%	1E-101	52.16%	YP_009336823.1
6PT-TERL-1	MZ375045	lizardfe006	SRX11831441	3434	264	terminase large subunit	complete	uncultured Caudovirales phage	phage terminase large subunit [Bacteroides xylanisolvens]	99%	0	85.83%	WP_087318669.1
6PT-TERL-2	MZ375046	lizardfe006	SRX11831441	3267	150	terminase large subunit	complete	uncultured Caudovirales phage	phage terminase large subunit family protein [Anaeromassilibacillus senegalensis]	98%	0	90.14%	WP_050697989.1
6PT-TERL-3	MZ375047	lizardfe006	SRX11831441	3156	196	terminase large subunit	complete	uncultured Caudovirales phage	terminase [Blautia glucerasea]	99%	0	75.56%	WP_173726525.1
6PT-TERL-4	MZ375048	lizardfe006	SRX11831441	3052	106	terminase large subunit	complete	uncultured Caudovirales phage	phage terminase large subunit family protein [Pseudoflavonifractor sp. 524-17]	99%	0	82.91%	NCE63876.1
6PT-TERL-5	MZ375049	lizardfe006	SRX11831441	2982	457	terminase large subunit	complete	uncultured Caudovirales phage	terminase large subunit [Dorea sp. 5-2]	98%	0	77.22%	WP_016221223.1
6PT-TERL-6	MZ375050	lizardfe006	SRX11831441	2922	348	terminase large subunit	complete	uncultured Caudovirales phage	terminase large subunit [Peptococcaceae bacterium]	99%	0	74.91%	NTW04541.1
6PT-TERL-7	MZ375051	lizardfe006	SRX11831441	2851	76	terminase large subunit	complete	uncultured Caudovirales phage	phage terminase large subunit family protein [Lachnospiraceae bacterium]	95%	0	72.32%	MBO5145022.1
6PT-TERL-8	MZ375052	lizardfe006	SRX11831441	2792	105	terminase large subunit	complete	uncultured Caudovirales phage	terminase large subunit [IAS virus]	97%	0	62.69%	YP_009981724.1
6PT-TERL-9	MZ375053	lizardfe006	SRX11831441	2780	65	terminase large subunit	complete	uncultured Caudovirales phage	phage terminase large subunit [Alistipes finegoldii]	99%	0	76.26%	KAA3157596.1
6PT-TERL-10	MZ375054	lizardfe006	SRX11831441	2722	66	terminase large subunit	complete	uncultured Caudovirales phage	terminase large subunit [Firmicutes bacterium]	99%	0	80.57%	NMB00765.1
6PT-TERL-11	MZ375055	lizardfe006	SRX11831441	2707	72	terminase large subunit	complete	uncultured Caudovirales phage	phage terminase large subunit [Prevotella sp.]	99%	0	69.22%	MBO4849872.1
6PT-TERL-12	MZ375056	lizardfe006	SRX11831441	2636	115	terminase large subunit	complete	uncultured Caudovirales phage	terminase [Hydrogeniiclostidium mannosilyticum]	99%	0	78.60%	WP_112333774.1
6PT-TERL-13	MZ375057	lizardfe006	SRX11831441	2605	183	terminase large subunit	complete	uncultured Caudovirales phage	terminase [Hespellia stercorisuis]	99%	0	72.27%	WP_084533959.1
6PT-TERL-14	MZ375058	lizardfe006	SRX11831441	2568	66	terminase large subunit	complete	uncultured Caudovirales phage	terminase [Clostridiales bacterium]	99%	0	69.88%	NLK76243.1
6PT-TERL-15	MZ375059	lizardfe006	SRX11831441	2555	82	terminase large subunit	complete	uncultured Caudovirales phage	PBSX family phage terminase large subunit [Clostridium jeddahense]	96%	4E-172	60.87%	WP_052335731.1
6PT-TERL-16	MZ375060	lizardfe006	SRX11831441	2509	69	terminase large subunit	complete	uncultured Caudovirales phage	PBSX family phage terminase large subunit [Anaeromassilibacillus senegalensis]	99%	0	96.12%	WP_050697715.1
6PT-TERL-17	MZ3750061	lizardfe006	SRX11831441	2355	70	terminase large subunit	complete	uncultured Caudovirales phage	PBSX family phage terminase large subunit [Clostridioides difficile]	90%	9E-112	43.40%	WP_207305839.1
6PT-TERL-18	MZ375062	lizardfe006	SRX11831441	2352	41	terminase large subunit	complete	uncultured Caudovirales phage	terminase large subunit [Bacteroides stercorirosoris]	99%	0	87.69%	WP_083595459.1
6PT-TERL-19	MZ375063	lizardfe006	SRX11831441	2348	89	terminase large subunit	complete	uncultured Caudovirales phage	TPA: PBSX family phage terminase large subunit [Candidatus Gastranaerophilales bacterium HUM_21]	99%	0	90.93%	DAB24131.1
6PT-TERL-20	MZ375064	lizardfe006	SRX11831441	2291	52	terminase large subunit	partial	uncultured Caudovirales phage	phage terminase large subunit family protein [Pseudoflavonifractor sp. 524-17]	79%	0	80.39%	NCE63876.1
6PT-TERL-22	MZ375065	lizardfe006	SRX11831441	2288	392	terminase large subunit	complete	uncultured Caudovirales phage	terminase family protein [Parabacteroides goldsteinii]	99%	0	92.32%	WP_195585337.1
6PT-TERL-23	MZ375066	lizardfe006	SRX11831441	2271	43	terminase large subunit	partial	uncultured Caudovirales phage	phage terminase large subunit family protein [Clostridiaceae bacterium]	98%	0	80.70%	NBH77717.1
6PT-TERL-24	MZ375067	lizardfe006	SRX11831441	2244	148	terminase large subunit	partial	uncultured Caudovirales phage	terminase family protein [Bacteroides stercoris]	90%	0	79.58%	WP_117984748.1
6PT-TERL-25	MZ375068	lizardfe006	SRX11831441	2135	42	terminase large subunit	complete	uncultured Caudovirales phage	hypothetical protein [Lachnoclostridium sp. An169]	97%	0	79.25%	WP_191404612.1
6PT-TERL-26	MZ375069	lizardfe006	SRX11831441	2132	33	terminase large subunit	partial	uncultured Caudovirales phage	phage terminase large subunit family protein [Selenomonas sp.]	99%	0	70.03%	MBF1689006.1
6PT-TERL-27	MZ375070	lizardfe006	SRX11831441	2126	58	terminase large subunit	complete	uncultured Caudovirales phage	terminase large subunit [Parabacteroides sp. TM07-1AC]	96%	0	85.50%	RHU31027.1
6PT-TERL-28	MZ375071	lizardfe006	SRX11831441	2104	50	terminase large subunit	complete	uncultured Caudovirales phage	terminase [Mogibacterium pumilum]	97%	0	60.43%	WP_094233631.1
6PT-TERL-29	MZ375072	lizardfe006	SRX11831441	1942	32	terminase large subunit	partial	uncultured Caudovirales phage	terminase [Intestinimonas timonensis]	98%	0	70.46%	WP_130850784.1
6PT-TERL-30	MZ375073	lizardfe006	SRX11831441	1831	50	terminase large subunit	partial	uncultured Caudovirales phage	PBSX family phage terminase large subunit [Bacteroides uniformis]	96%	0	90.51%	WP_151852711.1
6PT-TERL-31	MZ375074	lizardfe006	SRX11831441	1775	192	terminase large subunit	partial	uncultured Caudovirales phage	hypothetical protein [Odoribacter sp. OF09-27XD]	90%	7E-131	45.85%	WP_118774265.1
6PT-TERL-33	MZ375075	lizardfe006	SRX11831441	1688	28	terminase large subunit	partial	uncultured Caudovirales phage	terminase [Lachnospiraceae bacterium]	99%	0	91.69%	NBH27241.1
6PT-TERL-34	MZ375076	lizardfe006	SRX11831441	1679	16	terminase large subunit	partial	uncultured Caudovirales phage	putative terminase [Escherichia phage K1H]	99%	0	98.11%	YP_009168834.1
6PT-TERL-35	MZ375077	lizardfe006	SRX11831441	1665	33	terminase large subunit	partial	uncultured Caudovirales phage	hypothetical protein [Firmicutes bacterium]	99%	0	75.54%	MBR2511902.1

Virus strain name	GenBank No.	LibraryID	SRA Accession No.	Length	Virus Reads	Virus hallmark genes	Completeness of CDS	Classification	Blastx hits on known protein and viruses	Coverage (%)	E-value	Identity (%)	Accession no. of best match
6PT-TERL-36	MZ375078	lizardfe006	SRX11831441	1631	312	terminase large subunit	partial	uncultured Caudovirales phage	PBSX family phage terminase large subunit [Mailhella sp.]	98%	0	64.00%	MBO4317255.1
6PT-TERL-38	MZ375079	lizardfe006	SRX11831441	1596	50	terminase large subunit	partial	uncultured Caudovirales phage	PBSX family phage terminase large subunit [Bacteroides faecis]	95%	2E-180	73.11%	WP_130062192.1
6PT-TERL-39	MZ375080	lizardfe006	SRX11831441	1595	27	terminase large subunit	partial	uncultured Caudovirales phage	terminase [Lacrimispora saccharolytica]	98%	8E-149	65.00%	WP_013271461.1
6PT-TERL-40	MZ375081	lizardfe006	SRX11831441	1592	24	terminase large subunit	partial	uncultured Caudovirales phage	hypothetical protein [Faecalicatena fissicatena]	99%	0	79.95%	WP_087148937.1
6PT-TERL-41	MZ375082	lizardfe006	SRX11831441	1562	33	terminase large subunit	partial	uncultured Caudovirales phage	hypothetical protein [Bacteroides caccae]	99%	0	97.24%	WP_005676022.1
6PT-TERL-42	MZ375083	lizardfe006	SRX11831441	1480	37	terminase large subunit	partial	uncultured Caudovirales phage	terminase family protein [Bacteroidales bacterium]	99%	0	68.99%	MBR3286951.1
6PT-TERL-43	MZ375084	lizardfe006	SRX11831441	1454	16	terminase large subunit	partial	uncultured Caudovirales phage	PBSX family phage terminase large subunit [Candidatus Gastranaerophilales bacterium]	97%	5E-99	64.78%	MBQ4646389.1
6PT-TERL-44	MZ375085	lizardfe006	SRX11831441	1443	33	terminase large subunit	partial	uncultured Caudovirales phage	hypothetical protein DBY37_03210 [Desulfovibrionaceae bacterium]	95%	2E-175	54.64%	PWL63846.1
6PT-TERL-45	MZ375086	lizardfe006	SRX11831441	1411	30	terminase large subunit	partial	uncultured Caudovirales phage	terminase family protein [bacterium]	95%	4E-146	54.74%	MBQ2872329.1
6PT-TERL-46	MZ375087	lizardfe006	SRX11831441	1383	21	terminase large subunit	partial	uncultured Caudovirales phage	phage terminase large subunit family protein [Clostridium sp. TF11-13AC]	97%	0	82.85%	WP_118638210.1
6PT-TERL-47	MZ375088	lizardfe006	SRX11831441	1375	29	terminase large subunit	partial	uncultured Caudovirales phage	PBSX family phage terminase large subunit [Intestinimonas butyriciproducens]	99%	0	88.79%	WP_116722489.1
6PT-TERL-48	MZ375089	lizardfe006	SRX11831441	1357	21	terminase large subunit	partial	uncultured Caudovirales phage	PBSX family phage terminase large subunit [Anaerotruncus rubiinfantis]	99%	2E-141	70.92%	WP_066454558.1
6PT-TERL-49	MZ375090	lizardfe006	SRX11831441	1344	22	terminase large subunit	partial	uncultured Caudovirales phage	terminase large subunit [Clostridium jeddahense]	99%	0	84.80%	WP_195199241.1
6PT-TERL-50	MZ375091	lizardfe006	SRX11831441	1334	19	terminase large subunit	partial	uncultured Caudovirales phage	terminase [Candidatus Alangreenwoodia gallinarii]	99%	1E-149	76.03%	WP_191390896.1
6PT-TERL-51	MZ375092	lizardfe006	SRX11831441	1330	28	terminase large subunit	partial	uncultured Caudovirales phage	hypothetical protein [Parabacteroides sp. AF17-28]	99%	6E-172	81.63%	WP_122345209.1
6PT-TERL-52	MZ375093	lizardfe006	SRX11831441	1328	11	terminase large subunit	partial	uncultured Caudovirales phage	TPA: terminase [Lachnoclostridium sp.]	99%	2E-168	80.85%	HCD43851.1
6PT-TERL-53	MZ375094	lizardfe006	SRX11831441	1315	64	terminase large subunit	partial	uncultured Caudovirales phage	PBSX family phage terminase large subunit [Parabacteroides provencensis]	100%	0	89.69%	WP_099465169.1
6PT-TERL-55	MZ375095	lizardfe006	SRX11831441	1275	13	terminase large subunit	partial	uncultured Caudovirales phage	terminase large subunit [Clostridium sp. C2-6-12]	97%	0	77.26%	WP_160684363.1
6PT-TERL-56	MZ375096	lizardfe006	SRX11831441	1252	27	terminase large subunit	partial	uncultured Caudovirales phage	PBSX family phage terminase large subunit [Frisingicoccus caecimuris]	99%	8E-175	85.77%	WP_132087437.1
6PT-TERL-57	MZ375097	lizardfe006	SRX11831441	1186	17	terminase large subunit	partial	uncultured Caudovirales phage	PBSX family phage terminase large subunit [Alkalibaculum sporogenes]	96%	0	79.29%	WP_152803953.1
6PT-TERL-59	MZ375098	lizardfe006	SRX11831441	1137	15	terminase large subunit	partial	uncultured Caudovirales phage	hypothetical protein BGO29_14850 [Bacteroidales bacterium 36-12]	99%	4E-170	61.11%	OJV40844.1
6PT-TERL-60	MZ375099	lizardfe006	SRX11831441	1125	23	terminase large subunit	partial	uncultured Caudovirales phage	PBSX family phage terminase large subunit [Anaeromassilibacillus senegalensis]	99%	0	96.39%	WP_082236500.1
6PT-TERL-61	MZ375100	lizardfe006	SRX11831441	1117	35	terminase large subunit	partial	uncultured Caudovirales phage	terminase large subunit [Bacteroides thetaiotaomicron]	99%	0	72.39%	KAB4839554.1
6PT-TERL-62	MZ375101	lizardfe006	SRX11831441	1104	22	terminase large subunit	partial	uncultured Caudovirales phage	terminase large subunit [Alistipes sp.]	99%	0	94.26%	MBQ4533116.1
6PT-TERL-64	MZ375102	lizardfe006	SRX11831441	1099	16	terminase large subunit	partial	uncultured Caudovirales phage	Phage terminase large subunit (GpA) [Eubacteriaceae bacterium CHKCI004]	99%	0	89.62%	CVI68246.1
6PT-TERL-67	MZ375103	lizardfe006	SRX11831441	1052	24	terminase large subunit	partial	uncultured Caudovirales phage	terminase large subunit [Parabacteroides pacaensis]	99%	8E-117	71.56%	WP_106831157.1
6PT-TERL-68	MZ375104	lizardfe006	SRX11831441	1048	13	terminase large subunit	partial	uncultured Caudovirales phage	terminase large subunit [Crassaminicella sp. SY095]	95%	2E-161	73.36%	WP_148809743.1
6PT-TERL-69	MZ375105	lizardfe006	SRX11831441	1041	15	terminase large subunit	partial	uncultured Caudovirales phage	terminase family protein [Bacteroidales bacterium]	77%	2E-94	71.43%	MBR6903426.1
6PT-TERL-70	MZ375106	lizardfe006	SRX11831441	1038	10	terminase large subunit	partial	uncultured Caudovirales phage	phage terminase large subunit [Lawsonibacter sp. NSJ-52]	100%	0	93.06%	WP_186918593.1
6PT-TERL-71	MZ375107	lizardfe006	SRX11831441	1020	7	terminase large subunit	partial	uncultured Caudovirales phage	PBSX family phage terminase large subunit [Faecalibacterium prausnitzii]	95%	0	98.43%	WP_154265275.1
6PT-TERL-72	MZ375108	lizardfe006	SRX11831441	1003	19	terminase large subunit	partial	uncultured Caudovirales phage	TPA: hypothetical protein [Parabacteroides goldsteinii]	99%	0	97.31%	HBA31176.1
6PT-TERL-73	MZ375109	lizardfe006	SRX11831441	1002	22	terminase large subunit	partial	uncultured Caudovirales phage	phage terminase large subunit [Parabacteroides goldsteinii]	97%	0	96.00%	WP_129732321.1
6PT-TERL-74	MZ375110	lizardfe006	SRX11831441	1001	29	terminase large subunit	partial	uncultured Caudovirales phage	terminase [Faecalibacterium prausnitzii]	98%	7E-132	62.50%	WP_097840175.1
6PT-TERL-75	MZ375111	lizardfe006	SRX11831441	931	39	terminase large subunit	partial	uncultured Caudovirales phage	Uncharacterized conserved protein [uncultured Anaerotruncus sp.]	99%	2E-74	74.65%	SCJ35178.1

Table S3. The results of functional annotation of phage-encoded genes based on KEGG database.

Query ORF ID	Subject gene ID	Alignment length	Query start	Query end	Subject start	Subject end	e-value	Identity(%)	KO	Category	Subcategory	Gene name	Description	Pathway	Pathway definition	Abundance (fpkm)					
																(1) PE-NMC	(2) PE-NQ1	(3) PE-NQ2	(4) PT-SZZ	(5) PT-ZB	(6) PT-RT
Lizard01Caudovirales_VIRSorter_scaffold_1025-gene_2|316|1779|1463|+|Phage_cluster_558|321.1|1.2e-97|1|Cpl-	rto:RTO_00860	140	2	138	160	299	1.94E-32	45.714	**K02395**	Cellular Processes	Cell motility	flgJ	flagellar protein FlgJ	—	—	5598.76	408.25	1338.20	0.00	0.00	0.00
Lizard01Caudovirales_VIRSorter_scaffold_1025-gene_4|2174|3541|1367|+|Phage_cluster_118|578.5|8.4e-176|0|-|-|-(+).p1	csb:CLSA_c40930	89	369	455	419	507	1.74E-09	47.191	**K21449**	Brite Hierarchies	Protein families: signaling and cellular processes	ata, sadA, emaA	trimeric autotransporter adhesin	—	—	1345.27	69.57	243.65	0.00	0.00	0.00
Lizard01Caudovirales_VIRSorter_scaffold_9569-gene_7|8082|8561|479|-|-|-|-|-|Pfam-B_10271|43.3|2.7e-13(-).p1	bcel:BcellWH2_04908	140	1	140	34	172	1.90E-63	72.857	**K05989**	Not Included in Pathway or Brite	Unclassified: metabolism	ramA	alpha-L-rhamnosidase [EC:3.2.1.40]	—	—	26.80	32.84	2.20	0.00	0.00	138.37
Lizard01Caudovirales_VIRSorter_scaffold_9043-gene_4|5716|6852|1136|-|-|-|-|-|Mur_ligase_M|136.6|1.3e-41(-).p2	bvu:BVU_0516	377	1	377	56	432	0	85.411	**K01929**	Metabolism	Amino acid metabolism	murF	UDP-N-acetylmuramoyl-tripeptide--D-alanyl-D-alanine ligase [EC:6.3.2.10]	ko00300,ko00550,ko01502	Lysine biosynthesis|Peptidoglycan biosynthesis|Vancomycin resistance	448.49	1367.65	129.27	203.53	160.53	973.30
Lizard01Caudovirales_VIRSorter_scaffold_4612-gene_4|1093|1521|428|-|Phage_cluster_24|204.2|4.1e-	gma:AciX8_1851	140	1	138	4	141	9.76E-37	50	**K01185**	Not Included in Pathway or Brite	Unclassified: metabolism	E3.2.1.17	lysozyme [EC:3.2.1.17]	—	—	495.51	82.76	317.66	0.00	0.00	0.00
Lizard01Caudovirales_VIRSorter_scaffold_684-gene_1|2|886|884|+|gi_66392035_ref_YP_238960.1_|94.0|6.41e-	bxy:BXY_15630	294	1	294	229	522	0	96.939	**K03307**	Not Included in Pathway or Brite	Unclassified: signaling and cellular processes	TC.SSS	solute:Na+ symporter, SSS family	—	—	487435.01	1599973.90	327168.32	3579.62	109482.20	4587783.36
Lizard01Caudovirales_VIRSorter_scaffold_684-gene_2|912|1589|677|+|-|-|-|-|Pfam-B_19239|43.2|3.9e-13(+).p1	bcac:CGC64_10340	225	1	225	1	225	7.98E-157	92	**K03574**	Brite Hierarchies	Protein families: genetic information processing	mutT, NUDT15, MTH2	8-oxo-dGTP diphosphatase [EC:3.6.1.55]	—	—	435.49	1401.53	322.13	0.00	68.60	4316.22
Lizard01Caudovirales_VIRSorter_scaffold_684-gene_3|1633|2316|683|+|-|-|-|-|Aldolase_II|168.6|1.5e-51(+).p1	bcae:A4V03_06530	227	1	227	1	227	2.11E-165	97.797	**K03077**	Metabolism	Carbohydrate metabolism	araD, ulaF, sgaE, sgbE	L-ribulose-5-phosphate 4-epimerase [EC:5.1.3.4]	ko00040,ko00053	Pentose and glucuronate interconversions|Ascorbate and aldarate metabolism	729.64	1183.18	283.49	6.58	51.08	3677.70
Lizard01Caudovirales_VIRSorter_scaffold_684-gene_4|2354|3880|1526|+|-|-|-|-|Arabinose_Isome|547.6|1.6e-166(+).p1	bcac:CGC64_10330	508	1	508	1	508	0	97.244	**K01804**	Metabolism	Carbohydrate metabolism	araA	L-arabinose isomerase [EC:5.3.1.4]	ko00040	Pentose and glucuronate interconversions	1901.95	5179.83	915.21	22.81	270.20	13888.72
Lizard01Caudovirales_VIRSorter_scaffold_3729-gene_5|1136|1624|488|+|Phage_cluster_2634|277.1|5.8e-	bths:CNY62_01420	142	4	145	2	142	3.68E-23	38.732	**K01185**	Not Included in Pathway or Brite	Unclassified: metabolism	E3.2.1.17	lysozyme [EC:3.2.1.17]	—	—	254.27	6.53	15.67	9.45	9.18	1.30
Lizard01Caudovirales_VIRSorter_scaffold_1980-gene_4|2896|3255|359|-|Phage_cluster_9|98.4|4.4e-30|1|SSB|87.4|6.4e-	fgo:C4N16_06380	97	1	97	2	97	1.06E-23	46.392	**K03111**	Genetic Information Processing	Replication and repair	ssb	single-strand DNA-binding protein	ko03030,ko03430,ko03440	DNA replication|Mismatch repair|Homologous recombination	162.08	0.00	6.15	0.00	0.00	0.00
Lizard01Caudovirales_VIRSorter_scaffold_7293-gene_2|559|1524|965|+|-|-|-|-|Na_Ca_ex|204.1|8e-63(+).p1	pdi:BDI_2547	301	17	315	17	314	5.47E-171	81.728	**K07301**	Brite Hierarchies	Protein families: signaling and cellular processes	yrbG	cation:H+ antiporter	—	—	3810.86	19308.63	1847.40	5270.18	4756.45	8052.22
Lizard01Caudovirales_VIRSorter_scaffold_1777-gene_5|1529|2182|653|-|Phage_cluster_164|165.2|2.9e-	cby:CLM_0899	195	4	197	4	197	1.41E-61	51.282	**K07273**	Not Included in Pathway or Brite	Unclassified: signaling and cellular processes	acm	lysozyme	—	—	1818.30	144.50	1356.63	0.00	0.00	0.00
Lizard01Caudovirales_VIRSorter_scaffold_10335-gene_1|1|552|551|+|Phage_cluster_216|128.1|4.4e-	pcat:Pcatena_04740	168	1	166	121	287	4.06E-55	64.286	**K14059**	Not Included in Pathway or Brite	Unclassified: genetic information processing	int	integrase	—	—	433275.56	7414.15	0.00	0.00	0.00	0.00
Lizard01Caudovirales_VIRSorter_scaffold_11736-gene_3|523|921|398|+|Phage_cluster_9|125.9|1.4e-38|1|SSB|84.8|4.2e-	ova:OBV_19910	138	1	132	1	138	7.71E-35	50	**K03111**	Genetic Information Processing	Replication and repair	ssb	single-strand DNA-binding protein	ko03030,ko03430,ko03440	DNA replication|Mismatch repair|Homologous recombination	207.11	19.85	289.38	13.69	0.00	0.00
Lizard01unclassified_bacterial_viruses_VIRSorter_scaffold_18412-gene_3|573|971|398|+|Phage_cluster_10580|73.0|3.9e-	bdo:EL88_12655	111	1	111	1	109	1.45E-20	40.541	**K03111**	Genetic Information Processing	Replication and repair	ssb	single-strand DNA-binding protein	ko03030,ko03430,ko03440	DNA replication|Mismatch repair|Homologous recombination	378.08	2.59	0.00	0.00	0.00	0.00
Lizard01unclassified_bacterial_viruses_VIRSorter_scaffold_18412-gene_6|2574|3407|833|+|Phage_cluster_3199|125.5|4.8e-	bdo:EL88_11445	258	1	254	1	236	8.24E-47	39.535	**K00390**	Metabolism	Energy metabolism	cysH	phosphoadenosine phosphosulfate reductase [EC:1.8.4.8 1.8.4.10]	ko00920	Sulfur metabolism	280.55	1.15	0.00	0.00	0.00	0.00
Lizard01unclassified_bacterial_viruses_VIRSorter_scaffold_11214-gene_1|256|792|536|+|Phage_cluster_10580|83.5|2.2e-	bdo:EL88_12655	106	1	106	1	103	2.35E-21	46.226	**K03111**	Genetic Information Processing	Replication and repair	ssb	single-strand DNA-binding protein	ko03030,ko03430,ko03440	DNA replication|Mismatch repair|Homologous recombination	9026.57	0.00	17.39	0.00	0.00	0.00
Lizard01unclassified_bacterial_viruses_VIRSorter_scaffold_11315-gene_1|76|609|533|+|Phage_cluster_10580|80.6|1.7e-	pseg:D3H65_17955	108	1	108	1	107	5.10E-18	40.741	**K03111**	Genetic Information Processing	Replication and repair	ssb	single-strand DNA-binding protein	ko03030,ko03430,ko03440	DNA replication|Mismatch repair|Homologous recombination	950.17	0.00	0.00	0.00	0.00	0.00
Lizard01unclassified_dsDNA_phages_VIRSorter_scaffold_3158-gene_1|341|2413|2072|+|-|-|-|-|Pfam-B_12464|464.8|4.1e-143(+).p1	pdi:BDI_0607	691	1	690	14	704	0	78.871	**K01843**	Metabolism	Amino acid metabolism	kamA	lysine 2,3-aminomutase [EC:5.4.3.2]	ko00310	Lysine degradation	12176.61	25464.68	3694.16	1753.07	2599.07	5509.97
Lizard01unclassified_dsDNA_phages_VIRSorter_scaffold_3158-gene_3|4255|5085|830|-|Phage_cluster_8887|45.0|2.1e-	buy:D8S85_16620	233	42	271	54	284	1.01E-65	45.064	**K18954**	Brite Hierarchies	Protein families: genetic information processing	pobR	AraC family transcriptional regulator, transcriptional activator of pobA	—	—	653.39	1139.57	189.35	86.65	144.58	256.00
Lizard01unclassified_dsDNA_phages_VIRSorter_scaffold_3158-gene_4|5339|6358|1019|+|-|-|-|-|HlyD|95.1|1.5e-30(+).p1	bvu:BVU_1673	339	1	339	32	370	0	78.171	**K03585**	Human Diseases	Drug resistance: antimicrobial	acrA, mexA, adeI, smeD, mtrC, cmeA	membrane fusion protein, multidrug efflux system	ko01501,ko01503	beta-Lactam resistance|Cationic antimicrobial peptide (CAMP) resistance	811.53	1378.12	319.32	126.05	146.47	247.61
Lizard02Bicaudaviridae_VIRSorter_scaffold_3586-gene_1|1|358|357|-|-|-|-|-|Pfam-B_9784|42.5|3.4e-14(-).p2	pdi:BDI_0616	118	1	118	58	175	3.93E-63	84.746	**K03723**	Genetic Information Processing	Replication and repair	mfd	transcription-repair coupling factor (superfamily II helicase) [EC:3.6.4.-]	ko03420	Nucleotide excision repair	0.00	13345.47	0.00	10738.87	0.00	47230.80
Lizard02Bicaudaviridae_VIRSorter_scaffold_3586-gene_2|765|1502|737|+|Phage_cluster_8323|46.8|1.7e-	pdi:BDI_1302	244	1	244	1	244	1.58E-164	89.344	**K00721**	Metabolism	Glycan biosynthesis and metabolism	DPM1	dolichol-phosphate mannosyltransferase [EC:2.4.1.83]	ko00510	N-Glycan biosynthesis	0.00	63.97	29.09	74.87	27.26	200.01
Lizard02Bicaudaviridae_VIRSorter_scaffold_3586-gene_4|1882|2691|809|+|-|-|-|-|IclR|112.2|9.3e-36(+).p1	parc:CI960_07060	265	1	265	1	265	5.23E-168	85.66	**K19333**	Brite Hierarchies	Protein families: genetic information processing	kdgR	IclR family transcriptional regulator, KDG regulon repressor	—	—	0.00	45.70	4.73	34.24	0.00	78.95
Lizard02Bicaudaviridae_VIRSorter_scaffold_3586-gene_6|4485|5579|1094|+|-|-|-|-|-|-|-|-(+).p1	pdi:BDI_0624	363	1	363	1	363	0	73.003	**K18240**	Metabolism	Amino acid metabolism	xanB2	chorismate lyase / 3-hydroxybenzoate synthase [EC:4.1.3.40 4.1.3.45]	ko00130,ko00400	Ubiquinone and other terpenoid-quinone biosynthesis|Phenylalanine, tyrosine and tryptophan biosynthesis	64.40	108.11	12.90	53.47	18.60	159.94
Lizard02Bicaudaviridae_VIRSorter_scaffold_3586-gene_7|5822|6391|569|+|-|-|-|-|-|-|-|-(+).p1	pdi:BDI_0625	189	1	189	64	252	3.77E-125	88.889	**K09988**	Metabolism	Carbohydrate metabolism	lyxA	D-lyxose ketol-isomerase [EC:5.3.1.15]	ko00040	Pentose and glucuronate interconversions	0.00	34.89	4.59	13.53	3.58	36.00
Lizard02Caudovirales_VIRSorter_scaffold_90144-circular-gene_2|479|943|464|-|Phage_cluster_1201|291.4|2.1e-88|2|-|-|-(-).p1	osp:Odosp_0802	151	3	153	4	154	3.85E-88	79.47	**K01447**	Not Included in Pathway or Brite	Unclassified: metabolism	xlyAB	N-acetylmuramoyl-L-alanine amidase [EC:3.5.1.28]	—	—	0.00	34.05	0.00	7.47	0.00	10.27
Lizard02unclassified_dsDNA_phages_VIRSorter_scaffold_22892-gene_1|1|669|668|+|Phage_cluster_8887|45.7|2.1e-	buy:D8S85_16620	216	6	218	71	284	7.62E-65	46.759	**K18954**	Brite Hierarchies	Protein families: genetic information processing	pobR	AraC family transcriptional regulator, transcriptional activator of pobA	—	—	2419121.90	3960639.75	627629.03	297108.74	573478.20	890497.36
Lizard02unclassified_dsDNA_phages_VIRSorter_scaffold_22892-gene_3|2534|4645|2111|-|-|-|-|-|Pfam-B_12464|472.3|3.5e-144(-).p1	pdi:BDI_0607	703	1	702	2	704	0	78.094	**K01843**	Metabolism	Amino acid metabolism	kamA	lysine 2,3-aminomutase [EC:5.4.3.2]	ko00310	Lysine degradation	1610.11	3459.55	498.88	235.91	349.76	748.85
Lizard02unclassified_dsDNA_phages_VIRSorter_scaffold_22892-gene_4|4967|5365|398|-|Phage_cluster_3079|90.1|5.7e-	pdi:BDI_0606	127	5	131	5	131	9.15E-73	81.89	**K11443**	Environmental Information Processing	Signal transduction	divK	two-component system, cell cycle response regulator DivK	ko02020,ko04112	Two-component system|Cell cycle - Caulobacter	7.27	12.24	1.79	0.72	0.00	3.57
Lizard02unclassified_dsDNA_phages_VIRSorter_scaffold_22892-gene_16|21911|22684|773|-|-|-|-|-|Thi4|265.2|1.8e-81(-).p1	pdi:BDI_1609	253	1	253	2	254	1.00E-167	95.257	**K22699**	Metabolism	Metabolism of cofactors and vitamins	thi4	sulfide-dependent adenosine diphosphate thiazole synthase [EC:2.4.2.59]	ko00730	Thiamine metabolism	4.94	8.53	1.02	0.16	6.66	2.09
Lizard02unclassified_dsDNA_phages_VIRSorter_scaffold_22892-gene_17|22862|25201|2339|-|-|-|-|-|Glyco_hydro_3|112.0|1.4e-34(-).p1	fib:A6C57_10795	748	32	777	24	771	0	64.572	**K05349**	Metabolism	Carbohydrate metabolism	bglX	beta-glucosidase [EC:3.2.1.21]	ko00460,ko00500,ko00940	Cyanoamino acid metabolism|Starch and sucrose metabolism|Phenylpropanoid biosynthesis	18.95	24.45	2.92	0.78	8.21	7.88
Lizard02unclassified_dsDNA_phages_VIRSorter_scaffold_22892-gene_21|28869|29345|476|+|-|-|-|-|-|-|-|-(+).p1	pdi:BDI_1186	155	1	155	1	155	7.35E-81	73.548	**K08968**	Metabolism	Amino acid metabolism	msrC	L-methionine (R)-S-oxide reductase [EC:1.8.4.14]	ko00270	Cysteine and methionine metabolism	0.00	1.23	0.46	0.00	0.00	0.19
Lizard02unclassified_dsDNA_phages_VIRSorter_scaffold_22892-gene_22|29426|34177|4751|-|-|-|-|-|A2M_N_2|72.8|1.2e-22(-).p1	parc:CI960_08365	1582	1	1581	269	1844	0	74.589	**K06894**	Brite Hierarchies	Protein families: metabolism	yfhM	alpha-2-macroglobulin	—	—	12.27	10.88	2.12	1.22	7.09	7.01
Lizard02unclassified_dsDNA_phages_VIRSorter_scaffold_22892-gene_23|34217|34840|623|-|-|-|-|-|-|-|-|-(-).p2	parc:CI960_08365	206	1	206	48	253	1.64E-89	66.505	**K06894**	Brite Hierarchies	Protein families: metabolism	yfhM	alpha-2-macroglobulin	—	—	0.00	0.26	0.00	0.00	0.61	0.35
Lizard02unclassified_dsDNA_phages_VIRSorter_scaffold_49327-gene_2|3262|5223|1961|+|-|-|-|-|SusD|159.6|5.4e-49(+).p1	bfb:VU15_02015	653	1	653	1	653	0	90.046	**K21572**	Brite Hierarchies	Protein families: signaling and cellular processes	susD	starch-binding outer membrane protein, SusD/RagB family	—	—	0.00	238.20	2.73	2.19	51.14	3057.65
Lizard02unclassified_dsDNA_phages_VIRSorter_scaffold_49327-gene_3|6878|7864|986|+|-|-|-|-|HlyD|106.4|8.8e-33(+).p1	bcel:BcellWH2_03475	322	2	323	47	368	0	79.503	**K03585**	Human Diseases	Drug resistance: antimicrobial	acrA, mexA, adeI, smeD, mtrC, cmeA	membrane fusion protein, multidrug efflux system	ko01501,ko01503	beta-Lactam resistance|Cationic antimicrobial peptide (CAMP) resistance	0.00	25.01	0.00	0.00	7.58	417.81
Lizard02unclassified_dsDNA_phages_VIRSorter_scaffold_49327-gene_4|7884|11081|3197|+|-|-|-|-|ACR_tran|981.5|1.9e-297(+).p1	pdi:BDI_0972	1061	1	1060	1	1061	0	83.6	**K03296**	Not Included in Pathway or Brite	Unclassified: signaling and cellular processes	TC.HAE1	hydrophobic/amphiphilic exporter-1 (mainly G- bacteria), HAE1 family	—	—	0.00	139.74	1.13	0.45	66.13	1598.15
Lizard02unclassified_dsDNA_phages_VIRSorter_scaffold_49327-gene_5|11078|12442|1364|+|-|-|-|-|OEP|165.0|7.4e-51(+).p1	bdo:EL88_19915	455	2	454	3	457	0	74.725	**K18139**	Cellular Processes	Cellular community - prokaryotes	oprM, emhC, ttgC, cusC, adeK, smeF, mtrE, cmeC,	outer membrane protein, multidrug efflux system	ko01501,ko02024	beta-Lactam resistance|Quorum sensing	0.00	30.25	0.00	0.00	6.59	332.86
Lizard02unclassified_dsDNA_phages_VIRSorter_scaffold_49327-gene_6|12526|13335|809|-|-|-|-|-|Trp_syntA|257.2|4.4e-79(-).p1	pdi:BDI_0586	256	1	256	2	257	1.62E-156	80.859	**K01695**	Metabolism	Amino acid metabolism	trpA	tryptophan synthase alpha chain [EC:4.2.1.20]	ko00260,ko00400	Glycine, serine and threonine metabolism|Phenylalanine, tyrosine and tryptophan biosynthesis	0.00	8.64	0.00	0.00	0.00	111.31
Lizard02unclassified_dsDNA_phages_VIRSorter_scaffold_49327-gene_7|13360|13953|593|-|-|-|-|-|PRAI|116.1|7.8e-36(-).p1	pdi:BDI_0587	198	1	194	9	206	1.01E-77	58.586	**K01817**	Metabolism	Amino acid metabolism	trpF	phosphoribosylanthranilate isomerase [EC:5.3.1.24]	ko00400	Phenylalanine, tyrosine and tryptophan biosynthesis	0.00	4.22	0.00	0.00	0.78	48.06
Lizard02unclassified_dsDNA_phages_VIRSorter_scaffold_49327-gene_8|13971|14780|809|-|-|-|-|-|IGPS|241.2|4.7e-74(-).p1	pdi:BDI_0588	264	1	264	3	260	3.94E-135	71.591	**K01609**	Metabolism	Amino acid metabolism	trpC	indole-3-glycerol phosphate synthase [EC:4.1.1.48]	ko00400	Phenylalanine, tyrosine and tryptophan biosynthesis	0.00	7.85	0.00	0.00	0.00	77.97
Lizard02unclassified_dsDNA_phages_VIRSorter_scaffold_49327-gene_9|14789|15784|995|-|-|-|-|-|Glycos_transf_3|216.3|2.4e-66(-).p1	parc:CI960_06885	329	1	329	2	330	0	89.666	**K00766**	Metabolism	Amino acid metabolism	trpD	anthranilate phosphoribosyltransferase [EC:2.4.2.18]	ko00400	Phenylalanine, tyrosine and tryptophan biosynthesis	0.00	11.03	0.00	0.24	0.00	117.37
Lizard02unclassified_dsDNA_phages_VIRSorter_scaffold_49327-gene_10|15797|16363|566|-|-|-|-|-|GATase|162.9|3e-50(-).p1	pdi:BDI_0590	186	1	186	2	187	4.20E-121	89.247	**K01658**	Metabolism	Amino acid metabolism	trpG	anthranilate synthase component II [EC:4.1.3.27]	ko00400,ko00405,ko02024,ko02025	Phenylalanine, tyrosine and tryptophan biosynthesis|Phenazine biosynthesis|Quorum sensing|Biofilm formation - Pseudomonas aeruginosa	0.00	2.07	0.00	0.23	0.00	41.76
Lizard02unclassified_dsDNA_phages_VIRSorter_scaffold_49327-gene_11|16412|17815|1403|-|-|-|-|-|Chorismate_bind|256.5|1.3e-78(-).p1	pdi:BDI_0591	465	2	466	3	467	0	86.882	**K01657**	Metabolism	Amino acid metabolism	trpE	anthranilate synthase component I [EC:4.1.3.27]	ko00400,ko00405,ko02024,ko02025	Phenylalanine, tyrosine and tryptophan biosynthesis|Phenazine biosynthesis|Quorum sensing|Biofilm formation - Pseudomonas aeruginosa	0.00	10.66	0.00	0.00	0.00	120.27
Lizard02unclassified_dsDNA_phages_VIRSorter_scaffold_49327-gene_12|17857|19059|1202|-|-|-|-|-|PALP|150.8|2.7e-46(-).p1	parc:CI960_06900	391	1	391	2	392	0	92.072	**K01696**	Metabolism	Amino acid metabolism	trpB	tryptophan synthase beta chain [EC:4.2.1.20]	ko00260,ko00400	Glycine, serine and threonine metabolism|Phenylalanine, tyrosine and tryptophan biosynthesis	0.00	6.39	0.00	0.00	0.00	51.41
Lizard02unclassified_dsDNA_phages_VIRSorter_scaffold_7706-gene_8|11782|12669|887|-|-|-|-|-|Peptidase_S24|48.2|3.3e-15(-).p1	bcel:BcellWH2_04009	290	5	294	8	297	1.06E-151	70.345	**K03100**	Genetic Information Processing	Folding, sorting and degradation	lepB	signal peptidase I [EC:3.4.21.89]	ko03060	Protein export	0.00	12.84	0.38	3.04	0.00	0.63
Lizard02unclassified_dsDNA_phages_VIRSorter_scaffold_9540-gene_1|50|1423|1373|-|-|-|-|-|OEP|174.3|1e-53(-).p1	pdi:BDI_0971	455	1	455	2	454	0	65.714	**K18139**	Cellular Processes	Cellular community - prokaryotes	oprM, emhC, ttgC, cusC, adeK, smeF, mtrE, cmeC,	outer membrane protein, multidrug efflux system	ko01501,ko02024	beta-Lactam resistance|Quorum sensing	19497.40	89503.64	3115.89	12600.27	2502.45	16589.82
Lizard02unclassified_dsDNA_phages_VIRSorter_scaffold_9540-gene_4|4411|5280|869|-|-|-|-|-|HlyD|104.0|4.8e-32(-).p1	bvu:BVU_1673	285	1	285	31	315	1.07E-160	77.544	**K03585**	Human Diseases	Drug resistance: antimicrobial	acrA, mexA, adeI, smeD, mtrC, cmeA	membrane fusion protein, multidrug efflux system	ko01501,ko01503	beta-Lactam resistance|Cationic antimicrobial peptide (CAMP) resistance	0.00	198.67	6.05	3.25	4.73	45.40
Lizard02unclassified_dsDNA_phages_VIRSorter_scaffold_9540-gene_5|5959|6894|935|+|Phage_cluster_8887|49.6|1.3e-	pdi:BDI_0340	281	30	306	21	300	8.05E-57	36.655	**K18954**	Brite Hierarchies	Protein families: genetic information processing	pobR	AraC family transcriptional regulator, transcriptional activator of pobA	—	—	0.00	20.65	1.49	0.00	0.00	7.60
Lizard02unclassified_dsDNA_phages_VIRSorter_scaffold_9540-gene_8|7736|9343|1607|-|-|-|-|-|SusD|188.4|9.3e-58(-).p1	pet:PEIBARAKI_4373	535	1	534	2	536	0	72.523	**K21572**	Brite Hierarchies	Protein families: signaling and cellular processes	susD	starch-binding outer membrane protein, SusD/RagB family	—	—	0.00	29.90	1.15	0.00	0.00	0.00
Lizard02unclassified_dsDNA_phages_VIRSorter_scaffold_3622-gene_1|254|1201|947|-|Phage_cluster_2979|40.3|9.4e-	bdo:EL88_16405	303	10	312	3	295	3.58E-127	58.416	**K01055**	Metabolism	Xenobiotics biodegradation and	pcaD	3-oxoadipate enol-lactonase [EC:3.1.1.24]	ko00362	Benzoate degradation	0.00	449.52	0.00	169.12	0.00	21.31
Lizard02unclassified_dsDNA_phages_VIRSorter_scaffold_3622-gene_6|5420|8320|2900|-|-|-|-|-|ACR_tran|771.7|4.3e-234(-).p1	bfg:BF638R_3999	963	1	963	89	1051	0	95.742	**K03296**	Not Included in Pathway or Brite	Unclassified: signaling and cellular processes	TC.HAE1	hydrophobic/amphiphilic exporter-1 (mainly G- bacteria), HAE1 family	—	—	13.32	311.89	3.29	69.35	7.70	3.90
Lizard02unclassified_dsDNA_phages_VIRSorter_scaffold_3622-gene_8|9347|10234|887|-|-|-|-|-|OEP|145.9|5.4e-45(-).p1	bfs:BF9343_3859	342	1	294	95	436	8.70E-172	72.222	**K12340**	Environmental Information Processing	Membrane transport	tolC	outer membrane protein	ko01501,ko01503,ko02020,ko03070,ko04626,ko05133	beta-Lactam resistance|Cationic antimicrobial peptide (CAMP) resistance|Two-component system|Bacterial secretion system|Plant-pathogen interaction|Pertussis	11.59	40.61	0.95	14.55	0.00	0.84
Lizard02unclassified_dsDNA_phages_VIRSorter_scaffold_22609-gene_3|2343|3092|749|-|-|-|-|-|Steroid_dh|139.0|5.2e-43(-).p1	buy:D8S85_03720	243	8	248	1	243	1.62E-122	69.547	**K12343**	Metabolism	Lipid metabolism	SRD5A1	3-oxo-5-alpha-steroid 4-dehydrogenase 1 [EC:1.3.1.22]	ko00140	Steroid hormone biosynthesis	0.00	144.30	9.50	97.78	8.90	6.72
Lizard02unclassified_dsDNA_phages_VIRSorter_scaffold_53624-gene_1|32|1948|1916|-|-|-|-|-|ACR_tran|551.6|1.3e-167(-).p1	bfs:BF9343_0579	629	1	629	376	1004	0	93.8	**K18138**	Human Diseases	Drug resistance: antimicrobial	acrB, mexB, adeJ, smeE, mtrD, cmeB	multidrug efflux pump	ko01501,ko01503	beta-Lactam resistance|Cationic antimicrobial peptide (CAMP) resistance	2256.64	28173.78	1251.92	2237.26	1303.36	4919.87

Query ORF ID	Subject gene ID	Alignment length	Query start	Query end	Subject start	Subject end	e-value	Identity(%)	KO	Category	Subcategory	Gene name	Description	Pathway	Pathway definition	Abundance (fpkm)					
																(1) PE-NMC	(2) PE-NQ1	(3) PE-NQ2	(4) PT-SZZ	(5) PT-ZB	(6) PT-RT
Lizard02unclassified_dsDNA_phages_VIRSorter_scaffold_53624-gene_2|1963|2964|1001|-|-|-|-|-|ACR_tran|324.0|7.4e-99(-).p2	bcac:CGC64_00380	331	1	331	2	332	0	94.26	**K18138**	Human Diseases	Drug resistance: antimicrobial	acrB, mexB, adeJ, smeE, mtrD, cmeB	multidrug efflux pump	ko01501,ko01503	beta-Lactam resistance|Cationic antimicrobial peptide (CAMP) resistance	91.97	266.65	0.00	21.88	10.62	68.42
Lizard02unclassified_dsDNA_phages_VIRSorter_scaffold_53624-gene_3|2980|3642|662|-|-|-|-|-|HlyD_2|85.6|1.8e-26(-).p2	bcel:BcellWH2_03827	218	1	218	142	359	3.22E-130	83.945	**K03585**	Human Diseases	Drug resistance: antimicrobial	acrA, mexA, adeI, smeD, mtrC, cmeA	membrane fusion protein, multidrug efflux system	ko01501,ko01503	beta-Lactam resistance|Cationic antimicrobial peptide (CAMP) resistance	12.12	99.02	0.00	13.21	0.00	25.26
Lizard02unclassified_dsDNA_phages_VIRSorter_scaffold_3160-gene_2|1071|1958|887|-|Phage_cluster_415|156.8|3.2e-	bacc:BRDCF_p1971	287	4	290	10	296	3.20E-63	36.237	**K04763**	Brite Hierarchies	Protein families: genetic information processing	xerD	integrase/recombinase XerD	—	—	0.00	20.77	0.00	3.34	19.47	6.43
Lizard02UnclassifiedPhage_VIRSorter_scaffold_15748-gene_4|735|3782|3047|+|Phage_cluster_502|537.8|7.6e-163|2|zf-	bfs:BF9343_2238	998	1	998	1	997	0	77.655	**K02316**	Genetic Information Processing	Replication and repair	dnaG	DNA primase [EC:2.7.7.101]	ko03030	DNA replication	0.00	631.46	12.11	0.00	28.37	3.35
Lizard02UnclassifiedPhage_VIRSorter_scaffold_15748-gene_24|17559|18575|1016|+|-|-|-|-|-|-|-|-(+).p1	osp:Odosp_2590	340	1	330	1	328	2.69E-50	31.471	**K07448**	Brite Hierarchies	Protein families: signaling and cellular processes	mrr	restriction system protein	—	—	0.00	6.59	0.51	0.00	0.00	0.00
Lizard02UnclassifiedPhage_VIRSorter_scaffold_15748-gene_36|26455|27408|953|+|Phage_cluster_8936|213.7|9.1e-	lby:Lbys_0129	315	5	317	2	303	9.44E-99	50.794	**K02342**	Genetic Information Processing	Replication and repair	dnaQ	DNA polymerase III subunit epsilon [EC:2.7.7.7]	ko03030,ko03430,ko03440	DNA replication|Mismatch repair|Homologous recombination	0.00	1.63	0.00	0.00	0.00	0.00
Lizard02UnclassifiedPhage_VIRSorter_scaffold_15748-gene_52|42628|43116|488|+|Phage_cluster_91|180.1|1.3e-	bfg:BF638R_3632	164	1	162	1	164	1.30E-79	67.073	**K01447**	Not Included in Pathway or Brite	Unclassified: metabolism	xlyAB	N-acetylmuramoyl-L-alanine amidase [EC:3.5.1.28]	—	—	0.00	1.77	0.00	0.00	0.00	0.00
Lizard02UnclassifiedPhage_VIRSorter_scaffold_15748-gene_53|43229|44392|1163|-|Phage_cluster_10583|314.0|2.4e-	bhl:Bache_2435	368	19	386	21	388	9.88E-117	47.011	**K04763**	Brite Hierarchies	Protein families: genetic information processing	xerD	integrase/recombinase XerD	—	—	0.00	2.71	0.00	0.00	0.00	0.03
Lizard02UnclassifiedPhage_VIRSorter_scaffold_22090-gene_4|3570|4154|584|+|Phage_cluster_158|190.7|7.7e-	bfg:BF638R_3646	197	1	193	3	199	5.87E-98	73.604	**K06904**	Not Included in Pathway or Brite	Poorly characterized	K06904	uncharacterized protein	—	—	0.00	207.26	28.68	1.00	0.00	0.41
Lizard02UnclassifiedPhage_VIRSorter_scaffold_75370-gene_6|4832|5293|461|-|-|-|-|-|Toprim_Crpt|49.5|5.5e-15(-).p1	btho:Btheta7330_01666	152	1	152	479	630	3.30E-87	84.211	**K03169**	Brite Hierarchies	Protein families: genetic information processing	topB	DNA topoisomerase III [EC:5.6.2.1]	—	—	0.00	16.57	0.00	0.00	0.00	10.69
Lizard02UnclassifiedPhage_VIRSorter_scaffold_75370-gene_7|5311|5988|677|-|-|-|-|-|Topoisom_bac|158.1|6.6e-48(-).p1	btho:Btheta7330_01666	214	1	214	247	460	1.11E-133	85.981	**K03169**	Brite Hierarchies	Protein families: genetic information processing	topB	DNA topoisomerase III [EC:5.6.2.1]	—	—	0.00	55.56	0.00	0.00	1.96	33.35
Lizard02UnclassifiedPhage_VIRSorter_scaffold_597-gene_2|550|1095|545|-|Phage_cluster_1764|102.2|6.2e-	parc:CI960_10670	179	1	179	2	180	4.24E-98	73.743	**K10026**	Metabolism	Metabolism of cofactors and vitamins	queE	7-carboxy-7-deazaguanine synthase [EC:4.3.99.3]	ko00790	Folate biosynthesis	131.30	194.12	16.19	0.00	37.92	102.87
Lizard02UnclassifiedPhage_VIRSorter_scaffold_597-gene_4|2031|3275|1244|-|-|-|-|-|Nramp|358.3|8.2e-109(-).p1	parc:CI960_11125	416	1	413	2	417	0	88.942	**K03322**	Brite Hierarchies	Protein families: signaling and cellular processes	mntH	manganese transport protein	—	—	355.55	1313.45	203.82	223.84	231.02	279.79
Lizard02UnclassifiedPhage_VIRSorter_scaffold_597-gene_8|6154|7188|1034|+|-|-|-|-|OEP|130.5|1.2e-39(+).p1	parc:CI960_05235	196	149	344	249	444	1.52E-88	68.878	**K12340**	Environmental Information Processing	Membrane transport	tolC	outer membrane protein	ko01501,ko01503,ko02020,ko03070,ko04626,ko05133	beta-Lactam resistance|Cationic antimicrobial peptide (CAMP) resistance|Two-component system|Bacterial secretion system|Plant-pathogen interaction|Pertussis	457.64	1722.58	315.36	917.30	462.55	102.41
Lizard02UnclassifiedPhage_VIRSorter_scaffold_597-gene_9|7301|8356|1055|+|-|-|-|-|HlyD_2|65.7|8.7e-20(+).p1	pdi:BDI_0347	334	1	334	24	357	0	83.234	**K02005**	Not Included in Pathway or Brite	Unclassified: signaling and cellular processes	ABC.CD.TX	HlyD family secretion protein	—	—	613.23	2198.96	532.86	1161.50	564.12	155.33
Lizard02UnclassifiedPhage_VIRSorter_scaffold_597-gene_10|8361|9191|830|+|-|-|-|-|-|-|-|-(+).p1	psac:PSM36_3322	271	1	268	71	338	3.25E-73	43.542	**K02004**	Brite Hierarchies	Protein families: signaling and cellular processes	ABC.CD.P	putative ABC transport system permease protein	—	—	423.21	1185.24	276.31	831.86	510.06	91.44
Lizard02UnclassifiedPhage_VIRSorter_scaffold_25688-gene_4|3058|3804|746|+|-|-|-|-|ATP_bind_3|68.2|1.5e-20(+).p1	pdi:BDI_2113	248	1	248	1	248	8.21E-159	82.661	**K14058**	Brite Hierarchies	Protein families: genetic information processing	ttcA	tRNA 2-thiocytidine biosynthesis protein TtcA	—	—	0.00	19.40	0.00	0.00	0.00	1.45
Lizard02UnclassifiedPhage_VIRSorter_scaffold_25688-gene_6|4616|5578|962|-|-|-|-|-|IPPT|217.8|2.7e-66(-).p1	pdi:BDI_2111	297	21	317	2	298	0	82.828	**K00791**	Metabolism	Metabolism of terpenoids and polyketides	miaA, TRIT1	tRNA dimethylallyltransferase [EC:2.5.1.75]	ko00908	Zeatin biosynthesis	0.00	47.22	0.00	0.00	0.00	7.14
Lizard02UnclassifiedPhage_VIRSorter_scaffold_25688-gene_8|6417|7202|785|-|Phage_cluster_3074|92.0|6.1e-	pdi:BDI_2106	260	1	260	2	261	6.89E-177	91.538	**K00677**	Metabolism	Glycan biosynthesis and metabolism	lpxA	UDP-N-acetylglucosamine acyltransferase [EC:2.3.1.129]	ko00540,ko01503	Lipopolysaccharide biosynthesis|Cationic antimicrobial peptide (CAMP) resistance	0.00	40.21	0.00	0.00	0.00	8.43
Lizard02UnclassifiedPhage_VIRSorter_scaffold_25688-gene_9|7217|8353|1136|-|-|-|-|-|LpxC|168.9|2.6e-51(-).p1	parc:CI960_16960	377	1	377	85	461	0	92.838	**K16363**	Metabolism	Lipid metabolism	lpxC-fabZ	UDP-3-O-[3-hydroxymyristoyl] N-acetylglucosamine deacetylase / 3-hydroxyacyl-[acyl-carrier-protein] dehydratase [EC:3.5.1.108	ko00061,ko00540	Fatty acid biosynthesis|Lipopolysaccharide biosynthesis	0.00	63.49	0.62	0.99	1.44	12.75
Lizard02UnclassifiedPhage_VIRSorter_scaffold_8558-gene_2|305|625|320|-|-|-|-|-|DUF486|103.1|1.8e-31(-).p1	bfs:BF9343_1089	105	1	105	18	122	3.29E-68	90.476	**K09922**	Not Included in Pathway or Brite	Poorly characterized	K09922	uncharacterized protein	—	—	0.00	175.02	0.00	0.00	0.00	40.33
Lizard02UnclassifiedPhage_VIRSorter_scaffold_8558-gene_3|703|1452|749|-|-|-|-|-|ATP_bind_3|59.0|9.8e-18(-).p1	bcae:A4V03_04245	247	1	247	2	248	1.77E-166	87.045	**K14058**	Brite Hierarchies	Protein families: genetic information processing	ttcA	tRNA 2-thiocytidine biosynthesis protein TtcA	—	—	0.00	375.45	0.00	25.46	103.82	139.97
Lizard02UnclassifiedPhage_VIRSorter_scaffold_8558-gene_7|4595|5473|878|+|-|-|-|-|PNP_UDP_1|151.7|3.5e-46(+).p1	bfr:BF1175	292	1	292	1	292	0	92.808	**K00757**	Metabolism	Nucleotide metabolism	udp, UPP	uridine phosphorylase [EC:2.4.2.3]	ko00240,ko00983	Pyrimidine metabolism|Drug metabolism - other enzymes	0.00	43.57	0.00	3.12	0.00	11.03
Lizard02UnclassifiedPhage_VIRSorter_scaffold_8558-gene_8|5473|6000|527|+|Phage_cluster_8936|119.5|4.6e-	bfg:BF638R_1188	175	1	175	1	175	1.00E-111	85.143	**K02342**	Genetic Information Processing	Replication and repair	dnaQ	DNA polymerase III subunit epsilon [EC:2.7.7.7]	ko03030,ko03430,ko03440	DNA replication|Mismatch repair|Homologous recombination	0.00	24.11	0.00	1.31	0.00	3.06
Lizard02UnclassifiedPhage_VIRSorter_scaffold_8558-gene_9|5984|6634|650|-|-|-|-|-|NAD_binding_10|113.1|3.5e-34(-).p1	bfs:BF9343_1082	215	1	215	2	216	1.16E-135	86.977	**K07118**	Not Included in Pathway or Brite	Poorly characterized	K07118	uncharacterized protein	—	—	0.00	17.35	0.00	1.79	0.00	1.81
Lizard02UnclassifiedPhage_VIRSorter_scaffold_8558-gene_10|6786|7778|992|+|-|-|-|-|TrmE_N|132.7|1.2e-40(+).p1	bfb:VU15_05095	330	1	330	1	330	0	89.697	**K03650**	Brite Hierarchies	Protein families: genetic information processing	mnmE, trmE, MSS1	tRNA modification GTPase [EC:3.6.-.-]	—	—	0.00	29.06	0.00	2.11	1.54	3.34
Lizard02UnclassifiedPhage_VIRSorter_scaffold_33086-gene_1|1|613|612|-|-|-|-|-|ATP_bind_3|68.0|1.6e-20(-).p1	bcel:BcellWH2_04611	203	1	203	2	204	2.78E-136	87.192	**K14058**	Brite Hierarchies	Protein families: genetic information processing	ttcA	tRNA 2-thiocytidine biosynthesis protein TtcA	—	—	0.00	51899.06	0.00	28636.99	0.00	3443.91
Lizard02UnclassifiedPhage_VIRSorter_scaffold_33086-gene_5|3851|4729|878|+|-|-|-|-|PNP_UDP_1|150.6|7.8e-46(+).p1	bhl:Bache_1651	292	1	292	1	292	0	93.151	**K00757**	Metabolism	Nucleotide metabolism	udp, UPP	uridine phosphorylase [EC:2.4.2.3]	ko00240,ko00983	Pyrimidine metabolism|Drug metabolism - other enzymes	0.00	27.72	0.00	8.37	0.00	0.64
Lizard02UnclassifiedPhage_VIRSorter_scaffold_33086-gene_6|4733|6114|1381|+|-|-|-|-|TrmE_N|130.0|8.8e-40(+).p1	bhl:Bache_1650	460	1	460	1	460	0	85.435	**K03650**	Brite Hierarchies	Protein families: genetic information processing	mnmE, trmE, MSS1	tRNA modification GTPase [EC:3.6.-.-]	—	—	22.89	37.60	0.00	11.34	0.00	1.66
Lizard02UnclassifiedPhage_VIRSorter_scaffold_1367-gene_2|849|1640|791|-|-|-|-|-|BPD_transp_1|66.7|4.3e-20(-).p1	bcel:BcellWH2_05262	262	1	262	2	263	7.39E-165	88.168	**K11070**	Environmental Information Processing	Membrane transport	potC	spermidine/putrescine transport system permease protein	ko02010	ABC transporters	42.53	193.87	0.00	21.08	0.00	43.46
Lizard02UnclassifiedPhage_VIRSorter_scaffold_1367-gene_3|1637|2437|800|-|-|-|-|-|BPD_transp_1|61.3|1.9e-18(-).p1	bcel:BcellWH2_05263	265	1	265	2	266	4.97E-167	86.792	**K11071**	Environmental Information Processing	Membrane transport	potB	spermidine/putrescine transport system permease protein	ko02010	ABC transporters	0.00	66.12	5.44	28.43	0.00	18.03
Lizard02UnclassifiedPhage_VIRSorter_scaffold_1367-gene_4|2449|3831|1382|-|Phage_cluster_405|181.7|5e-	pdi:BDI_1648	458	1	458	4	461	0	90.611	**K11072**	Environmental Information Processing	Membrane transport	potA	spermidine/putrescine transport system ATP-binding protein [EC:7.6.2.11]	ko02010	ABC transporters	0.00	66.00	0.00	19.00	0.00	14.87
Lizard02UnclassifiedPhage_VIRSorter_scaffold_4349-gene_4|1354|2886|1532|+|Phage_cluster_75|245.6|1.7e-	ain:Acin_1351	509	1	462	48	499	5.88E-76	35.56	**K00558**	Metabolism	Amino acid metabolism	DNMT1, dcm	DNA (cytosine-5)-methyltransferase 1 [EC:2.1.1.37]	ko00270,ko05206	Cysteine and methionine metabolism|MicroRNAs in cancer	26.67	48.19	6.57	0.00	0.00	0.00
Lizard02UnclassifiedPhage_VIRSorter_scaffold_15587-gene_4|2357|3751|1394|-|Phage_cluster_126|421.9|5.9e-	ccel:CCDG5_1548	431	5	428	18	431	2.41E-105	42.691	**K06909**	Not Included in Pathway or Brite	Unclassified: genetic information processing	xtmB	phage terminase large subunit	—	—	107.23	37.75	100.09	4.56	0.00	0.00
Lizard02UnclassifiedPhage_VIRSorter_scaffold_15587-gene_5|3930|4304|374|-|Phage_cluster_180|75.3|9.9e-23|1|-|-|-(-).p1	sje:AAV35_012750	106	2	105	4	109	4.43E-22	45.283	**K07474**	Not Included in Pathway or Brite	Unclassified: genetic information processing	xtmA	phage terminase small subunit	—	—	9.19	1.13	1.13	0.00	0.00	0.00
Lizard02UnclassifiedPhage_VIRSorter_scaffold_21501-gene_2|312|2363|2051|+|Phage_cluster_2488|59.7|3.3e-18|2|E1-	btho:Btheta7330_04373	642	3	633	6	647	0	64.798	**K01534**	Not Included in Pathway or Brite	Unclassified: metabolism	zntA	Zn2+/Cd2+-exporting ATPase [EC:7.2.2.12 7.2.2.21]	—	—	0.00	408.73	0.00	126.20	0.00	29.96
Lizard02UnclassifiedPhage_VIRSorter_scaffold_21501-gene_3|2275|2634|359|+|-|-|-|-|-|-|-|-(+).p1	buy:D8S85_21020	118	1	118	20	137	2.20E-62	77.966	**K03711**	Brite Hierarchies	Protein families: genetic information processing	fur, zur, furB	Fur family transcriptional regulator, ferric uptake regulator	—	—	0.00	7.82	0.00	0.00	0.00	0.22
Lizard02UnclassifiedPhage_VIRSorter_scaffold_21501-gene_4|2631|3839|1208|-|Phage_cluster_1231|149.5|2.2e-	bcac:CGC64_04645	401	1	401	3	403	0	89.526	**K11717**	Metabolism	Metabolism of other amino acids	sufS	cysteine desulfurase / selenocysteine lyase [EC:2.8.1.7 4.4.1.16]	ko00450	Selenocompound metabolism	0.00	25.36	0.00	13.61	0.00	0.93
Lizard02UnclassifiedPhage_VIRSorter_scaffold_28571-gene_2|363|1463|1100|+|-|-|-|-|AAA_14|98.3|7.1e-30(+).p1	dun:FDZ78_03870	365	2	366	53	417	8.59E-157	59.726	**K07133**	Not Included in Pathway or Brite	Poorly characterized	K07133	uncharacterized protein	—	—	0.00	142.97	12.26	0.00	0.00	0.00
Lizard02UnclassifiedPhage_VIRSorter_scaffold_28571-gene_3|1716|2819|1103|+|Phage_cluster_1231|137.8|8.3e-	pdi:BDI_3543	367	1	367	38	404	0	90.191	**K11717**	Metabolism	Metabolism of other amino acids	sufS	cysteine desulfurase / selenocysteine lyase [EC:2.8.1.7 4.4.1.16]	ko00450	Selenocompound metabolism	0.00	37.16	5.19	0.00	0.00	0.00
Lizard02UnclassifiedPhage_VIRSorter_scaffold_28571-gene_4|3417|3956|539|+|-|-|-|-|RNase_HII|147.2|1e-44(+).p1	pdi:BDI_3545	179	1	179	1	179	7.23E-113	87.151	**K03470**	Genetic Information Processing	Replication and repair	rnhB	ribonuclease HII [EC:3.1.26.4]	ko03030	DNA replication	0.00	2.60	0.00	0.00	0.00	0.00
Lizard02UnclassifiedPhage_VIRSorter_scaffold_21370-gene_1|2|508|506|+|-|-|-|-|-|-|-|-(+).p1	bdh:GV66_02155	170	1	168	467	636	1.81E-82	71.176	**K16092**	Brite Hierarchies	Protein families: signaling and cellular processes	btuB	vitamin B12 transporter	—	—	0.00	8896.98	0.00	32216.61	20853.75	0.00
Lizard02UnclassifiedPhage_VIRSorter_scaffold_21370-gene_4|2108|3834|1726|+|-|-|-|-|Pfam-B_18073|66.9|3.8e-20(+).p1	buy:D8S85_15565	274	302	575	20	293	1.99E-91	54.745	**K07648**	Environmental Information Processing	Signal transduction	arcB	two-component system, OmpR family, aerobic respiration control sensor histidine kinase ArcB [EC:2.7.13.3]	ko02020,ko02026	Two-component system|Biofilm formation - Escherichia coli	17.13	37.28	0.00	152.83	34.62	81.92
Lizard02UnclassifiedPhage_VIRSorter_scaffold_60490-gene_2|243|1058|815|-|-|-|-|-|BPD_transp_1|44.0|3.9e-13(-).p1	rbp:B6259_08235	270	1	270	2	271	2.35E-135	68.889	**K11071**	Environmental Information Processing	Membrane transport	potB	spermidine/putrescine transport system permease protein	ko02010	ABC transporters	0.00	36.61	0.00	0.00	0.00	0.00
Lizard02UnclassifiedPhage_VIRSorter_scaffold_60490-gene_3|1055|2185|1130|-|Phage_cluster_405|163.6|1.4e-	ruk:A4V00_14110	361	4	364	7	367	0	67.867	**K11072**	Environmental Information Processing	Membrane transport	potA	spermidine/putrescine transport system ATP-binding protein [EC:7.6.2.11]	ko02010	ABC transporters	0.00	30.92	0.00	0.00	0.00	0.00
Lizard02UnclassifiedPhage_VIRSorter_scaffold_48025-gene_3|1007|1981|974|-|Phage_cluster_371|327.8|2.9e-	det:DET1070	268	6	227	223	490	8.63E-77	49.254	**K07273**	Not Included in Pathway or Brite	Unclassified: signaling and cellular processes	acm	lysozyme	—	—	143.42	32.40	8.84	7.11	0.00	0.00
Lizard02UnclassifiedPhage_VIRSorter_scaffold_14900-gene_2|465|830|365|+|Phage_cluster_10580|71.2|1.4e-21|2|SSB|50.7|3e-	ash:AL1_01660	111	2	112	1	109	1.59E-25	43.243	**K03111**	Genetic Information Processing	Replication and repair	ssb	single-strand DNA-binding protein	ko03030,ko03430,ko03440	DNA replication|Mismatch repair|Homologous recombination	0.00	25.51	0.00	0.00	0.00	0.00
Lizard02UnclassifiedPhage_VIRSorter_scaffold_65039-gene_2|412|777|365|+|Phage_cluster_10580|63.8|2.7e-	ash:AL1_01660	109	1	109	1	107	1.61E-22	40.367	**K03111**	Genetic Information Processing	Replication and repair	ssb	single-strand DNA-binding protein	ko03030,ko03430,ko03440	DNA replication|Mismatch repair|Homologous recombination	87.64	71.98	97.24	0.00	0.00	0.00
Lizard02UnclassifiedPhage_VIRSorter_scaffold_27787-gene_1|109|642|533|+|Phage_cluster_10580|86.3|3e-26|2|SSB|48.2|1.8e-	bdo:EL88_12655	109	1	109	1	106	6.73E-23	42.202	**K03111**	Genetic Information Processing	Replication and repair	ssb	single-strand DNA-binding protein	ko03030,ko03430,ko03440	DNA replication|Mismatch repair|Homologous recombination	0.00	149.64	40.84	0.00	0.00	0.00
Lizard02UnclassifiedPhage_VIRSorter_scaffold_4179-gene_2|675|1436|761|-|Phage_cluster_8304|129.1|3.8e-39|1|-|-|-(-).p1	buy:D8S85_04025	250	2	251	523	772	1.18E-108	66.8	**K03546**	Brite Hierarchies	Protein families: genetic information processing	sbcC, rad50	DNA repair protein SbcC/Rad50	—	—	0.00	19.77	0.00	0.00	0.00	0.00
Lizard02UnclassifiedPhage_VIRSorter_scaffold_4179-gene_3|1454|2164|710|-|-|-|-|-|-|-|-|-(-).p2	buy:D8S85_04025	197	1	189	229	417	1.27E-20	34.518	**K03546**	Brite Hierarchies	Protein families: genetic information processing	sbcC, rad50	DNA repair protein SbcC/Rad50	—	—	24.83	4.08	0.00	0.00	0.00	0.00
Lizard02UnclassifiedPhage_VIRSorter_scaffold_52255-gene_3|1590|1988|398|-|Phage_cluster_10580|73.6|2.6e-	bdo:EL88_12655	110	1	110	2	109	1.36E-18	39.091	**K03111**	Genetic Information Processing	Replication and repair	ssb	single-strand DNA-binding protein	ko03030,ko03430,ko03440	DNA replication|Mismatch repair|Homologous recombination	136.25	1.87	0.00	0.00	0.00	0.00
Lizard02UnclassifiedPhage_VIRSorter_scaffold_30965-gene_1|3|707|704|+|-|-|-|-|AMP-binding|121.4|7.2e-37(+).p1	aqd:D1816_06095	211	7	217	166	376	5.42E-77	53.555	**K03367**	Metabolism	Metabolism of other amino acids	dltA	D-alanine--poly(phosphoribitol) ligase subunit 1 [EC:6.1.1.13]	ko00473,ko01503,ko02020,ko05150	D-Alanine metabolism|Cationic antimicrobial peptide (CAMP) resistance|Two-component system|Staphylococcus aureus infection	48141.73	3954.21	1483.76	0.00	0.00	164.00
Lizard02UnclassifiedPhage_VIRSorter_scaffold_30965-gene_2|679|1122|443|-|Phage_cluster_176|113.9|1.8e-34|1|rve|55.1|1.7e-	pje:CRM71_08850	146	1	146	169	312	5.98E-52	61.644	**K07482**	Not Included in Pathway or Brite	Unclassified: genetic information processing	K07482	transposase, IS30 family	—	—	106.35	2.18	6.56	0.00	0.00	5.80
Lizard02UnclassifiedPhage_VIRSorter_scaffold_27630-gene_1|1|762|761|-|Phage_cluster_438|206.9|9.5e-63|1|CbiA|67.2|2.9e-	bprl:CL2_26050	253	3	250	10	249	1.03E-54	38.34	**K03496**	Brite Hierarchies	Protein families: genetic information processing	parA, soj	chromosome partitioning protein	—	—	0.00	8896.98	0.00	0.00	0.00	0.00
Lizard02UnclassifiedPhage_VIRSorter_scaffold_24285-gene_1|1|483|482|+|Phage_cluster_1197|114.9|8.6e-35|2|-|-|-(+).p1	ele:Elen_2643	150	1	150	237	373	1.15E-20	35.333	**K00986**	Not Included in Pathway or Brite	Unclassified: metabolism	ltrA	RNA-directed DNA polymerase [EC:2.7.7.49]	—	—	0.00	14828.30	0.00	0.00	0.00	0.00
Lizard02UnclassifiedPhage_VIRSorter_scaffold_35134-gene_1|1|335|334|-|Phage_cluster_91|129.8|2.9e-	boa:Bovatus_03282	110	1	110	2	111	6.22E-54	72.727	**K01447**	Not Included in Pathway or Brite	Unclassified: metabolism	xlyAB	N-acetylmuramoyl-L-alanine amidase [EC:3.5.1.28]	—	—	0.00	25208.12	0.00	0.00	0.00	0.00
Lizard02UnclassifiedPhage_VIRSorter_scaffold_18928-gene_1|102|929|827|+|-|-|-|-|Inositol_P|181.4|4.8e-55(+).p1	psac:PSM36_1321	257	15	270	1	257	2.55E-80	47.082	**K01092**	Metabolism	Carbohydrate metabolism	E3.1.3.25, IMPA, suhB	myo-inositol-1(or 4)-monophosphatase [EC:3.1.3.25]	ko00521,ko00562,ko04070	Streptomycin biosynthesis|Inositol phosphate metabolism|Phosphatidylinositol signaling system	0.00	101.76	0.00	0.00	0.00	9.65

Query ORF ID	Subject gene ID	Alignment length	Query start	Query end	Subject start	Subject end	e-value	Identity(%)	KO	Category	Subcategory	Gene name	Description	Pathway	Pathway definition	Abundance (fpkm)					
																(1) PE-NMC	(2) PE-NQ1	(3) PE-NQ2	(4) PT-SZZ	(5) PT-ZB	(6) PT-RT
Lizard02UnclassifiedPhage_VIRSorter_scaffold_21847-gene_1|127|429|302|-|-|-|-|-|Plasmid_stabil|50.8|3.8e-15(-).p1	ddl:Desdi_1423	100	1	99	3	102	4.75E-41	67	**K19092**	Brite Hierarchies	Protein families: signaling and cellular processes	parE1_3_4	toxin ParE1/3/4	—	—	0.00	23.35	0.00	0.00	0.00	0.00
Lizard03Inoviridae_VIRSorter_scaffold_22911-gene_3|651|1022|371|+|-|-|-|-|Zot|62.1|6.5e-20(+).p1	byl:A4V09_21350	122	3	123	55	175	9.39E-36	48.361	**K10954**	Human Diseases	Infectious disease: bacterial	zot	zona occludens toxin	ko05110	Vibrio cholerae infection	0.00	0.00	41.03	0.00	0.00	0.00
Lizard03unclassified_bacterial_viruses_VIRSorter_scaffold_2122-gene_16|15266|15850|584|-|Phage_cluster_158|190.7|3.7e-	bfg:BF638R_3646	196	1	192	4	199	6.69E-97	73.469	**K06904**	Not Included in Pathway or Brite	Poorly characterized	K06904	uncharacterized protein	—	—	0.00	48.28	6.71	0.23	0.00	0.10
Lizard03unclassified_bacterial_viruses_VIRSorter_scaffold_24962-gene_7|4769|6337|1568|+|Phage_cluster_152|296.7|2.5e-	myr:MYRA21_0097	327	102	420	2	314	5.03E-71	41.284	**K00986**	Not Included in Pathway or Brite	Unclassified: metabolism	ltrA	RNA-directed DNA polymerase [EC:2.7.7.49]	—	—	0.00	0.00	101.74	0.00	10.93	16.61
Lizard03unclassified_bacterial_viruses_VIRSorter_scaffold_12487-gene_9|5003|5500|497|+|Phage_cluster_10580|90.9|5.3e-	bdo:EL88_12655	108	1	108	1	105	5.34E-26	48.148	**K03111**	Genetic Information Processing	Replication and repair	ssb	single-strand DNA-binding protein	ko03030,ko03430,ko03440	DNA replication|Mismatch repair|Homologous recombination	0.00	0.59	205.53	0.00	0.00	0.00
Lizard03unclassified_bacterial_viruses_VIRSorter_scaffold_13641-gene_5|1996|2322|326|-|Phage_cluster_171|47.8|9.1e-15|1|-|-|-(-).p1	sbo:SBO_1935	93	7	93	2	80	1.80E-07	38.71	**K00571**	Brite Hierarchies	Protein families: signaling and cellular processes	E2.1.1.72	site-specific DNA-methyltransferase (adenine-specific) [EC:2.1.1.72]	—	—	0.00	0.00	2.23	0.00	0.00	0.49
Lizard03unclassified_bacterial_viruses_VIRSorter_scaffold_13641-gene_10|4429|5400|971|-|Phage_cluster_925|518.5|8.1e-	erb:A4V01_13080	323	1	321	2	324	0	75.851	**K00558**	Metabolism	Amino acid metabolism	DNMT1, dcm	DNA (cytosine-5)-methyltransferase 1 [EC:2.1.1.37]	ko00270,ko05206	Cysteine and methionine metabolism|MicroRNAs in cancer	0.00	0.00	17.09	0.00	0.00	0.00
Lizard03unclassified_bacterial_viruses_VIRSorter_scaffold_10569-gene_7|5117|5491|374|-|Phage_cluster_10580|65.2|4.9e-	ash:AL1_01660	117	3	119	1	114	2.04E-22	41.026	**K03111**	Genetic Information Processing	Replication and repair	ssb	single-strand DNA-binding protein	ko03030,ko03430,ko03440	DNA replication|Mismatch repair|Homologous recombination	7.06	4.06	11.31	0.00	0.00	0.00
Lizard03unclassified_bacterial_viruses_VIRSorter_scaffold_2812-gene_4|1076|1375|299|+|Phage_cluster_438|128.0|5.4e-	bhan:CGC63_03940	99	1	99	1	99	3.08E-57	85.859	**K03496**	Brite Hierarchies	Protein families: genetic information processing	parA, soj	chromosome partitioning protein	—	—	0.00	0.00	16.55	0.00	0.00	0.00
Lizard03unclassified_bacterial_viruses_VIRSorter_scaffold_2812-gene_5|1415|1879|464|+|Phage_cluster_438|145.9|1.8e-	bhan:CGC63_03940	133	22	154	130	262	3.99E-77	84.962	**K03496**	Brite Hierarchies	Protein families: genetic information processing	parA, soj	chromosome partitioning protein	—	—	0.00	0.00	22.02	0.00	0.00	0.00
Lizard03unclassified_bacterial_viruses_VIRSorter_scaffold_2812-gene_6|1885|3096|1211|+|Phage_cluster_682|214.1|2.8e-65|1|-|-|-(+).p1	bhan:CGC63_03950	246	4	248	2	244	3.10E-138	83.333	**K03497**	Brite Hierarchies	Protein families: genetic information processing	parB, spo0J	ParB family transcriptional regulator, chromosome partitioning protein	—	—	0.00	1.57	170.02	0.00	0.00	0.00
Lizard03unclassified_bacterial_viruses_VIRSorter_scaffold_2179-gene_1|1|327|326|+|-|-|-|-|-|-|-|-(+).p1	bcac:CGC64_10315	108	1	108	562	669	1.41E-64	95.37	**K00615**	Metabolism	Carbohydrate metabolism	E2.2.1.1, tktA, tktB	transketolase [EC:2.2.1.1]	ko00030,ko00710,ko01051	Pentose phosphate pathway|Carbon fixation in photosynthetic organisms|Biosynthesis of ansamycins	288850.38	732518.17	160245.71	0.00	52134.38	1440539.38
Lizard03unclassified_bacterial_viruses_VIRSorter_scaffold_2179-gene_2|327|761|434|+|-|-|-|-|-|LacAB_rpiB|174.4|1e-53(+).p1	bth:BT_0346	144	1	144	1	144	3.41E-95	90.972	**K01808**	Metabolism	Carbohydrate metabolism	rpiB	ribose 5-phosphate isomerase B [EC:5.3.1.6]	ko00030,ko00051,ko00710	Pentose phosphate pathway|Fructose and mannose metabolism|Carbon fixation in photosynthetic organisms	662.50	2639.17	381.15	0.00	191.32	5736.84
Lizard03unclassified_bacterial_viruses_VIRSorter_scaffold_3300-gene_5|1648|3195|1547|-|-|-|-|-|DUF87|92.3|4.3e-28(-).p1	byl:A4V09_00630	514	1	514	2	515	0	88.132	**K06915**	Not Included in Pathway or Brite	Poorly characterized	K06915	uncharacterized protein	—	—	87.64	26.09	316.02	30.41	0.00	0.60
Lizard03unclassified_bacterial_viruses_VIRSorter_scaffold_6045-gene_2|1364|1786|422|+|Phage_cluster_10580|74.5|6.5e-	ark:D6B99_12705	106	3	108	1	106	1.44E-21	40.566	**K03111**	Genetic Information Processing	Replication and repair	ssb	single-strand DNA-binding protein	ko03030,ko03430,ko03440	DNA replication|Mismatch repair|Homologous recombination	0.00	0.00	39.16	0.00	0.00	0.00
Lizard03unclassified_bacterial_viruses_VIRSorter_scaffold_1258-gene_1|240|758|518|+|Phage_cluster_10580|90.2|8.8e-	ash:AL1_01660	105	1	105	1	102	2.75E-23	43.81	**K03111**	Genetic Information Processing	Replication and repair	ssb	single-strand DNA-binding protein	ko03030,ko03430,ko03440	DNA replication|Mismatch repair|Homologous recombination	5115.06	6.18	3190.08	0.00	0.00	0.00
Lizard03unclassified_bacterial_viruses_VIRSorter_scaffold_20874-gene_3|1043|1678|635|-|Phage_cluster_2094|147.4|4.2e-	lps:LPST_C2031	144	3	146	69	209	4.14E-28	42.361	**K07455**	Brite Hierarchies	Protein families: genetic information processing	recT	recombination protein RecT	—	—	69.24	125.11	55.48	0.00	0.00	0.00
Lizard04Caudovirales_VIRSorter_scaffold_1125-gene_2|742|2277|1535|-|Phage_cluster_5430|59.2|1.9e-17|1|-|-|-(-).p1	adg:Adeg_1750	165	2	163	7	167	2.87E-09	29.697	**K03546**	Brite Hierarchies	Protein families: genetic information processing	sbcC, rad50	DNA repair protein SbcC/Rad50	—	—	0.00	0.00	0.00	1162.65	0.00	0.00
Lizard04Caudovirales_VIRSorter_scaffold_1125-gene_3|2277|3230|953|-|Phage_cluster_359|133.5|5.7e-	ctm:Cabther_A0040	265	39	298	56	306	9.12E-23	31.321	**K03553**	Genetic Information Processing	Replication and repair	recA	recombination protein RecA	ko03440	Homologous recombination	0.00	0.00	0.00	256.25	18.32	0.00
Lizard04Caudovirales_VIRSorter_scaffold_1125-gene_10|8406|9020|614|-|-|-|-|-|-|-|-|-(-).p1	asan:AWM72_08675	179	15	180	9	162	1.63E-19	35.754	**K00759**	Metabolism	Nucleotide metabolism	APRT, apt	adenine phosphoribosyltransferase [EC:2.4.2.7]	ko00230	Purine metabolism	0.00	0.00	0.00	37.47	0.00	0.00
Lizard04Caudovirales_VIRSorter_scaffold_1125-gene_11|9007|9612|605|-|-|-|-|-|PNP_UDP_1|63.1|1.3e-18(-).p1	sscu:CEP64_05255	210	1	172	2	209	1.88E-06	26.19	**K01243**	Metabolism	Amino acid metabolism	mtnN, mtn, pfs	adenosylhomocysteine nucleosidase [EC:3.2.2.9]	ko00270	Cysteine and methionine metabolism	0.00	0.00	0.00	44.51	0.00	0.00
Lizard04Caudovirales_VIRSorter_scaffold_1125-gene_12|9609|10091|482|-|-|-|-|-|LuxS|78.7|2.4e-23(-).p1	fpa:FPR_21730	125	8	131	5	121	2.02E-16	37.6	**K07173**	Metabolism	Amino acid metabolism	luxS	S-ribosylhomocysteine lyase [EC:4.4.1.21]	ko00270,ko02024,ko02026,ko05111	Cysteine and methionine metabolism|Quorum sensing|Biofilm formation - Escherichia coli|Biofilm formation - Vibrio cholerae	0.00	0.00	0.00	27.19	0.00	0.00
Lizard04Caudovirales_VIRSorter_scaffold_1125-gene_13|10102|11013|911|-|-|-|-|-|-|-|-|-(-).p1	cpor:BED41_05010	294	2	272	25	301	3.76E-22	28.912	**K03698**	Brite Hierarchies	Protein families: genetic information processing	cbf, cbf1	3'-5' exoribonuclease [EC:3.1.-.-]	—	—	0.00	0.00	0.00	74.41	1.03	0.00
Lizard04Caudovirales_VIRSorter_scaffold_1125-gene_14|11094|11621|527|-|-|-|-|-|Ferritin|78.7|2.4e-23(-).p1	tam:Theam_0658	158	13	170	7	159	5.02E-23	35.443	**K03594**	Metabolism	Metabolism of cofactors and vitamins	bfr	bacterioferritin [EC:1.16.3.1]	ko00860	Porphyrin and chlorophyll metabolism	0.00	0.00	0.00	9.36	0.00	0.00
Lizard04Caudovirales_VIRSorter_scaffold_1125-gene_25|25020|26186|1166|-|Phage_cluster_4339|221.8|8.5e-67|2|S-	plv:ERIC2_c00860	402	1	385	7	390	5.83E-82	39.801	**K00789**	Metabolism	Amino acid metabolism	metK	S-adenosylmethionine synthetase [EC:2.5.1.6]	ko00270	Cysteine and methionine metabolism	0.00	0.00	0.00	49.36	0.00	0.00
Lizard04Caudovirales_VIRSorter_scaffold_1125-gene_47|42524|43480|956|-|Phage_cluster_465|52.0|2.3e-15|1|-|-|-(-).p1	mmas:MYMAC_003054	261	87	313	660	908	9.82E-08	26.054	**K02335**	Genetic Information Processing	Replication and repair	polA	DNA polymerase I [EC:2.7.7.7]	ko03030,ko03410,ko03420,ko03440	DNA replication|Base excision repair|Nucleotide excision repair|Homologous recombination	0.00	0.00	0.00	31.82	0.00	0.00
Lizard04Caudovirales_VIRSorter_scaffold_1125-gene_52|47066|49753|2687|-|Phage_cluster_205|208.1|1.3e-	pul:NT08PM_0156	550	32	559	356	854	1.32E-39	25.818	**K02335**	Genetic Information Processing	Replication and repair	polA	DNA polymerase I [EC:2.7.7.7]	ko03030,ko03410,ko03420,ko03440	DNA replication|Base excision repair|Nucleotide excision repair|Homologous recombination	0.00	0.00	0.00	51.57	0.00	0.00
Lizard04Caudovirales_VIRSorter_scaffold_1125-gene_68|65369|66865|1496|-|Phage_cluster_1627|73.9|4.8e-	vpa:VP1385	84	218	301	199	281	8.91E-21	55.952	**K19304**	Brite Hierarchies	Protein families: metabolism	mepM	murein DD-endopeptidase [EC:3.4.24.-]	—	—	0.00	0.00	0.00	20.64	0.32	0.00
Lizard04Caudovirales_VIRSorter_scaffold_1125-gene_86|83375|86104|2729|-|Phage_cluster_3071|120.8|4.2e-	cch:Cag_0934	178	625	791	52	229	6.35E-29	40.449	**K02343**	Genetic Information Processing	Replication and repair	dnaX	DNA polymerase III subunit gamma/tau [EC:2.7.7.7]	ko03030,ko03430,ko03440	DNA replication|Mismatch repair|Homologous recombination	0.00	0.00	0.00	30.05	0.13	0.00
Lizard04Caudovirales_VIRSorter_scaffold_1125-gene_88|86836|91860|5024|-|-|-|-|-|LAGLIDADG_3|60.3|1.1e-17(-).p1	tig:THII_2912	471	615	1035	262	716	1.12E-18	23.355	**K00789**	Metabolism	Amino acid metabolism	metK	S-adenosylmethionine synthetase [EC:2.5.1.6]	ko00270	Cysteine and methionine metabolism	0.00	0.00	0.00	75.23	0.48	0.00
Lizard04Caudovirales_VIRSorter_scaffold_16159-gene_12|4715|6742|2027|+|Phage_cluster_197|768.9|2.3e-232|1|Pfam-	cbol:CGC65_27805	671	6	675	2	661	0	73.323	**K02334**	Not Included in Pathway or Brite	Unclassified: metabolism	dpo	DNA polymerase bacteriophage-type [EC:2.7.7.7]	—	—	0.00	3.14	0.00	97.18	0.00	3.13
Lizard04Caudovirales_VIRSorter_scaffold_16159-gene_13|6751|9186|2435|+|Phage_cluster_204|757.0|9.5e-	cbol:CGC65_27800	804	1	799	1	804	0	72.264	**K06919**	Not Included in Pathway or Brite	Unclassified: genetic information processing	K06919	putative DNA primase/helicase	—	—	0.00	5.05	0.00	127.26	0.00	6.27
Lizard04Caudovirales_VIRSorter_scaffold_16159-gene_21|14225|15529|1304|+|Phage_cluster_1610|390.1|7.7e-	ruk:A4V00_13210	418	1	418	32	448	0	65.55	**K06909**	Not Included in Pathway or Brite	Unclassified: genetic information processing	xtmB	phage terminase large subunit	—	—	10.15	14.80	5.32	50.33	2.93	0.03
Lizard04Caudovirales_VIRSorter_scaffold_26040-gene_1|197|1195|998|-|-|-|-|-|DUF377|241.1|8.1e-73(-).p2	pdi:BDI_0634	331	1	331	2	332	0	88.822	**K18785**	Not Included in Pathway or Brite	Unclassified: metabolism	mp2	beta-1,4-mannooligosaccharide/beta-1,4-mannosyl-N-acetylglucosamine phosphorylase [EC:2.4.1.319 2.4.1.320]	—	—	733.12	4373.22	542.29	1798.90	211.71	5961.29
Lizard04Caudovirales_VIRSorter_scaffold_26040-gene_2|1319|3184|1865|-|gi_66392035_ref_YP_238960.1_|55.5|1.96e-	bvu:BVU_0580	614	1	612	34	627	0	66.775	**K03307**	Not Included in Pathway or Brite	Unclassified: signaling and cellular processes	TC.SSS	solute:Na+ symporter, SSS family	—	—	492.73	2128.13	283.48	987.86	221.34	2235.39
Lizard04Caudovirales_VIRSorter_scaffold_26040-gene_5|4636|5508|872|-|-|-|-|-|DeoC|51.9|4.4e-15(-).p1	pdi:BDI_0631	289	1	289	2	290	0	91.349	**K08321**	Cellular Processes	Cellular community - prokaryotes	lsrF	3-hydroxy-5-phosphonooxypentane-2,4-dione thiolase [EC:2.3.1.245]	ko02024	Quorum sensing	46.73	510.80	46.09	135.12	58.48	376.10
Lizard04Caudovirales_VIRSorter_scaffold_26040-gene_6|5834|6448|614|-|-|-|-|-|Dak2|140.8|2.4e-42(-).p1	pdi:BDI_0630	203	1	203	2	204	3.73E-114	80.296	**K05879**	Metabolism	Lipid metabolism	dhaL	phosphoenolpyruvate---glycerone phosphotransferase subunit DhaL [EC:2.7.1.121]	ko00561	Glycerolipid metabolism	111.40	315.17	35.10	62.59	23.23	156.52
Lizard04Caudovirales_VIRSorter_scaffold_26040-gene_7|6458|7474|1016|-|-|-|-|-|Dak1|226.6|2.1e-68(-).p1	parc:CI960_07095	337	1	337	2	338	0	88.131	**K05878**	Metabolism	Lipid metabolism	dhaK	phosphoenolpyruvate---glycerone phosphotransferase subunit DhaK [EC:2.7.1.121]	ko00561	Glycerolipid metabolism	218.05	793.77	96.50	161.85	83.96	332.38
Lizard04Caudovirales_VIRSorter_scaffold_26040-gene_11|14820|15389|569|-|-|-|-|-|-|-|-|-(-).p1	pdi:BDI_0625	187	2	188	66	252	1.91E-124	89.305	**K09988**	Metabolism	Carbohydrate metabolism	lyxA	D-lyxose ketol-isomerase [EC:5.3.1.15]	ko00040	Pentose and glucuronate interconversions	0.00	12.81	1.80	5.31	1.41	14.04
Lizard04Caudovirales_VIRSorter_scaffold_26040-gene_12|15632|16726|1094|-|-|-|-|-|-|-|-|-(-).p1	pdi:BDI_0624	362	1	362	2	363	0	72.652	**K18240**	Metabolism	Amino acid metabolism	xanB2	chorismate lyase / 3-hydroxybenzoate synthase [EC:4.1.3.40 4.1.3.45]	ko00130,ko00400	Ubiquinone and other terpenoid-quinone biosynthesis|Phenylalanine, tyrosine and tryptophan biosynthesis	18.48	30.92	3.70	15.34	6.00	45.70
Lizard04Caudovirales_VIRSorter_scaffold_26040-gene_14|18520|19305|785|-|-|-|-|-|IclR|112.3|8.8e-34(-).p1	parc:CI960_07060	256	1	256	10	265	6.29E-165	87.5	**K19333**	Brite Hierarchies	Protein families: genetic information processing	kdgR	IclR family transcriptional regulator, KDG regulon repressor	—	—	0.00	4.56	0.48	3.48	0.00	7.94
Lizard04Caudovirales_VIRSorter_scaffold_11156-gene_5|6132|6899|767|+|Phage_cluster_3510|91.2|3.9e-	mbu:Mbur_1605	253	1	253	1	253	6.19E-117	63.241	**K00978**	Metabolism	Carbohydrate metabolism	rfbF	glucose-1-phosphate cytidylyltransferase [EC:2.7.7.33]	ko00500,ko00520	Starch and sucrose metabolism|Amino sugar and nucleotide sugar metabolism	0.00	0.00	0.00	59.54	1.70	25.43
Lizard04Caudovirales_VIRSorter_scaffold_11156-gene_6|6900|7727|827|-|-|-|-|-|LicD|147.5|3.7e-44(-).p1	boa:Bovatus_03501	268	1	268	2	269	6.78E-138	65.672	**K07271**	Not Included in Pathway or Brite	Unclassified: metabolism	licD	lipopolysaccharide cholinephosphotransferase [EC:2.7.8.-]	—	—	0.00	0.00	0.00	88.71	27.20	30.87
Lizard04Caudovirales_VIRSorter_scaffold_11156-gene_7|7852|8946|1094|+|Phage_cluster_398|134.6|2.3e-	tar:TALC_00085	351	6	356	6	356	0	67.806	**K08678**	Metabolism	Carbohydrate metabolism	UXS1, uxs	UDP-glucuronate decarboxylase [EC:4.1.1.35]	ko00520	Amino sugar and nucleotide sugar metabolism	0.00	0.00	0.00	138.13	35.85	42.04
Lizard04Caudovirales_VIRSorter_scaffold_11156-gene_8|8940|10199|1259|+|Phage_cluster_3799|141.9|1.3e-	eyy:EGYY_16600	423	1	419	1	418	5.67E-176	59.338	**K00012**	Metabolism	Carbohydrate metabolism	UGDH, ugd	UDPglucose 6-dehydrogenase [EC:1.1.1.22]	ko00040,ko00053,ko00520	Pentose and glucuronate interconversions|Ascorbate and aldarate metabolism|Amino sugar and nucleotide sugar metabolism	0.00	0.00	0.00	393.60	83.97	113.37
Lizard04Caudovirales_VIRSorter_scaffold_11156-gene_10|11025|11711|686|-|-|-|-|-|Peptidase_M22|96.3|1.5e-28(-).p1	osp:Odosp_1555	227	1	227	2	228	8.90E-133	76.652	**K14742**	Brite Hierarchies	Protein families: genetic information processing	tsaB	tRNA threonylcarbamoyladenosine biosynthesis protein TsaB	—	—	0.00	0.00	0.00	143.83	30.26	45.74
Lizard04Caudovirales_VIRSorter_scaffold_11156-gene_11|11729|12340|611|-|-|-|-|-|Sua5_yciO_yrdC|155.1|7.8e-47(-).p2	osp:Odosp_1554	190	13	202	1	190	8.54E-118	86.316	**K07566**	Brite Hierarchies	Protein families: genetic information processing	tsaC, rimN, SUA5	L-threonylcarbamoyladenylate synthase [EC:2.7.7.87]	—	—	0.00	0.00	0.00	112.62	24.00	43.41
Lizard04Caudovirales_VIRSorter_scaffold_11156-gene_13|13328|14224|896|-|-|-|-|-|-|-|-|-(-).p1	osp:Odosp_1546	297	1	297	2	298	0	85.859	**K03340**	Metabolism	Amino acid metabolism	dapdh	diaminopimelate dehydrogenase [EC:1.4.1.16]	ko00300	Lysine biosynthesis	0.00	0.00	0.00	154.43	54.76	104.36
Lizard04Caudovirales_VIRSorter_scaffold_11156-gene_14|14255|15073|818|-|-|-|-|-|THDPS_N_2|77.0|5.5e-23(-).p1	osp:Odosp_1545	271	1	271	2	272	6.67E-168	83.026	**K00674**	Metabolism	Amino acid metabolism	dapD	2,3,4,5-tetrahydropyridine-2,6-dicarboxylate N-succinyltransferase [EC:2.3.1.117]	ko00300	Lysine biosynthesis	0.00	0.00	0.00	117.77	56.32	82.28
Lizard04Caudovirales_VIRSorter_scaffold_11156-gene_16|16569|17135|566|+|-|-|-|-|Adenine_glyco|260.5|4.1e-79(+).p1	bdo:EL88_23710	188	1	188	1	188	1.88E-128	90.426	**K01246**	Genetic Information Processing	Replication and repair	tag	DNA-3-methyladenine glycosylase I [EC:3.2.2.20]	ko03410	Base excision repair	2.18	0.72	0.00	50.77	15.10	20.31
Lizard04Caudovirales_VIRSorter_scaffold_21291-gene_2|668|1003|335|+|-|-|-|-|RBFA|94.3|2.9e-28(+).p1	osp:Odosp_2634	111	1	111	1	111	5.05E-65	84.685	**K02834**	Brite Hierarchies	Protein families: genetic information processing	rbfA	ribosome-binding factor A	—	—	0.00	0.00	0.00	182.20	15.61	238.63
Lizard04Caudovirales_VIRSorter_scaffold_21291-gene_3|1003|2205|1202|+|-|-|-|-|MacB_PCD|63.3|1.7e-18(+).p1	osp:Odosp_2635	400	1	400	1	400	0	67.75	**K09808**	Environmental Information Processing	Membrane transport	lolC_E	lipoprotein-releasing system permease protein	ko02010	ABC transporters	0.00	0.00	0.00	1445.41	259.89	1357.75
Lizard04Caudovirales_VIRSorter_scaffold_21291-gene_6|3531|4550|1019|+|-|-|-|-|tRNA-synt_2d|324.4|2.5e-98(+).p1	osp:Odosp_2638	339	1	339	1	339	0	91.445	**K01889**	Genetic Information Processing	Translation	FARSA, pheS	phenylalanyl-tRNA synthetase alpha chain [EC:6.1.1.20]	ko00970	Aminoacyl-tRNA biosynthesis	0.00	0.00	0.00	516.01	118.12	377.32
Lizard04Caudovirales_VIRSorter_scaffold_21291-gene_8|5542|6144|602|+|-|-|-|-|DUF3897|175.4|6.1e-53(+).p1	osp:Odosp_2641	200	1	200	1	200	4.34E-132	87.5	**K07337**	Not Included in Pathway or Brite	Unclassified: signaling and cellular processes	K07337	penicillin-binding protein activator	—	—	0.00	0.00	0.00	173.10	30.10	131.65
Lizard04Caudovirales_VIRSorter_scaffold_21291-gene_9|6240|7529|1289|+|-|-|-|-|Pfam-B_10531|40.4|1.6e-11(+).p1	osp:Odosp_2642	430	1	429	32	461	0	77.674	**K09859**	Not Included in Pathway or Brite	Poorly characterized	K09859	uncharacterized protein	—	—	0.00	0.00	1.43	615.53	205.53	474.72
Lizard04Caudovirales_VIRSorter_scaffold_21291-gene_12|10295|11233|938|+|Phage_cluster_352|139.4|7.7e-	osp:Odosp_2644	312	1	312	1	312	0	93.59	**K00948**	Metabolism	Carbohydrate metabolism	PRPS, prsA	ribose-phosphate pyrophosphokinase [EC:2.7.6.1]	ko00030,ko00230	Pentose phosphate pathway|Purine metabolism	7.01	0.00	0.00	90.06	20.26	73.50
Lizard04Caudovirales_VIRSorter_scaffold_21291-gene_15|12690|14066|1376|-	osp:Odosp_2647	457	1	457	20	476	0	77.024	**K03307**	Not Included in Pathway or Brite	Unclassified: signaling and cellular processes	TC.SSS	solute:Na+ symporter, SSS family	—	—	0.00	0.00	0.00	133.42	18.90	95.41
Lizard04Caudovirales_VIRSorter_scaffold_21291-gene_16|14288|17056|2768|+|-|-|-|-|tRNA-synt_1_2|216.8|9.9e-66(+).p1	osp:Odosp_2649	922	1	922	1	922	0	90.456	**K01869**	Genetic Information Processing	Translation	LARS, leuS	leucyl-tRNA synthetase [EC:6.1.1.4]	ko00970	Aminoacyl-tRNA biosynthesis	0.00	0.10	0.00	287.61	40.87	342.82

Query ORF ID	Subject gene ID	Alignment length	Query start	Query end	Subject start	Subject end	e-value	Identity(%)	KO	Category	Subcategory	Gene name	Description	Pathway	Pathway definition	Abundance (fpkm)					
																(1) PE-NMC	(2) PE-NQ1	(3) PE-NQ2	(4) PT-SZZ	(5) PT-ZB	(6) PT-RT
Lizard04Caudovirales_VIRSorter_scaffold_8420-gene_3|4563|5699|1136|-|-|-|-|-|Mur_ligase_M|136.0|1.1e-40(-).p2	bdo:EL88_21955	377	1	377	56	432	0	85.146	**K01929**	Metabolism	Amino acid metabolism	murF	UDP-N-acetylmuramoyl-tripeptide--D-alanyl-D-alanine ligase [EC:6.3.2.10]	ko00300,ko00550,ko01502	Lysine biosynthesis|Peptidoglycan biosynthesis|Vancomycin resistance	561.81	1641.74	151.20	264.37	207.94	1224.31
Lizard04Caudovirales_VIRSorter_scaffold_8420-gene_4|6303|7160|857|+|-|-|-|-|Pterin_bind|226.2|1.9e-68(+).p1	bvu:BVU_0517	285	1	285	1	285	0	85.965	**K00796**	Metabolism	Metabolism of cofactors and vitamins	folP	dihydropteroate synthase [EC:2.5.1.15]	ko00790	Folate biosynthesis	189.04	369.59	34.60	113.02	33.09	314.80
Lizard04Caudovirales_VIRSorter_scaffold_8420-gene_5|7225|7989|764|+|-|-|-|-|DisA_N|153.1|1.2e-46(+).p1	bvu:BVU_0518	254	1	254	1	254	5.33E-178	96.457	**K18672**	Not Included in Pathway or Brite	Unclassified: metabolism	dacA	diadenylate cyclase [EC:2.7.7.85]	—	—	59.97	167.27	33.89	27.25	15.87	140.89
Lizard04Caudovirales_VIRSorter_scaffold_8420-gene_7|9444|10346|902|-|-|-|-|-|DUF2156|189.5|4e-57(-).p1	bvu:BVU_0520	299	1	299	2	300	0	92.308	**K01163**	Not Included in Pathway or Brite	Poorly characterized	K01163	uncharacterized protein	—	—	7.65	65.00	12.73	30.32	15.46	63.40
Lizard04Caudovirales_VIRSorter_scaffold_8420-gene_9|11728|12636|908|+|-|-|-|-|PTA_PTB|346.9|6.7e-105(+).p1	bvu:BVU_0523	302	1	302	36	337	0	96.689	**K00625**	Metabolism	Carbohydrate metabolism	E2.3.1.8, pta	phosphate acetyltransferase [EC:2.3.1.8]	ko00430,ko00620,ko00640,ko00680,ko00720	Taurine and hypotaurine metabolism|Pyruvate metabolism|Propanoate metabolism|Methane metabolism|Carbon fixation pathways in prokaryotes	18.47	22.25	5.31	15.57	5.33	35.74
Lizard04Caudovirales_VIRSorter_scaffold_8420-gene_10|12652|13848|1196|+|-|-|-|-|Acetate_kinase|535.7|3.2e-162(+).p1	bvu:BVU_0524	398	1	398	1	398	0	95.226	**K00925**	Metabolism	Carbohydrate metabolism	ackA	acetate kinase [EC:2.7.2.1]	ko00430,ko00620,ko00640,ko00680,ko00720	Taurine and hypotaurine metabolism|Pyruvate metabolism|Propanoate metabolism|Methane metabolism|Carbon fixation pathways in prokaryotes	11.42	25.32	5.63	12.73	7.42	35.58
Lizard04Caudovirales_VIRSorter_scaffold_8420-gene_11|14554|15240|686|-|-|-|-|-|-|-|-|-(-).p1	bvu:BVU_0525	227	1	227	29	255	1.32E-159	93.833	**K03269**	Metabolism	Glycan biosynthesis and metabolism	lpxH	UDP-2,3-diacylglucosamine hydrolase [EC:3.6.1.54]	ko00540	Lipopolysaccharide biosynthesis	12.40	5.60	0.00	4.67	0.00	11.02
Lizard04Caudovirales_VIRSorter_scaffold_2072-gene_3|2659|3366|707|-|-|-|-|-|FtsX|50.6|1e-14(-).p1	sli:Slin_2084	215	1	213	299	513	4.10E-34	33.023	**K02004**	Brite Hierarchies	Protein families: signaling and cellular processes	ABC.CD.P	putative ABC transport system permease protein	—	—	0.00	0.56	0.00	21.54	3.92	0.00
Lizard04Caudovirales_VIRSorter_scaffold_2072-gene_5|3891|5057|1166|+|Phage_cluster_3804|44.1|7.3e-	pdi:BDI_0460	379	1	379	48	426	0	78.364	**K03327**	Brite Hierarchies	Protein families: signaling and cellular processes	TC.MATE, SLC47A, norM, mdtK, dinF	multidrug resistance protein, MATE family	—	—	0.00	25.91	2.29	35.88	26.80	44.76
Lizard04Caudovirales_VIRSorter_scaffold_2072-gene_6|5505|6662|1157|-|-|-|-|-|AcylCoA_dehyd_C|175.1|2.4e-53(-).p1	bcel:BcellWH2_01353	377	8	384	192	568	0	89.39	**K20035**	Metabolism	Energy metabolism	dmdC	3-(methylthio)propanoyl-CoA dehydrogenase [EC:1.3.8.-]	ko00920	Sulfur metabolism	13.12	51.99	3.23	81.28	32.20	74.00
Lizard04Caudovirales_VIRSorter_scaffold_2072-gene_7|7064|8083|1019|-|-|-|-|-|ETF|129.9|5.2e-39(-).p1	pdi:BDI_0462	338	1	338	2	339	0	93.491	**K03522**	Brite Hierarchies	Protein families: signaling and cellular processes	fixB, etfA	electron transfer flavoprotein alpha subunit	—	—	15.33	68.64	11.97	49.66	13.28	60.66
Lizard04Caudovirales_VIRSorter_scaffold_2072-gene_8|8089|8997|908|-|-|-|-|-|ETF|136.4|5.2e-41(-).p1	pdi:BDI_0463	301	1	301	1	301	0	87.708	**K03521**	Not Included in Pathway or Brite	Unclassified: metabolism	fixA, etfB	electron transfer flavoprotein beta subunit	—	—	17.85	45.65	8.80	41.16	11.60	41.36
Lizard04Caudovirales_VIRSorter_scaffold_2072-gene_10|9681|10148|467|-|-|-|-|-|HTH_18|59.0|2.6e-17(-).p1	parc:CI960_12690	155	1	152	2	156	9.38E-72	68.387	**K18954**	Brite Hierarchies	Protein families: genetic information processing	pobR	AraC family transcriptional regulator, transcriptional activator of pobA	—	—	0.00	18.99	2.76	6.29	6.46	10.98
Lizard04Caudovirales_VIRSorter_scaffold_2072-gene_12|11671|12498|827|-|Phage_cluster_15279|298.4|5e-90|1|Pfam-	parc:CI960_12675	274	1	274	677	950	1.03E-177	94.161	**K00281**	Metabolism	Carbohydrate metabolism	GLDC, gcvP	glycine dehydrogenase [EC:1.4.4.2]	ko00260,ko00630	Glycine, serine and threonine metabolism|Glyoxylate and dicarboxylate metabolism	15.47	71.15	11.44	29.14	13.40	51.30
Lizard04Caudovirales_VIRSorter_scaffold_2072-gene_13|12554|13945|1391|-|Phage_cluster_15279|372.9|1.5e-	pdi:BDI_0467	453	1	453	140	592	0	86.093	**K00281**	Metabolism	Carbohydrate metabolism	GLDC, gcvP	glycine dehydrogenase [EC:1.4.4.2]	ko00260,ko00630	Glycine, serine and threonine metabolism|Glyoxylate and dicarboxylate metabolism	8.63	51.26	8.86	42.20	10.80	54.63
Lizard04Caudovirales_VIRSorter_scaffold_2072-gene_14|14237|14875|638|-|-|-|-|-|Lactamase_B|97.4|5.7e-29(-).p1	pdi:BDI_0468	211	1	211	2	212	1.57E-123	77.251	**K01069**	Metabolism	Carbohydrate metabolism	gloB, gloC, HAGH	hydroxyacylglutathione hydrolase [EC:3.1.2.6]	ko00620	Pyruvate metabolism	0.00	2.40	0.94	6.03	0.00	3.52
Lizard04Caudovirales_VIRSorter_scaffold_2072-gene_15|14872|15345|473|-|-|-|-|-|GidB|93.6|5.7e-28(-).p1	parc:CI960_12665	156	1	156	65	220	1.20E-90	82.051	**K03501**	Brite Hierarchies	Protein families: genetic information processing	gidB, rsmG	16S rRNA (guanine527-N7)-methyltransferase [EC:2.1.1.170]	—	—	0.00	1.20	0.30	4.81	0.70	2.61
Lizard04Caudovirales_VIRSorter_scaffold_9008-gene_5|2358|2777|419|+|Phage_cluster_460|150.2|3.5e-	anx:ACH33_08635	139	3	108	4	142	3.82E-29	43.885	**K07474**	Not Included in Pathway or Brite	Unclassified: genetic information processing	xtmA	phage terminase small subunit	—	—	0.00	0.00	0.00	56.17	0.00	0.00
Lizard04Caudovirales_VIRSorter_scaffold_9008-gene_7|3995|5416|1421|+|Phage_cluster_767|500.1|3.5e-	bprm:CL3_06830	451	10	457	21	471	0	57.206	**K09961**	Not Included in Pathway or Brite	Poorly characterized	K09961	uncharacterized protein	—	—	0.00	0.74	0.00	200.71	0.00	0.12
Lizard04Caudovirales_VIRSorter_scaffold_9008-gene_11|7369|8550|1181|+|Phage_cluster_1403|274.5|9.8e-	bprm:CL3_06810	231	1	222	1	224	2.55E-65	52.814	**K09960**	Not Included in Pathway or Brite	Poorly characterized	K09960	uncharacterized protein	—	—	0.00	0.00	0.00	79.18	0.00	0.27
Lizard04Caudovirales_VIRSorter_scaffold_3938-gene_26|11982|12587|605|-|Phage_cluster_579|179.8|3.5e-	bhan:CGC63_04110	167	1	152	39	199	1.33E-34	44.91	**K01447**	Not Included in Pathway or Brite	Unclassified: metabolism	xlyAB	N-acetylmuramoyl-L-alanine amidase [EC:3.5.1.28]	—	—	0.00	0.00	0.00	39.73	0.00	0.04
Lizard04Caudovirales_VIRSorter_scaffold_2214-gene_7|6118|7266|1148|+|Phage_cluster_396|357.5|3.2e-	bcae:A4V03_05100	417	1	379	1	411	2.64E-127	51.799	**K00558**	Metabolism	Amino acid metabolism	DNMT1, dcm	DNA (cytosine-5)-methyltransferase 1 [EC:2.1.1.37]	ko00270,ko05206	Cysteine and methionine metabolism|MicroRNAs in cancer	0.00	0.00	0.00	154.47	0.00	0.00
Lizard04Caudovirales_VIRSorter_scaffold_1436-gene_12|5559|7685|2126|-|Phage_cluster_10908|780.0|1.2e-	cmiu:B1H56_09665	306	376	680	56	357	6.23E-69	38.889	**K17680**	Human Diseases	Neurodegenerative disease	PEO1	twinkle protein [EC:3.6.4.12]	ko05017	Spinocerebellar ataxia	0.00	39.74	3.20	112.69	510.18	529.16
Lizard04Caudovirales_VIRSorter_scaffold_13693-gene_3|896|1417|521|-|Phage_cluster_1430|190.5|1e-	cpro:CPRO_13950	169	4	172	4	172	2.67E-41	37.87	**K06908**	Not Included in Pathway or Brite	Poorly characterized	K06908	uncharacterized protein	—	—	0.00	9.93	0.00	71.91	11.64	0.00
Lizard04Caudovirales_VIRSorter_scaffold_13693-gene_4|1421|2869|1448|-|Phage_cluster_740|547.2|1.3e-165|2|-|-|-(-).p1	cpro:CPRO_13940	485	1	481	2	482	0	59.588	**K06907**	Not Included in Pathway or Brite	Poorly characterized	K06907	uncharacterized protein	—	—	0.00	39.65	0.00	176.34	22.01	0.35
Lizard04Caudovirales_VIRSorter_scaffold_10142-gene_1|1|894|893|-|-|-|-|Calx-beta|49.6|2e-14(-).p2	ndo:DDD_2081	277	19	294	5932	6190	5.68E-11	25.993	**K20276**	Cellular Processes	Cellular community - prokaryotes	bapA	large repetitive protein	ko02024	Quorum sensing	0.00	0.00	0.00	214777.40	396221.30	432457.01
Lizard04Caudovirales_VIRSorter_scaffold_10142-gene_3|1963|4656|2693|-|Phage_cluster_10584|49.7|1.6e-	osp:Odosp_2397	907	4	895	42	892	1.55E-158	36.935	**K14645**	Cellular Processes	Cellular community - prokaryotes	K14645	serine protease [EC:3.4.21.-]	ko02024	Quorum sensing	0.00	0.00	0.00	311.83	207.16	667.68
Lizard04Caudovirales_VIRSorter_scaffold_10142-gene_5|6418|8334|1916|-|-|-|-|-|BNR_2|85.3|3.1e-25(-).p2	phe:Phep_2216	675	12	634	33	652	2.07E-56	28.741	**K01186**	Metabolism	Lipid metabolism	NEU1	sialidase-1 [EC:3.2.1.18]	ko00511,ko00600,ko04142	Other glycan degradation|Sphingolipid metabolism|Lysosome	0.00	0.00	0.00	47.41	29.24	93.91
Lizard04Caudovirales_VIRSorter_scaffold_10142-gene_6|8364|9854|1490|-|-|-|-|-|SusD-like_3|70.5|1.3e-20(-).p1	phe:Phep_2217	503	2	495	27	519	3.89E-112	37.773	**K21572**	Brite Hierarchies	Protein families: signaling and cellular processes	susD	starch-binding outer membrane protein, SusD/RagB family	—	—	0.00	0.00	0.00	19.26	6.23	46.06
Lizard04Caudovirales_VIRSorter_scaffold_2307-gene_1|74|754|680|+|Phage_cluster_4904|142.9|5.7e-	osp:Odosp_0573	226	1	226	1	226	9.85E-141	84.071	**K09125**	Brite Hierarchies	Protein families: signaling and cellular processes	K09125	uncharacterized protein	—	—	0.00	0.00	0.00	241.87	281.81	46.54
Lizard04Caudovirales_VIRSorter_scaffold_2307-gene_2|845|1273|428|+|-|-|-|-|-|-|-|-(+).p1	bfs:BF9343_3049	95	1	95	1	95	1.27E-27	52.632	**K23775**	Brite Hierarchies	Protein families: genetic information processing	ohrR	MarR family transcriptional regulator, organic hydroperoxide resistance regulator	—	—	0.00	0.00	0.00	21.18	0.00	0.58
Lizard04Caudovirales_VIRSorter_scaffold_2307-gene_4|2439|3506|1067|+|-|-|-|-|HlyD|104.9|3.5e-31(+).p1	pdi:BDI_0683	346	7	352	8	353	4.35E-170	66.763	**K03543**	Brite Hierarchies	Protein families: signaling and cellular processes	emrA	membrane fusion protein, multidrug efflux system	—	—	0.00	1.22	0.00	51.37	8.55	4.84
Lizard04Caudovirales_VIRSorter_scaffold_18406-gene_9|6052|6609|557|-|Phage_cluster_158|214.7|1.1e-	plw:D5F53_29240	183	1	181	9	190	4.30E-73	58.47	**K06904**	Not Included in Pathway or Brite	Poorly characterized	K06904	uncharacterized protein	—	—	0.00	0.00	0.00	10.65	0.00	0.00
Lizard04Caudovirales_VIRSorter_scaffold_13017-gene_5|3402|5075|1673|-|Phage_cluster_11567|297.7|1.1e-	spun:BFF78_20295	554	15	542	19	555	1.11E-106	38.628	**K06400**	Not Included in Pathway or Brite	Unclassified: signaling and cellular processes	spoIVCA	site-specific DNA recombinase	—	—	0.00	0.00	0.00	70.50	0.00	0.00
Lizard04Caudovirales_VIRSorter_scaffold_467-gene_1|26|340|314|-|Phage_cluster_3100|88.8|1.3e-26|1|DPBB_1|48.1|6.2e-14(-).p2	pdi:BDI_1049	101	2	102	9	109	2.19E-46	72.277	**K03642**	Not Included in Pathway or Brite	Unclassified: signaling and cellular processes	rlpA	rare lipoprotein A	—	—	0.00	570.32	342.41	413.03	0.00	264.92
Lizard04Caudovirales_VIRSorter_scaffold_467-gene_2|366|1715|1349|-|-|-|-|-|CCP_MauG|171.4|1.3e-51(-).p1	pdi:BDI_1048	450	1	448	10	459	0	77.333	**K00428**	Not Included in Pathway or Brite	Unclassified: metabolism	E1.11.1.5	cytochrome c peroxidase [EC:1.11.1.5]	—	—	4242.00	3314.09	790.53	2024.54	598.26	1044.47
Lizard04Caudovirales_VIRSorter_scaffold_467-gene_7|3873|4313|440|-|-|-|-|-|LytTR|54.6|6e-16(-).p1	pdi:BDI_0805	88	56	143	167	254	6.03E-33	67.045	**K07705**	Environmental Information Processing	Signal transduction	lytT, lytR	two-component system, LytTR family, response regulator LytT	ko02020	Two-component system	177.13	600.33	167.80	450.11	250.37	59.83
Lizard04Caudovirales_VIRSorter_scaffold_467-gene_10|5958|7853|1895|-|-|-|-|-|ACR_tran|273.9|1.4e-82(-).p1	bth:BT_3969	690	1	613	760	1441	0	60.145	**K15726**	Brite Hierarchies	Protein families: signaling and cellular processes	czcA, cusA, cnrA	heavy metal efflux system protein	—	—	1339.29	3402.94	1379.91	2284.88	1814.82	600.08
Lizard04Caudovirales_VIRSorter_scaffold_467-gene_12|8659|9018|359|+|-|-|-|-|-|-|-|-(+).p3	bvs:BARVI_11220	119	1	119	300	418	2.77E-61	88.235	**K15726**	Brite Hierarchies	Protein families: signaling and cellular processes	czcA, cusA, cnrA	heavy metal efflux system protein	—	—	145.94	410.65	151.65	229.85	184.24	62.56
Lizard04Caudovirales_VIRSorter_scaffold_12929-gene_2|951|2204|1253|-|-|-|-|-|Cys_Met_Meta_PP|465.4|6.1e-141(-).p1	bxy:BXY_09990	410	1	410	16	425	0	89.756	**K01740**	Metabolism	Amino acid metabolism	metY	O-acetylhomoserine (thiol)-lyase [EC:2.5.1.49]	ko00270	Cysteine and methionine metabolism	1290.87	11224.92	243.39	831.86	811.34	785.83
Lizard04Caudovirales_VIRSorter_scaffold_12929-gene_3|2423|2884|461|+|-|-|-|-|AsnC_trans_reg|73.5|5.7e-22(+).p1	bvu:BVU_2702	153	1	153	4	156	2.89E-95	87.582	**K03719**	Brite Hierarchies	Protein families: genetic information processing	lrp	Lrp/AsnC family transcriptional regulator, leucine-responsive regulatory protein	—	—	0.00	0.00	0.00	36.93	21.52	20.91
Lizard04Caudovirales_VIRSorter_scaffold_12929-gene_4|2887|3771|884|-|-|-|-|-|LysR_substrate|135.0|1.3e-40(-).p1	pdi:BDI_1820	288	1	288	2	289	1.13E-161	77.083	**K21900**	Brite Hierarchies	Protein families: genetic information processing	cysL	LysR family transcriptional regulator, transcriptional activator of the cysJI operon	—	—	125.07	1075.53	27.75	114.07	0.00	62.88
Lizard04Caudovirales_VIRSorter_scaffold_12929-gene_7|8526|8996|470|-|Phage_cluster_398|78.9|2e-23|1|Pfam-	pdi:BDI_1834	153	1	153	227	379	5.08E-96	88.889	**K01710**	Metabolism	Glycan biosynthesis and metabolism	rfbB, rmlB, rffG	dTDP-glucose 4,6-dehydratase [EC:4.2.1.46]	ko00521,ko00523,ko00525,ko01055	Streptomycin biosynthesis|Polyketide sugar unit biosynthesis|Acarbose and validamycin biosynthesis|Biosynthesis of vancomycin group antibiotics	0.00	14.09	0.00	4.20	0.00	6.12
Lizard04Caudovirales_VIRSorter_scaffold_6119-gene_1|110|2338|2228|-|Phage_cluster_1607|597.6|1.8e-	osp:Odosp_3107	741	1	741	17	756	0	88.394	**K03657**	Genetic Information Processing	Replication and repair	uvrD, pcrA	DNA helicase II / ATP-dependent DNA helicase PcrA [EC:3.6.4.12]	ko03420,ko03430	Nucleotide excision repair|Mismatch repair	0.00	0.00	0.00	3058.95	1232.27	116.29
Lizard04Caudovirales_VIRSorter_scaffold_6119-gene_4|8392|8724|332|-|-|-|-|-|Transposase_20|51.3|6.9e-15(-).p1	tfo:BFO_3048	99	1	99	277	375	1.18E-41	62.626	**K07486**	Not Included in Pathway or Brite	Unclassified: genetic information processing	K07486	transposase	—	—	12.91	2.30	1.59	7.68	1.24	1.88
Lizard04Caudovirales_VIRSorter_scaffold_10848-gene_2|204|668|464|+|Phage_cluster_7048|161.1|8.4e-	parc:CI960_02720	153	1	153	1	153	1.70E-105	92.157	**K09457**	Metabolism	Metabolism of cofactors and vitamins	queF	7-cyano-7-deazaguanine reductase [EC:1.7.1.13]	ko00790	Folate biosynthesis	0.00	974.02	21.82	210.57	0.00	231.52
Lizard04Caudovirales_VIRSorter_scaffold_10848-gene_5|2463|3845|1382|+|-|-|-|-|OEP|205.9|2.9e-62(+).p1	bcel:BcellWH2_00662	434	26	459	42	475	0	76.267	**K12340**	Environmental Information Processing	Membrane transport	tolC	outer membrane protein	ko01501,ko01503,ko02020,ko03070,ko04626,ko05133	beta-Lactam resistance|Cationic antimicrobial peptide (CAMP) resistance|Two-component system|Bacterial secretion system|Plant-pathogen interaction|Pertussis	527.74	3547.23	370.49	540.65	190.50	817.98
Lizard04Caudovirales_VIRSorter_scaffold_10848-gene_6|4012|5220|1208|+|-|-|-|-|HlyD_2|117.5|4.7e-35(+).p1	pdi:BDI_3353	404	3	402	4	407	0	75.248	**K02005**	Not Included in Pathway or Brite	Unclassified: signaling and cellular processes	ABC.CD.TX	HlyD family secretion protein	—	—	530.97	2074.56	227.45	352.43	145.54	549.87
Lizard04Caudovirales_VIRSorter_scaffold_10848-gene_7|5266|6009|743|+|Phage_cluster_405|145.1|1.8e-	parc:CI960_22780	241	1	241	1	241	2.56E-160	90.456	**K02003**	Brite Hierarchies	Protein families: signaling and cellular processes	ABC.CD.A	putative ABC transport system ATP-binding protein	—	—	233.12	903.05	91.29	198.49	59.40	225.44
Lizard04Caudovirales_VIRSorter_scaffold_10848-gene_8|6108|7328|1220|+|Phage_cluster_6774|45.5|2.3e-	parc:CI960_22785	406	1	406	1	406	0	91.872	**K02004**	Brite Hierarchies	Protein families: signaling and cellular processes	ABC.CD.P	putative ABC transport system permease protein	—	—	857.14	2595.44	310.45	346.94	223.63	640.68
Lizard04Caudovirales_VIRSorter_scaffold_8326-gene_1|1|453|452|-|Phage_cluster_1822|148.9|9.4e-45|1|-|-|-(-).p2	selo:AXE86_06070	148	3	150	3	141	1.21E-46	50.676	**K06905**	Not Included in Pathway or Brite	Poorly characterized	K06905	uncharacterized protein	—	—	0.00	0.00	0.00	25057.36	0.00	0.00
Lizard04Caudovirales_VIRSorter_scaffold_8326-gene_6|4815|5333|518|-|Phage_cluster_1430|159.1|4.5e-	mana:MAMMFC1_01723	171	1	171	2	172	3.08E-73	58.48	**K06908**	Not Included in Pathway or Brite	Poorly characterized	K06908	uncharacterized protein	—	—	0.00	0.00	0.00	11.89	0.00	0.00
Lizard04Caudovirales_VIRSorter_scaffold_8326-gene_7|5352|6788|1436|-|Phage_cluster_740|557.6|9.7e-	mana:MAMMFC1_01722	484	1	476	2	484	0	63.843	**K06907**	Not Included in Pathway or Brite	Poorly characterized	K06907	uncharacterized protein	—	—	0.00	0.00	0.00	34.11	0.00	0.00
Lizard04Caudovirales_VIRSorter_scaffold_1669-gene_1|1|909|908|-|-|-|-|UT|275.3|3.3e-83(-).p1	osp:Odosp_1239	287	16	302	15	301	2.14E-130	64.46	**K08717**	Brite Hierarchies	Protein families: signaling and cellular processes	utp	urea transporter	—	—	686019.64	4442559.60	592018.87	1496282.56	1449335.81	2234115.20
Lizard04Caudovirales_VIRSorter_scaffold_1669-gene_2|948|2279|1331|-|-|-|-|-|UreD|115.9|1.2e-34(-).p1	afd:Alfi_1057	297	152	442	8	304	1.82E-154	71.717	**K03190**	Not Included in Pathway or Brite	Unclassified: genetic information processing	ureD, ureH	urease accessory protein	—	—	2742.25	7731.68	967.26	2537.45	1891.80	3429.90
Lizard04Caudovirales_VIRSorter_scaffold_1669-gene_3|2303|2725|422|-|-|-|-|-|UreF|56.1|3.1e-16(-).p1	afd:Alfi_1059	139	1	139	92	230	8.60E-60	63.309	**K03188**	Not Included in Pathway or Brite	Unclassified: genetic information processing	ureF	urease accessory protein	—	—	219.49	795.82	139.16	278.23	217.32	407.18
Lizard04Caudovirales_VIRSorter_scaffold_1669-gene_4|2722|3150|428|-|-|-|-|-|-|-|-|-(-).p1	afd:Alfi_1060	126	1	126	2	126	1.21E-49	63.492	**K03187**	Not Included in Pathway or Brite	Unclassified: genetic information processing	ureE	urease accessory protein	—	—	132.65	398.76	47.42	114.41	118.75	158.33
Lizard04Caudovirales_VIRSorter_scaffold_1669-gene_5|3674|5392|1718|-|-|-|-|-|Amidohydro_1|186.9|5.2e-56(-).p1	afd:Alfi_1061	571	1	571	2	572	0	83.713	**K01428**	Metabolism	Nucleotide metabolism	ureC	urease subunit alpha [EC:3.5.1.5]	ko00220,ko00230,ko00791,ko05120	Arginine biosynthesis|Purine metabolism|Atrazine degradation|Epithelial cell signaling in Helicobacter pylori infection	1218.61	4577.24	610.63	1380.60	1001.82	2120.61

Query ORF ID	Subject gene ID	Alignment length	Query start	Query end	Subject start	Subject end	e-value	Identity(%)	KO	Category	Subcategory	Gene name	Description	Pathway	Pathway definition	Abundance (fpkm)					
																(1) PE-NMC	(2) PE-NQ1	(3) PE-NQ2	(4) PT-SZZ	(5) PT-ZB	(6) PT-RT
Lizard04Caudovirales_VIRSorter_scaffold_1669-gene_6|5401|5955|554|-|-|-|-|-|Urease_beta|136.1|2.1e-41(-).p1	pary:A4V02_02710	175	6	180	27	201	1.41E-84	68.571	**K01429**	Metabolism	Nucleotide metabolism	ureB	urease subunit beta [EC:3.5.1.5]	ko00220,ko00230,ko00791	Arginine biosynthesis|Purine metabolism|Atrazine degradation	100.28	339.89	50.27	129.24	88.81	154.95
Lizard04Caudovirales_VIRSorter_scaffold_1669-gene_7|5974|6276|302|-|-|-|-|-|Urease_gamma|136.3|2e-41(-).p1	afd:Alfi_1063	99	1	99	2	100	1.97E-56	87.879	**K01430**	Metabolism	Nucleotide metabolism	ureA	urease subunit gamma [EC:3.5.1.5]	ko00220,ko00230,ko00791	Arginine biosynthesis|Purine metabolism|Atrazine degradation	24.18	75.21	6.71	18.58	5.24	34.59
Lizard04Caudovirales_VIRSorter_scaffold_1669-gene_8|6416|6793|377|-|-|-|-|-|CRCB|104.5|2.2e-31(-).p1	osp:Odosp_1237	124	1	124	2	125	9.60E-71	80.645	**K06199**	Brite Hierarchies	Protein families: signaling and cellular processes	crcB, FEX	fluoride exporter	—	—	0.00	58.24	8.33	20.09	19.50	24.84
Lizard04Caudovirales_VIRSorter_scaffold_4882-gene_5|2383|3234|851|-|Phage_cluster_52|249.3|5.9e-	psor:RSJ16_07610	286	3	281	4	280	6.38E-77	46.853	**K01358**	Cellular Processes	Cell growth and death	clpP, CLPP	ATP-dependent Clp protease, protease subunit [EC:3.4.21.92]	ko04112,ko04212	Cell cycle - Caulobacter|Longevity regulating pathway - worm	0.00	0.00	0.00	33.05	0.00	0.41
Lizard04Caudovirales_VIRSorter_scaffold_3559-gene_1|1|456|455|+|-|-|-|-|Bgal_small_N|116.9|5.7e-35(+).p1	bvu:BVU_0607	151	1	151	878	1028	1.14E-68	72.848	**K01190**	Metabolism	Carbohydrate metabolism	lacZ	beta-galactosidase [EC:3.2.1.23]	ko00052,ko00511,ko00600	Galactose metabolism|Other glycan degradation|Sphingolipid metabolism	0.00	136420.39	8902.54	28636.99	72988.13	36899.06
Lizard04Caudovirales_VIRSorter_scaffold_3559-gene_2|684|2195|1511|+|Phage_cluster_7861|119.8|6.3e-36|1|FGE-	pdi:BDI_3845	500	1	499	25	524	0	87.6	**K13444**	Cellular Processes	Transport and catabolism	SUMF1, FGE	formylglycine-generating enzyme [EC:1.8.3.7]	ko04142	Lysosome	0.00	1684.44	13.02	497.17	350.61	237.36
Lizard04Caudovirales_VIRSorter_scaffold_3559-gene_7|5985|6418|433|+|-|-|-|-|Plug|43.8|1.8e-12(+).p1	parc:CI960_02635	144	1	144	1	143	2.13E-73	81.25	**K02014**	Brite Hierarchies	Protein families: signaling and cellular processes	TC.FEV.OM	iron complex outermembrane recepter protein	—	—	18.10	207.13	2.97	41.27	26.13	8.96
Lizard04Caudovirales_VIRSorter_scaffold_2180-gene_2|1589|2209|620|-|-|-|-|-|-|-|-|-(-).p1	pdi:BDI_1990	202	2	203	3	203	5.80E-75	57.921	**K06133**	Metabolism	Metabolism of cofactors and vitamins	LYS5, acpT	4'-phosphopantetheinyl transferase [EC:2.7.8.-]	ko00770	Pantothenate and CoA biosynthesis	0.00	1.87	0.00	281.59	6.56	0.31
Lizard04Caudovirales_VIRSorter_scaffold_2180-gene_4|2784|4121|1337|-|-|-|-|-|DUF21|122.5|8.1e-37(-).p1	pdi:BDI_1992	443	1	443	2	444	0	85.553	**K03699**	Brite Hierarchies	Protein families: signaling and cellular processes	tlyC	putative hemolysin	—	—	0.00	0.00	0.00	309.87	26.22	0.88
Lizard04Caudovirales_VIRSorter_scaffold_2180-gene_5|4147|4638|491|-|Phage_cluster_9|142.1|7.4e-	pdi:BDI_1993	168	1	162	2	169	4.04E-90	80.357	**K03111**	Genetic Information Processing	Replication and repair	ssb	single-strand DNA-binding protein	ko03030,ko03430,ko03440	DNA replication|Mismatch repair|Homologous recombination	0.00	0.00	0.00	54.38	2.51	0.12
Lizard04Caudovirales_VIRSorter_scaffold_2180-gene_6|4682|5764|1082|-|-|-|-|-|HhH-GPD|60.7|1e-17(-).p1	pdi:BDI_1994	354	4	357	6	359	0	72.316	**K03575**	Genetic Information Processing	Replication and repair	mutY	A/G-specific adenine glycosylase [EC:3.2.2.31]	ko03410	Base excision repair	0.00	0.32	0.00	87.16	31.18	0.32
Lizard04Caudovirales_VIRSorter_scaffold_13064-gene_1|1|414|413|+|Phage_cluster_118|105.1|2e-31|0|-|-|-(+).p1	csb:CLSA_c40930	94	40	133	417	505	8.56E-06	43.617	**K21449**	Brite Hierarchies	Protein families: signaling and cellular processes	ata, sadA, emaA	trimeric autotransporter adhesin	—	—	0.00	0.00	0.00	1900780.00	166830.02	0.00
Lizard04Caudovirales_VIRSorter_scaffold_24606-gene_3|2418|3839|1421|-|Phage_cluster_1610|92.2|1.9e-	paci:A4V11_04270	426	55	460	26	423	5.69E-14	23.474	**K06909**	Not Included in Pathway or Brite	Unclassified: genetic information processing	xtmB	phage terminase large subunit	—	—	0.00	0.00	0.00	109.55	0.00	0.00
Lizard04Caudovirales_VIRSorter_scaffold_27764-gene_6|3196|5280|2084|+|Phage_cluster_423|171.8|1.3e-	dti:Desti_0995	601	119	693	171	764	2.60E-44	26.456	**K06919**	Not Included in Pathway or Brite	Unclassified: genetic information processing	K06919	putative DNA primase/helicase	—	—	11.30	3.25	4.18	58.24	0.00	1.23
Lizard04Caudovirales_VIRSorter_scaffold_2321-gene_1|109|1113|1004|+|gi_209427798_ref_YP_002274211.1_|77.4|4.3	osp:Odosp_0480	334	1	334	150	482	1.71E-122	53.593	**K02478**	Brite Hierarchies	Protein families: metabolism	K02478	two-component system, LytTR family, sensor kinase [EC:2.7.13.3]	—	—	0.00	0.00	0.00	9753.65	5739.56	24770.89
Lizard04Caudovirales_VIRSorter_scaffold_22747-gene_2|1493|3964|2471|+|-|-|-|-|Pfam-B_18789|176.2|4.3e-53(+).p1	pdi:BDI_1243	823	1	823	1	822	0	86.877	**K07456**	Genetic Information Processing	Replication and repair	mutS2	DNA mismatch repair protein MutS2	ko03430	Mismatch repair	0.00	48.67	17.89	702.50	118.73	168.72
Lizard04Caudovirales_VIRSorter_scaffold_15413-gene_1|1|318|317|+|-|-|-|-|CM_2|73.9|6e-22(+).p1	pdi:BDI_1379	104	1	104	253	356	1.40E-55	85.577	**K04516**	Metabolism	Amino acid metabolism	AROA1, aroA	chorismate mutase [EC:5.4.99.5]	ko00400	Phenylalanine, tyrosine and tryptophan biosynthesis	108318.89	311394.36	40061.43	150344.18	93841.89	114633.09
Lizard04Caudovirales_VIRSorter_scaffold_15413-gene_2|585|1328|743|+|-|-|-|-|PDH|48.9|2.7e-14(+).p1	pdi:BDI_1380	247	1	247	11	257	7.58E-166	90.283	**K04517**	Metabolism	Amino acid metabolism	tyrA2	prephenate dehydrogenase [EC:1.3.1.12]	ko00400,ko00401	Phenylalanine, tyrosine and tryptophan biosynthesis|Novobiocin biosynthesis	246.88	1890.93	266.32	752.64	481.24	716.54
Lizard04Caudovirales_VIRSorter_scaffold_7434-gene_9|3620|4033|413|+|Phage_cluster_9|145.7|5.7e-44|1|SSB|121.3|1e-	blr:BRLA_c031440	138	1	137	2	139	2.96E-40	46.377	**K03111**	Genetic Information Processing	Replication and repair	ssb	single-strand DNA-binding protein	ko03030,ko03430,ko03440	DNA replication|Mismatch repair|Homologous recombination	0.00	0.00	0.00	14.83	0.00	0.00
Lizard04Caudovirales_VIRSorter_scaffold_2323-gene_9|3759|4568|809|-|Phage_cluster_4352|216.5|2.4e-	amij:EQM06_09690	248	23	268	20	263	9.83E-64	45.968	**K02315**	Brite Hierarchies	Protein families: genetic information processing	dnaC	DNA replication protein DnaC	—	—	0.00	0.00	0.00	123.80	5.55	0.00
Lizard04Caudovirales_VIRSorter_scaffold_18100-gene_1|1|579|578|-|-|-|-|-|Orn_DAP_Arg_deC|119.3|4.7e-36(-).p1	osp:Odosp_2583	192	1	192	434	625	1.47E-133	96.875	**K01585**	Metabolism	Amino acid metabolism	speA	arginine decarboxylase [EC:4.1.1.19]	ko00330	Arginine and proline metabolism	0.00	0.00	0.00	82331.34	62561.26	63466.39
Lizard04Caudovirales_VIRSorter_scaffold_18100-gene_2|461|1804|1343|-|-|-|-|-|Orn_Arg_deC_N|179.5|4.5e-54(-).p1	osp:Odosp_2583	382	1	382	2	383	0	94.241	**K01585**	Metabolism	Amino acid metabolism	speA	arginine decarboxylase [EC:4.1.1.19]	ko00330	Arginine and proline metabolism	0.00	0.00	0.00	869.67	542.83	935.95
Lizard04Caudovirales_VIRSorter_scaffold_15067-gene_2|1186|3831|2645|-|Phage_cluster_256|222.5|5.7e-	bfb:VU15_01330	881	1	880	2	882	0	87.514	**K02621**	Brite Hierarchies	Protein families: genetic information processing	parC	topoisomerase IV subunit A [EC:5.6.2.2]	—	—	152.22	1132.75	7.51	344.08	52.75	24.89
Lizard04Caudovirales_VIRSorter_scaffold_25812-gene_1|67|387|320|-|Phage_cluster_3529|81.1|2.8e-24|1|Thioredoxin|85.9|9.2e-26(-).p2	bfs:BF9343_1215	100	1	100	6	105	4.40E-33	50	**K03671**	Organismal Systems	Immune system	trxA	thioredoxin 1	ko04621,ko05132,ko05418	NOD-like receptor signaling pathway|Salmonella infection|Fluid shear stress and atherosclerosis	0.00	1482.83	0.00	641.13	155.63	0.00
Lizard04Caudovirales_VIRSorter_scaffold_455-gene_2|1316|2455|1139|+|Phage_cluster_2806|65.0|3.2e-19|1|-|-|-(+).p1	spoa:EQM13_01985	360	9	352	3	330	2.05E-34	27.778	**K06871**	Not Included in Pathway or Brite	Poorly characterized	K06871	uncharacterized protein	—	—	0.00	10.14	0.00	108.80	0.00	0.00
Lizard04Caudovirales_VIRSorter_scaffold_5277-gene_2|832|2670|1838|-|-|-|-|-|Peptidase_U32|233.2|1.3e-70(-).p1	pdi:BDI_1489	610	3	608	4	613	0	80	**K08303**	Human Diseases	Infectious disease: bacterial	K08303	putative protease [EC:3.4.-.-]	ko05120	Epithelial cell signaling in Helicobacter pylori infection	0.00	185.35	5.35	344.19	25.06	914.79
Lizard04Caudovirales_VIRSorter_scaffold_5277-gene_3|2968|3636|668|-|-|-|-|-|HTS|328.8|1.5e-99(-).p1	pdi:BDI_1490	221	1	221	90	310	1.89E-147	87.783	**K00651**	Metabolism	Energy metabolism	metA	homoserine O-succinyltransferase/O-acetyltransferase [EC:2.3.1.46 2.3.1.31]	ko00270,ko00920	Cysteine and methionine metabolism|Sulfur metabolism	0.00	17.99	0.00	12.06	0.00	27.52
Lizard04Caudovirales_VIRSorter_scaffold_9539-gene_3|1695|2870|1175|-|Phage_cluster_4339|279.0|3.4e-84|2|S-	tex:Teth514_1243	388	4	390	5	388	1.10E-167	62.371	**K00789**	Metabolism	Amino acid metabolism	metK	S-adenosylmethionine synthetase [EC:2.5.1.6]	ko00270	Cysteine and methionine metabolism	0.00	0.87	0.00	103.48	0.00	0.00
Lizard04Caudovirales_VIRSorter_scaffold_657-gene_2|307|666|359|+|Phage_cluster_4580|42.8|2.2e-12|1|-|-|-(+).p1	hap:HAPS_0519	37	62	98	716	752	9.61E-08	75.676	**K21449**	Brite Hierarchies	Protein families: signaling and cellular processes	ata, sadA, emaA	trimeric autotransporter adhesin	—	—	0.00	0.00	0.00	314.82	0.00	0.00
Lizard04Caudovirales_VIRSorter_scaffold_3060-gene_2|693|998|305|+|Phage_cluster_8319|49.1|2.7e-	spir:CWM47_03590	99	1	99	143	241	1.22E-18	42.424	**K07741**	Not Included in Pathway or Brite	Unclassified: genetic information processing	antB	anti-repressor protein	—	—	0.00	0.00	0.00	20.66	0.00	0.00
Lizard04Caudovirales_VIRSorter_scaffold_11733-gene_4|1508|2149|641|+|Phage_cluster_1958|64.9|4.3e-	stk:STP_0841	229	1	205	1	220	2.22E-28	39.301	**K01447**	Not Included in Pathway or Brite	Unclassified: metabolism	xlyAB	N-acetylmuramoyl-L-alanine amidase [EC:3.5.1.28]	—	—	0.00	0.00	0.00	75.96	0.00	0.00
Lizard04Caudovirales_VIRSorter_scaffold_907-gene_2|91|1371|1280|+|-|-|-|-|PTR2|110.7|4.8e-33(+).p1	bsa:Bacsa_1427	428	1	426	83	510	0	76.402	**K03305**	Not Included in Pathway or Brite	Unclassified: signaling and cellular processes	TC.POT	proton-dependent oligopeptide transporter, POT family	—	—	0.00	2639.76	0.00	1770.14	1031.23	627.15
Lizard04Caudovirales_VIRSorter_scaffold_907-gene_4|1789|2769|980|+|-|-|-|-|BATS|74.5|3.1e-22(+).p1	parc:CI960_14670	323	2	324	3	325	1.66E-180	74.303	**K01012**	Metabolism	Metabolism of cofactors and vitamins	bioB	biotin synthase [EC:2.8.1.6]	ko00780	Biotin metabolism	0.00	571.08	9.95	330.15	29.14	59.95
Lizard04Caudovirales_VIRSorter_scaffold_23947-gene_1|2|1021|1019|+|Phage_cluster_52|189.7|6.6e-	med:MELS_1094	308	1	307	54	323	2.16E-58	38.961	**K01358**	Cellular Processes	Cell growth and death	clpP, CLPP	ATP-dependent Clp protease, protease subunit [EC:3.4.21.92]	ko04112,ko04212	Cell cycle - Caulobacter|Longevity regulating pathway - worm	0.00	0.00	0.00	66223.03	0.00	0.00
Lizard04Caudovirales_VIRSorter_scaffold_4842-gene_5|2209|2565|356|-|Phage_cluster_164|47.5|5.6e-14|1|-|-|-(-).p1	arf:AR1Y2_1345	123	3	117	8	127	7.90E-14	34.959	**K07273**	Not Included in Pathway or Brite	Unclassified: signaling and cellular processes	acm	lysozyme	—	—	0.00	0.00	0.00	4.86	0.00	0.00
Lizard04Caudovirales_VIRSorter_scaffold_11970-gene_3|639|968|329|-|Phage_cluster_4362|99.3|1.7e-29|2|-|-|-(-).p1	csh:Closa_3662	79	12	90	202	280	9.93E-21	53.165	**K03660**	Genetic Information Processing	Replication and repair	OGG1	N-glycosylase/DNA lyase [EC:3.2.2.- 4.2.99.18]	ko03410	Base excision repair	0.00	0.00	0.00	33.61	0.00	0.00
Lizard04Caudovirales_VIRSorter_scaffold_6635-gene_4|1495|2664|1169|-|Phage_cluster_457|500.1|3.6e-	ccl:Clocl_0797	386	6	388	12	396	1.00E-155	57.513	**K14415**	Brite Hierarchies	Protein families: genetic information processing	RTCB, rtcB	tRNA-splicing ligase RtcB (3'-phosphate/5'-hydroxy nucleic acid ligase) [EC:6.5.1.8]	—	—	0.00	0.00	0.00	43.10	0.00	0.33
Lizard04Caudovirales_VIRSorter_scaffold_1406-gene_1|17|1486|1469|-|Phage_cluster_2040|371.6|3.4e-	parc:CI960_07185	484	1	484	2	485	0	76.24	**K00036**	Metabolism	Carbohydrate metabolism	G6PD, zwf	glucose-6-phosphate 1-dehydrogenase [EC:1.1.1.49 1.1.1.363]	ko00030,ko00480,ko05230	Pentose phosphate pathway|Glutathione metabolism|Central carbon metabolism in cancer	116814.49	666401.39	119398.76	327219.69	215897.67	62221.95
Lizard04Caudovirales_VIRSorter_scaffold_1406-gene_3|1860|2603|743|+|-|-|-|-|-|-|-|-|-(+).p2	parc:CI960_07180	226	1	226	21	246	8.74E-130	76.991	**K00033**	Metabolism	Carbohydrate metabolism	PGD, gnd, gntZ	6-phosphogluconate dehydrogenase [EC:1.1.1.44 1.1.1.343]	ko00030,ko00480	Pentose phosphate pathway|Glutathione metabolism	427.06	1258.01	205.81	683.21	285.90	124.85
Lizard04Caudovirales_VIRSorter_scaffold_3506-gene_4|718|1491|773|+|Phage_cluster_164|148.3|1.9e-	rus:RBI_II00521	217	7	222	6	210	9.36E-43	40.553	**K07273**	Not Included in Pathway or Brite	Unclassified: signaling and cellular processes	acm	lysozyme	—	—	0.00	0.00	0.00	99.71	0.00	0.00
Lizard04Caudovirales_VIRSorter_scaffold_34080-gene_2|201|1271|1070|-|-|-|-|-|BPD_transp_1|112.7|9.9e-34(-).p1	mbf:BHV42_08765	351	5	355	5	355	0	74.359	**K02034**	Cellular Processes	Cellular community - prokaryotes	ABC.PE.P1	peptide/nickel transport system permease protein	ko02024	Quorum sensing	0.00	0.00	0.00	391.80	0.00	58.74
Lizard04Caudovirales_VIRSorter_scaffold_34080-gene_3|1271|2239|968|-|-|-|-|-|BPD_transp_1|153.0|4.3e-46(-).p1	mbf:BHV42_08760	318	1	318	2	319	2.21E-176	76.101	**K02033**	Cellular Processes	Cellular community - prokaryotes	ABC.PE.P	peptide/nickel transport system permease protein	ko02024	Quorum sensing	0.00	0.00	0.00	53.51	0.00	6.19
Lizard04Caudovirales_VIRSorter_scaffold_16525-gene_2|257|1150|893|+|-|-|-|-|DUF4143|71.1|4.7e-21(+).p1	ert:EUR_01470	299	1	297	145	442	1.70E-123	59.866	**K07133**	Not Included in Pathway or Brite	Poorly characterized	K07133	uncharacterized protein	—	—	0.00	0.00	0.00	181.07	40.57	11.49
Lizard04Caudovirales_VIRSorter_scaffold_16509-gene_2|66|380|314|-|Phage_cluster_3723|69.2|1.8e-20|2|-|-|-(-).p2	cpro:CPRO_14020	82	16	97	16	96	6.30E-16	42.683	**K06903**	Not Included in Pathway or Brite	Poorly characterized	K06903	uncharacterized protein	—	—	0.00	0.00	0.00	54.24	157.98	0.00
Lizard04Caudovirales_VIRSorter_scaffold_16509-gene_5|1203|2117|914|-|Phage_cluster_1822|267.5|8.8e-	cace:CACET_c15710	313	1	302	28	338	2.79E-63	35.783	**K06905**	Not Included in Pathway or Brite	Poorly characterized	K06905	uncharacterized protein	—	—	0.00	0.00	0.00	53.56	0.00	0.00
Lizard04Caudovirales_VIRSorter_scaffold_19006-gene_3|482|1855|1373|+|Phage_cluster_118|562.3|3.8e-170|0|-|-|-(+).p1	csb:CLSA_c40930	87	370	454	419	505	1.28E-09	44.828	**K21449**	Brite Hierarchies	Protein families: signaling and cellular processes	ata, sadA, emaA	trimeric autotransporter adhesin	—	—	0.00	0.00	0.00	178.24	0.00	0.00
Lizard04Caudovirales_VIRSorter_scaffold_12828-gene_1|140|838|698|+|Phage_cluster_376|209.7|3e-63|1|-|-|-(+).p1	csh:Closa_0735	229	1	229	36	264	1.70E-133	75.983	**K06223**	Genetic Information Processing	Replication and repair	dam	DNA adenine methylase [EC:2.1.1.72]	ko03430	Mismatch repair	0.00	0.00	0.00	230.12	0.00	0.00
Lizard04Caudovirales_VIRSorter_scaffold_19887-gene_1|1|582|581|-|Phage_cluster_4482|144.8|1.2e-43|1|-|-|-(-).p1	calt:Cal6303_2202	110	1	109	1180	1289	3.40E-07	32.727	**K01406**	Human Diseases	Drug resistance: antimicrobial	prtC	serralysin [EC:3.4.24.40]	ko01503	Cationic antimicrobial peptide (CAMP) resistance	0.00	0.00	0.00	96649.83	0.00	0.00
Lizard04Caudovirales_VIRSorter_scaffold_5295-gene_2|149|538|389|+|Phage_cluster_204|127.8|2.9e-38|1|-|-|-(+).p1	ova:OBV_43150	129	1	129	2	130	4.05E-52	68.217	**K06919**	Not Included in Pathway or Brite	Unclassified: genetic information processing	K06919	putative DNA primase/helicase	—	—	0.00	9.95	0.00	48.05	0.00	9.91
Lizard04Caudovirales_VIRSorter_scaffold_31254-gene_1|178|810|632|-|Phage_cluster_945|131.1|3.9e-	css:Cst_c04310	202	15	203	20	217	8.78E-47	48.02	**K01447**	Not Included in Pathway or Brite	Unclassified: metabolism	xlyAB	N-acetylmuramoyl-L-alanine amidase [EC:3.5.1.28]	—	—	0.00	0.00	0.00	201.10	0.00	0.00
Lizard04Caudovirales_VIRSorter_scaffold_29391-gene_3|712|1092|380|+|Phage_cluster_6578|159.7|7e-	bpro:PMF13cell1_05629	117	5	119	4	120	9.99E-43	63.248	**K01448**	Human Diseases	Drug resistance: antimicrobial	amiABC	N-acetylmuramoyl-L-alanine amidase [EC:3.5.1.28]	ko01503	Cationic antimicrobial peptide (CAMP) resistance	0.00	0.00	0.00	20.11	0.00	0.69
Lizard04Caudovirales_VIRSorter_scaffold_16706-gene_1|1|678|677|-|Phage_cluster_5430|147.5|3.4e-44|1|AAA_23|51.2|1.4e-14(-).p1	btht:H175_ch1821	220	1	220	4	223	5.67E-60	46.818	**K03546**	Brite Hierarchies	Protein families: genetic information processing	sbcC, rad50	DNA repair protein SbcC/Rad50	—	—	0.00	0.00	0.00	35796.23	0.00	0.00
Lizard04Caudovirales_VIRSorter_scaffold_27694-gene_3|481|861|380|-|-|-|-|-|-|-|-|-(-).p1	dfg:B0537_15475	134	1	125	2	130	2.10E-26	41.791	**K07497**	Not Included in Pathway or Brite	Unclassified: genetic information processing	K07497	putative transposase	—	—	0.00	6.17	9.25	14.88	0.00	0.00
Lizard04Caudovirales_VIRSorter_scaffold_20289-gene_1|1|492|491|+|Phage_cluster_423|160.9|2.6e-48|1|-|-|-(+).p1	bprs:CK3_30100	167	1	163	330	496	9.61E-89	77.844	**K06919**	Not Included in Pathway or Brite	Unclassified: genetic information processing	K06919	putative DNA primase/helicase	—	—	0.00	2965.66	4451.27	10738.87	0.00	491.99
Lizard04Caudovirales_VIRSorter_scaffold_25498-gene_3|458|793|335|-|Phage_cluster_376|58.9|2.9e-17|1|-|-|-(-).p2	cpro:CPRO_13780	82	26	107	184	263	5.95E-20	47.561	**K06223**	Genetic Information Processing	Replication and repair	dam	DNA adenine methylase [EC:2.1.1.72]	ko03430	Mismatch repair	0.00	0.00	0.00	15.63	0.00	0.00
Lizard04unclassified_bacterial_viruses_VIRSorter_scaffold_5626-gene_1|3|482|479|+|-|-|-|-|Pfam-B_8079|54.9|3.6e-17(+).p1	osp:Odosp_3270	117	39	155	133	249	7.43E-73	95.726	**K09013**	Brite Hierarchies	Protein families: signaling and cellular processes	sufC	Fe-S cluster assembly ATP-binding protein	—	—	0.00	0.00	0.00	2386.42	13902.50	27387.30
Lizard04unclassified_bacterial_viruses_VIRSorter_scaffold_5626-gene_2|496|1254|758|+|-|-|-|-|-|-|-|-|-(+).p1	osp:Odosp_3271	252	1	252	1	252	1.04E-150	81.746	**K09015**	Not Included in Pathway or Brite	Poorly characterized	sufD	Fe-S cluster assembly protein SufD	—	—	0.00	0.00	0.00	50.52	357.37	626.89
Lizard04unclassified_bacterial_viruses_VIRSorter_scaffold_5626-gene_3|1369|1860|491|+|-|-|-|-|UPF0051|177.6|2.3e-54(+).p1	osp:Odosp_3271	161	1	161	292	452	4.86E-104	90.683	**K09015**	Not Included in Pathway or Brite	Poorly characterized	sufD	Fe-S cluster assembly protein SufD	—	—	0.00	0.00	0.00	13.07	45.70	125.78

Query ORF ID	Subject gene ID	Alignment length	Query start	Query end	Subject start	Subject end	e-value	Identity(%)	KO	Category	Subcategory	Gene name	Description	Pathway	Pathway definition	Abundance (fpkm)					
																(1) PE-NMC	(2) PE-NQ1	(3) PE-NQ2	(4) PT-SZZ	(5) PT-ZB	(6) PT-RT
Lizard04unclassified_bacterial_viruses_VIRSorter_scaffold_5626-gene_4|1874|3094|1220|+|Phage_cluster_1231|165.0|1.6e-	osp:Odosp_3272	404	1	404	1	404	0	81.683	**K11717**	Metabolism	Metabolism of other amino acids	sufS	cysteine desulfurase / selenocysteine lyase [EC:2.8.1.7 4.4.1.16]	ko00450	Selenocompound metabolism	0.00	0.00	0.00	40.11	216.99	465.47
Lizard04unclassified_bacterial_viruses_VIRSorter_scaffold_5626-gene_5|3104|3562|458|+|Phage_cluster_5579|81.5|3.7e-	osp:Odosp_3273	141	12	152	1	141	2.77E-84	82.27	**K02426**	Not Included in Pathway or Brite	Poorly characterized	sufE	cysteine desulfuration protein SufE	—	—	0.00	0.00	0.00	23.06	43.67	85.75
Lizard04unclassified_bacterial_viruses_VIRSorter_scaffold_5626-gene_7|3887|4789|902|+|-|-|-|-|Fer4_16|60.7|1.5e-18(+).p1	buy:D8S85_08410	298	4	299	2	298	5.95E-116	55.705	**K18979**	Brite Hierarchies	Protein families: genetic information processing	queG	epoxyqueuosine reductase [EC:1.17.99.6]	—	—	0.00	0.00	0.00	23.02	174.36	165.94
Lizard04unclassified_bacterial_viruses_VIRSorter_scaffold_5626-gene_8|4873|6372|1499|+|-|-|-|-|BATS|64.8|4.2e-20(+).p1	osp:Odosp_3276	499	1	499	1	499	0	92.585	**K03150**	Metabolism	Metabolism of cofactors and vitamins	thiH	2-iminoacetate synthase [EC:4.1.99.19]	ko00730	Thiamine metabolism	0.00	0.00	0.00	66.11	301.70	367.00
Lizard04unclassified_bacterial_viruses_VIRSorter_scaffold_5626-gene_11|8570|9274|704|-|-|-|-|-|Pfam-B_4295|42.3|5.4e-13(-).p1	shg:Sph21_1835	241	1	233	3	242	2.76E-47	38.589	**K01104**	Environmental Information Processing	Signal transduction	E3.1.3.48	protein-tyrosine phosphatase [EC:3.1.3.48]	ko02020	Two-component system	0.00	0.00	0.00	8.35	55.97	62.35
Lizard04unclassified_bacterial_viruses_VIRSorter_scaffold_1148-gene_8|5558|7591|2033|-|Phage_cluster_197|730.2|1.5e-221|1|Pfam-	ova:OBV_43170	676	1	676	3	672	0	65.976	**K02334**	Not Included in Pathway or Brite	Unclassified: metabolism	dpo	DNA polymerase bacteriophage-type [EC:2.7.7.7]	—	—	0.00	0.00	0.00	72.78	0.00	1.95
Lizard04unclassified_bacterial_viruses_VIRSorter_scaffold_17448-gene_2|393|2381|1988|-|Phage_cluster_197|770.1|1.2e-233|1|Pfam-	cpas:Clopa_0561	660	2	661	3	657	0	65	**K02334**	Not Included in Pathway or Brite	Unclassified: metabolism	dpo	DNA polymerase bacteriophage-type [EC:2.7.7.7]	—	—	0.00	22.64	45.31	409.88	0.00	11.27
Lizard04unclassified_bacterial_viruses_VIRSorter_scaffold_10024-gene_1|151|669|518|+|Phage_cluster_460|171.8|1.1e-	cbm:CBF_2413	162	3	141	4	165	8.56E-37	43.827	**K07474**	Not Included in Pathway or Brite	Unclassified: genetic information processing	xtmA	phage terminase small subunit	—	—	0.00	0.00	0.00	165.94	0.00	0.00
Lizard04unclassified_bacterial_viruses_VIRSorter_scaffold_5519-gene_1|33|980|947|-|Phage_cluster_579|146.1|8.8e-	bka:AH68_05245	259	72	314	18	266	2.24E-38	41.699	**K01447**	Not Included in Pathway or Brite	Unclassified: metabolism	xlyAB	N-acetylmuramoyl-L-alanine amidase [EC:3.5.1.28]	—	—	0.00	0.00	0.00	1518.63	0.00	0.00
Lizard04unclassified_bacterial_viruses_VIRSorter_scaffold_5519-gene_6|2559|3020|461|-|Phage_cluster_1197|159.3|1.1e-48|2|-|-|-(-).p1	ele:Elen_2643	151	1	150	228	378	1.36E-69	72.185	**K00986**	Not Included in Pathway or Brite	Unclassified: metabolism	ltrA	RNA-directed DNA polymerase [EC:2.7.7.49]	—	—	0.00	0.00	0.00	5.60	0.00	0.00
Lizard04unclassified_bacterial_viruses_VIRSorter_scaffold_22043-gene_2|839|2848|2009|-|Phage_cluster_197|731.3|6.7e-222|1|Pfam-	ova:OBV_43170	676	4	668	3	672	0	61.834	**K02334**	Not Included in Pathway or Brite	Unclassified: metabolism	dpo	DNA polymerase bacteriophage-type [EC:2.7.7.7]	—	—	0.00	5.30	15.92	170.66	62.14	48.67
Lizard04unclassified_bacterial_viruses_VIRSorter_scaffold_24276-gene_1|2|949|947|+|Phage_cluster_152|361.2|5.2e-	cfm:BJL90_15775	293	1	293	59	351	4.69E-113	54.608	**K00986**	Not Included in Pathway or Brite	Unclassified: metabolism	ltrA	RNA-directed DNA polymerase [EC:2.7.7.49]	—	—	0.00	0.00	0.00	37586.05	5213.44	0.00
Lizard04unclassified_bacterial_viruses_VIRSorter_scaffold_554-gene_2|468|1364|896|-|Phage_cluster_1337|277.8|8.2e-85|1|-|-|-(-).p1	gsb:GSUB_15980	298	5	291	69	351	1.77E-61	42.617	**K13444**	Cellular Processes	Transport and catabolism	SUMF1, FGE	formylglycine-generating enzyme [EC:1.8.3.7]	ko04142	Lysosome	0.00	0.00	0.00	145.33	0.00	0.00
Lizard04unclassified_bacterial_viruses_VIRSorter_scaffold_11840-gene_4|741|1145|404|-|Phage_cluster_579|149.2|1e-	bhan:CGC63_04110	103	8	101	69	169	1.24E-26	50.485	**K01447**	Not Included in Pathway or Brite	Unclassified: metabolism	xlyAB	N-acetylmuramoyl-L-alanine amidase [EC:3.5.1.28]	—	—	0.00	0.00	0.00	72.46	0.00	0.00
Lizard04unclassified_dsDNA_phages_VIRSorter_scaffold_6680-gene_1|2|376|374|+|-|-|-|-|Pfam-B_12464|56.2|2.7e-18(+).p1	pdi:BDI_0607	125	1	124	25	149	9.96E-43	64	**K01843**	Metabolism	Amino acid metabolism	kamA	lysine 2,3-aminomutase [EC:5.4.3.2]	ko00310	Lysine degradation	0.00	135678.97	15579.44	5369.44	5213.44	32471.17
Lizard04unclassified_dsDNA_phages_VIRSorter_scaffold_6680-gene_2|380|2029|1649|+|-|-|-|-|Pfam-B_12464|377.9|3.9e-116(+).p1	pdi:BDI_0607	555	1	549	152	704	0	81.261	**K01843**	Metabolism	Amino acid metabolism	kamA	lysine 2,3-aminomutase [EC:5.4.3.2]	ko00310	Lysine degradation	10261.79	20716.70	3115.89	1488.37	2140.25	4378.69
Lizard04unclassified_dsDNA_phages_VIRSorter_scaffold_6680-gene_4|3871|4701|830|-|Phage_cluster_8887|43.4|2.3e-	buy:D8S85_16620	233	42	271	54	284	2.55E-65	44.635	**K18954**	Brite Hierarchies	Protein families: genetic information processing	pobR	AraC family transcriptional regulator, transcriptional activator of pobA	—	—	699.55	1235.76	201.23	95.25	158.92	282.92
Lizard04unclassified_dsDNA_phages_VIRSorter_scaffold_6680-gene_5|4955|5974|1019|+|-|-|-|-|HlyD|95.1|5.3e-30(+).p1	bvu:BVU_1673	339	1	339	32	370	0	78.171	**K03585**	Human Diseases	Drug resistance: antimicrobial	acrA, mexA, adeI, smeD, mtrC, cmeA	membrane fusion protein, multidrug efflux system	ko01501,ko01503	beta-Lactam resistance|Cationic antimicrobial peptide (CAMP) resistance	837.99	1482.53	343.17	138.71	157.82	267.19
Lizard04unclassified_dsDNA_phages_VIRSorter_scaffold_6680-gene_7|9212|10564|1352|+|-|-|-|-|OEP|172.8|6.8e-54(+).p1	bcel:BcellWH2_03477	440	1	440	15	454	0	66.364	**K18139**	Cellular Processes	Cellular community - prokaryotes	oprM, emhC, ttgC, cusC, adeK, smeF, mtrE, cmeC,	outer membrane protein, multidrug efflux system	ko01501,ko02024	beta-Lactam resistance|Quorum sensing	235.17	483.38	78.76	20.59	76.97	111.35
Lizard04unclassified_dsDNA_phages_VIRSorter_scaffold_6680-gene_8|10548|11363|815|+|Phage_cluster_3074|67.2|1.3e-	pro:HMPREF0669_00055	244	14	256	11	254	1.54E-65	45.082	**K00677**	Metabolism	Glycan biosynthesis and metabolism	lpxA	UDP-N-acetylglucosamine acyltransferase [EC:2.3.1.129]	ko00540,ko01503	Lipopolysaccharide biosynthesis|Cationic antimicrobial peptide (CAMP) resistance	82.15	150.14	20.68	8.14	3.95	37.69
Lizard04unclassified_dsDNA_phages_VIRSorter_scaffold_1413-gene_2|1905|3278|1373|-|-|-|-|-|OEP|173.5|3.9e-54(-).p1	pdi:BDI_0971	455	1	455	2	454	0	65.275	**K18139**	Cellular Processes	Cellular community - prokaryotes	oprM, emhC, ttgC, cusC, adeK, smeF, mtrE, cmeC,	outer membrane protein, multidrug efflux system	ko01501,ko02024	beta-Lactam resistance|Quorum sensing	511.74	2290.80	81.78	334.47	65.68	435.94
Lizard04unclassified_dsDNA_phages_VIRSorter_scaffold_1413-gene_3|3268|6495|3227|-|-|-|-|-|ACR_tran|973.9|7.9e-296(-).p1	pdi:BDI_0972	1066	1	1066	2	1067	0	82.833	**K03296**	Not Included in Pathway or Brite	Unclassified: signaling and cellular processes	TC.HAE1	hydrophobic/amphiphilic exporter-1 (mainly G- bacteria), HAE1 family	—	—	243.07	2888.07	59.93	345.04	181.86	675.35
Lizard04unclassified_dsDNA_phages_VIRSorter_scaffold_1413-gene_4|6508|7182|674|-|-|-|-|-|HlyD_3|41.2|1.9e-13(-).p1	pdi:BDI_0973	223	1	223	146	368	2.00E-128	80.717	**K03585**	Human Diseases	Drug resistance: antimicrobial	acrA, mexA, adeI, smeD, mtrC, cmeA	membrane fusion protein, multidrug efflux system	ko01501,ko01503	beta-Lactam resistance|Cationic antimicrobial peptide (CAMP) resistance	0.00	123.04	4.79	4.95	1.60	26.69
Lizard04unclassified_dsDNA_phages_VIRSorter_scaffold_244-gene_5|5537|6286|749|+|-|-|-|-|Steroid_dh|138.6|1.5e-43(+).p1	buy:D8S85_03720	243	9	249	1	243	2.80E-122	69.959	**K12343**	Metabolism	Lipid metabolism	SRD5A1	3-oxo-5-alpha-steroid 4-dehydrogenase 1 [EC:1.3.1.22]	ko00140	Steroid hormone biosynthesis	0.00	57.85	4.02	43.96	3.77	2.84
Lizard05Caudovirales_VIRSorter_scaffold_22949-gene_7|6201|6794|593|-|Phage_cluster_898|131.3|1.1e-	abi:Aboo_1416	201	2	189	38	234	2.61E-26	37.313	**K07273**	Not Included in Pathway or Brite	Unclassified: signaling and cellular processes	acm	lysozyme	—	—	0.00	0.00	0.00	0.00	82.39	0.00
Lizard05Caudovirales_VIRSorter_scaffold_1435-gene_1|1|479|478|-|-|-|-|-|DEDD_Tnp_IS110|55.8|9.9e-17(-).p1	pdt:Prede_2238	157	2	158	7	163	2.52E-63	59.236	**K07486**	Not Included in Pathway or Brite	Unclassified: genetic information processing	K07486	transposase	—	—	108318.89	19276.79	13353.81	121707.19	72988.13	16727.57
Lizard05Caudovirales_VIRSorter_scaffold_5649-gene_3|603|2729|2126|-|Phage_cluster_10908|781.6|1.4e-	cmiu:B1H56_09665	294	376	668	56	346	3.13E-68	38.776	**K17680**	Human Diseases	Neurodegenerative disease	PEO1	twinkle protein [EC:3.6.4.12]	ko05017	Spinocerebellar ataxia	119.76	400.83	29.53	943.88	5256.67	5353.12
Lizard05Caudovirales_VIRSorter_scaffold_16929-gene_5|2781|3959|1178|-|Phage_cluster_3763|459.0|2.5e-	ccha:ELD05_12440	387	1	375	6	383	1.33E-115	49.354	**K07496**	Not Included in Pathway or Brite	Unclassified: genetic information processing	K07496	putative transposase	—	—	0.00	22.93	3.20	0.00	1267.27	341.61
Lizard05Caudovirales_VIRSorter_scaffold_16929-gene_12|6456|7187|731|-|Phage_cluster_1089|168.0|6e-	str:Sterm_0817	223	20	238	3	220	9.13E-60	47.085	**K07741**	Not Included in Pathway or Brite	Unclassified: genetic information processing	antB	anti-repressor protein	—	—	0.00	0.00	0.00	0.00	321.40	351.62
Lizard05Caudovirales_VIRSorter_scaffold_11419-gene_5|4181|5335|1154|-|Phage_cluster_289|415.0|7.4e-	mhaz:BHR79_02945	352	1	350	20	371	2.98E-127	51.42	**K07496**	Not Included in Pathway or Brite	Unclassified: genetic information processing	K07496	putative transposase	—	—	0.00	0.00	0.00	0.00	69.83	22.48
Lizard05Caudovirales_VIRSorter_scaffold_6289-gene_1|3|1070|1067|+|Phage_cluster_398|134.9|6.6e-	eyy:EGYY_16610	335	21	355	1	335	1.00E-180	70.448	**K08678**	Metabolism	Carbohydrate metabolism	UXS1, uxs	UDP-glucuronate decarboxylase [EC:4.1.1.35]	ko00520	Amino sugar and nucleotide sugar metabolism	0.00	0.00	0.00	355575.92	93841.89	108893.23
Lizard05Caudovirales_VIRSorter_scaffold_6289-gene_2|1064|2323|1259|+|Phage_cluster_3799|141.9|4.6e-	eyy:EGYY_16600	423	1	419	1	418	5.67E-176	59.338	**K00012**	Metabolism	Carbohydrate metabolism	UGDH, ugd	UDPglucose 6-dehydrogenase [EC:1.1.1.22]	ko00040,ko00053,ko00520	Pentose and glucuronate interconversions|Ascorbate and aldarate metabolism|Amino sugar and nucleotide sugar metabolism	0.00	0.00	0.00	3307.11	705.58	952.53
Lizard05Caudovirales_VIRSorter_scaffold_6289-gene_4|3149|3835|686|-|-|-|-|-|Peptidase_M22|96.3|5.4e-29(-).p1	osp:Odosp_1555	227	1	227	2	228	8.90E-133	76.652	**K14742**	Brite Hierarchies	Protein families: genetic information processing	tsaB	tRNA threonylcarbamoyladenosine biosynthesis protein TsaB	—	—	0.00	0.00	0.00	503.58	105.96	160.14
Lizard05Caudovirales_VIRSorter_scaffold_6289-gene_5|3853|4464|611|-|-|-|-|-|Sua5_yciO_yrdC|155.1|2.8e-47(-).p2	osp:Odosp_1554	190	13	202	1	190	8.54E-118	86.316	**K07566**	Brite Hierarchies	Protein families: genetic information processing	tsaC, rimN, SUA5	L-threonylcarbamoyladenylate synthase [EC:2.7.7.87]	—	—	0.00	0.00	0.00	342.82	73.07	132.16
Lizard05Caudovirales_VIRSorter_scaffold_6289-gene_7|5452|6348|896|-|-|-|-|-|-|-|-|-(-).p1	osp:Odosp_1546	297	1	297	2	298	0	85.859	**K03340**	Metabolism	Amino acid metabolism	dapdh	diaminopimelate dehydrogenase [EC:1.4.1.16]	ko00300	Lysine biosynthesis	0.00	0.00	0.00	376.22	133.87	253.57
Lizard05Caudovirales_VIRSorter_scaffold_6289-gene_8|6379|7197|818|-|-|-|-|-|THDPS_N_2|77.0|2e-23(-).p1	osp:Odosp_1545	271	1	271	2	272	6.67E-168	83.026	**K00674**	Metabolism	Amino acid metabolism	dapD	2,3,4,5-tetrahydropyridine-2,6-dicarboxylate N-succinyltransferase [EC:2.3.1.117]	ko00300	Lysine biosynthesis	0.00	0.00	0.00	263.18	125.86	183.87
Lizard05Caudovirales_VIRSorter_scaffold_10213-gene_4|3398|3988|590|+|Phage_cluster_158|205.6|2.4e-	afl:Aflv_0663	187	1	185	3	189	8.35E-75	59.358	**K06904**	Not Included in Pathway or Brite	Poorly characterized	K06904	uncharacterized protein	—	—	0.00	0.00	0.00	0.00	70.58	0.00
Lizard05Caudovirales_VIRSorter_scaffold_475-gene_5|2785|3783|998|+|Phage_cluster_559|247.2|2.9e-	marm:YQ22_07930	232	90	320	3	223	6.31E-32	34.914	**K00560**	Metabolism	Nucleotide metabolism	thyA, TYMS	thymidylate synthase [EC:2.1.1.45]	ko00240,ko00670,ko01523	Pyrimidine metabolism|One carbon pool by folate|Antifolate resistance	0.00	0.00	0.00	1870.14	138.53	0.00
Lizard05Caudovirales_VIRSorter_scaffold_17667-gene_1|75|1073|998|+|Phage_cluster_593|328.9|8.3e-	bvs:BARVI_04895	312	18	327	32	340	1.27E-90	46.795	**K03553**	Genetic Information Processing	Replication and repair	recA	recombination protein RecA	ko03440	Homologous recombination	0.00	0.00	0.00	0.00	7229.30	767.50
Lizard05Caudovirales_VIRSorter_scaffold_17667-gene_4|1619|2575|956|+|Phage_cluster_54|177.8|6.2e-	mdv:C5Q96_00325	316	1	313	1	270	4.98E-59	36.392	**K00560**	Metabolism	Nucleotide metabolism	thyA, TYMS	thymidylate synthase [EC:2.1.1.45]	ko00240,ko00670,ko01523	Pyrimidine metabolism|One carbon pool by folate|Antifolate resistance	0.00	0.00	0.00	0.00	173.89	22.49
Lizard05Caudovirales_VIRSorter_scaffold_155-gene_1|64|1440|1376|-|-|-|-|-|CCP_MauG|163.7|1e-49(-).p1	pdi:BDI_1048	453	7	457	7	459	0	76.159	**K00428**	Not Included in Pathway or Brite	Unclassified: metabolism	E1.11.1.5	cytochrome c peroxidase [EC:1.11.1.5]	—	—	22002.27	18882.92	4868.58	11130.39	3421.32	5926.91
Lizard05Caudovirales_VIRSorter_scaffold_155-gene_5|3274|3675|401|-|-|-|-|-|LytTR|53.8|4e-16(-).p1	pdi:BDI_0805	70	61	130	185	254	6.02E-32	80	**K07705**	Environmental Information Processing	Signal transduction	lytT, lytR	two-component system, LytTR family, response regulator LytT	ko02020	Two-component system	55.14	219.21	67.98	185.87	111.47	22.54
Lizard05Caudovirales_VIRSorter_scaffold_155-gene_7|4649|5071|422|-|-|-|-|-|HlyD_3|56.7|6.3e-17(-).p3	pdi:BDI_0803	138	2	139	171	308	8.52E-63	65.217	**K15727**	Brite Hierarchies	Protein families: signaling and cellular processes	czcB, cusB, cnrB	membrane fusion protein, heavy metal efflux system	—	—	186.40	638.87	292.03	459.68	358.85	89.64
Lizard05Caudovirales_VIRSorter_scaffold_18848-gene_1|110|2815|2705|-|Phage_cluster_4477|197.5|6.9e-	ceu:A7L45_13615	296	395	682	24	279	5.58E-13	27.703	**K02238**	Brite Hierarchies	Protein families: signaling and cellular processes	comEC	competence protein ComEC	—	—	0.00	0.00	0.00	13277.15	25972.40	0.00
Lizard05Caudovirales_VIRSorter_scaffold_7365-gene_4|2263|2748|485|+|-|-|-|-|RuvC|85.0|1.1e-25(+).p1	dca:Desca_1237	149	1	147	1	148	5.37E-22	36.242	**K01159**	Genetic Information Processing	Replication and repair	ruvC	crossover junction endodeoxyribonuclease RuvC [EC:3.1.22.4]	ko03440	Homologous recombination	0.00	0.00	0.00	0.00	69.11	0.22
Lizard05Caudovirales_VIRSorter_scaffold_23826-gene_4|2198|4798|2600|-|-|-|-|-|tRNA-synt_1_2|215.7|7.6e-66(-).p1	osp:Odosp_2649	865	1	865	38	902	0	89.48	**K01869**	Genetic Information Processing	Translation	LARS, leuS	leucyl-tRNA synthetase [EC:6.1.1.4]	ko00970	Aminoacyl-tRNA biosynthesis	0.00	0.67	0.00	1773.53	256.17	2112.10
Lizard05Caudovirales_VIRSorter_scaffold_21930-gene_5|869|2581|1712|+|Phage_cluster_205|448.2|1.3e-	mec:Q7C_2234	586	7	565	330	897	8.61E-68	31.058	**K02335**	Genetic Information Processing	Replication and repair	polA	DNA polymerase I [EC:2.7.7.7]	ko03030,ko03410,ko03420,ko03440	DNA replication|Base excision repair|Nucleotide excision repair|Homologous recombination	0.00	0.00	0.00	9264.18	647.93	0.00
Lizard05Caudovirales_VIRSorter_scaffold_21930-gene_7|3146|4123|977|+|Phage_cluster_12003|458.6|2.5e-	epo:Epro_0773	233	3	222	7	227	8.81E-07	26.609	**K07465**	Not Included in Pathway or Brite	Unclassified: genetic information processing	K07465	putative RecB family exonuclease	—	—	0.00	0.00	0.00	1278.92	125.94	0.00
Lizard05Caudovirales_VIRSorter_scaffold_6049-gene_3|2338|3825|1487|-	osp:Odosp_0480	494	1	494	2	482	2.36E-151	45.749	**K02478**	Brite Hierarchies	Protein families: metabolism	K02478	two-component system, LytTR family, sensor kinase [EC:2.7.13.3]	—	—	0.00	0.00	3.81	854.33	486.11	2169.54
Lizard05Caudovirales_VIRSorter_scaffold_13162-gene_9|3216|3644|428|-|Phage_cluster_303|88.7|9.2e-	elm:ELI_4246	87	9	95	56	140	1.91E-18	44.828	**K08640**	Brite Hierarchies	Protein families: metabolism	E3.4.17.14	zinc D-Ala-D-Ala carboxypeptidase [EC:3.4.17.14]	—	—	0.00	0.00	0.00	220.39	16.21	0.00
Lizard05Caudovirales_VIRSorter_scaffold_17711-gene_1|231|1421|1190|-|Phage_cluster_3763|359.2|5e-	mev:Metev_2296	392	8	388	10	391	3.42E-88	43.112	**K07496**	Not Included in Pathway or Brite	Unclassified: genetic information processing	K07496	putative transposase	—	—	0.00	0.00	0.00	0.00	1263.86	51.12
Lizard05Caudovirales_VIRSorter_scaffold_2911-gene_1|1|809|808|-|-|-|-|-|Urease_alpha|168.3|1.7e-51(-).p1	afd:Alfi_1061	268	1	268	2	269	1.73E-165	84.701	**K01428**	Metabolism	Nucleotide metabolism	ureC	urease subunit alpha [EC:3.5.1.5]	ko00220,ko00230,ko00791,ko05120	Arginine biosynthesis|Purine metabolism|Atrazine degradation|Epithelial cell signaling in Helicobacter pylori infection	1444251.88	6616388.83	721105.70	2251583.09	1647446.46	3038022.76
Lizard05Caudovirales_VIRSorter_scaffold_2911-gene_2|818|1372|554|-|-|-|-|-|Urease_beta|136.1|7.4e-42(-).p1	pary:A4V02_02710	175	6	180	27	201	6.22E-85	68.571	**K01429**	Metabolism	Nucleotide metabolism	ureB	urease subunit beta [EC:3.5.1.5]	ko00220,ko00230,ko00791	Arginine biosynthesis|Purine metabolism|Atrazine degradation	662.10	2246.00	331.94	835.83	560.86	1014.05
Lizard05Caudovirales_VIRSorter_scaffold_2911-gene_4|1833|2210|377|-|-|-|-|-|CRCB|104.7|6.4e-32(-).p1	osp:Odosp_1237	124	1	124	2	125	2.26E-70	79.839	**K06199**	Brite Hierarchies	Protein families: signaling and cellular processes	crcB, FEX	fluoride exporter	—	—	0.00	197.39	26.71	70.30	68.26	85.35
Lizard05Caudovirales_VIRSorter_scaffold_1965-gene_2|236|874|638|+|Phage_cluster_579|186.5|1.2e-	csh:Closa_0864	176	11	174	8	180	2.60E-40	43.75	**K01447**	Not Included in Pathway or Brite	Unclassified: metabolism	xlyAB	N-acetylmuramoyl-L-alanine amidase [EC:3.5.1.28]	—	—	0.00	1463.98	0.00	0.00	486.00	0.00
Lizard05Caudovirales_VIRSorter_scaffold_12266-gene_2|386|1510|1124|-|Phage_cluster_6140|294.4|2.6e-	blq:L21SP5_03347	257	15	268	180	422	1.87E-08	24.514	**K02314**	Genetic Information Processing	Replication and repair	dnaB	replicative DNA helicase [EC:3.6.4.12]	ko03030,ko04112	DNA replication|Cell cycle - Caulobacter	0.00	0.00	0.00	0.00	1080.51	245.99
Lizard05Caudovirales_VIRSorter_scaffold_700-gene_1|1|699|698|+|-|-|-|-|-|-|-(+).p1	pdi:BDI_2134	227	1	226	370	596	3.82E-123	81.498	**K07316**	Brite Hierarchies	Protein families: signaling and cellular processes	mod	adenine-specific DNA-methyltransferase [EC:2.1.1.72]	—	—	0.00	0.00	0.00	0.00	145976.27	0.00
Lizard05Caudovirales_VIRSorter_scaffold_700-gene_3|1012|2016|1004|+|Phage_cluster_593|323.9|2.7e-	bvs:BARVI_04895	331	4	329	13	340	1.27E-89	45.317	**K03553**	Genetic Information Processing	Replication and repair	recA	recombination protein RecA	ko03440	Homologous recombination	0.00	0.00	0.00	0.00	360.61	35.98

Query ORF ID	Subject gene ID	Alignment length	Query start	Query end	Subject start	Subject end	e-value	Identity(%)	KO	Category	Subcategory	Gene name	Description	Pathway	Pathway definition	Abundance (fpkm)					
																(1) PE-NMC	(2) PE-NQ1	(3) PE-NQ2	(4) PT-SZZ	(5) PT-ZB	(6) PT-RT
Lizard05Caudovirales_VIRSorter_scaffold_2104-gene_1|2|556|554|+|-|-|-|-|LepA_C|165.4|8e-51|(+).p1	osp:Odosp_2553	184	1	184	412	595	1.14E-112	95.652	**K03596**	Human Diseases	Infectious disease: bacterial	lepA	GTP-binding protein LepA	ko05134	Legionellosis	0.00	0.00	0.00	35796.23	15640.31	75274.09
Lizard05Caudovirales_VIRSorter_scaffold_1211-gene_5|776|2163|1387|+|Phage_cluster_38|230.7|5.3e-	bdh:GV66_19085	470	1	461	2	442	6.70E-94	38.298	**K19789**	Brite Hierarchies	Protein families: genetic information processing	radD	DNA repair protein RadD	—	—	0.00	0.00	0.00	9.23	335.92	99.54
Lizard05Caudovirales_VIRSorter_scaffold_8309-gene_2|117|644|527|-|-|-|-|-|-|-|-|-(-).p2	ash:AL1_32680	163	17	173	637	793	1.07E-14	34.356	**K14645**	Cellular Processes	Cellular community - prokaryotes	K14645	serine protease [EC:3.4.21.-]	ko02024	Quorum sensing	0.00	0.00	0.00	703.69	623.83	1219.46
Lizard05Caudovirales_VIRSorter_scaffold_11847-gene_3|886|1344|458|+|Phage_cluster_152|95.8|5.8e-29|1|-|-|-(+).p1	bdh:GV66_19320	112	41	152	2	111	3.17E-18	34.821	**K00986**	Not Included in Pathway or Brite	Unclassified: metabolism	ltrA	RNA-directed DNA polymerase [EC:2.7.7.49]	—	—	0.00	0.00	105.50	0.00	23.54	10.00
Lizard05unclassified_bacterial_viruses_VIRSorter_scaffold_4818-gene_4|2412|3179|767|+|-|-|-|-|Poly_export|57.9|1.2e-18|(+).p1	pola:BXQ17_14005	256	1	255	1	255	8.27E-76	48.438	**K01991**	Environmental Information Processing	Signal transduction	wza, gfcE	polysaccharide biosynthesis/export protein	ko02020,ko02026	Two-component system|Biofilm formation - Escherichia coli	0.00	0.00	0.00	16.32	56.20	129.12
Lizard05unclassified_bacterial_viruses_VIRSorter_scaffold_4818-gene_6|5567|6223|656|+|-|-|-|-|-|-|-|-(+).p1	grl:LPB144_02215	189	2	190	22	209	1.55E-46	40.212	**K01104**	Environmental Information Processing	Signal transduction	E3.1.3.48	protein-tyrosine phosphatase [EC:3.1.3.48]	ko02020	Two-component system	0.00	0.00	0.00	14.79	84.28	89.17
Lizard05unclassified_bacterial_viruses_VIRSorter_scaffold_4818-gene_9|8421|9920|1499|-|-|-|-|-|BATS|64.8|8.9e-21|(-).p2	osp:Odosp_3276	498	1	498	2	499	0	92.57	**K03150**	Metabolism	Metabolism of cofactors and vitamins	thiH	2-iminoacetate synthase [EC:4.1.99.19]	ko00730	Thiamine metabolism	0.00	0.00	0.00	38.26	177.06	212.37
Lizard05unclassified_bacterial_viruses_VIRSorter_scaffold_4818-gene_10|10005|10907|902|-|-|-|-|-|DUF1730|61.3|9.9e-20|(-).p1	buy:D8S85_08410	298	3	298	2	298	2.89E-116	56.04	**K18979**	Brite Hierarchies	Protein families: genetic information processing	queG	epoxyqueuosine reductase [EC:1.17.99.6]	—	—	0.00	0.00	0.00	8.94	67.74	64.42
Lizard05unclassified_bacterial_viruses_VIRSorter_scaffold_4818-gene_12|11232|11690|458|-|Phage_cluster_5579|81.5|8e-	osp:Odosp_3273	141	11	151	1	141	2.77E-84	82.27	**K02426**	Not Included in Pathway or Brite	Poorly characterized	sufE	cysteine desulfuration protein SufE	—	—	0.00	0.00	0.00	6.37	12.07	23.70
Lizard05unclassified_bacterial_viruses_VIRSorter_scaffold_4818-gene_13|11700|12920|1220|-|Phage_cluster_1231|169.5|1.5e-	osp:Odosp_3272	403	1	403	2	404	0	82.382	**K11717**	Metabolism	Metabolism of other amino acids	sufS	cysteine desulfurase / selenocysteine lyase [EC:2.8.1.7 4.4.1.16]	ko00450	Selenocompound metabolism	0.00	0.00	0.00	6.42	34.76	74.64
Lizard05unclassified_bacterial_viruses_VIRSorter_scaffold_4818-gene_14|12934|14298|1364|-|-|-|-|-|UPF0051|255.1|9.9e-79|(-).p1	osp:Odosp_3271	450	2	451	3	452	0	85.111	**K09015**	Not Included in Pathway or Brite	Poorly characterized	sufD	Fe-S cluster assembly protein SufD	—	—	0.00	0.00	0.00	3.60	27.41	51.09
Lizard05unclassified_bacterial_viruses_VIRSorter_scaffold_24943-gene_3|866|1843|977|-|Phage_cluster_1231|114.0|1.1e-	parc:CI960_00830	324	1	324	81	404	0	89.198	**K11717**	Metabolism	Metabolism of other amino acids	sufS	cysteine desulfurase / selenocysteine lyase [EC:2.8.1.7 4.4.1.16]	ko00450	Selenocompound metabolism	0.00	0.00	0.00	37.20	180.60	103.40
Lizard05unclassified_dsDNA_phages_VIRSorter_scaffold_5475-gene_1|1|498|497|+|-|-|-|-|T2SF|74.8|2.8e-24|(+).p1	osp:Odosp_1507	163	3	165	188	350	8.55E-66	66.258	**K02653**	Brite Hierarchies	Protein families: signaling and cellular processes	pilC	type IV pilus assembly protein PilC	—	—	0.00	0.00	0.00	121707.19	312806.29	1478422.41
Lizard05unclassified_dsDNA_phages_VIRSorter_scaffold_5475-gene_5|3681|4658|977|-|-|-|-|-|ACR_tran|286.1|2e-88|(-).p1	osp:Odosp_2536	314	5	318	673	986	0	94.268	**K03296**	Not Included in Pathway or Brite	Unclassified: signaling and cellular processes	TC.HAE1	hydrophobic/amphiphilic exporter-1 (mainly G- bacteria), HAE1 family	—	—	29.43	2.01	1.21	50.57	121.80	438.26
Lizard06Bicaudaviridae_VIRSorter_scaffold_36489-gene_1|3|452|449|+|-|-|-|-|Rnf-Nqr|153.6|1e-47|(+).p1	osp:Odosp_1404	149	1	149	47	195	1.56E-92	91.946	**K03613**	Not Included in Pathway or Brite	Unclassified: metabolism	rnfE	Na+-translocating ferredoxin:NAD+ oxidoreductase subunit E	—	—	0.00	0.00	0.00	2386.42	0.00	655.98
Lizard06Bicaudaviridae_VIRSorter_scaffold_36489-gene_2|526|1098|572|+|-|-|-|-|Rnf-Nqr|225.0|1.4e-69|(+).p1	osp:Odosp_1405	190	1	190	1	190	4.46E-124	95.789	**K03617**	Not Included in Pathway or Brite	Unclassified: metabolism	rnfA	Na+-translocating ferredoxin:NAD+ oxidoreductase subunit A	—	—	0.00	0.00	0.00	0.00	0.00	10.29
Lizard06Bicaudaviridae_VIRSorter_scaffold_36489-gene_4|1817|3220|1403|+|Phage_cluster_1765|242.2|1.6e-	osp:Odosp_1406	467	1	467	1	467	0	85.867	**K00970**	Genetic Information Processing	Folding, sorting and degradation	pcnB	poly(A) polymerase [EC:2.7.7.19]	ko03018	RNA degradation	0.00	0.00	0.00	11.82	11.48	83.94
Lizard06Bicaudaviridae_VIRSorter_scaffold_36489-gene_5|3367|4968|1601|-|-|-|-|-|Sugar_tr|399.3|4.4e-122|(-).p1	mbas:ALGA_4252	523	8	530	13	535	0	63.48	**K08138**	Brite Hierarchies	Protein families: signaling and cellular processes	xylE	MFS transporter, SP family, xylose:H+ symportor	—	—	0.00	0.00	0.00	2.13	3.10	49.68
Lizard06Bicaudaviridae_VIRSorter_scaffold_36489-gene_6|4973|6289|1316|-|-|-|-|-|Pfam-B_393|93.9|1.4e-29|(-).p1	bzg:C4H11_11325	436	1	436	2	437	0	84.862	**K01805**	Metabolism	Carbohydrate metabolism	xylA	xylose isomerase [EC:5.3.1.5]	ko00040,ko00051	Pentose and glucuronate interconversions|Fructose and mannose metabolism	14.52	0.00	0.00	0.00	4.19	8.21
Lizard06Bicaudaviridae_VIRSorter_scaffold_36489-gene_7|6318|7367|1049|-|-|-|-|-|FGGY_N|68.7|1.1e-21|(-).p1	aps:CFPG_513	348	1	348	143	490	5.57E-170	67.529	**K00854**	Metabolism	Carbohydrate metabolism	xylB, XYLB	xylulokinase [EC:2.7.1.17]	ko00040	Pentose and glucuronate interconversions	0.00	0.00	0.00	1.13	0.00	4.52
Lizard06Bicaudaviridae_VIRSorter_scaffold_6938-gene_7|2535|3206|671|+|Phage_cluster_648|124.9|7.5e-	gpa:GPA_31450	139	57	191	51	188	3.70E-42	56.835	**K03465**	Metabolism	Nucleotide metabolism	thyX, thy1	thymidylate synthase (FAD) [EC:2.1.1.148]	ko00240,ko00670	Pyrimidine metabolism|One carbon pool by folate	0.00	0.00	0.00	0.00	0.00	4.08
Lizard06Bicaudaviridae_VIRSorter_scaffold_1876-gene_1|1|545|544|-|-|-|-|-|Amidohydro_4|50.3|7.7e-16|(-).p1	bfs:BF9343_2879	181	1	180	217	396	8.09E-99	78.453	**K01465**	Metabolism	Nucleotide metabolism	URA4, pyrC	dihydroorotase [EC:3.5.2.3]	ko00240	Pyrimidine metabolism	0.00	213527.56	0.00	0.00	0.00	27059.31
Lizard06Bicaudaviridae_VIRSorter_scaffold_1876-gene_2|602|1003|401|-|-|-|-|-|Amidohydro_5|57.3|2.4e-18|(-).p1	bfs:BF9343_2879	132	1	132	2	132	1.27E-72	83.333	**K01465**	Metabolism	Nucleotide metabolism	URA4, pyrC	dihydroorotase [EC:3.5.2.3]	ko00240	Pyrimidine metabolism	59.98	194.59	0.00	11.89	69.28	18.80
Lizard06Bicaudaviridae_VIRSorter_scaffold_1876-gene_3|1037|1789|752|-|Phage_cluster_3811|43.0|6.2e-	bfs:BF9343_2878	245	2	246	3	247	4.41E-169	90.612	**K00721**	Metabolism	Glycan biosynthesis and metabolism	DPM1	dolichol-phosphate mannosyltransferase [EC:2.4.1.83]	ko00510	N-Glycan biosynthesis	0.00	383.22	0.00	17.26	20.11	29.41
Lizard06Bicaudaviridae_VIRSorter_scaffold_1876-gene_4|1905|2605|700|+|-|-|-|-|Pfam-B_9784|84.9|1.1e-26|(+).p1	bfr:BF3127	233	1	233	1	233	2.67E-134	87.983	**K03723**	Genetic Information Processing	Replication and repair	mfd	transcription-repair coupling factor (superfamily II helicase) [EC:3.6.4.-]	ko03420	Nucleotide excision repair	37.91	241.30	0.00	13.15	0.00	21.44
Lizard06Caudovirales_VIRSorter_scaffold_8473-gene_76|48208|48765|557|+|-|-|-|-|Cas_Cas5d|81.6|1.2e-23|(+).p1	lacy:A4V08_13970	210	2	179	5	214	3.82E-66	49.524	**K19119**	Brite Hierarchies	Protein families: signaling and cellular processes	cas5d	CRISPR-associated protein Cas5d	—	—	0.00	0.00	0.00	0.00	0.00	2.80
Lizard06Caudovirales_VIRSorter_scaffold_8473-gene_84|53182|53976|794|-|Phage_cluster_376|292.0|6.4e-	mdv:C5Q96_07025	254	1	253	2	255	1.04E-87	50	**K06223**	Genetic Information Processing	Replication and repair	dam	DNA adenine methylase [EC:2.1.1.72]	ko03430	Mismatch repair	0.00	0.00	0.00	0.00	0.00	12.61
Lizard06Caudovirales_VIRSorter_scaffold_8473-gene_86|54199|55101|902|-|Phage_cluster_1218|165.6|1.9e-	arf:AR1Y2_0409	240	36	273	6	241	8.05E-79	53.75	**K02315**	Brite Hierarchies	Protein families: genetic information processing	dnaC	DNA replication protein DnaC	—	—	0.00	0.00	0.00	0.00	0.00	10.14
Lizard06Caudovirales_VIRSorter_scaffold_8473-gene_89|56148|56561|413|-|Phage_cluster_5230|138.2|2.1e-	rum:CK1_14170	132	2	133	2	133	2.83E-39	50	**K03469**	Genetic Information Processing	Replication and repair	rnhA, RNASEH1	ribonuclease HI [EC:3.1.26.4]	ko03030	DNA replication	0.00	0.00	0.00	0.00	0.00	2.75
Lizard06Caudovirales_VIRSorter_scaffold_8473-gene_94|57791|58402|611|-|Phage_cluster_7884|51.9|7.7e-15|2|-|-|-(-).p1	ova:OBV_01900	51	146	196	295	345	1.08E-12	64.706	**K00558**	Metabolism	Amino acid metabolism	DNMT1, dcm	DNA (cytosine-5)-methyltransferase 1 [EC:2.1.1.37]	ko00270,ko05206	Cysteine and methionine metabolism|MicroRNAs in cancer	0.00	0.00	0.00	0.00	0.00	7.87
Lizard06Caudovirales_VIRSorter_scaffold_8473-gene_96|60068|60790|722|-|Phage_cluster_172|276.6|2.1e-	snb:SP670_0057	224	9	232	8	224	2.48E-80	57.589	**K00571**	Brite Hierarchies	Protein families: signaling and cellular processes	E2.1.1.72	site-specific DNA-methyltransferase (adenine-specific) [EC:2.1.1.72]	—	—	0.60	0.00	0.00	0.00	0.00	5.64
Lizard06Caudovirales_VIRSorter_scaffold_8473-gene_97|60790|62703|1913|-|Phage_cluster_759|396.0|1.3e-	hsm:HSM_0241	239	377	615	39	252	1.88E-95	60.251	**K00571**	Brite Hierarchies	Protein families: signaling and cellular processes	E2.1.1.72	site-specific DNA-methyltransferase (adenine-specific) [EC:2.1.1.72]	—	—	0.59	0.00	0.00	0.00	0.00	7.70
Lizard06Caudovirales_VIRSorter_scaffold_8473-gene_105|65908|66339|431|-|Phage_cluster_9|141.8|2.5e-	cbol:CGC65_03165	153	1	142	2	154	2.52E-59	59.477	**K03111**	Genetic Information Processing	Replication and repair	ssb	single-strand DNA-binding protein	ko03030,ko03430,ko03440	DNA replication|Mismatch repair|Homologous recombination	0.00	0.00	0.00	0.00	0.00	4.34
Lizard06Caudovirales_VIRSorter_scaffold_8473-gene_130|78835|79746|911|-|Phage_cluster_519|158.0|4.7e-	cbi:CLJ_B2576	116	185	300	161	276	2.11E-56	75	**K07741**	Not Included in Pathway or Brite	Unclassified: genetic information processing	antB	anti-repressor protein	—	—	0.00	0.00	0.00	0.00	0.00	4.34
Lizard06Caudovirales_VIRSorter_scaffold_8473-gene_134|80645|81070|425|-|Phage_cluster_12|44.5|2.3e-	sae:NWMN_0268	140	1	140	11	149	4.75E-40	47.143	**K07741**	Not Included in Pathway or Brite	Unclassified: genetic information processing	antB	anti-repressor protein	—	—	0.00	0.00	0.00	0.00	0.00	2.53
Lizard06Caudovirales_VIRSorter_scaffold_8473-gene_139|83030|84271|1241|-|Phage_cluster_3797|528.6|1.7e-	cbol:CGC65_09015	411	1	411	2	412	0	88.808	**K07493**	Not Included in Pathway or Brite	Unclassified: genetic information processing	K07493	putative transposase	—	—	0.00	0.00	0.00	0.00	0.00	10.22
Lizard06Caudovirales_VIRSorter_scaffold_8473-gene_141|84671|85606|935|-	byl:A4V09_04265	308	3	308	4	310	4.08E-130	58.766	**K01971**	Genetic Information Processing	Replication and repair	ligD	bifunctional non-homologous end joining protein LigD [EC:6.5.1.1]	ko03450	Non-homologous end-joining	0.00	0.00	0.00	0.00	0.00	1.85
Lizard06Caudovirales_VIRSorter_scaffold_8473-gene_142|85603|86415|812|-|Phage_cluster_14425|158.2|4.1e-	byl:A4V09_04270	265	2	264	2	266	1.81E-136	68.679	**K10979**	Genetic Information Processing	Replication and repair	ku	DNA end-binding protein Ku	ko03450	Non-homologous end-joining	0.00	0.00	0.00	0.00	0.00	4.62
Lizard06Caudovirales_VIRSorter_scaffold_8473-gene_143|87035|87886|851|-|Phage_cluster_415|172.2|2.5e-	cbol:CGC65_06465	279	1	279	2	280	1.13E-179	86.738	**K04763**	Brite Hierarchies	Protein families: genetic information processing	xerD	integrase/recombinase XerD	—	—	0.00	0.00	0.00	0.00	0.00	3.32
Lizard06Caudovirales_VIRSorter_scaffold_40011-gene_4|3361|3942|581|+|Phage_cluster_158|215.1|2.3e-	afl:Aflv_0663	188	4	189	6	193	5.31E-83	63.298	**K06904**	Not Included in Pathway or Brite	Poorly characterized	K06904	uncharacterized protein	—	—	0.00	4.85	11.92	0.00	0.00	7.03
Lizard06Caudovirales_VIRSorter_scaffold_40011-gene_22|19262|20227|965|+|Phage_cluster_415|288.3|1.2e-	cld:CLSPO_c24690	317	10	319	17	330	1.20E-91	44.479	**K04763**	Brite Hierarchies	Protein families: genetic information processing	xerD	integrase/recombinase XerD	—	—	0.00	0.00	0.46	0.00	0.00	0.49
Lizard06Caudovirales_VIRSorter_scaffold_40011-gene_28|23936|24988|1052|-|Phage_cluster_8947|297.0|2.2e-	ibu:IB211_01356	349	3	349	32	346	5.81E-123	54.728	**K02238**	Brite Hierarchies	Protein families: signaling and cellular processes	comEC	competence protein ComEC	—	—	0.00	0.06	1.30	0.00	0.00	0.80
Lizard06Caudovirales_VIRSorter_scaffold_20281-gene_2|1192|2694|1502|-|-|-|-|-|GTP_EFTU|134.4|6e-40|(-).p2	pdi:BDI_2510	499	1	499	28	526	0	91.984	**K02837**	Brite Hierarchies	Protein families: genetic information processing	prfC	peptide chain release factor 3	—	—	0.00	6.22	0.00	5117.18	1679.50	783.38
Lizard06Caudovirales_VIRSorter_scaffold_20281-gene_3|2862|3722|860|-	pdi:BDI_2511	283	1	283	2	284	4.81E-147	69.965	**K00067**	Metabolism	Glycan biosynthesis and metabolism	rfbD, rmlD	dTDP-4-dehydrorhamnose reductase [EC:1.1.1.133]	ko00521,ko00523	Streptomycin biosynthesis|Polyketide sugar unit biosynthesis	0.00	2.07	3.11	822.99	265.95	192.36
Lizard06Caudovirales_VIRSorter_scaffold_20281-gene_5|4272|4880|608|-|-|-|-|-|-|-|-|-(-).p1	parc:CI960_18815	201	1	201	108	308	1.73E-117	79.104	**K02558**	Not Included in Pathway or Brite	Unclassified: metabolism	mpl	UDP-N-acetylmuramate: L-alanyl-gamma-D-glutamyl-meso-diaminopimelate ligase [EC:6.3.2.45]	—	—	0.00	0.69	0.00	271.49	82.99	38.47
Lizard06Caudovirales_VIRSorter_scaffold_20281-gene_7|6351|8531|2180|+|-|-|-|-|Glyco_hydro_20|410.8|9.4e-124|(+).p1	parc:CI960_18855	725	1	725	48	772	0	75.172	**K12373**	Metabolism	Carbohydrate metabolism	HEXA_B	hexosaminidase [EC:3.2.1.52]	ko00511,ko00513,ko00520,ko00531,ko00603,ko00604,ko04142	Other glycan degradation|Various types of N-glycan biosynthesis|Amino sugar and nucleotide sugar metabolism|Glycosaminoglycan degradation|Glycosphingolipid biosynthesis - globo and isoglobo series|Glycosphingolipid biosynthesis - ganglio series|Lysosome	11.37	3.04	0.00	620.56	215.07	153.07
Lizard06Caudovirales_VIRSorter_scaffold_60647-gene_3|457|1545|1088|+|Phage_cluster_396|296.7|2.9e-	rch:RUM_20080	373	30	356	1	359	1.10E-69	39.142	**K17398**	Metabolism	Amino acid metabolism	DNMT3A	DNA (cytosine-5)-methyltransferase 3A [EC:2.1.1.37]	ko00270,ko05206	Cysteine and methionine metabolism|MicroRNAs in cancer	0.00	0.00	0.00	0.00	0.00	322.97
Lizard06Caudovirales_VIRSorter_scaffold_60647-gene_21|8690|9883|1193|+|Phage_cluster_338|443.0|2e-	pes:SOPEG_0600	401	1	393	1	401	3.92E-157	54.364	**K06909**	Not Included in Pathway or Brite	Unclassified: genetic information processing	xtmB	phage terminase large subunit	—	—	0.00	0.00	0.00	0.00	0.00	9.51
Lizard06Caudovirales_VIRSorter_scaffold_60647-gene_22|10057|11445|1388|+|Phage_cluster_6297|366.7|3.3e-	sfg:AV650_15545	452	1	451	42	486	6.09E-141	48.451	**K09961**	Not Included in Pathway or Brite	Poorly characterized	K09961	uncharacterized protein	—	—	0.00	0.00	0.00	0.00	0.00	20.74
Lizard06Caudovirales_VIRSorter_scaffold_60647-gene_24|12236|13633|1397|+|Phage_cluster_3115|278.5|2.5e-	pfac:PFJ30894_00722	475	6	430	8	476	4.83E-111	45.053	**K09960**	Not Included in Pathway or Brite	Poorly characterized	K09960	uncharacterized protein	—	—	0.00	0.00	0.00	0.00	0.00	21.11
Lizard06Caudovirales_VIRSorter_scaffold_924-gene_30|22811|23488|677|-|Phage_cluster_3343|57.4|2.4e-16|1|-|-|-(-).p2	pow:IJ21_39200	111	108	218	1183	1293	3.02E-12	42.342	**K21449**	Brite Hierarchies	Protein families: signaling and cellular processes	ata, sadA, emaA	trimeric autotransporter adhesin	—	—	0.00	0.00	0.00	0.00	2.29	1.94
Lizard06Caudovirales_VIRSorter_scaffold_5589-gene_24|18912|19244|332|-|Phage_cluster_6687|88.8|6.3e-26|1|-|-|-(-).p1	lacy:A4V08_16015	109	1	109	219	327	3.58E-54	76.147	**K07481**	Not Included in Pathway or Brite	Unclassified: genetic information processing	K07481	transposase, IS5 family	—	—	0.00	1.65	0.24	4.16	0.00	0.68
Lizard06Caudovirales_VIRSorter_scaffold_18345-gene_23|14638|15183|545|-|Phage_cluster_158|202.5|1.5e-	afl:Aflv_0663	173	7	177	23	194	1.91E-70	61.272	**K06904**	Not Included in Pathway or Brite	Poorly characterized	K06904	uncharacterized protein	—	—	0.00	0.00	0.00	0.49	1.42	1.75
Lizard06Caudovirales_VIRSorter_scaffold_2674-gene_4|6064|7569|1505|+|-|-|-|-|SusD|187.8|5.1e-56|(+).p1	bcel:BcellWH2_05310	496	6	501	1	496	0	85.282	**K21572**	Brite Hierarchies	Protein families: signaling and cellular processes	susD	starch-binding outer membrane protein, SusD/RagB family	—	—	0.00	3.18	0.73	3.54	3.44	41.22
Lizard06Caudovirales_VIRSorter_scaffold_2674-gene_5|7667|9085|1418|+|-|-|-|-|SusE|74.9|9.1e-22|(+).p1	bcel:BcellWH2_05311	472	1	472	27	498	0	81.78	**K21571**	Not Included in Pathway or Brite	Unclassified: signaling and cellular processes	susE_F	starch-binding outer membrane protein SusE/F	—	—	14.13	1.74	0.00	0.93	0.00	19.12
Lizard06Caudovirales_VIRSorter_scaffold_2674-gene_6|9294|11108|1814|+|-|-|-|-|Glyco_hydro_66|660.3|4e-199|(+).p1	bcel:BcellWH2_05312	603	1	602	1	596	0	79.602	**K05988**	Metabolism	Carbohydrate metabolism	dexA	dextranase [EC:3.2.1.11]	ko00500	Starch and sucrose metabolism	0.00	1.91	2.87	3.08	0.00	39.44
Lizard06Caudovirales_VIRSorter_scaffold_2674-gene_7|11668|12297|629|+|-|-|-|-|Gal_mutarotas_2|87.2|9.3e-26|(+).p1	bcel:BcellWH2_05313	201	1	201	37	237	3.78E-130	90.547	**K01187**	Metabolism	Carbohydrate metabolism	malZ	alpha-glucosidase [EC:3.2.1.20]	ko00052,ko00500	Galactose metabolism|Starch and sucrose metabolism	9.28	0.64	0.76	4.60	1.79	8.73
Lizard06Caudovirales_VIRSorter_scaffold_2674-gene_8|12266|13864|1598|+|-|-|-|-|Glyco_hydro_31|397.1|1.8e-119|(+).p1	bcel:BcellWH2_05313	531	2	532	302	832	0	84.934	**K01187**	Metabolism	Carbohydrate metabolism	malZ	alpha-glucosidase [EC:3.2.1.20]	ko00052,ko00500	Galactose metabolism|Starch and sucrose metabolism	0.00	1.45	1.81	2.04	1.70	27.88

Query ORF ID	Subject gene ID	Alignment length	Query start	Query end	Subject start	Subject end	e-value	Identity(%)	KO	Category	Subcategory	Gene name	Description	Pathway	Pathway definition	Abundance (fpkm) (1) PE-NMC	(2) PE-NQ1	(3) PE-NQ2	(4) PT-SZZ	(5) PT-ZB	(6) PT-RT
Lizard06Caudovirales_VIRSorter_scaffold_2674-gene_9|14070|16217|2147|+|-|-|-|-|Glyco_hydro_97|343.0|1.8e-103(+).p1	bcel:BcellWH2_05314	714	1	714	1	714	0	86.835	**K21574**	Metabolism	Carbohydrate metabolism	susB	glucan 1,4-alpha-glucosidase [EC:3.2.1.3]	ko00500	Starch and sucrose metabolism	10.26	4.53	1.90	4.33	8.15	68.19
Lizard06Caudovirales_VIRSorter_scaffold_2674-gene_11|17921|18631|710|-|Phage_cluster_10848|75.6|5.7e-22|2|AMP-	bcac:CGC64_12480	235	1	235	320	554	4.21E-153	87.234	**K01897**	Metabolism	Lipid metabolism	ACSL, fadD	long-chain acyl-CoA synthetase [EC:6.2.1.3]	ko00061,ko00071,ko02024,ko03320,ko04146,ko04216,ko04714,ko04920	Fatty acid biosynthesis|Fatty acid degradation|Quorum sensing|PPAR signaling pathway|Peroxisome|Ferroptosis|Thermogenesis|Adipocytokine signaling pathway	0.00	0.74	0.99	0.00	0.00	13.70
Lizard06Caudovirales_VIRSorter_scaffold_5080-gene_5|4111|5616|1505|+|-|-|-|-|Glyco_hydro_31|211.0|5.3e-63(+).p1	bfs:BF9343_1267	499	2	500	54	549	0	67.535	**K01187**	Metabolism	Carbohydrate metabolism	malZ	alpha-glucosidase [EC:3.2.1.20]	ko00052,ko00500	Galactose metabolism|Starch and sucrose metabolism	0.00	2.16	0.00	0.00	5.07	18.43
Lizard06Caudovirales_VIRSorter_scaffold_5080-gene_6|6108|7226|1118|+|-|-|-|-|GFO_IDH_MocA|60.9|3e-17(+).p1	pdi:BDI_1154	372	1	372	47	418	0	91.667	**K00010**	Metabolism	Carbohydrate metabolism	iolG	myo-inositol 2-dehydrogenase / D-chiro-inositol 1-dehydrogenase [EC:1.1.1.18 1.1.1.369]	ko00521,ko00562	Streptomycin biosynthesis|Inositol phosphate metabolism	0.00	2.67	0.00	0.00	1.71	42.77
Lizard06Caudovirales_VIRSorter_scaffold_5080-gene_9|11053|12360|1307|-|-|-|-|-|ABC2_membrane_2|99.5|2.9e-29(-).p1	spon:HME9304_03030	401	1	374	3	385	1.42E-10	23.441	**K01992**	Brite Hierarchies	Protein families: signaling and cellular processes	ABC-2.P	ABC-2 type transport system permease protein	—	—	0.00	0.94	0.00	0.00	40.56	269.03
Lizard06Caudovirales_VIRSorter_scaffold_5080-gene_10|12382|13917|1535|-|-|-|-|-|ABC2_membrane_2|119.3|2.7e-35	marf:CJ739_1593	452	18	443	28	439	3.20E-15	24.336	**K01992**	Brite Hierarchies	Protein families: signaling and cellular processes	ABC-2.P	ABC-2 type transport system permease protein	—	—	0.00	0.60	0.00	0.00	14.32	123.77
Lizard06Caudovirales_VIRSorter_scaffold_5080-gene_11|13944|14666|722|-|Phage_cluster_405|128.5|5.1e-	smon:AWR27_00030	230	1	229	2	230	3.35E-85	56.087	**K01990**	Brite Hierarchies	Protein families: signaling and cellular processes	ABC-2.A	ABC-2 type transport system ATP-binding protein	—	—	0.00	0.21	0.00	0.00	4.49	13.76
Lizard06Caudovirales_VIRSorter_scaffold_5080-gene_13|16605|18113|1508|-|-|-|-|-|OEP|118.0|6.9e-35(-).p1	pet:PEIBARAKI_4846	493	3	485	15	491	6.53E-69	30.832	**K12340**	Environmental Information Processing	Membrane transport	tolC	outer membrane protein	ko01501,ko01503,ko02020,ko03070,ko04626,ko05133	beta-Lactam resistance|Cationic antimicrobial peptide (CAMP) resistance|Two-component system|Bacterial secretion system|Plant-pathogen interaction|Pertussis	0.00	0.54	0.00	0.00	0.00	4.47
Lizard06Caudovirales_VIRSorter_scaffold_15940-gene_9|14426|15016|590|+|Phage_cluster_629|184.7|3.6e-	btho:Btheta7330_03644	191	1	191	1	191	5.73E-111	76.963	**K01448**	Human Diseases	Drug resistance: antimicrobial	amiABC	N-acetylmuramoyl-L-alanine amidase [EC:3.5.1.28]	ko01503	Cationic antimicrobial peptide (CAMP) resistance	0.00	0.00	0.00	0.00	0.00	0.48
Lizard06Caudovirales_VIRSorter_scaffold_576-gene_23|8857|10794|1937|+|Phage_cluster_503|285.5|1.2e-85|1|Pfam-	cbz:Cbs_1619	693	1	645	1	655	2.14E-37	25.397	**K03546**	Brite Hierarchies	Protein families: genetic information processing	sbcC, rad50	DNA repair protein SbcC/Rad50	—	—	0.00	0.00	0.00	1.21	6823.32	123.37
Lizard06Caudovirales_VIRSorter_scaffold_576-gene_28|12545|13402|857|+|Phage_cluster_2094|194.5|3.4e-	bpus:UP12_14440	296	1	283	1	292	4.93E-43	33.446	**K07455**	Brite Hierarchies	Protein families: genetic information processing	recT	recombination protein RecT	—	—	0.00	0.24	0.00	0.57	1186.89	24.75
Lizard06Caudovirales_VIRSorter_scaffold_61107-gene_15|15688|16314|626|+|Phage_cluster_4904|136.1|2e-	osp:Odosp_0573	208	1	208	1	208	2.39E-126	84.135	**K09125**	Brite Hierarchies	Protein families: signaling and cellular processes	K09125	uncharacterized protein	—	—	0.00	0.00	0.00	0.68	1.33	0.22
Lizard06Caudovirales_VIRSorter_scaffold_107610-gene_1|1|580|579|-|-|-|-|-|POR|118.2|6.6e-35(-).p1	tjr:TherJR_1676	177	5	181	5	178	1.53E-47	41.243	**K00177**	Metabolism	Carbohydrate metabolism	korC, oorC	2-oxoglutarate ferredoxin oxidoreductase subunit gamma [EC:1.2.7.3]	ko00020,ko00720	Citrate cycle (TCA cycle)|Carbon fixation pathways in prokaryotes	0.00	0.00	0.00	0.00	0.00	5411.86
Lizard06Caudovirales_VIRSorter_scaffold_107610-gene_2|580|1338|758|-|-|-|-|-|TPP_enzyme_C|73.9|2e-21(-).p1	mbf:BHV42_06960	245	2	246	3	247	4.32E-93	51.837	**K00175**	Metabolism	Carbohydrate metabolism	korB, oorB, oforB	2-oxoglutarate/2-oxoacid ferredoxin oxidoreductase subunit beta [EC:1.2.7.3 1.2.7.11]	ko00010,ko00020,ko00620,ko00650,ko00720	Glycolysis / Gluconeogenesis|Citrate cycle (TCA cycle)|Pyruvate metabolism|Butanoate metabolism|Carbon fixation pathways in prokaryotes	0.00	0.00	0.00	0.00	0.00	34.78
Lizard06Caudovirales_VIRSorter_scaffold_107610-gene_3|1338|2432|1094|-|-|-|-|-|POR_N|180.8|5.3e-54(-).p1	fia:NA23_02050	346	5	348	3	348	1.06E-137	54.913	**K00174**	Metabolism	Carbohydrate metabolism	korA, oorA, oforA	2-oxoglutarate/2-oxoacid ferredoxin oxidoreductase subunit alpha [EC:1.2.7.3 1.2.7.11]	ko00010,ko00020,ko00620,ko00650,ko00720	Glycolysis / Gluconeogenesis|Citrate cycle (TCA cycle)|Pyruvate metabolism|Butanoate metabolism|Carbon fixation pathways in prokaryotes	0.00	0.00	0.00	0.00	0.00	53.32
Lizard06Caudovirales_VIRSorter_scaffold_107610-gene_8|7778|8368|590|-|-|-|-|-|CoaE|179.2|9.4e-54(-).p1	mbf:BHV42_02095	193	1	193	2	190	1.46E-48	43.005	**K00859**	Metabolism	Metabolism of cofactors and vitamins	coaE	dephospho-CoA kinase [EC:2.7.1.24]	ko00770	Pantothenate and CoA biosynthesis	0.00	0.00	0.00	0.00	0.00	1.08
Lizard06Caudovirales_VIRSorter_scaffold_107610-gene_9|8394|9830|1436|-|-|-|-|-|tRNA-synt_1e|399.5|1.6e-120(-).p1	mbf:BHV42_04605	477	1	477	1	457	0	68.134	**K01883**	Genetic Information Processing	Translation	CARS, cysS	cysteinyl-tRNA synthetase [EC:6.1.1.16]	ko00970	Aminoacyl-tRNA biosynthesis	0.00	0.00	0.00	0.00	0.00	4.28
Lizard06Caudovirales_VIRSorter_scaffold_107610-gene_12|12680|14065|1385|-|-|-|-|-|tRNA_U5-meth_tr|75.1|7.6e-22(-).p1	mbf:BHV42_01265	465	17	455	12	472	5.98E-149	46.667	**K03215**	Brite Hierarchies	Protein families: genetic information processing	rumA	23S rRNA (uracil1939-C5)-methyltransferase [EC:2.1.1.190]	—	—	0.00	0.00	0.00	0.00	0.00	2.99
Lizard06Caudovirales_VIRSorter_scaffold_107610-gene_13|14067|15293|1226|-|Phage_cluster_8323|62.4|7.9e-	mbf:BHV42_03375	392	7	396	1	392	7.78E-176	59.694	**K19003**	Metabolism	Lipid metabolism	mgdA	1,2-diacylglycerol 3-beta-glucosyltransferase [EC:2.4.1.336]	ko00561	Glycerolipid metabolism	0.00	0.00	0.00	0.00	0.00	2.34
Lizard06Caudovirales_VIRSorter_scaffold_23214-gene_5|3152|3745|593|+|Phage_cluster_158|187.9|4.5e-	bbe:BBR47_53780	168	22	189	28	192	3.79E-44	47.619	**K06904**	Not Included in Pathway or Brite	Poorly characterized	K06904	uncharacterized protein	—	—	0.00	0.00	0.00	0.00	6.62	11.71
Lizard06Caudovirales_VIRSorter_scaffold_48869-gene_6|4335|5483|1148|+|Phage_cluster_559|269.2|4.2e-	nen:NCHU2750_57570	243	138	378	4	237	1.17E-33	35.802	**K00560**	Metabolism	Nucleotide metabolism	thyA, TYMS	thymidylate synthase [EC:2.1.1.45]	ko00240,ko00670,ko01523	Pyrimidine metabolism|One carbon pool by folate|Antifolate resistance	0.00	0.00	0.00	0.00	0.00	6.81
Lizard06Caudovirales_VIRSorter_scaffold_48869-gene_23|13384|13995|611|-|Phage_cluster_303|92.5|4.7e-	elm:ELI_0090	152	1	152	1	148	8.84E-27	35.526	**K08640**	Brite Hierarchies	Protein families: metabolism	E3.4.17.14	zinc D-Ala-D-Ala carboxypeptidase [EC:3.4.17.14]	—	—	0.00	0.00	0.00	0.00	0.00	0.74
Lizard06Caudovirales_VIRSorter_scaffold_37464-gene_2|3316|4755|1439|+|-|-|-|-|SusD-like_3|101.8|9.2e-30(+).p1	osp:Odosp_3533	479	1	479	1	479	0	64.927	**K21572**	Brite Hierarchies	Protein families: signaling and cellular processes	susD	starch-binding outer membrane protein, SusD/RagB family	—	—	0.00	0.00	0.00	6.48	9.43	20.77
Lizard06Caudovirales_VIRSorter_scaffold_37464-gene_7|8936|11137|2201|-|-|-|-|-|Glyco_hydro_35|397.9|6.8e-120(-).p1	boa:Bovatus_03773	733	1	732	46	778	0	81.583	**K12308**	Metabolism	Carbohydrate metabolism	bgaB, lacA	beta-galactosidase [EC:3.2.1.23]	ko00052	Galactose metabolism	0.00	0.00	0.00	4.01	2.33	22.63
Lizard06Caudovirales_VIRSorter_scaffold_37464-gene_9|12622|13299|677|+|Phage_cluster_989|215.9|7.7e-	osp:Odosp_2071	225	1	225	1	225	1.14E-158	92.889	**K01358**	Cellular Processes	Cell growth and death	clpP, CLPP	ATP-dependent Clp protease, protease subunit [EC:3.4.21.92]	ko04112,ko04212	Cell cycle - Caulobacter|Longevity regulating pathway - worm	0.00	0.00	0.00	1.13	0.00	0.47
Lizard06Caudovirales_VIRSorter_scaffold_37464-gene_10|13306|14520|1214|+|Phage_cluster_5496|342.7|3e-	osp:Odosp_2072	404	1	404	1	404	0	95.297	**K03544**	Cellular Processes	Cell growth and death	clpX, CLPX	ATP-dependent Clp protease ATP-binding subunit ClpX	ko04112	Cell cycle - Caulobacter	0.00	0.00	0.00	0.00	0.00	1.48
Lizard06Caudovirales_VIRSorter_scaffold_15095-gene_6|6549|7316|767|+|Phage_cluster_3510|91.3|1e-	mbu:Mbur_1605	253	1	253	1	253	4.36E-117	63.241	**K00978**	Metabolism	Carbohydrate metabolism	rfbF	glucose-1-phosphate cytidylyltransferase [EC:2.7.7.33]	ko00500,ko00520	Starch and sucrose metabolism|Amino sugar and nucleotide sugar metabolism	0.00	0.00	0.00	55.75	1.59	23.89
Lizard06Caudovirales_VIRSorter_scaffold_15095-gene_7|7317|8144|827|-|-|-|-|-|LicD|147.6|9.8e-44(-).p1	boa:Bovatus_03501	268	1	268	2	269	1.37E-138	66.045	**K07271**	Not Included in Pathway or Brite	Unclassified: metabolism	licD	lipopolysaccharide cholinephosphotransferase [EC:2.7.8.-]	—	—	0.00	0.00	0.00	82.68	25.65	29.65
Lizard06Caudovirales_VIRSorter_scaffold_15095-gene_8|8269|9363|1094|+|Phage_cluster_398|135.1|4.2e-	tar:TALC_00085	351	6	356	6	356	0	67.521	**K08678**	Metabolism	Carbohydrate metabolism	UXS1, uxs	UDP-glucuronate decarboxylase [EC:4.1.1.35]	ko00520	Amino sugar and nucleotide sugar metabolism	0.00	0.00	0.00	131.17	34.05	40.28
Lizard06Caudovirales_VIRSorter_scaffold_15095-gene_9|9357|10616|1259|+|Phage_cluster_3799|139.7|1.7e-	eyy:EGYY_16600	423	1	419	1	418	2.73E-175	58.865	**K00012**	Metabolism	Carbohydrate metabolism	UGDH, ugd	UDPglucose 6-dehydrogenase [EC:1.1.1.22]	ko00040,ko00053,ko00520	Pentose and glucuronate interconversions|Ascorbate and aldarate metabolism|Amino sugar and nucleotide sugar metabolism	0.00	0.00	0.00	369.55	80.23	110.15
Lizard06Caudovirales_VIRSorter_scaffold_15095-gene_11|11441|12127|686|-|-|-|-|-|Peptidase_M22|96.4|3.7e-28(-).p1	osp:Odosp_1555	227	1	227	2	228	7.00E-135	78.414	**K14742**	Brite Hierarchies	Protein families: genetic information processing	tsaB	tRNA threonylcarbamoyladenosine biosynthesis protein TsaB	—	—	0.00	0.00	0.00	134.22	28.25	45.15
Lizard06Caudovirales_VIRSorter_scaffold_15095-gene_12|12145|12756|611|-|-|-|-|-|Sua5_yciO_yrdC|152.9|1e-45(-).p1	osp:Odosp_1554	190	13	202	1	190	4.01E-117	85.789	**K07566**	Brite Hierarchies	Protein families: genetic information processing	tsaC, rimN, SUA5	L-threonylcarbamoyladenylate synthase [EC:2.7.7.87]	—	—	0.00	0.00	0.00	102.27	23.18	43.18
Lizard06Caudovirales_VIRSorter_scaffold_23275-gene_4|2884|6720|3836|-|Phage_cluster_5160|54.8|6.8e-	mbf:BHV42_03520	1272	7	1274	7	1278	0	76.336	**K03046**	Genetic Information Processing	Transcription	rpoC	DNA-directed RNA polymerase subunit beta' [EC:2.7.7.6]	ko03020	RNA polymerase	25.04	1.03	6.17	0.00	14.46	53.05
Lizard06Caudovirales_VIRSorter_scaffold_23275-gene_5|6782|8500|1718|-|Phage_cluster_3301|103.4|1.5e-	mbf:BHV42_03525	570	1	570	2	569	0	88.772	**K03046**	Genetic Information Processing	Transcription	rpoC	DNA-directed RNA polymerase subunit beta' [EC:2.7.7.6]	ko03020	RNA polymerase	0.00	0.00	0.66	0.00	1.54	8.71
Lizard06Caudovirales_VIRSorter_scaffold_23275-gene_6|8520|11837|3317|-|Phage_cluster_7519|63.3|1.9e-	mbf:BHV42_03530	1102	1	1102	2	1102	0	89.564	**K03043**	Genetic Information Processing	Transcription	rpoB	DNA-directed RNA polymerase subunit beta [EC:2.7.7.6]	ko03020	RNA polymerase	12.71	0.00	6.79	0.42	0.00	14.72
Lizard06Caudovirales_VIRSorter_scaffold_23275-gene_8|12509|13327|818|-|-|-|-|-|Flagellin_N|52.6|7.5e-15(-).p1	hhh:CLM76_06630	260	20	271	20	271	9.91E-32	31.538	**K02406**	Environmental Information Processing	Signal transduction	fliC	flagellin	ko02020,ko02040,ko04621,ko04626,ko05131,ko05132,ko05134	Two-component system|Flagellar assembly|NOD-like receptor signaling pathway|Plant-pathogen interaction|Shigellosis|Salmonella infection|Legionellosis	0.00	0.00	0.00	0.00	0.00	1.34
Lizard06Caudovirales_VIRSorter_scaffold_33331-gene_1|54|740|686|+|Phage_cluster_405|175.7|2.6e-	bcel:BcellWH2_00320	225	1	225	1	225	3.22E-160	96	**K02003**	Brite Hierarchies	Protein families: signaling and cellular processes	ABC.CD.A	putative ABC transport system ATP-binding protein	—	—	0.00	54.92	0.00	0.00	579.27	11507.04
Lizard06Caudovirales_VIRSorter_scaffold_33331-gene_2|914|3277|2363|+|-|-|-|-|MacB_PCD|93.5|2.8e-27(+).p1	bcel:BcellWH2_00318	787	1	787	1	775	0	59.593	**K02004**	Brite Hierarchies	Protein families: signaling and cellular processes	ABC.CD.P	putative ABC transport system permease protein	—	—	0.00	0.00	0.00	0.00	102.67	4909.65
Lizard06Caudovirales_VIRSorter_scaffold_33331-gene_3|3471|5789|2318|+|-|-|-|-|FtsX|95.7|2.9e-28(+).p1	bcac:CGC64_12545	776	1	772	1	774	0	66.495	**K02004**	Brite Hierarchies	Protein families: signaling and cellular processes	ABC.CD.P	putative ABC transport system permease protein	—	—	0.00	13.67	11.54	8.25	33.04	1472.28
Lizard06Caudovirales_VIRSorter_scaffold_33331-gene_9|10975|11712|737|+|-|-|-|-|Pantoate_transf|338.7|3.3e-102(+).p1	bhl:Bache_2185	245	1	245	28	272	8.20E-171	93.878	**K00606**	Metabolism	Metabolism of cofactors and vitamins	panB	3-methyl-2-oxobutanoate hydroxymethyltransferase [EC:2.1.2.11]	ko00770	Pantothenate and CoA biosynthesis	0.00	0.41	0.00	0.00	0.00	48.82
Lizard06Caudovirales_VIRSorter_scaffold_33331-gene_11|12758|13111|353|-|-|-|-|-|DAGK_prokar|125.5|1.1e-37(-).p1	bhl:Bache_2187	114	1	114	2	115	1.36E-59	71.93	**K00901**	Metabolism	Lipid metabolism	dgkA, DGK	diacylglycerol kinase (ATP) [EC:2.7.1.107]	ko00561,ko00564,ko04070,ko04072,ko04361,ko05231	Glycerolipid metabolism|Glycerophospholipid metabolism|Phosphatidylinositol signaling system|Phospholipase D signaling pathway|Axon regeneration|Choline	0.00	0.12	0.00	0.00	0.00	1.23
Lizard06Caudovirales_VIRSorter_scaffold_14641-gene_7|6154|7362|1208|-|Phage_cluster_289|439.4|2.1e-	mhaz:BHR79_02945	370	1	368	2	371	5.18E-137	51.622	**K07496**	Not Included in Pathway or Brite	Unclassified: genetic information processing	K07496	putative transposase	—	—	0.00	0.00	0.00	0.00	47.44	15.75
Lizard06Caudovirales_VIRSorter_scaffold_80604-gene_2|3672|5267|1595|+|gi_169257206_ref_YP_001700584.1_|118|1.	bfr:BF1729	531	1	531	14	544	0	89.831	**K01186**	Metabolism	Lipid metabolism	NEU1	sialidase-1 [EC:3.2.1.18]	ko00511,ko00600,ko04142	Other glycan degradation|Sphingolipid metabolism|Lysosome	9.83	512.85	2.42	33.14	5.68	38.05
Lizard06Caudovirales_VIRSorter_scaffold_80604-gene_5|8118|10193|2075|+|-|-|-|-|DUF303|260.3|2.9e-78(+).p1	bfb:VU15_07355	689	1	689	1	689	0	81.277	**K05970**	Not Included in Pathway or Brite	Unclassified: metabolism	SIAE	sialate O-acetylesterase [EC:3.1.1.53]	—	—	17.79	198.19	1.10	8.38	5.14	15.76
Lizard06Caudovirales_VIRSorter_scaffold_80604-gene_6|10253|12730|2477|+|-|-|-|-|Pfam-B_1496|339.5|9.7e-102(+).p1	bfg:BF638R_1732	806	1	806	15	819	0	81.762	**K01192**	Metabolism	Glycan biosynthesis and metabolism	E3.2.1.25, MANBA, manB	beta-mannosidase [EC:3.2.1.25]	ko00511,ko04142	Other glycan degradation|Lysosome	3.52	140.86	1.74	1.40	4.07	7.97
Lizard06Caudovirales_VIRSorter_scaffold_16643-gene_1|1|539|538|-|-|-|-|-|UxaC|243.5|6.9e-73(-).p1	ash:AL1_02740	178	1	178	2	179	2.14E-108	82.022	**K01812**	Metabolism	Carbohydrate metabolism	uxaC	glucuronate isomerase [EC:5.3.1.12]	ko00040	Pentose and glucuronate interconversions	0.00	0.00	0.00	0.00	0.00	19187.51
Lizard06Caudovirales_VIRSorter_scaffold_16643-gene_6|5068|6312|1244|-|-|-|-|-|OEP|187.2|4.3e-56(-).p1	osp:Odosp_1039	405	5	409	14	418	0	82.222	**K12340**	Environmental Information Processing	Membrane transport	tolC	outer membrane protein	ko01501,ko01503,ko02020,ko03070,ko04626,ko05133	beta-Lactam resistance|Cationic antimicrobial peptide (CAMP) resistance|Two-component system|Bacterial secretion system|Plant-pathogen interaction|Pertussis	0.00	0.00	0.00	0.00	0.00	33.39
Lizard06Caudovirales_VIRSorter_scaffold_16643-gene_8|9449|10462|1013|-|-|-|-|-|HlyD_2|95.9|5e-28(-).p2	osp:Odosp_1041	337	1	336	15	351	0	83.68	**K18302**	Brite Hierarchies	Protein families: signaling and cellular processes	mexJ	membrane fusion protein, multidrug efflux system	—	—	0.00	0.00	0.00	1.14	0.00	6.14
Lizard06Caudovirales_VIRSorter_scaffold_16643-gene_9|10713|11489|776|-|Phage_cluster_8293|139.3|2.2e-	osp:Odosp_1043	256	2	257	3	258	1.95E-168	87.109	**K02342**	Genetic Information Processing	Replication and repair	dnaQ	DNA polymerase III subunit epsilon [EC:2.7.7.7]	ko03030,ko03430,ko03440	DNA replication|Mismatch repair|Homologous recombination	0.00	0.00	0.00	0.00	0.00	1.01
Lizard06Caudovirales_VIRSorter_scaffold_16643-gene_10|11543|12511|968|-|Phage_cluster_14685|169.2|2.5e-	osp:Odosp_1044	311	1	311	54	364	0	92.283	**K02338**	Genetic Information Processing	Replication and repair	dnaN	DNA polymerase III subunit beta [EC:2.7.7.7]	ko03030,ko03430,ko03440	DNA replication|Mismatch repair|Homologous recombination	0.00	0.00	0.00	0.31	0.00	0.55
Lizard06Caudovirales_VIRSorter_scaffold_102358-gene_2|453|1409|956|+|Phage_cluster_7121|63.2|2.9e-	mbf:BHV42_00545	329	1	314	1	322	1.76E-76	39.818	**K00604**	Metabolism	Metabolism of cofactors and vitamins	MTFMT, fmt	methionyl-tRNA formyltransferase [EC:2.1.2.9]	ko00670,ko00970	One carbon pool by folate|Aminoacyl-tRNA biosynthesis	0.00	0.00	0.00	0.00	0.00	30.41
Lizard06Caudovirales_VIRSorter_scaffold_102358-gene_3|1449|4076|2627|-|-|-|-|-|Peptidase_M16_C|246.0|4e-74(-).p1	mbf:BHV42_03540	871	1	868	1	859	0	51.665	**K07263**	Brite Hierarchies	Protein families: metabolism	pqqL	zinc protease [EC:3.4.24.-]	—	—	0.00	0.00	0.00	0.00	0.00	42.78
Lizard06Caudovirales_VIRSorter_scaffold_102358-gene_4|4229|5806|1577|+|-|-|-|-|Prismane|107.0|1.5e-31(+).p1	mbf:BHV42_01140	529	3	523	7	523	2.22E-76	32.892	**K05601**	Metabolism	Energy metabolism	hcp	hydroxylamine reductase [EC:1.7.99.1]	ko00910	Nitrogen metabolism	0.00	0.00	0.00	0.00	0.00	7.68
Lizard06Caudovirales_VIRSorter_scaffold_102358-gene_5|5817|6470|653|-|-|-|-|-|Lactamase_B|89.1|5.4e-26(-).p1	cbt:CLH_0967	206	7	212	1	198	5.03E-55	48.058	**K01069**	Metabolism	Carbohydrate metabolism	gloB, gloC, HAGH	hydroxyacylglutathione hydrolase [EC:3.1.2.6]	ko00620	Pyruvate metabolism	0.00	0.00	0.00	0.00	0.00	0.68
Lizard06Caudovirales_VIRSorter_scaffold_102358-gene_6|6577|8691|2114|+|-|-|-|-|FHIPEP|834.8|8.8e-252(+).p1	sri:SELR_05570	692	11	699	21	695	0	51.301	**K02400**	Cellular Processes	Cell motility	flhA	flagellar biosynthesis protein FlhA	ko02040	Flagellar assembly	0.00	0.00	0.00	0.00	0.00	13.09
Lizard06Caudovirales_VIRSorter_scaffold_102358-gene_9|9622|11214|1592|+|Phage_cluster_2466|83.7|1.6e-	bex:A11Q_399	497	55	527	166	642	1.48E-92	36.217	**K02482**	Brite Hierarchies	Protein families: metabolism	K02482	two-component system, NtrC family, sensor kinase [EC:2.7.13.3]	—	—	0.00	0.00	0.00	0.00	0.00	5.57
Lizard06Caudovirales_VIRSorter_scaffold_15856-gene_6|3002|3379|377|+|Phage_cluster_8512|98.0|9.2e-29|2|-|-|-(+).p1	cls:CXIVA_24570	106	16	119	18	123	2.78E-18	38.679	**K03469**	Genetic Information Processing	Replication and repair	rnhA, RNASEH1	ribonuclease HI [EC:3.1.26.4]	ko03030	DNA replication	0.00	0.00	0.00	0.00	0.00	3.44
Lizard06Caudovirales_VIRSorter_scaffold_43673-gene_2|2906|5689|2783|-|Phage_cluster_2718|616.7|8.5e-186|1|-|-|-(-).p1	bpsl:WS57_27665	142	108	243	216	357	2.67E-10	33.803	**K02314**	Genetic Information Processing	Replication and repair	dnaB	replicative DNA helicase [EC:3.6.4.12]	ko03030,ko04112	DNA replication|Cell cycle - Caulobacter	0.00	0.00	0.00	0.00	7.18	25.56

Query ORF ID	Subject gene ID	Alignment length	Query start	Query end	Subject start	Subject end	e-value	Identity(%)	KO	Category	Subcategory	Gene name	Description	Pathway	Pathway definition	Abundance (fpkm)					
																(1) PE-NMC	(2) PE-NQ1	(3) PE-NQ2	(4) PT-SZZ	(5) PT-ZB	(6) PT-RT
Lizard06Caudovirales_VIRSorter_scaffold_86473-gene_1|1|600|599|+|-|-|-|FliP|259.7|3e-78(+).p1	dmi:Desmer_3905	197	1	197	57	253	2.07E-85	59.391	**K02419**	Cellular Processes	Cell motility	fliP	flagellar biosynthetic protein FliP	ko02040	Flagellar assembly	0.00	0.00	0.00	0.00	0.00	9347.76
Lizard06Caudovirales_VIRSorter_scaffold_86473-gene_3|998|1693|695|+|-|-|-|-|Bac_export_1|152.8|1.8e-45(+).p1	dbr:Deba_2317	216	1	216	28	237	3.77E-37	36.111	**K02421**	Cellular Processes	Cell motility	fliR	flagellar biosynthetic protein FliR	ko02040	Flagellar assembly	0.00	0.00	0.00	0.00	20.90	20.21
Lizard06Caudovirales_VIRSorter_scaffold_86473-gene_4|1703|2818|1115|+|-|-|-|-|Bac_export_2|328.0|1.1e-98(+).p1	bmur:ABE28_008040	354	1	354	11	358	2.47E-95	44.35	**K02401**	Cellular Processes	Cell motility	flhB	flagellar biosynthetic protein FlhB	ko02040	Flagellar assembly	0.00	0.00	0.00	0.00	0.00	9.24
Lizard06Caudovirales_VIRSorter_scaffold_86473-gene_5|2896|5361|2465|+|-|-|-|-|B3_4|196.5|4.4e-59(+).p1	mbf:BHV42_04425	824	1	821	1	821	0	62.743	**K01890**	Genetic Information Processing	Translation	FARSB, pheT	phenylalanyl-tRNA synthetase beta chain [EC:6.1.1.20]	ko00970	Aminoacyl-tRNA biosynthesis	0.00	0.00	0.00	0.00	0.00	20.56
Lizard06Caudovirales_VIRSorter_scaffold_86473-gene_8|7537|8301|764|+|-|-|-|-|TatD_DNase|276.1|4.4e-83(+).p1	mbf:BHV42_01995	253	2	254	1	251	7.92E-99	56.522	**K03424**	Not Included in Pathway or Brite	Unclassified: metabolism	tatD	TatD DNase family protein [EC:3.1.21.-]	—	—	0.00	0.00	0.00	0.00	0.00	3.20
Lizard06Caudovirales_VIRSorter_scaffold_86473-gene_9|8431|11256|2825|-|-|-|-|-|tRNA-synt_1|666.8|4e-201(-).p1	mbf:BHV42_04405	935	7	937	10	940	0	69.733	**K01870**	Genetic Information Processing	Translation	IARS, ileS	isoleucyl-tRNA synthetase [EC:6.1.1.5]	ko00970	Aminoacyl-tRNA biosynthesis	0.00	0.00	0.00	0.00	0.00	11.85
Lizard06Caudovirales_VIRSorter_scaffold_39861-gene_2|439|3117|2678|-	mbf:BHV42_07155	891	1	891	2	892	0	86.981	**K01006**	Metabolism	Carbohydrate metabolism	ppdK	pyruvate, orthophosphate dikinase [EC:2.7.9.1]	ko00620,ko00710,ko00720	Pyruvate metabolism|Carbon fixation in photosynthetic organisms|Carbon fixation pathways in prokaryotes	0.00	13.51	70.98	48.92	166.26	430.35
Lizard06Caudovirales_VIRSorter_scaffold_39861-gene_7|8883|9842|959|-|Phage_cluster_405|123.5|1.7e-	mbf:BHV42_05470	312	3	314	5	316	1.20E-146	64.423	**K02031**	Cellular Processes	Cellular community - prokaryotes	ddpD	peptide/nickel transport system ATP-binding protein	ko02024	Quorum sensing	0.00	0.00	0.00	0.00	0.00	2.94
Lizard06Caudovirales_VIRSorter_scaffold_39861-gene_8|9842|10264|422|-|-|-|-|-|DAP_epimerase|106.6|1.4e-31(-).p1	mbf:BHV42_06255	129	5	133	150	276	2.27E-45	59.69	**K01778**	Metabolism	Amino acid metabolism	dapF	diaminopimelate epimerase [EC:5.1.1.7]	ko00300	Lysine biosynthesis	0.00	0.00	0.00	0.00	0.00	1.05
Lizard06Caudovirales_VIRSorter_scaffold_39861-gene_9|10388|10696|308|-|-|-|-|-|DAP_epimerase|75.8|4.9e-22(-).p2	mbf:BHV42_06255	100	3	101	4	103	1.52E-37	57	**K01778**	Metabolism	Amino acid metabolism	dapF	diaminopimelate epimerase [EC:5.1.1.7]	ko00300	Lysine biosynthesis	0.00	0.00	0.00	0.00	0.00	0.19
Lizard06Caudovirales_VIRSorter_scaffold_30160-gene_1|1|1143|1142|-|-|-|-|-|ABC_membrane|105.0|8.9e-31(-).p1	osp:Odosp_1931	261	1	261	9	269	2.11E-132	73.946	**K06147**	Brite Hierarchies	Protein families: signaling and cellular processes	ABCB-BAC	ATP-binding cassette, subfamily B, bacterial	—	—	0.00	1155124.81	0.00	286369.87	20853.75	11807.70
Lizard06Caudovirales_VIRSorter_scaffold_30160-gene_3|1798|3177|1379|+|-|-|-|-|Pyr_redox_2|122.5|4e-36(+).p1	cbut:ATN24_11785	458	1	458	1	458	4.62E-166	49.563	**K21739**	Not Included in Pathway or Brite	Unclassified: metabolism	rclA	probable pyridine nucleotide-disulfide oxidoreductase	—	—	0.00	445.34	4.95	127.42	57.99	56.92
Lizard06Caudovirales_VIRSorter_scaffold_30160-gene_4|3438|4883|1445|+|Phage_cluster_499|204.9|2.6e-	parc:CI960_07180	485	1	480	1	485	0	71.34	**K00033**	Metabolism	Carbohydrate metabolism	PGD, gnd, gntZ	6-phosphogluconate dehydrogenase [EC:1.1.1.44 1.1.1.343]	ko00030,ko00480	Pentose phosphate pathway|Glutathione metabolism	0.00	223.85	0.00	55.18	21.23	89.01
Lizard06Caudovirales_VIRSorter_scaffold_30160-gene_5|4918|6411|1493|+|Phage_cluster_2040|341.3|1.5e-	parc:CI960_07185	487	1	487	8	484	0	67.556	**K00036**	Metabolism	Carbohydrate metabolism	G6PD, zwf	glucose-6-phosphate 1-dehydrogenase [EC:1.1.1.49 1.1.1.363]	ko00030,ko00480,ko05230	Pentose phosphate pathway|Glutathione metabolism|Central carbon metabolism in cancer	0.00	184.83	0.00	19.65	21.20	168.66
Lizard06Caudovirales_VIRSorter_scaffold_30160-gene_6|7122|7823|701|+|-|-|-|-|Glucosamine_iso|161.4|4.2e-48(+).p1	buy:D8S85_06800	231	1	231	1	228	2.17E-93	56.277	**K01057**	Metabolism	Carbohydrate metabolism	PGLS, pgl, devB	6-phosphogluconolactonase [EC:3.1.1.31]	ko00030	Pentose phosphate pathway	0.00	19.78	0.00	5.03	0.00	12.09
Lizard06Caudovirales_VIRSorter_scaffold_30160-gene_7|7883|9553|1670|-|-|-|-|-|Alk_phosphatase|202.3|2.1e-60(-).p1	pdi:BDI_0912	550	13	555	18	566	0	71.636	**K01077**	Metabolism	Metabolism of cofactors and vitamins	E3.1.3.1, phoA, phoB	alkaline phosphatase [EC:3.1.3.1]	ko00730,ko00790,ko02020	Thiamine metabolism|Folate biosynthesis|Two-component system	0.00	46.27	0.00	14.08	0.00	15.48
Lizard06Caudovirales_VIRSorter_scaffold_12570-gene_8|8554|10032|1478|-|Phage_cluster_126|395.9|3.7e-	ova:OBV_01690	464	30	491	21	465	0	56.034	**K06909**	Not Included in Pathway or Brite	Unclassified: genetic information processing	xtmB	phage terminase large subunit	—	—	0.00	0.00	0.00	0.00	0.00	17.77
Lizard06Caudovirales_VIRSorter_scaffold_83807-gene_4|3417|5000|1583|+|-|-|-|-|Bac_Ubq_Cox|567.9|2e-171(+).p1	parc:CI960_19455	517	1	517	1	517	0	85.493	**K00425**	Metabolism	Energy metabolism	cydA	cytochrome bd ubiquinol oxidase subunit I [EC:7.1.1.7]	ko00190,ko02020	Oxidative phosphorylation|Two-component system	21.13	28.21	0.00	33.52	0.00	43.34
Lizard06Caudovirales_VIRSorter_scaffold_83807-gene_5|5135|6223|1088|+|-|-|-|-|Cyto_ox_2|222.5|1.5e-66(+).p1	pdi:BDI_2649	357	6	362	28	383	0	85.994	**K00426**	Metabolism	Energy metabolism	cydB	cytochrome bd ubiquinol oxidase subunit II [EC:7.1.1.7]	ko00190,ko02020	Oxidative phosphorylation|Two-component system	42.19	25.70	0.00	8.37	0.00	25.39
Lizard06Caudovirales_VIRSorter_scaffold_83807-gene_6|6731|7543|812|+|-|-|-|-|Lactamase_B_2|90.2|2.3e-26(+).p1	pdi:BDI_2651	270	1	270	35	304	2.48E-170	83.333	**K00784**	Genetic Information Processing	Translation	rnz	ribonuclease Z [EC:3.1.26.11]	ko03013	RNA transport	0.00	19.61	0.00	12.23	0.00	15.57
Lizard06Caudovirales_VIRSorter_scaffold_83807-gene_8|8006|9463|1457|-|-|-|-|-|Toluene_X|216.3|9.2e-65(-).p1	buy:D8S85_21035	497	1	484	2	486	1.70E-148	46.881	**K06076**	Brite Hierarchies	Protein families: signaling and cellular processes	fadL	long-chain fatty acid transport protein	—	—	0.00	0.00	0.00	0.00	0.00	46.09
Lizard06Caudovirales_VIRSorter_scaffold_83807-gene_9|9740|10405|665|+|Phage_cluster_932|198.8|1.2e-	pdi:BDI_3912	217	1	216	1	217	2.76E-145	88.94	**K06920**	Metabolism	Metabolism of cofactors and vitamins	queC	7-cyano-7-deazaguanine synthase [EC:6.3.4.20]	ko00790	Folate biosynthesis	0.00	19.03	0.00	11.03	0.00	12.88
Lizard06Caudovirales_VIRSorter_scaffold_40874-gene_5|2091|2603|512|+|Phage_cluster_78|110.7|1.3e-32|1|-|-|-(+).p1	dfg:B0537_03395	168	3	168	1	139	1.75E-19	34.524	**K01520**	Metabolism	Nucleotide metabolism	dut, DUT	dUTP pyrophosphatase [EC:3.6.1.23]	ko00240,ko00983	Pyrimidine metabolism|Drug metabolism - other enzymes	0.00	0.00	2.13	0.00	3031.82	85.88
Lizard06Caudovirales_VIRSorter_scaffold_36112-gene_1|2|1012|1010|+|Phage_cluster_57|454.3|8.5e-	byl:A4V09_00730	338	3	336	211	548	2.29E-130	56.213	**K06909**	Not Included in Pathway or Brite	Unclassified: genetic information processing	xtmB	phage terminase large subunit	—	—	0.00	0.00	0.00	0.00	0.00	4427.89
Lizard06Caudovirales_VIRSorter_scaffold_36112-gene_12|7962|8660|698|+|Phage_cluster_305|49.9|5.9e-	cpro:CPRO_07490	114	6	119	4	108	4.56E-25	45.614	**K07741**	Not Included in Pathway or Brite	Unclassified: genetic information processing	antB	anti-repressor protein	—	—	0.00	0.56	0.00	0.00	0.00	1.11
Lizard06Caudovirales_VIRSorter_scaffold_31230-gene_2|623|1642|1019|+|-|-|-|-|HlyD_2|100.1|2.5e-29(+).p1	osp:Odosp_2797	338	2	339	25	362	0	73.669	**K21137**	Brite Hierarchies	Protein families: signaling and cellular processes	triB	membrane fusion protein, multidrug efflux system	—	—	0.00	0.00	0.00	0.00	0.00	26.85
Lizard06Caudovirales_VIRSorter_scaffold_31230-gene_6|7767|10298|2531|-|-|-|-|-|Tricorn_C1|97.6|5.5e-29(-).p1	osp:Odosp_2802	842	1	842	234	1072	0	80.048	**K08676**	Brite Hierarchies	Protein families: metabolism	tri	tricorn protease [EC:3.4.21.-]	—	—	0.00	0.00	0.00	0.00	0.00	20.27
Lizard06Caudovirales_VIRSorter_scaffold_54405-gene_9|6094|6711|617|-|Phage_cluster_158|206.1|1.3e-	lgy:T479_19975	186	3	187	7	192	1.32E-72	58.602	**K06904**	Not Included in Pathway or Brite	Poorly characterized	K06904	uncharacterized protein	—	—	0.00	0.00	0.00	0.00	1.71	5.57
Lizard06Caudovirales_VIRSorter_scaffold_22691-gene_4|3189|3884|695|+|Phage_cluster_52|167.2|1.1e-	aeq:AEQU_1181	177	1	176	1	177	9.58E-40	42.373	**K01358**	Cellular Processes	Cell growth and death	clpP, CLPP	ATP-dependent Clp protease, protease subunit [EC:3.4.21.92]	ko04112,ko04212	Cell cycle - Caulobacter|Longevity regulating pathway - worm	0.00	0.00	0.00	2.24	3.27	4.17
Lizard06Caudovirales_VIRSorter_scaffold_5236-gene_3|1063|2775|1712|-|Phage_cluster_205|456.7|2.9e-	mej:Q7A_2480	588	6	564	330	897	4.05E-68	31.463	**K02335**	Genetic Information Processing	Replication and repair	polA	DNA polymerase I [EC:2.7.7.7]	ko03030,ko03410,ko03420,ko03440	DNA replication|Base excision repair|Nucleotide excision repair|Homologous recombination	0.00	0.00	0.00	0.00	0.00	31.94
Lizard06Caudovirales_VIRSorter_scaffold_5236-gene_8|3939|5870|1931|-|Phage_cluster_8081|1189.2|0|2|-|-|-(-).p1	cth:Cthe_1734	479	40	511	35	489	5.22E-40	28.601	**K06919**	Not Included in Pathway or Brite	Unclassified: genetic information processing	K06919	putative DNA primase/helicase	—	—	0.00	0.00	0.00	0.00	0.00	11.37
Lizard06Caudovirales_VIRSorter_scaffold_5236-gene_10|6918|7388|470|-|Phage_cluster_11019|214.0|1.6e-	mear:Mpt1_c12350	134	10	141	15	142	1.39E-16	34.328	**K01493**	Metabolism	Nucleotide metabolism	comEB	dCMP deaminase [EC:3.5.4.12]	ko00240	Pyrimidine metabolism	0.00	0.00	0.00	0.00	0.00	1.35
Lizard06Caudovirales_VIRSorter_scaffold_51211-gene_5|2047|2622|575|+|-|-|-|-|-|-|-|-(+).p2	bty:Btoyo_1927	181	9	189	452	626	1.78E-13	28.177	**K03466**	Brite Hierarchies	Protein families: genetic information processing	ftsK, spoIIIE	DNA segregation ATPase FtsK/SpoIIIE, S-DNA-T family	—	—	0.00	0.00	0.00	0.00	0.00	7.45
Lizard06Caudovirales_VIRSorter_scaffold_51211-gene_10|4467|4895|428|+|Phage_cluster_9|164.0|3.6e-	eha:Ethha_2440	149	1	142	1	148	1.94E-46	54.362	**K03111**	Genetic Information Processing	Replication and repair	ssb	single-strand DNA-binding protein	ko03030,ko03430,ko03440	DNA replication|Mismatch repair|Homologous recombination	0.00	0.00	0.00	0.00	0.00	3.52
Lizard06Caudovirales_VIRSorter_scaffold_85115-gene_5|4962|7163|2201|+|-|-|-|-|-|-|-|-(+).p1	cthd:CDO33_15605	85	207	288	10	93	1.74E-07	47.059	**K09700**	Not Included in Pathway or Brite	Poorly characterized	K09700	uncharacterized protein	—	—	0.00	0.00	0.00	0.00	0.00	4.86
Lizard06Caudovirales_VIRSorter_scaffold_28892-gene_1|2|1189|1187|+|-|-|-|-|Peptidase_U32|222.1|8.5e-67(+).p1	parc:CI960_22200	390	1	390	17	406	0	87.436	**K08303**	Human Diseases	Infectious disease: bacterial	K08303	putative protease [EC:3.4.-.-]	ko05120	Epithelial cell signaling in Helicobacter pylori infection	0.00	18535.38	0.00	30426.80	31280.63	42310.92
Lizard06Caudovirales_VIRSorter_scaffold_28892-gene_2|1186|1593|407|+|-|-|-|-|-|-|-|-(+).p1	pdi:BDI_3192	133	1	133	1	133	5.54E-81	87.218	**K07107**	Not Included in Pathway or Brite	Unclassified: metabolism	ybgC	acyl-CoA thioester hydrolase [EC:3.1.2.-]	—	—	0.00	5.00	0.00	6.04	8.79	18.67
Lizard06Caudovirales_VIRSorter_scaffold_28892-gene_3|1590|2699|1109|+|gi_526118339_ref_YP_008240336.1_|103|3.	pdi:BDI_3193	367	1	367	1	367	1.11E-179	66.485	**K04096**	Not Included in Pathway or Brite	Unclassified: genetic information processing	smf	DNA processing protein	—	—	0.00	22.38	0.00	31.52	45.90	57.86
Lizard06Caudovirales_VIRSorter_scaffold_28892-gene_5|4104|5030|926|-|-|-|-|-|DUF1730|66.0|3.4e-19(-).p1	parc:CI960_21585	306	1	305	2	303	1.02E-154	68.954	**K18979**	Brite Hierarchies	Protein families: genetic information processing	queG	epoxyqueuosine reductase [EC:1.17.99.6]	—	—	0.00	11.92	0.00	8.72	58.44	42.92
Lizard06Caudovirales_VIRSorter_scaffold_28892-gene_7|6172|6807|635|-|-|-|-|-|Polysacc_deac_1|85.5|4e-25(-).p1	pdi:BDI_3078	202	1	202	2	203	3.04E-129	84.653	**K22278**	Not Included in Pathway or Brite	Unclassified: metabolism	pdgA	peptidoglycan-N-acetylglucosamine deacetylase [EC:3.5.1.104]	—	—	0.00	7.69	0.72	8.70	6.76	13.63
Lizard06Caudovirales_VIRSorter_scaffold_3326-gene_1|1|1326|1325|+|-|-|-|-|SSF|67.3|1.8e-19(+).p1	bvu:BVU_0580	440	1	438	208	627	6.63E-165	60.227	**K03307**	Not Included in Pathway or Brite	Unclassified: signaling and cellular processes	TC.SSS	solute:Na+ symporter, SSS family	—	—	469381.86	1709703.35	262624.91	737402.41	177256.90	1858728.74
Lizard06Caudovirales_VIRSorter_scaffold_3326-gene_2|1702|2127|425|+|-|-|-|-|DUF377|41.6|1.2e-11(+).p1	pdi:BDI_0634	119	1	119	1	119	8.58E-74	89.076	**K18785**	Not Included in Pathway or Brite	Unclassified: metabolism	mp2	beta-1,4-mannooligosaccharide/beta-1,4-mannosyl-N-acetylglucosamine phosphorylase [EC:2.4.1.319 2.4.1.320]	—	—	42.43	82.77	7.85	37.86	0.00	123.14
Lizard06Caudovirales_VIRSorter_scaffold_3326-gene_3|2108|2500|392|+|-|-|-|-|DUF377|139.1|2.4e-41(+).p1	pdi:BDI_0634	130	1	130	203	332	7.03E-78	84.615	**K18785**	Not Included in Pathway or Brite	Unclassified: metabolism	mp2	beta-1,4-mannooligosaccharide/beta-1,4-mannosyl-N-acetylglucosamine phosphorylase [EC:2.4.1.319 2.4.1.320]	—	—	17.13	102.00	14.78	30.57	9.89	105.49
Lizard06Caudovirales_VIRSorter_scaffold_3326-gene_4|4389|5048|659|-|Phage_cluster_3140|181.4|3.5e-	parc:CI960_09730	209	10	218	11	215	1.28E-121	78.469	**K04564**	Environmental Information Processing	Signal transduction	SOD2	superoxide dismutase, Fe-Mn family [EC:1.15.1.1]	ko04013,ko04068,ko04146,ko04211,ko04212,ko04213,ko05016	MAPK signaling pathway - fly|FoxO signaling pathway|Peroxisome|Longevity regulating pathway|Longevity regulating pathway - worm|Longevity regulating pathway - multiple species|Huntington disease	0.00	12.16	0.00	4.08	2.38	19.95
Lizard06Caudovirales_VIRSorter_scaffold_3326-gene_5|5324|5863|539|+|-|-|-|-|PvlArgDC|83.5|2.3e-24(+).p1	pdi:BDI_1202	179	1	179	1	179	8.71E-129	97.765	**K02626**	Metabolism	Amino acid metabolism	pdaD	arginine decarboxylase [EC:4.1.1.19]	ko00330	Arginine and proline metabolism	0.00	9.19	0.84	3.36	0.00	15.16
Lizard06Caudovirales_VIRSorter_scaffold_3326-gene_6|5885|6667|782|+|-|-|-|-|AP_endonuc_2|129.1|2.6e-38(+).p1	pdi:BDI_1200	255	1	255	8	262	5.35E-145	75.686	**K01151**	Genetic Information Processing	Replication and repair	nfo	deoxyribonuclease IV [EC:3.1.21.2]	ko03410	Base excision repair	0.00	12.85	1.51	7.91	1.77	35.95
Lizard06Caudovirales_VIRSorter_scaffold_3326-gene_9|8101|9140|1039|+|-|-|-|-|Peptidase_C69|269.6|7.8e-81(+).p1	parc:CI960_09705	346	1	346	1	346	0	89.595	**K08659**	Brite Hierarchies	Protein families: metabolism	pepDA, pepDB	dipeptidase [EC:3.4.-.-]	—	—	0.00	16.47	0.00	7.51	0.00	22.90
Lizard06Caudovirales_VIRSorter_scaffold_4923-gene_8|3552|4253|701|+|Phage_cluster_3199|200.2|6.8e-	afd:Alfi_2693	234	1	233	49	280	3.19E-81	48.718	**K00390**	Metabolism	Energy metabolism	cysH	phosphoadenosine phosphosulfate reductase [EC:1.8.4.8 1.8.4.10]	ko00920	Sulfur metabolism	0.00	3.76	0.00	0.00	9464.03	67.32
Lizard06Caudovirales_VIRSorter_scaffold_4923-gene_16|6198|7301|1103|+|-|-|-|-|-|-|-|-(+).p1	net:Neut_0002	377	1	365	1	372	2.88E-11	21.22	**K02338**	Genetic Information Processing	Replication and repair	dnaN	DNA polymerase III subunit beta [EC:2.7.7.7]	ko03030,ko03430,ko03440	DNA replication|Mismatch repair|Homologous recombination	0.00	0.00	0.00	0.00	492.91	13.49
Lizard06Caudovirales_VIRSorter_scaffold_4923-gene_20|8390|9080|690|+|Phage_cluster_396|257.5|2.5e-	bcae:A4V03_05100	243	1	220	1	238	2.45E-78	54.733	**K00558**	Metabolism	Amino acid metabolism	DNMT1, dcm	DNA (cytosine-5)-methyltransferase 1 [EC:2.1.1.37]	ko00270,ko05206	Cysteine and methionine metabolism|MicroRNAs in cancer	0.00	0.00	1.59	0.00	1537.31	99.98
Lizard06Caudovirales_VIRSorter_scaffold_71252-gene_1|232|1788|1556|+|-|-|-|-|Pectinesterase|244.1|2.2e-73(+).p1	bcel:BcellWH2_04045	554	1	517	29	579	0	55.235	**K01051**	Metabolism	Carbohydrate metabolism	E3.1.1.11	pectinesterase [EC:3.1.1.11]	ko00040,ko02020	Pentose and glucuronate interconversions|Two-component system	0.00	0.00	0.00	432.02	449.43	818.57
Lizard06Caudovirales_VIRSorter_scaffold_71252-gene_2|1985|3058|1073|+|-|-|-|-|Glycos_transf_4|107.3|1.3e-31(+).p1	osp:Odosp_0938	357	1	357	1	357	0	79.832	**K02851**	Metabolism	Glycan biosynthesis and metabolism	wecA, tagO, rfe	UDP-GlcNAc:undecaprenyl-phosphate/decaprenyl-phosphate GlcNAc-1-phosphate transferase [EC:2.7.8.33 2.7.8.35]	ko00572	Arabinogalactan biosynthesis - Mycobacterium	0.00	0.00	0.00	18.03	0.00	25.53
Lizard06Caudovirales_VIRSorter_scaffold_71252-gene_3|3082|3954|872|+|Phage_cluster_3510|299.8|2.1e-	osp:Odosp_0937	290	1	290	1	290	0	83.103	**K00973**	Metabolism	Glycan biosynthesis and metabolism	rfbA, rmlA, rffH	glucose-1-phosphate thymidylyltransferase [EC:2.7.7.24]	ko00521,ko00523,ko00525	Streptomycin biosynthesis|Polyketide sugar unit biosynthesis|Acarbose and validamycin biosynthesis	0.00	0.00	0.00	2.32	0.00	13.89
Lizard06Caudovirales_VIRSorter_scaffold_71252-gene_4|3980|4525|545|+|Phage_cluster_4297|218.3|1.1e-	osp:Odosp_0936	181	1	181	1	181	1.45E-100	78.453	**K01790**	Metabolism	Glycan biosynthesis and metabolism	rfbC, rmlC	dTDP-4-dehydrorhamnose 3,5-epimerase [EC:5.1.3.13]	ko00521,ko00523	Streptomycin biosynthesis|Polyketide sugar unit biosynthesis	0.00	0.00	0.00	0.90	0.00	2.35
Lizard06Caudovirales_VIRSorter_scaffold_71252-gene_5|4551|5408|857|+|gi_448260370_ref_YP_007348468.1_|159|8.6	osp:Odosp_0935	284	1	284	1	284	4.54E-168	78.521	**K00067**	Metabolism	Glycan biosynthesis and metabolism	rfbD, rmlD	dTDP-4-dehydrorhamnose reductase [EC:1.1.1.133]	ko00521,ko00523	Streptomycin biosynthesis|Polyketide sugar unit biosynthesis	0.00	0.00	0.00	3.93	0.00	7.78
Lizard06Caudovirales_VIRSorter_scaffold_71252-gene_6|5434|6489|1055|+|Phage_cluster_398|347.4|1e-	osp:Odosp_0934	348	1	348	1	348	0	94.828	**K01710**	Metabolism	Glycan biosynthesis and metabolism	rfbB, rmlB, rffG	dTDP-glucose 4,6-dehydratase [EC:4.2.1.46]	ko00521,ko00523,ko00525,ko01055	Streptomycin biosynthesis|Polyketide sugar unit biosynthesis|Acarbose and validamycin biosynthesis|Biosynthesis of vancomycin group antibiotics	0.00	0.00	0.00	3.29	15.35	5.16
Lizard06Caudovirales_VIRSorter_scaffold_10523-gene_8|7516|8136|620|-|Phage_cluster_164|152.3|3.2e-	cbv:U729_2621	208	1	205	3	208	2.23E-47	43.75	**K07273**	Not Included in Pathway or Brite	Unclassified: signaling and cellular processes	acm	lysozyme	—	—	0.00	0.39	0.00	0.00	0.00	0.72
Lizard06Caudovirales_VIRSorter_scaffold_23972-gene_13|7410|8737|1327|+|Phage_cluster_10704|61.7|9.5e-18|2|-|-|-	sfk:KY5_5747	379	81	438	296	646	2.82E-49	31.926	**K03655**	Genetic Information Processing	Replication and repair	recG	ATP-dependent DNA helicase RecG [EC:3.6.4.12]	ko03440	Homologous recombination	0.00	0.00	18.02	0.00	0.00	7.24

Query ORF ID	Subject gene ID	Alignment length	Query start	Query end	Subject start	Subject end	e-value	Identity(%)	KO	Category	Subcategory	Gene name	Description	Pathway	Pathway definition	Abundance (fpkm)					
																(1) PE-NMC	(2) PE-NQ1	(3) PE-NQ2	(4) PT-SZZ	(5) PT-ZB	(6) PT-RT
Lizard06Caudovirales_VIRSorter_scaffold_10394-gene_2|268|1362|1094|+|-|-|-|-|-|-|-|-(+).p1	pdi:BDI_0624	363	1	363	1	363	0	73.003	**K18240**	Metabolism	Amino acid metabolism	xanB2	chorismate lyase / 3-hydroxybenzoate synthase [EC:4.1.3.40 4.1.3.45]	ko00130,ko00400	Ubiquinone and other terpenoid-quinone biosynthesis|Phenylalanine, tyrosine and tryptophan biosynthesis	1077.80	1787.14	215.92	894.91	311.25	2705.93
Lizard06Caudovirales_VIRSorter_scaffold_10394-gene_3|1605|2174|569|+|-|-|-|-|-|-|-|-(+).p1	pdi:BDI_0625	189	1	189	64	252	3.20E-125	88.889	**K09988**	Metabolism	Carbohydrate metabolism	lyxA	D-lyxose ketol-isomerase [EC:5.3.1.15]	ko00040	Pentose and glucuronate interconversions	0.00	121.95	16.64	49.07	12.99	133.34
Lizard06Caudovirales_VIRSorter_scaffold_44566-gene_6|6211|7194|983|-|-|-|-|-|-|-|-|-(-).p1	mbf:BHV42_06395	288	39	325	1	288	4.49E-88	47.569	**K06881**	Metabolism	Energy metabolism	nrnA	bifunctional oligoribonuclease and PAP phosphatase NrnA [EC:3.1.3.7 3.1.13.3]	ko00920	Sulfur metabolism	0.00	0.00	0.00	0.00	0.00	1.35
Lizard06Caudovirales_VIRSorter_scaffold_44566-gene_7|7225|8184|959|+|-|-|-|-|Na_Ca_ex|61.1|1.6e-17(+).p1	clc:Calla_0057	304	6	307	5	302	1.63E-83	50	**K07301**	Brite Hierarchies	Protein families: signaling and cellular processes	yrbG	cation:H+ antiporter	—	—	0.00	0.00	0.00	0.00	0.00	2.18
Lizard06Caudovirales_VIRSorter_scaffold_25273-gene_2|470|991|521|-|Phage_cluster_3124|61.7|1e-	bhl:Bache_2853	169	1	169	29	197	9.50E-109	88.166	**K00860**	Metabolism	Energy metabolism	cysC	adenylylsulfate kinase [EC:2.7.1.25]	ko00230,ko00920	Purine metabolism|Sulfur metabolism	0.00	224.00	0.00	0.00	0.00	25.12
Lizard06Caudovirales_VIRSorter_scaffold_25273-gene_4|2767|3591|824|-|-|-|-|-|Inositol_P|172.2|2.5e-51(-).p1	parc:CI960_07880	267	1	267	3	269	8.24E-165	77.903	**K01082**	Metabolism	Energy metabolism	cysQ, MET22, BPNT1	3'(2'), 5'-bisphosphate nucleotidase [EC:3.1.3.7]	ko00920	Sulfur metabolism	0.00	122.18	3.22	0.00	7.54	42.85
Lizard06Caudovirales_VIRSorter_scaffold_25273-gene_6|5663|6943|1280|+|Phage_cluster_2703|548.4|1.5e-	pdi:BDI_1271	426	1	426	1	426	0	95.54	**K00600**	Metabolism	Carbohydrate metabolism	glyA, SHMT	glycine hydroxymethyltransferase [EC:2.1.2.1]	ko00260,ko00460,ko00630,ko00670,ko00680,ko01523	Glycine, serine and threonine metabolism|Cyanoamino acid metabolism|Glyoxylate and dicarboxylate metabolism|One carbon pool by	63.76	1110.75	8.65	25.92	29.46	214.15
Lizard06Caudovirales_VIRSorter_scaffold_25273-gene_7|7301|8247|946|+|-|-|-|-|Glyco_hydro_20|244.9|2.5e-73(+).p1	pdi:BDI_1272	313	3	315	30	342	0	78.914	**K12373**	Metabolism	Carbohydrate metabolism	HEXA_B	hexosaminidase [EC:3.2.1.52]	ko00511,ko00513,ko00520,ko00531,ko00603,ko00604,ko04142	Other glycan degradation|Various types of N-glycan biosynthesis|Amino sugar and nucleotide sugar metabolism|Glycosaminoglycan degradation|Glycosphingolipid biosynthesis - globo and isoglobo series|Glycosphingolipid biosynthesis - ganglio series|Lysosome	113.74	928.98	28.65	17.16	42.84	277.97
Lizard06Caudovirales_VIRSorter_scaffold_18949-gene_2|841|1755|914|-|Phage_cluster_3012|136.5|1.4e-	peo:AS203_03465	303	1	302	17	319	1.52E-159	69.967	**K06223**	Genetic Information Processing	Replication and repair	dam	DNA adenine methylase [EC:2.1.1.72]	ko03430	Mismatch repair	0.00	0.00	0.00	8.51	173.57	44.46
Lizard06Caudovirales_VIRSorter_scaffold_31215-gene_3|918|2228|1310|+|-|-|-|-|DAO|51.7|1.2e-14(+).p1	mbf:BHV42_07090	437	1	436	1	437	5.79E-172	55.378	**K00313**	Not Included in Pathway or Brite	Unclassified: metabolism	fixC	electron transfer flavoprotein-quinone oxidoreductase [EC:1.5.5.-]	—	—	0.00	0.00	0.00	0.00	0.00	46.09
Lizard06Caudovirales_VIRSorter_scaffold_31215-gene_5|3000|5399|2399|+|Phage_cluster_3078|318.9|9.4e-	mbf:BHV42_05845	736	20	754	10	739	0	45.788	**K07462**	Genetic Information Processing	Replication and repair	recJ	single-stranded-DNA-specific exonuclease [EC:3.1.-.-]	ko03410,ko03430,ko03440	Base excision repair|Mismatch repair|Homologous recombination	0.00	0.00	0.00	0.00	6.95	29.85
Lizard06Caudovirales_VIRSorter_scaffold_31215-gene_6|5431|7323|1892|-|-|-|-|-|Cation_efflux|166.6|1.2e-49(-).p1	ccz:CCALI_00982	259	357	615	254	510	5.44E-58	40.541	**K03699**	Brite Hierarchies	Protein families: signaling and cellular processes	tlyC	putative hemolysin	—	—	0.00	0.00	0.00	0.00	0.00	12.23
Lizard06Caudovirales_VIRSorter_scaffold_31215-gene_7|7699|8130|431|-|-|-|-|-|DUF296|100.5|8.8e-30(-).p1	twi:Thewi_0281	134	9	142	10	141	1.59E-39	50	**K06934**	Not Included in Pathway or Brite	Poorly characterized	K06934	uncharacterized protein	—	—	0.00	0.00	0.00	0.00	0.00	0.58
Lizard06Caudovirales_VIRSorter_scaffold_4415-gene_1|1|1046|1045|-|Phage_cluster_10704|49.1|6.5e-14|2|-|-|-(-).p1	led:BBK82_21665	301	60	347	267	555	2.61E-36	32.558	**K03655**	Genetic Information Processing	Replication and repair	recG	ATP-dependent DNA helicase RecG [EC:3.6.4.12]	ko03440	Homologous recombination	0.00	0.00	0.00	0.00	41707.51	11315.71
Lizard06Caudovirales_VIRSorter_scaffold_60555-gene_4|4013|6229|2216|+|-|-|-|-|RelA_SpoT|119.4|1.5e-35(+).p1	pdi:BDI_2052	738	1	738	1	737	0	78.049	**K01139**	Metabolism	Nucleotide metabolism	spoT	GTP diphosphokinase / guanosine-3',5'-bis(diphosphate) 3'-diphosphatase [EC:2.7.6.5 3.1.7.2]	ko00230	Purine metabolism	0.00	0.00	0.00	1.78	0.00	17.65
Lizard06Caudovirales_VIRSorter_scaffold_60555-gene_5|6404|6778|374|+|-|-|-|-|-|-|-|-(+).p1	parc:CI960_12760	118	9	124	10	126	5.80E-17	47.458	**K07483**	Not Included in Pathway or Brite	Unclassified: genetic information processing	K07483	transposase	—	—	0.00	24.78	0.00	0.56	0.00	1.61
Lizard06Caudovirales_VIRSorter_scaffold_60555-gene_6|6775|7653|878|+|Phage_cluster_2234|222.3|1.2e-	parc:CI960_12755	261	32	292	2	262	2.24E-107	59.387	**K07497**	Not Included in Pathway or Brite	Unclassified: genetic information processing	K07497	putative transposase	—	—	0.00	125.41	0.00	2.11	15.39	4.21
Lizard06Caudovirales_VIRSorter_scaffold_50099-gene_3|933|1496|563|-|Phage_cluster_5178|52.9|5.6e-	buy:D8S85_03610	176	1	176	2	177	2.09E-92	71.591	**K01737**	Metabolism	Metabolism of cofactors and vitamins	queD, ptpS, PTS	6-pyruvoyltetrahydropterin/6-carboxytetrahydropterin synthase [EC:4.2.3.12 4.1.2.50]	ko00790	Folate biosynthesis	0.00	0.00	0.00	0.00	0.00	7.38
Lizard06Caudovirales_VIRSorter_scaffold_50099-gene_4|1490|2176|686|-|Phage_cluster_932|233.5|3.2e-	osp:Odosp_0744	220	1	220	2	221	3.72E-125	77.727	**K06920**	Metabolism	Metabolism of cofactors and vitamins	queC	7-cyano-7-deazaguanine synthase [EC:6.3.4.20]	ko00790	Folate biosynthesis	0.00	0.00	0.00	4.80	0.00	7.26
Lizard06Caudovirales_VIRSorter_scaffold_50099-gene_7|4515|5474|959|+|-|-|-|-|BATS|76.1|2.7e-22(+).p1	osp:Odosp_0742	315	1	315	1	315	0	78.73	**K01012**	Metabolism	Metabolism of cofactors and vitamins	bioB	biotin synthase [EC:2.8.1.6]	ko00780	Biotin metabolism	0.00	0.00	0.00	0.79	0.00	2.18
Lizard06Caudovirales_VIRSorter_scaffold_50099-gene_8|5476|6780|1304|+|Phage_cluster_15111|58.9|7e-	osp:Odosp_0741	425	5	429	1	425	0	80.706	**K00833**	Metabolism	Metabolism of cofactors and vitamins	bioA	adenosylmethionine---8-amino-7-oxononanoate aminotransferase [EC:2.6.1.62]	ko00780	Biotin metabolism	0.00	0.00	0.00	9.15	7.62	3.50
Lizard06Caudovirales_VIRSorter_scaffold_50099-gene_9|6773|7930|1157|+|-|-|-|-|Aminotran_1_2|192.3|2.2e-57(+).p1	osp:Odosp_0740	384	1	384	1	384	0	85.938	**K00652**	Metabolism	Metabolism of cofactors and vitamins	bioF	8-amino-7-oxononanoate synthase [EC:2.3.1.47]	ko00780	Biotin metabolism	0.00	0.00	0.00	2.64	0.00	2.40
Lizard06Caudovirales_VIRSorter_scaffold_1851-gene_6|2649|3836|1187|-|Phage_cluster_457|491.4|4.2e-	ctx:Clo1313_1979	393	7	394	5	396	2.73E-158	60.305	**K14415**	Brite Hierarchies	Protein families: genetic information processing	RTCB, rtcB	tRNA-splicing ligase RtcB (3'-phosphate/5'-hydroxy nucleic acid ligase) [EC:6.5.1.8]	—	—	0.00	0.00	0.00	0.00	0.00	21.36
Lizard06Caudovirales_VIRSorter_scaffold_1851-gene_14|7483|7785|302|-|Phage_cluster_78|69.1|5.8e-	magq:MGMAQ_3933	98	2	97	63	148	1.52E-16	46.939	**K01520**	Metabolism	Nucleotide metabolism	dut, DUT	dUTP pyrophosphatase [EC:3.6.1.23]	ko00240,ko00983	Pyrimidine metabolism|Drug metabolism - other enzymes	0.00	0.00	0.00	0.00	0.00	1.51
Lizard06Caudovirales_VIRSorter_scaffold_2833-gene_2|666|1361|695|-|Phage_cluster_415|112.0|5.2e-	dai:Desaci_2597	225	1	225	112	334	1.97E-101	64	**K04763**	Brite Hierarchies	Protein families: genetic information processing	xerD	integrase/recombinase XerD	—	—	0.00	0.00	0.00	0.00	0.00	28.81
Lizard06Caudovirales_VIRSorter_scaffold_2833-gene_3|1669|2664|995|-|Phage_cluster_415|92.3|5.3e-	evi:Echvi_4349	318	4	320	6	319	1.50E-97	47.799	**K04763**	Brite Hierarchies	Protein families: genetic information processing	xerD	integrase/recombinase XerD	—	—	0.00	0.00	0.00	0.00	0.00	10.61
Lizard06Caudovirales_VIRSorter_scaffold_2833-gene_4|2657|3916|1259|-|Phage_cluster_415|107.4|1.3e-	dai:Desaci_2595	410	6	414	7	415	1.03E-110	43.659	**K04763**	Brite Hierarchies	Protein families: genetic information processing	xerD	integrase/recombinase XerD	—	—	0.00	0.00	0.00	0.00	3.92	7.96
Lizard06Caudovirales_VIRSorter_scaffold_2833-gene_6|4671|5369|698|-|Phage_cluster_8516|51.3|1.3e-	pmz:HMPREF0659_A5856	225	1	225	2	226	5.86E-108	66.667	**K07485**	Not Included in Pathway or Brite	Unclassified: genetic information processing	K07485	transposase	—	—	0.00	10.48	3.81	3.83	0.00	16.12
Lizard06Caudovirales_VIRSorter_scaffold_35027-gene_1|1|1437|1436|-|gi_509139598_ref_YP_008058489.1_|64.3|3.46e-	bsa:Bacsa_3375	482	1	477	484	956	3.75E-153	50.622	**K07317**	Brite Hierarchies	Protein families: signaling and cellular processes	K07317	adenine-specific DNA-methyltransferase [EC:2.1.1.72]	—	—	0.00	2965.66	0.00	0.00	0.00	72322.16
Lizard06Caudovirales_VIRSorter_scaffold_35027-gene_2|1430|2821|1391|-|-|-|-|-|Pfam-B_1496|46.5|4.5e-13(-).p1	bsa:Bacsa_3375	463	2	461	3	463	0	73.65	**K07317**	Brite Hierarchies	Protein families: signaling and cellular processes	K07317	adenine-specific DNA-methyltransferase [EC:2.1.1.72]	—	—	0.00	5.18	0.00	0.00	0.00	91.17
Lizard06Caudovirales_VIRSorter_scaffold_28162-gene_4|3298|4017|719|+|Phage_cluster_52|234.4|5.3e-	aeq:AEQU_1181	177	1	176	1	177	8.16E-75	63.277	**K01358**	Cellular Processes	Cell growth and death	clpP, CLPP	ATP-dependent Clp protease, protease subunit [EC:3.4.21.92]	ko04112,ko04212	Cell cycle - Caulobacter|Longevity regulating pathway - worm	0.00	0.00	0.00	0.00	0.00	5.67
Lizard06Caudovirales_VIRSorter_scaffold_30650-gene_1|215|1285|1070|+|-|-|-|-|URO-D|86.2|3.7e-25(+).p1	pet:PEIBARAKI_4700	348	4	351	1	348	0	77.874	**K01599**	Metabolism	Metabolism of cofactors and vitamins	hemE, UROD	uroporphyrinogen decarboxylase [EC:4.1.1.37]	ko00860	Porphyrin and chlorophyll metabolism	0.00	75.87	20.70	133.20	0.00	169.34
Lizard06Caudovirales_VIRSorter_scaffold_58023-gene_2|223|858|635|+|Phage_cluster_164|166.2|2e-	cby:CLM_0899	199	1	198	1	197	3.69E-61	49.749	**K07273**	Not Included in Pathway or Brite	Unclassified: signaling and cellular processes	acm	lysozyme	—	—	485.73	33.25	59.88	0.00	0.00	37.51
Lizard06Caudovirales_VIRSorter_scaffold_3584-gene_3|803|1783|980|+|-|-|-|-|Aldose_epim|256.8|4.3e-77(+).p1	bth:BT_0372	326	1	325	39	364	0	75.46	**K01785**	Metabolism	Carbohydrate metabolism	galM, GALM	aldose 1-epimerase [EC:5.1.3.3]	ko00010,ko00052	Glycolysis / Gluconeogenesis|Galactose metabolism	0.00	0.00	0.00	0.00	0.00	18.99
Lizard06Caudovirales_VIRSorter_scaffold_3584-gene_4|1831|3072|1241|+|-|-|-|-|MFS_1|92.1|5.2e-27(+).p1	ppn:Palpr_0176	429	1	413	1	429	0	65.268	**K02429**	Brite Hierarchies	Protein families: signaling and cellular processes	fucP	MFS transporter, FHS family, L-fucose permease	—	—	0.00	0.00	0.00	0.00	11.39	17.73
Lizard06Caudovirales_VIRSorter_scaffold_3584-gene_5|3173|4327|1154|+|-|-|-|-|GalKase_gal_bdg|69.3|2.2e-20(+).p1	bdo:EL88_04680	383	1	383	1	383	0	93.211	**K00849**	Metabolism	Carbohydrate metabolism	galK	galactokinase [EC:2.7.1.6]	ko00052,ko00520	Galactose metabolism|Amino sugar and nucleotide sugar metabolism	0.00	0.00	0.00	0.00	0.00	7.44
Lizard06Caudovirales_VIRSorter_scaffold_3584-gene_6|4531|5682|1151|+|-|-|-|-|Aldose_epim|294.1|2e-88(+).p1	bcel:BcellWH2_00928	382	4	383	1	379	0	79.058	**K01785**	Metabolism	Carbohydrate metabolism	galM, GALM	aldose 1-epimerase [EC:5.1.3.3]	ko00010,ko00052	Glycolysis / Gluconeogenesis|Galactose metabolism	0.00	0.00	0.00	0.00	0.00	6.19
Lizard06Caudovirales_VIRSorter_scaffold_3584-gene_7|5707|7401|1694|+|gi_66392035_ref_YP_238960.1_|190|2.20e-	bth:BT_0355	564	1	564	1	564	0	92.376	**K03307**	Not Included in Pathway or Brite	Unclassified: signaling and cellular processes	TC.SSS	solute:Na+ symporter, SSS family	—	—	0.00	0.00	0.00	0.00	0.00	17.76
Lizard06Caudovirales_VIRSorter_scaffold_922-gene_2|661|2424|1763|-|Phage_cluster_2466|162.3|2.2e-	osp:Odosp_2022	586	1	586	2	586	0	76.28	**K07636**	Environmental Information Processing	Signal transduction	phoR	two-component system, OmpR family, phosphate regulon sensor histidine kinase PhoR [EC:2.7.13.3]	ko02020	Two-component system	0.00	0.00	0.00	0.00	94.65	2556.70
Lizard06Caudovirales_VIRSorter_scaffold_922-gene_3|2448|3158|710|-|-|-|-|-|PhoU|97.6|8e-29(-).p1	osp:Odosp_2020	234	2	235	3	236	4.88E-125	76.068	**K02039**	Not Included in Pathway or Brite	Unclassified: signaling and cellular processes	phoU	phosphate transport system protein	—	—	0.00	1.21	0.00	1.46	12.78	167.01
Lizard06Caudovirales_VIRSorter_scaffold_922-gene_4|3189|3959|770|-|Phage_cluster_405|164.9|5.1e-	osp:Odosp_2019	255	1	255	1	255	4.46E-161	90.588	**K02036**	Environmental Information Processing	Membrane transport	pstB	phosphate transport system ATP-binding protein [EC:7.3.2.1]	ko02010	ABC transporters	0.00	0.00	0.00	0.00	0.00	85.62
Lizard06Caudovirales_VIRSorter_scaffold_922-gene_5|3982|4866|884|-|-|-|-|-|BPD_transp_1|79.6|3.9e-23(-).p1	osp:Odosp_2018	291	2	292	3	293	7.01E-169	82.474	**K02038**	Environmental Information Processing	Membrane transport	pstA	phosphate transport system permease protein	ko02010	ABC transporters	0.00	0.00	0.00	0.00	0.00	120.71
Lizard06Caudovirales_VIRSorter_scaffold_922-gene_6|4869|5651|782|-|-|-|-|-|BPD_transp_1|73.9|2.2e-21(-).p1	osp:Odosp_2017	259	1	259	25	283	5.32E-163	87.645	**K02037**	Environmental Information Processing	Membrane transport	pstC	phosphate transport system permease protein	ko02010	ABC transporters	0.00	0.00	0.00	0.00	2.14	77.40
Lizard06Caudovirales_VIRSorter_scaffold_922-gene_7|5738|6784|1046|-|Phage_cluster_1648|243.6|4.8e-	osp:Odosp_2016	351	1	347	2	352	0	75.499	**K02040**	Environmental Information Processing	Membrane transport	pstS	phosphate transport system substrate-binding protein	ko02010,ko02020,ko05152	ABC transporters|Two-component system|Tuberculosis	0.00	0.00	0.00	0.00	0.00	68.77
Lizard06Caudovirales_VIRSorter_scaffold_30483-gene_1|136|495|359|+|-|-|-|-|-|-|-|-(+).p1	parc:CI960_16865	118	2	119	469	586	4.87E-56	73.729	**K02343**	Genetic Information Processing	Replication and repair	dnaX	DNA polymerase III subunit gamma/tau [EC:2.7.7.7]	ko03030,ko03430,ko03440	DNA replication|Mismatch repair|Homologous recombination	0.00	10.90	0.00	0.00	0.00	28.94
Lizard06Caudovirales_VIRSorter_scaffold_30483-gene_3|1434|1898|464|-|Phage_cluster_7048|165.8|8.4e-	parc:CI960_02720	153	1	153	2	154	1.38E-101	88.889	**K09457**	Metabolism	Metabolism of cofactors and vitamins	queF	7-cyano-7-deazaguanine reductase [EC:1.7.1.13]	ko00790	Folate biosynthesis	0.00	0.00	0.00	0.00	0.00	3.77
Lizard06Caudovirales_VIRSorter_scaffold_30483-gene_4|1931|2596|665|-|Phage_cluster_932|204.5|2.3e-	pdi:BDI_3912	218	1	217	2	219	1.24E-141	86.239	**K06920**	Metabolism	Metabolism of cofactors and vitamins	queC	7-cyano-7-deazaguanine synthase [EC:6.3.4.20]	ko00790	Folate biosynthesis	0.00	3.07	0.00	0.00	0.00	13.76
Lizard06Caudovirales_VIRSorter_scaffold_30483-gene_5|2849|4270|1421|+|-|-|-|-|Toluene_X|193.2|9.2e-58(+).p1	osp:Odosp_3257	436	1	436	1	387	1.61E-143	47.936	**K06076**	Brite Hierarchies	Protein families: signaling and cellular processes	fadL	long-chain fatty acid transport protein	—	—	12.67	3.12	0.00	0.00	14.64	28.49
Lizard06Caudovirales_VIRSorter_scaffold_30483-gene_6|4324|6738|2414|-|-|-|-|-|Phosphorylase|70.5|1.4e-20(-).p1	parc:CI960_15645	803	1	803	54	856	0	90.909	**K00688**	Metabolism	Carbohydrate metabolism	PYG, glgP	glycogen phosphorylase [EC:2.4.1.1]	ko00500,ko02026,ko04217,ko04910,ko04922,ko04931	Starch and sucrose metabolism|Biofilm formation - Escherichia coli|Necroptosis|Insulin signaling pathway|Glucagon signaling pathway|Insulin resistance	8.35	8.92	2.06	0.00	0.00	43.12
Lizard06Caudovirales_VIRSorter_scaffold_108397-gene_2|674|1711|1037|-|-|-|-|-|GHMP_kinases_C|43.5|5.3e-12(-).p1	fva:FV113G1_09550	345	1	344	2	343	6.83E-124	52.174	**K07031**	Metabolism	Glycan biosynthesis and metabolism	hddA	D-glycero-alpha-D-manno-heptose-7-phosphate kinase [EC:2.7.1.168]	ko00540	Lipopolysaccharide biosynthesis	0.00	30.80	0.00	5.31	92.82	42.34
Lizard06Caudovirales_VIRSorter_scaffold_108397-gene_3|1708|2433|725|-|Phage_cluster_7979|46.7|4.8e-	flu:CHH17_06280	235	1	231	25	259	2.75E-89	52.766	**K00568**	Metabolism	Metabolism of cofactors and vitamins	ubiG	2-polyprenyl-6-hydroxyphenyl methylase / 3-demethylubiquinone-9 3-methyltransferase [EC:2.1.1.222 2.1.1.64]	ko00130	Ubiquinone and other terpenoid-quinone biosynthesis	0.00	3.47	0.00	0.00	12.21	12.67
Lizard06Caudovirales_VIRSorter_scaffold_108397-gene_4|2521|3939|1418|-|-|-|-|-|AdoHcyase|459.6|4.5e-139(-).p1	pdi:BDI_2775	471	1	471	2	472	0	90.446	**K01251**	Metabolism	Amino acid metabolism	E3.3.1.1, ahcY	adenosylhomocysteinase [EC:3.3.1.1]	ko00270	Cysteine and methionine metabolism	0.00	16.47	0.00	2.84	0.00	46.06
Lizard06Caudovirales_VIRSorter_scaffold_108397-gene_5|4450|6108|1658|-|Phage_cluster_10848|127.7|7.1e-38|2|AMP-	parc:CI960_20095	550	2	551	1	549	0	82.909	**K00666**	Brite Hierarchies	Protein families: metabolism	K00666	fatty-acyl-CoA synthase [EC:6.2.1.-]	—	—	0.00	7.00	0.00	3.22	16.40	33.06
Lizard06Caudovirales_VIRSorter_scaffold_56466-gene_1|283|918|635|-|-|-|-|-|TPK_catalytic|80.3|1.6e-23(-).p1	pdi:BDI_3529	201	6	206	12	212	1.99E-101	70.647	**K00949**	Metabolism	Metabolism of cofactors and vitamins	thiN, TPK1, THI80	thiamine pyrophosphokinase [EC:2.7.6.2]	ko00730	Thiamine metabolism	0.00	10.48	0.00	0.00	147.38	59.11
Lizard06Caudovirales_VIRSorter_scaffold_56466-gene_2|902|1534|632|-	parc:CI960_00765	199	4	201	4	192	1.61E-90	68.342	**K03811**	Brite Hierarchies	Protein families: signaling and cellular processes	pnuC	nicotinamide mononucleotide transporter	—	—	0.00	0.00	0.00	0.00	23.12	15.27
Lizard06Caudovirales_VIRSorter_scaffold_56466-gene_3|1570|3858|2288|-|-|-|-|-|Pfam-B_13725|166.4|2.3e-49(-).p1	pdi:BDI_3531	761	1	761	2	732	0	68.857	**K02014**	Brite Hierarchies	Protein families: signaling and cellular processes	TC.FEV.OM	iron complex outermembrane recepter protein	—	—	0.00	13.22	0.00	31.92	53.13	65.81
Lizard06Caudovirales_VIRSorter_scaffold_56466-gene_4|4280|5362|1082|+|-|-|-|-|MS_channel|155.5|2.2e-46(+).p1	bth:BT_4688	354	1	354	1	354	0	71.469	**K16052**	Brite Hierarchies	Protein families: signaling and cellular processes	ynaI, mscMJ	MscS family membrane protein	—	—	0.00	0.35	2.08	5.85	9.74	14.14
Lizard06Caudovirales_VIRSorter_scaffold_22083-gene_2|1824|2276|452|-|Phage_cluster_5294|77.1|2.5e-	pdi:BDI_2482	148	1	148	2	149	5.83E-87	87.162	**K09117**	Not Included in Pathway or Brite	Poorly characterized	K09117	uncharacterized protein	—	—	0.00	3.25	0.00	0.00	0.00	17.26

Query ORF ID	Subject gene ID	Alignment length	Query start	Query end	Subject start	Subject end	e-value	Identity(%)	KO	Category	Subcategory	Gene name	Description	Pathway	Pathway definition	Abundance (fpkm)					
																(1) PE-NMC	(2) PE-NQ1	(3) PE-NQ2	(4) PT-SZZ	(5) PT-ZB	(6) PT-RT
Lizard06Caudovirales_VIRSorter_scaffold_22083-gene_3|2329|3696|1367|-|-|-|-|-|Tubulin|127.8|9.7e-38(-).p1	pdi:BDI_2483	454	1	454	2	454	0	83.26	**K03531**	Cellular Processes	Cell growth and death	ftsZ	cell division protein FtsZ	ko04112	Cell cycle - Caulobacter	0.00	113.97	0.00	0.00	0.00	152.10
Lizard06Caudovirales_VIRSorter_scaffold_22083-gene_4|3725|5065|1340|-|-|-|-|-|FtsA|112.2|2.3e-33(-).p1	parc:CI960_18670	446	1	443	2	446	0	77.354	**K03590**	Cellular Processes	Cell growth and death	ftsA	cell division protein FtsA	ko04112	Cell cycle - Caulobacter	0.00	48.17	0.00	0.00	0.00	94.17
Lizard06Caudovirales_VIRSorter_scaffold_22083-gene_5|5071|5799|728|-|-|-|-|-|Pfam-B_308|162.6|2.4e-48(-).p1	pdi:BDI_2485	241	1	241	2	242	7.62E-140	77.178	**K03589**	Cellular Processes	Cell growth and death	ftsQ	cell division protein FtsQ	ko04112	Cell cycle - Caulobacter	0.00	5.85	0.00	0.00	0.00	20.86
Lizard06Caudovirales_VIRSorter_scaffold_22083-gene_6|5796|6554|758|-|-|-|-|-|Mur_ligase_C|77.3|1.3e-22(-).p1	pdi:BDI_2486	250	1	250	226	458	6.84E-106	70	**K01924**	Metabolism	Metabolism of other amino acids	murC	UDP-N-acetylmuramate--alanine ligase [EC:6.3.2.8]	ko00471,ko00550	D-Glutamine and D-glutamate metabolism|Peptidoglycan biosynthesis	0.00	9.72	0.00	0.00	0.00	23.77
Lizard06Caudovirales_VIRSorter_scaffold_14131-gene_2|707|1351|644|+|-|-|-|-|Pyridox_oxidase|79.5|2.8e-23(+).p1	bcel:BcellWH2_00518	214	1	214	1	214	1.20E-122	76.636	**K00275**	Metabolism	Metabolism of cofactors and vitamins	pdxH, PNPO	pyridoxamine 5'-phosphate oxidase [EC:1.4.3.5]	ko00750	Vitamin B6 metabolism	0.00	0.00	0.00	0.00	29.50	12.53
Lizard06Caudovirales_VIRSorter_scaffold_14131-gene_4|2488|3216|728|-|-|-|-|-|Glucosamine_iso|161.6|3.7e-48(-).p1	bhl:Bache_2718	229	1	229	2	230	6.11E-123	72.052	**K01057**	Metabolism	Carbohydrate metabolism	PGLS, pgl, devB	6-phosphogluconolactonase [EC:3.1.1.31]	ko00030	Pentose phosphate pathway	0.00	54.83	16.10	1.44	0.00	6.13
Lizard06Caudovirales_VIRSorter_scaffold_14131-gene_5|3213|4613|1400|-|Phage_cluster_2040|315.7|8.4e-	bcel:BcellWH2_00672	465	1	465	34	495	0	82.581	**K00036**	Metabolism	Carbohydrate metabolism	G6PD, zwf	glucose-6-phosphate 1-dehydrogenase [EC:1.1.1.49 1.1.1.363]	ko00030,ko00480,ko05230	Pentose phosphate pathway|Glutathione metabolism|Central carbon metabolism in cancer	101.14	99.22	0.00	4.46	12.98	7.66
Lizard06Caudovirales_VIRSorter_scaffold_14131-gene_6|4906|6333|1427|-|Phage_cluster_499|200.4|6.2e-	bcel:BcellWH2_00673	474	1	474	22	495	0	84.388	**K00033**	Metabolism	Carbohydrate metabolism	PGD, gnd, gntZ	6-phosphogluconate dehydrogenase [EC:1.1.1.44 1.1.1.343]	ko00030,ko00480	Pentose phosphate pathway|Glutathione metabolism	14.72	98.23	10.89	5.11	0.00	5.21
Lizard06Caudovirales_VIRSorter_scaffold_100206-gene_1|38|1174|1136|+|Phage_cluster_3149|53.2|4.9e-	mbf:BHV42_01890	379	7	378	2	345	2.07E-102	45.91	**K03686**	Brite Hierarchies	Protein families: genetic information processing	dnaJ	molecular chaperone DnaJ	—	—	0.00	0.00	0.00	0.00	0.00	414.31
Lizard06Caudovirales_VIRSorter_scaffold_100206-gene_2|1167|1850|683|+|Phage_cluster_1764|113.7|1.6e-	mbf:BHV42_01885	226	5	227	4	222	2.29E-66	47.345	**K10026**	Metabolism	Metabolism of cofactors and vitamins	queE	7-carboxy-7-deazaguanine synthase [EC:4.3.99.3]	ko00790	Folate biosynthesis	0.00	0.00	0.00	0.00	0.00	7.17
Lizard06Caudovirales_VIRSorter_scaffold_100206-gene_4|2641|3225|584|-|-|-|-|-|HD|41.6|2.1e-11(-).p1	mbf:BHV42_05095	180	8	185	8	184	1.36E-35	40	**K01524**	Metabolism	Nucleotide metabolism	ppx-gppA	exopolyphosphatase / guanosine-5'-triphosphate,3'-diphosphate pyrophosphatase [EC:3.6.1.11 3.6.1.40]	ko00230	Purine metabolism	0.00	0.00	0.00	0.00	0.00	2.98
Lizard06Caudovirales_VIRSorter_scaffold_100206-gene_5|3218|3691|473|-|Phage_cluster_3079|59.4|5.1e-	prh:LT40_20795	157	1	153	609	761	3.13E-21	33.121	**K13490**	Environmental Information Processing	Signal transduction	wspE	two-component system, chemotaxis family, sensor histidine kinase and response regulator WspE	ko02020,ko02025	Two-component system|Biofilm formation - Pseudomonas aeruginosa	0.00	0.00	0.00	0.00	0.00	3.06
Lizard06Caudovirales_VIRSorter_scaffold_100206-gene_6|3707|6259|2552|-|-|-|-|-|HATPase_c|66.9|2.3e-19(-).p1	oac:Oscil6304_2363	442	416	849	279	706	1.79E-85	34.615	**K03407**	Environmental Information Processing	Signal transduction	cheA	two-component system, chemotaxis family, sensor kinase CheA [EC:2.7.13.3]	ko02020,ko02030	Two-component system|Bacterial chemotaxis	0.00	0.00	0.00	0.00	0.00	13.94
Lizard06Caudovirales_VIRSorter_scaffold_6827-gene_2|818|1780|962|-|Phage_cluster_3510|52.4|7.8e-	pseg:D3H65_10795	196	1	194	3	197	1.99E-54	48.469	**K15669**	Metabolism	Glycan biosynthesis and metabolism	hddC	D-glycero-alpha-D-manno-heptose 1-phosphate guanylyltransferase [EC:2.7.7.71]	ko00540	Lipopolysaccharide biosynthesis	0.00	0.00	0.00	0.00	0.00	8.42
Lizard06Caudovirales_VIRSorter_scaffold_6827-gene_4|2359|2934|575|-|-|-|-|-|-|-|-|-(-).p1	tpi:TREPR_2396	188	1	188	155	341	4.03E-55	50	**K07031**	Metabolism	Glycan biosynthesis and metabolism	hddA	D-glycero-alpha-D-manno-heptose-7-phosphate kinase [EC:2.7.1.168]	ko00540	Lipopolysaccharide biosynthesis	0.00	0.00	0.00	0.00	0.00	3.13
Lizard06Caudovirales_VIRSorter_scaffold_6827-gene_6|3407|4180|773|-|-|-|-|-|Methyltransf_23|70.8|2e-20(-).p2	flu:CHH17_06280	257	2	254	3	259	5.53E-96	52.14	**K00568**	Metabolism	Metabolism of cofactors and vitamins	ubiG	2-polyprenyl-6-hydroxyphenyl methylase / 3-demethylubiquinone-9 3-methyltransferase [EC:2.1.1.222 2.1.1.64]	ko00130	Ubiquinone and other terpenoid-quinone biosynthesis	0.00	0.00	0.00	0.00	0.00	4.62
Lizard06Caudovirales_VIRSorter_scaffold_6827-gene_7|4208|5626|1418|-|-|-|-|-|AdoHcyase|458.4|1e-138(-).p1	pdi:BDI_2775	471	1	471	2	472	0	90.021	**K01251**	Metabolism	Amino acid metabolism	E3.3.1.1, ahcY	adenosylhomocysteinase [EC:3.3.1.1]	ko00270	Cysteine and methionine metabolism	0.00	2.47	0.00	0.85	0.00	13.45
Lizard06Caudovirales_VIRSorter_scaffold_83857-gene_2|211|708|497|-|-|-|-|-|Acetyltransf_3|45.9|1.2e-12(-).p1	epo:Epro_1335	158	5	162	25	181	1.98E-42	48.101	**K22441**	Not Included in Pathway or Brite	Unclassified: metabolism	paiA	diamine N-acetyltransferase [EC:2.3.1.57]	—	—	0.00	0.00	0.00	0.00	0.00	27.98
Lizard06Caudovirales_VIRSorter_scaffold_83857-gene_3|717|1745|1028|-|Phage_cluster_593|416.8|1.2e-	mbf:BHV42_04885	339	1	339	2	340	0	79.646	**K03553**	Genetic Information Processing	Replication and repair	recA	recombination protein RecA	ko03440	Homologous recombination	0.00	0.00	0.00	0.00	0.00	37.74
Lizard06Caudovirales_VIRSorter_scaffold_48089-gene_4|3226|4464|1238|-|-|-|-|-|Exonuc_VII_L|231.5|2.7e-69(-).p1	bvu:BVU_0271	430	5	411	6	434	0	66.744	**K03601**	Genetic Information Processing	Replication and repair	xseA	exodeoxyribonuclease VII large subunit [EC:3.1.11.6]	ko03430	Mismatch repair	0.00	1.84	0.00	0.00	0.00	4.12
Lizard06Caudovirales_VIRSorter_scaffold_50463-gene_9|4412|5722|1310|-|Phage_cluster_296|213.6|6.7e-	shu:SHYC_05295	439	4	424	1	424	7.37E-61	35.535	**K10747**	Genetic Information Processing	Replication and repair	LIG1	DNA ligase 1 [EC:6.5.1.1 6.5.1.6 6.5.1.7]	ko03030,ko03410,ko03420,ko03430	DNA replication|Base excision repair|Nucleotide excision repair|Mismatch repair	0.00	0.00	0.00	0.00	0.00	5.24
Lizard06Caudovirales_VIRSorter_scaffold_27859-gene_2|330|1787|1457|-|Phage_cluster_15279|303.4|4.2e-91|1|GDC-	txy:Thexy_0917	478	2	477	5	477	0	59.833	**K00283**	Metabolism	Carbohydrate metabolism	gcvPB	glycine dehydrogenase subunit 2 [EC:1.4.4.2]	ko00260,ko00630	Glycine, serine and threonine metabolism|Glyoxylate and dicarboxylate metabolism	0.00	0.00	0.00	0.00	0.00	59.63
Lizard06Caudovirales_VIRSorter_scaffold_27859-gene_3|1883|2971|1088|+|-|-|-|-|SBP_bac_8|104.4|1.7e-30(+).p1	mbf:BHV42_04920	360	1	360	50	404	2.58E-131	54.444	**K17244**	Environmental Information Processing	Membrane transport	chiE	putative chitobiose transport system substrate-binding protein	ko02010	ABC transporters	0.00	0.00	0.00	0.00	0.00	3.14
Lizard06Caudovirales_VIRSorter_scaffold_27859-gene_6|5327|5707|380|+|-|-|-|-|Ribosomal_S30AE|86.9|1.7e-25(+).p1	mbf:BHV42_06425	114	1	113	1	113	4.76E-50	69.298	**K05808**	Brite Hierarchies	Protein families: genetic information processing	yhbH	putative sigma-54 modulation protein	—	—	0.00	0.00	0.00	0.00	0.00	0.37
Lizard06Caudovirales_VIRSorter_scaffold_68788-gene_2|1179|1667|488|-|Phage_cluster_415|128.0|7e-	mbf:BHV42_05605	159	1	159	146	304	3.05E-75	67.925	**K04763**	Brite Hierarchies	Protein families: genetic information processing	xerD	integrase/recombinase XerD	—	—	0.00	0.00	0.00	0.00	0.00	7.09
Lizard06Caudovirales_VIRSorter_scaffold_68788-gene_3|1630|1971|341|-|-|-|-|-|Phage_int_SAM_1|60.7|2.2e-17(-).p1	mbf:BHV42_05605	97	1	97	2	98	1.11E-31	53.608	**K04763**	Brite Hierarchies	Protein families: genetic information processing	xerD	integrase/recombinase XerD	—	—	0.00	0.00	0.00	0.00	0.00	0.60
Lizard06Caudovirales_VIRSorter_scaffold_68788-gene_4|2082|2897|815|+|-|-|-|-|DUF1009|212.0|1.2e-63(+).p1	mbf:BHV42_08900	268	4	271	1	268	7.50E-120	63.806	**K09949**	Metabolism	Glycan biosynthesis and metabolism	K09949	uncharacterized protein	—	—	0.00	0.00	0.00	0.00	0.00	9.69
Lizard06Caudovirales_VIRSorter_scaffold_68788-gene_5|2902|4032|1130|+|-|-|-|-|LpxB|262.9|6.6e-79(+).p1	mbf:BHV42_08905	373	3	375	2	372	3.24E-165	63.003	**K00748**	Metabolism	Glycan biosynthesis and metabolism	lpxB	lipid-A-disaccharide synthase [EC:2.4.1.182]	ko00540	Lipopolysaccharide biosynthesis	0.00	0.00	0.00	0.00	0.00	5.09
Lizard06Caudovirales_VIRSorter_scaffold_68788-gene_6|4029|4625|596|+|-|-|-|-|Chromate_transp|127.9|5.4e-38(+).p1	tur:AT726_00810	167	7	172	5	171	2.00E-39	41.916	**K07240**	Brite Hierarchies	Protein families: signaling and cellular processes	chrA	chromate transporter	—	—	0.00	0.00	0.00	0.00	0.00	1.59
Lizard06Caudovirales_VIRSorter_scaffold_68788-gene_7|4618|5136|518|+|-|-|-|-|Chromate_transp|129.3|2e-38(+).p1	tur:AT726_00805	165	6	170	5	169	7.08E-39	44.242	**K07240**	Brite Hierarchies	Protein families: signaling and cellular processes	chrA	chromate transporter	—	—	0.00	0.00	0.00	0.00	0.00	0.32
Lizard06Caudovirales_VIRSorter_scaffold_43393-gene_2|380|1465|1085|-|Phage_cluster_88|44.8|1.5e-	mbf:BHV42_00750	354	1	351	8	340	1.22E-112	50.847	**K11752**	Metabolism	Metabolism of cofactors and vitamins	ribD	diaminohydroxyphosphoribosylaminopyrimidine deaminase / 5-amino-6-(5-phosphoribosylamino)uracil reductase [EC:3.5.4.26	ko00740,ko02024	Riboflavin metabolism|Quorum sensing	0.00	0.00	0.00	0.00	0.00	36.25
Lizard06Caudovirales_VIRSorter_scaffold_43393-gene_4|2106|3401|1295|-|-|-|-|-|Lyase_1|187.8|5.2e-56(-).p1	mbf:BHV42_06610	426	1	426	2	426	0	63.38	**K01756**	Metabolism	Nucleotide metabolism	purB, ADSL	adenylosuccinate lyase [EC:4.3.2.2]	ko00230,ko00250	Purine metabolism|Alanine, aspartate and glutamate metabolism	0.00	0.00	0.00	0.00	0.00	13.32
Lizard06Caudovirales_VIRSorter_scaffold_43393-gene_6|4256|5413|1157|+|-|-|-|-|Ala_racemase_N|198.4|2e-59(+).p1	mbf:BHV42_06540	374	14	385	5	374	5.28E-158	59.626	**K01775**	Metabolism	Metabolism of other amino acids	alr	alanine racemase [EC:5.1.1.1]	ko00473,ko01502	D-Alanine metabolism|Vancomycin resistance	0.00	0.00	0.00	0.00	0.00	7.40
Lizard06Caudovirales_VIRSorter_scaffold_28793-gene_1|783|3665|2882|-|Phage_cluster_15279|966.6|1.6e-291|1|GDC-	osp:Odosp_1928	960	1	959	2	961	0	92.292	**K00281**	Metabolism	Carbohydrate metabolism	GLDC, gcvP	glycine dehydrogenase [EC:1.4.4.2]	ko00260,ko00630	Glycine, serine and threonine metabolism|Glyoxylate and dicarboxylate metabolism	0.00	0.00	0.00	54.86	53.27	186.62
Lizard06Caudovirales_VIRSorter_scaffold_28793-gene_3|4676|5869|1193|-|-|-|-|-|iPGM_N|219.5|6.6e-66(-).p1	osp:Odosp_3352	395	1	395	112	506	0	91.899	**K15633**	Metabolism	Carbohydrate metabolism	gpmI	2,3-bisphosphoglycerate-independent phosphoglycerate mutase [EC:5.4.2.12]	ko00010,ko00260,ko00680	Glycolysis / Gluconeogenesis|Glycine, serine and threonine metabolism|Methane metabolism	0.00	0.32	0.00	1.53	0.00	2.21
Lizard06Caudovirales_VIRSorter_scaffold_61365-gene_2|225|635|410|+|-|-|-|-|PIN_3|75.8|8.2e-22(+).p1	bsa:Bacsa_1737	135	1	135	1	135	7.35E-47	54.074	**K07063**	Not Included in Pathway or Brite	Poorly characterized	K07063	uncharacterized protein	—	—	0.00	13.18	0.00	79.55	0.00	2.19
Lizard06Caudovirales_VIRSorter_scaffold_61365-gene_3|668|1585|917|+|-|-|-|-|HTS|454.6|1.8e-137(+).p1	bhl:Bache_1156	304	1	304	1	304	0	90.789	**K00651**	Metabolism	Energy metabolism	metA	homoserine O-succinyltransferase/O-acetyltransferase [EC:2.3.1.46 2.3.1.31]	ko00270,ko00920	Cysteine and methionine metabolism|Sulfur metabolism	0.00	17.76	19.99	0.00	0.00	22.83
Lizard06Caudovirales_VIRSorter_scaffold_61365-gene_4|1582|3435|1853|+|-|-|-|-|Peptidase_U32|230.3|2.7e-69(+).p1	bhl:Bache_1157	615	5	617	4	615	0	76.911	**K08303**	Human Diseases	Infectious disease: bacterial	K08303	putative protease [EC:3.4.-.-]	ko05120	Epithelial cell signaling in Helicobacter pylori infection	0.00	4.69	0.00	2.26	19.77	24.57
Lizard06Caudovirales_VIRSorter_scaffold_61365-gene_6|4472|5834|1362|+|-|-|-|-|-|-|-|-(+).p1	bhl:Bache_1161	454	1	454	1	446	0	80.176	**K07137**	Not Included in Pathway or Brite	Poorly characterized	K07137	uncharacterized protein	—	—	0.00	1.66	0.00	2.40	16.32	5.17
Lizard06Caudovirales_VIRSorter_scaffold_55491-gene_2|569|1363|794|-|Phage_cluster_38|135.7|2.4e-	bdh:GV66_19085	263	1	263	196	458	1.34E-156	80.989	**K19789**	Brite Hierarchies	Protein families: genetic information processing	radD	DNA repair protein RadD	—	—	253.82	33.88	62.58	0.00	18.32	16.43
Lizard06Caudovirales_VIRSorter_scaffold_95016-gene_2|855|1778|923|+|Phage_cluster_629|188.7|2.2e-	boa:Bovatus_00439	191	114	304	1	191	5.28E-79	61.78	**K01448**	Human Diseases	Drug resistance: antimicrobial	amiABC	N-acetylmuramoyl-L-alanine amidase [EC:3.5.1.28]	ko01503	Cationic antimicrobial peptide (CAMP) resistance	0.00	0.00	0.00	0.00	0.00	24.74
Lizard06Caudovirales_VIRSorter_scaffold_26663-gene_1|1|488|487|-|Phage_cluster_75|159.6|1.7e-47|1|DNA_methylase|205.0|3.1e-61(-).p1	bdh:GV66_10920	161	1	161	2	162	7.10E-100	84.472	**K00558**	Metabolism	Amino acid metabolism	DNMT1, dcm	DNA (cytosine-5)-methyltransferase 1 [EC:2.1.1.37]	ko00270,ko05206	Cysteine and methionine metabolism|MicroRNAs in cancer	0.00	0.00	0.00	0.00	0.00	36899.06
Lizard06Caudovirales_VIRSorter_scaffold_26663-gene_2|485|1501|1016|-|Phage_cluster_75|175.1|3.4e-	bdh:GV66_10915	333	1	333	1	333	0	89.189	**K00558**	Metabolism	Amino acid metabolism	DNMT1, dcm	DNA (cytosine-5)-methyltransferase 1 [EC:2.1.1.37]	ko00270,ko05206	Cysteine and methionine metabolism|MicroRNAs in cancer	0.00	0.00	0.00	0.00	0.00	185.64
Lizard06Caudovirales_VIRSorter_scaffold_26663-gene_4|2629|2934|305|-	boa:Bovatus_02623	97	1	97	73	168	2.48E-38	67.01	**K07458**	Brite Hierarchies	Protein families: genetic information processing	vsr	DNA mismatch endonuclease, patch repair protein [EC:3.1.-.-]	—	—	0.00	0.00	0.00	0.00	0.00	6.18
Lizard06Caudovirales_VIRSorter_scaffold_23425-gene_1|553|2124|1571|-|-|-|-|-|RNase_E_G|317.3|1.4e-95(-).p1	pdi:BDI_1996	521	1	521	2	522	0	93.474	**K08301**	Brite Hierarchies	Protein families: genetic information processing	rng, cafA	ribonuclease G [EC:3.1.26.-]	—	—	0.00	16.09	0.00	12.95	75.42	490.21
Lizard06Caudovirales_VIRSorter_scaffold_23425-gene_3|2814|3899|1085|+|-|-|-|-|HhH-GPD|61.7|1.4e-17(+).p1	pdi:BDI_1994	359	1	359	1	359	0	74.93	**K03575**	Genetic Information Processing	Replication and repair	mutY	A/G-specific adenine glycosylase [EC:3.2.2.31]	ko03410	Base excision repair	0.00	3.16	0.00	0.00	0.00	71.68
Lizard06Caudovirales_VIRSorter_scaffold_23425-gene_4|3945|4451|506|+|Phage_cluster_9|135.2|2.8e-	pdi:BDI_1993	171	1	168	1	169	1.49E-95	80.702	**K03111**	Genetic Information Processing	Replication and repair	ssb	single-strand DNA-binding protein	ko03030,ko03430,ko03440	DNA replication|Mismatch repair|Homologous recombination	0.00	0.00	0.00	0.00	0.00	29.18
Lizard06Caudovirales_VIRSorter_scaffold_23425-gene_5|4531|5522|991|+|-|-|-|-|DUF21|125.2|3.3e-37(+).p1	pdi:BDI_1992	330	1	330	19	348	0	86.667	**K03699**	Brite Hierarchies	Protein families: signaling and cellular processes	tlyC	putative hemolysin	—	—	0.00	2.62	0.98	0.00	18.41	67.76
Lizard06Caudovirales_VIRSorter_scaffold_58737-gene_7|2727|3809|1082|-|Phage_cluster_3763|440.0|1.1e-	kbs:EPA93_42240	346	10	338	46	387	6.77E-87	42.775	**K07496**	Not Included in Pathway or Brite	Unclassified: genetic information processing	K07496	putative transposase	—	—	0.00	1.09	3.26	0.00	0.00	71.26
Lizard06Caudovirales_VIRSorter_scaffold_66312-gene_3|911|2443|1532|+|-|-|-|-|HMGL-like|199.5|1.2e-59(+).p1	fsu:Fisuc_2358	520	2	507	4	516	4.29E-172	49.038	**K01649**	Metabolism	Carbohydrate metabolism	leuA, IMS	2-isopropylmalate synthase [EC:2.3.3.13]	ko00290,ko00620	Valine, leucine and isoleucine biosynthesis|Pyruvate metabolism	0.00	0.00	0.00	0.00	0.00	47.52
Lizard06Caudovirales_VIRSorter_scaffold_66312-gene_4|2483|3451|968|+|-|-|-|-|BPD_transp_1|157.7|4.3e-47(+).p1	mbf:BHV42_08760	319	1	319	1	319	2.43E-179	77.429	**K02033**	Cellular Processes	Cellular community - prokaryotes	ABC.PE.P	peptide/nickel transport system permease protein	ko02024	Quorum sensing	0.00	0.00	0.00	0.00	0.00	8.52
Lizard06Caudovirales_VIRSorter_scaffold_66312-gene_5|3451|4521|1070|+|-|-|-|-|BPD_transp_1|114.9|5.8e-34(+).p1	mbf:BHV42_08765	353	4	356	3	355	0	77.62	**K02034**	Cellular Processes	Cellular community - prokaryotes	ABC.PE.P1	peptide/nickel transport system permease protein	ko02024	Quorum sensing	0.00	0.00	0.00	0.00	0.00	9.41
Lizard06Caudovirales_VIRSorter_scaffold_66312-gene_6|4565|5275|710|+|-|-|-|-|MgtC|136.7|8.2e-41(+).p1	mbf:BHV42_08770	242	5	236	7	242	7.61E-67	50	**K07507**	Brite Hierarchies	Protein families: signaling and cellular processes	mgtC	putative Mg2+ transporter-C (MgtC) family protein	—	—	0.00	0.00	0.00	0.00	0.00	2.05
Lizard06Caudovirales_VIRSorter_scaffold_62201-gene_1|1|2541|2540|+|Phage_cluster_125|651.1|4.8e-	mbf:BHV42_00365	848	1	845	306	1147	0	60.731	**K02337**	Genetic Information Processing	Replication and repair	dnaE	DNA polymerase III subunit alpha [EC:2.7.7.7]	ko03030,ko03430,ko03440	DNA replication|Mismatch repair|Homologous recombination	0.00	0.00	0.00	0.00	0.00	109221.22
Lizard06Caudovirales_VIRSorter_scaffold_62201-gene_2|2700|3953|1253|-|Phage_cluster_3116|41.9|1.4e-	mbf:BHV42_09030	411	5	415	3	386	1.61E-113	46.959	**K01258**	Brite Hierarchies	Protein families: metabolism	pepT	tripeptide aminopeptidase [EC:3.4.11.4]	—	—	0.00	0.00	0.00	0.00	0.00	12.94
Lizard06Caudovirales_VIRSorter_scaffold_62201-gene_4|4284|5417|1133|-|-|-|-|-|EPSP_synthase|265.2|1.4e-79(-).p1	mbf:BHV42_04960	352	1	352	72	423	0	78.693	**K00790**	Metabolism	Carbohydrate metabolism	murA	UDP-N-acetylglucosamine 1-carboxyvinyltransferase [EC:2.5.1.7]	ko00520,ko00550	Amino sugar and nucleotide sugar metabolism|Peptidoglycan biosynthesis	0.00	0.00	0.00	0.00	0.00	10.11
Lizard06Caudovirales_VIRSorter_scaffold_11219-gene_5|3359|5182|1823|-|Phage_cluster_532|688.8|1.2e-	elm:ELI_3221	586	22	586	287	862	2.48E-129	40.273	**K06919**	Not Included in Pathway or Brite	Unclassified: genetic information processing	K06919	putative DNA primase/helicase	—	—	0.00	0.00	0.00	0.00	0.00	9.67
Lizard06Caudovirales_VIRSorter_scaffold_89512-gene_1|3|2120|2117|+|Phage_cluster_334|134.9|4.6e-40|1|-|-|-(+).p1	bprm:CL3_21810	701	1	635	1413	2079	4.41E-90	32.81	**K21449**	Brite Hierarchies	Protein families: signaling and cellular processes	ata, sadA, emaA	trimeric autotransporter adhesin	—	—	0.00	3459.94	0.00	0.00	0.00	10823.72

Query ORF ID	Subject gene ID	Alignment length	Query start	Query end	Subject start	Subject end	e-value	Identity(%)	KO	Category	Subcategory	Gene name	Description	Pathway	Pathway definition	Abundance (fpkm)					
																(1) PE-NMC	(2) PE-NQ1	(3) PE-NQ2	(4) PT-SZZ	(5) PT-ZB	(6) PT-RT
Lizard06Caudovirales_VIRSorter_scaffold_21507-gene_2|1707|2678|971|+|-|-|-|-|Pfam-B_2744|51.3|1.9e-14(+).p1	parc:CI960_08615	323	1	323	35	357	0	85.449	**K06889**	Not Included in Pathway or Brite	Poorly characterized	K06889	uncharacterized protein	—	—	0.00	3.47	0.00	0.00	48.87	19.02
Lizard06Caudovirales_VIRSorter_scaffold_21507-gene_6|4470|5332|862|+|Phage_cluster_3071|121.6|6.5e-	pdi:BDI_2088	262	1	262	132	390	1.64E-155	87.786	**K02343**	Genetic Information Processing	Replication and repair	dnaX	DNA polymerase III subunit gamma/tau [EC:2.7.7.7]	ko03030,ko03430,ko03440	DNA replication|Mismatch repair|Homologous recombination	0.00	1.33	1.00	0.00	2.33	7.70
Lizard06Caudovirales_VIRSorter_scaffold_24121-gene_1|1|561|560|-|Phage_cluster_164|119.3|3e-35|1|Glyco_hydro_25|60.8|2.8e-17(-).p3	bhan:CGC63_11730	195	1	186	3	196	4.87E-50	46.154	**K07273**	Not Included in Pathway or Brite	Unclassified: signaling and cellular processes	acm	lysozyme	—	—	0.00	0.00	0.00	0.00	0.00	8363.79
Lizard06Caudovirales_VIRSorter_scaffold_4117-gene_1|1|691|690|-|Phage_cluster_396|258.9|9.5e-78|1|DNA_methylase|99.0|5.4e-29(-).p1	bcae:A4V03_05100	242	1	219	2	238	9.45E-76	54.132	**K00558**	Metabolism	Amino acid metabolism	DNMT1, dcm	DNA (cytosine-5)-methyltransferase 1 [EC:2.1.1.37]	ko00270,ko05206	Cysteine and methionine metabolism|MicroRNAs in cancer	0.00	0.00	13353.81	0.00	12345421.56	845234.51
Lizard06Caudovirales_VIRSorter_scaffold_4117-gene_10|3721|4830|1109|-|-|-|-|-|-|-|-|-(-).p1	rti:DC20_16515	327	15	338	16	339	1.28E-08	22.018	**K02338**	Genetic Information Processing	Replication and repair	dnaN	DNA polymerase III subunit beta [EC:2.7.7.7]	ko03030,ko03430,ko03440	DNA replication|Mismatch repair|Homologous recombination	19.41	31.08	2.39	104.86	1062.02	1321.40
Lizard06Caudovirales_VIRSorter_scaffold_110485-gene_5|2948|3646|698|+|Phage_cluster_1305|175.2|2.1e-	mol:YLM1_1835	223	4	217	2	217	2.68E-29	35.874	**K03648**	Genetic Information Processing	Replication and repair	UNG, UDG	uracil-DNA glycosylase [EC:3.2.2.27]	ko03410,ko05340	Base excision repair|Primary immunodeficiency	0.00	0.00	0.00	0.00	0.00	1.34
Lizard06Caudovirales_VIRSorter_scaffold_43851-gene_3|1158|1820|662|+|-|-|-|-|HD|40.2|5.8e-11(+).p1	buy:D8S85_06570	220	1	220	1	220	5.78E-125	82.273	**K09163**	Not Included in Pathway or Brite	Poorly characterized	K09163	uncharacterized protein	—	—	0.00	0.00	0.00	0.00	0.00	5.52
Lizard06Caudovirales_VIRSorter_scaffold_43851-gene_6|3330|4229|899|-|Phage_cluster_3607|154.4|6.4e-	osp:Odosp_1770	296	2	297	22	315	1.33E-174	80.068	**K02395**	Cellular Processes	Cell motility	flgJ	flagellar protein FlgJ	—	—	0.00	0.00	0.00	0.00	0.00	3.99
Lizard06Caudovirales_VIRSorter_scaffold_43851-gene_7|4471|4821|350|+|-|-|-|-|dCMP_cyt_deam_1|41.3|1.8e-11(+).p1	buy:D8S85_08510	114	1	114	51	164	1.28E-51	66.667	**K01489**	Metabolism	Nucleotide metabolism	cdd, CDA	cytidine deaminase [EC:3.5.4.5]	ko00240,ko00983	Pyrimidine metabolism|Drug metabolism - other enzymes	0.00	0.00	0.00	0.00	0.00	0.77
Lizard06Caudovirales_VIRSorter_scaffold_28885-gene_4|2025|3185|1160|+|Phage_cluster_289|430.5|1e-	metm:MSMTP_2767	369	1	367	1	363	2.89E-126	50.949	**K07496**	Not Included in Pathway or Brite	Unclassified: genetic information processing	K07496	putative transposase	—	—	0.00	0.00	0.00	0.00	453.12	73.62
Lizard06Caudovirales_VIRSorter_scaffold_41137-gene_4|3689|4288|599|+|-|-|-|-|DUF218|85.6|4.5e-25(+).p1	bcac:CGC64_11830	193	4	196	16	208	6.84E-92	65.803	**K03748**	Not Included in Pathway or Brite	Poorly characterized	sanA	SanA protein	—	—	0.00	2.81	0.00	0.00	0.00	3.73
Lizard06Caudovirales_VIRSorter_scaffold_27590-gene_2|726|2666|1940|-|-|-|-|-|ATP-grasp_5|177.2|5.2e-53(-).p1	pdi:BDI_1804	644	2	645	43	686	0	95.186	**K24012**	Metabolism	Carbohydrate metabolism	acdAB	acetate---CoA ligase (ADP-forming) [EC:6.2.1.13]	ko00010,ko00620,ko00640	Glycolysis / Gluconeogenesis|Pyruvate metabolism|Propanoate metabolism	0.00	102.12	12.26	0.00	0.00	64.38
Lizard06Caudovirales_VIRSorter_scaffold_27590-gene_3|3004|3861|857|-|-|-|-|-|Epimerase|50.7|3e-14(-).p1	bthu:YBT1518_02740	295	2	283	3	297	3.97E-82	44.407	**K07071**	Not Included in Pathway or Brite	Poorly characterized	K07071	uncharacterized protein	—	—	0.00	1.48	0.00	0.00	0.00	4.59
Lizard06Caudovirales_VIRSorter_scaffold_35676-gene_1|191|1792|1601|+|Phage_cluster_2466|126.6|1.5e-	pdi:BDI_0115	575	1	532	3	571	0	61.217	**K11383**	Environmental Information Processing	Signal transduction	kinB	two-component system, NtrC family, sensor histidine kinase KinB [EC:2.7.13.3]	ko02020	Two-component system	0.00	2453.27	0.00	262.38	0.00	1725.82
Lizard06Caudovirales_VIRSorter_scaffold_35676-gene_2|1988|3313|1325|+|-|-|-|-|AA_kinase|141.3|6.7e-42(+).p1	pdi:BDI_3936	437	1	437	1	436	0	68.879	**K00928**	Metabolism	Amino acid metabolism	lysC	aspartate kinase [EC:2.7.2.4]	ko00260,ko00261,ko00270,ko00300	Glycine, serine and threonine metabolism|Monobactam biosynthesis|Cysteine and methionine metabolism|Lysine biosynthesis	0.00	85.03	0.00	19.81	0.00	65.83
Lizard06Caudovirales_VIRSorter_scaffold_35676-gene_3|3717|4939|1222|+|Phage_cluster_3071|265.3|1.3e-	parc:CI960_16865	407	1	407	1	396	0	80.59	**K02343**	Genetic Information Processing	Replication and repair	dnaX	DNA polymerase III subunit gamma/tau [EC:2.7.7.7]	ko03030,ko03430,ko03440	DNA replication|Mismatch repair|Homologous recombination	0.00	25.53	0.00	0.00	0.00	15.09
Lizard06Caudovirales_VIRSorter_scaffold_16309-gene_4|3455|4825|1370|-|-|-|-|-|SusD-like_3|84.6|1.7e-24(-).p1	osp:Odosp_3606	458	1	455	24	474	4.01E-125	45.197	**K21572**	Brite Hierarchies	Protein families: signaling and cellular processes	susD	starch-binding outer membrane protein, SusD/RagB family	—	—	0.00	1.29	0.00	0.00	6.04	9.40
Lizard06Caudovirales_VIRSorter_scaffold_68093-gene_2|373|2403|2030|-|Phage_cluster_205|368.4|1.4e-	aah:CF65_00531	659	14	632	259	854	1.07E-67	31.563	**K02335**	Genetic Information Processing	Replication and repair	polA	DNA polymerase I [EC:2.7.7.7]	ko03030,ko03410,ko03420,ko03440	DNA replication|Base excision repair|Nucleotide excision repair|Homologous recombination	0.00	0.00	0.00	0.00	0.00	96.29
Lizard06Caudovirales_VIRSorter_scaffold_68093-gene_3|2464|3870|1406|-|Phage_cluster_57|376.0|4.6e-	ipo:Ilyop_2057	416	19	432	13	403	2.72E-112	43.269	**K06909**	Not Included in Pathway or Brite	Unclassified: genetic information processing	xtmB	phage terminase large subunit	—	—	0.00	0.00	0.00	0.00	0.00	7.99
Lizard06Caudovirales_VIRSorter_scaffold_16333-gene_3|954|1910|956|-|Phage_cluster_54|175.7|1.9e-	mdv:C5Q96_00325	315	1	312	2	270	1.55E-58	36.19	**K00560**	Metabolism	Nucleotide metabolism	thyA, TYMS	thymidylate synthase [EC:2.1.1.45]	ko00240,ko00670,ko01523	Pyrimidine metabolism|One carbon pool by folate|Antifolate resistance	0.00	0.00	0.00	0.00	295.10	40.74
Lizard06Caudovirales_VIRSorter_scaffold_16333-gene_6|2456|3454|998|-|Phage_cluster_593|325.5|6.6e-	bvs:BARVI_04895	312	17	326	32	340	1.52E-89	46.474	**K03553**	Genetic Information Processing	Replication and repair	recA	recombination protein RecA	ko03440	Homologous recombination	0.00	0.00	0.00	0.00	220.76	23.44
Lizard06Caudovirales_VIRSorter_scaffold_30956-gene_3|1836|2942|1106|-	pdi:BDI_3193	365	2	364	3	367	5.58E-165	64.11	**K04096**	Not Included in Pathway or Brite	Unclassified: genetic information processing	smf	DNA processing protein	—	—	0.00	2.42	0.00	0.00	0.00	54.40
Lizard06Caudovirales_VIRSorter_scaffold_30956-gene_4|2951|3355|404|-|-|-|-|-|-|-|-|-(-).p1	pdi:BDI_3192	131	1	131	2	132	7.45E-75	83.206	**K07107**	Not Included in Pathway or Brite	Unclassified: metabolism	ybgC	acyl-CoA thioester hydrolase [EC:3.1.2.-]	—	—	0.00	0.00	0.00	0.00	0.00	9.00
Lizard06Caudovirales_VIRSorter_scaffold_30956-gene_5|3368|4645|1277|-|-|-|-|-|Peptidase_U32|225.1|1.1e-67(-).p1	pdi:BDI_3191	415	9	423	1	415	0	80.964	**K08303**	Human Diseases	Infectious disease: bacterial	K08303	putative protease [EC:3.4.-.-]	ko05120	Epithelial cell signaling in Helicobacter pylori infection	0.00	1.32	0.00	2.13	0.00	126.94
Lizard06Caudovirales_VIRSorter_scaffold_62577-gene_5|2517|3278|761|-|-|-|-|-|Acyl-ACP_TE|130.2|1.2e-38(-).p1	osp:Odosp_3525	252	2	252	2	252	3.83E-137	76.587	**K01071**	Metabolism	Lipid metabolism	MCH	medium-chain acyl-[acyl-carrier-protein] hydrolase [EC:3.1.2.21]	ko00061	Fatty acid biosynthesis	0.00	0.00	0.00	2.84	16.57	4.69
Lizard06Caudovirales_VIRSorter_scaffold_62577-gene_6|3344|4360|1016|-|-|-|-|-|MurB_C|94.4|5.9e-28(-).p1	osp:Odosp_3524	337	1	337	2	338	1.78E-167	68.249	**K00075**	Metabolism	Carbohydrate metabolism	murB	UDP-N-acetylmuramate dehydrogenase [EC:1.3.1.98]	ko00520,ko00550	Amino sugar and nucleotide sugar metabolism|Peptidoglycan biosynthesis	0.00	0.00	0.00	6.42	0.00	4.71
Lizard06Caudovirales_VIRSorter_scaffold_5189-gene_2|1021|2916|1895|+|Phage_cluster_10908|743.6|3.6e-	cmiu:B1H56_09665	294	300	592	56	346	1.02E-68	38.776	**K17680**	Human Diseases	Neurodegenerative disease	PEO1	twinkle protein [EC:3.6.4.12]	ko05017	Spinocerebellar ataxia	70.73	193.16	17.44	532.91	2747.14	2779.42
Lizard06Caudovirales_VIRSorter_scaffold_5189-gene_6|4132|4709|577|+|Phage_cluster_1089|146.8|1.3e-	bcel:BcellWH2_05539	172	24	189	8	178	3.30E-42	47.093	**K07741**	Not Included in Pathway or Brite	Unclassified: genetic information processing	antB	anti-repressor protein	—	—	0.00	0.00	0.00	0.00	398.70	425.79
Lizard06Caudovirales_VIRSorter_scaffold_38878-gene_1|3|965|962|+|Phage_cluster_12424|92.1|4.5e-	mcn:Mcup_1526	133	17	137	7	139	8.52E-20	42.105	**K01507**	Metabolism	Energy metabolism	ppa	inorganic pyrophosphatase [EC:3.6.1.1]	ko00190	Oxidative phosphorylation	0.00	0.00	0.00	0.00	0.00	2787.93
Lizard06Caudovirales_VIRSorter_scaffold_5108-gene_2|1226|2725|1499|+|-|-|-|-|PGM_PMM_II|98.8|3.7e-29(+).p1	mbf:BHV42_01210	503	8	496	6	494	0	58.052	**K15778**	Metabolism	Carbohydrate metabolism	pmm-pgm	phosphomannomutase / phosphoglucomutase [EC:5.4.2.8 5.4.2.2]	ko00010,ko00030,ko00051,ko00052,ko00230,ko00500,ko00520,ko00521	Glycolysis / Gluconeogenesis|Pentose phosphate pathway|Fructose and mannose metabolism|Galactose metabolism|Purine metabolism|Starch and sucrose metabolism|Amino sugar and nucleotide sugar metabolism|Streptomycin biosynthesis	0.00	0.00	0.00	0.00	0.00	44.95
Lizard06Caudovirales_VIRSorter_scaffold_10886-gene_5|3458|4102|644|+|Phage_cluster_1193|84.1|1.6e-	bdh:GV66_10915	216	1	214	116	331	6.03E-98	62.963	**K00558**	Metabolism	Amino acid metabolism	DNMT1, dcm	DNA (cytosine-5)-methyltransferase 1 [EC:2.1.1.37]	ko00270,ko05206	Cysteine and methionine metabolism|MicroRNAs in cancer	0.00	0.00	0.00	0.00	0.00	49.08
Lizard06Caudovirales_VIRSorter_scaffold_8602-gene_1|1|1071|1070|+|-|-|-|-|Pfam-B_5933|80.4|9e-24(+).p1	bfs:BF9343_2852	356	1	356	192	547	0	95.787	**K00895**	Metabolism	Carbohydrate metabolism	pfp, PFP	diphosphate-dependent phosphofructokinase [EC:2.7.1.90]	ko00010,ko00030,ko00051	Glycolysis / Gluconeogenesis|Pentose phosphate pathway|Fructose and mannose metabolism	72212.59	1229266.33	40061.43	71592.47	31280.63	144644.32
Lizard06Caudovirales_VIRSorter_scaffold_8602-gene_2|1372|2100|728|+|Phage_cluster_898|104.9|8.9e-	bfr:BF3098	242	1	242	44	285	7.58E-157	84.298	**K07273**	Not Included in Pathway or Brite	Unclassified: signaling and cellular processes	acm	lysozyme	—	—	0.00	172.92	0.00	10.44	0.00	27.61
Lizard06Caudovirales_VIRSorter_scaffold_8602-gene_3|2177|3523|1346|+|-|-|-|-|Pyr_redox_2|129.9|2.2e-38(+).p1	bfs:BF9343_1551	449	1	448	1	449	0	82.628	**K00382**	Metabolism	Carbohydrate metabolism	DLD, lpd, pdhD	dihydrolipoamide dehydrogenase [EC:1.8.1.4]	ko00010,ko00020,ko00260,ko00280,ko00310,ko00380,ko00620,ko00630,ko00640	Glycolysis / Gluconeogenesis|Citrate cycle (TCA cycle)|Glycine, serine and threonine metabolism|Valine, leucine and isoleucine degradation|Lysine degradation|Tryptophan metabolism|Pyruvate metabolism|Glyoxylate and dicarboxylate metabolism|Propanoate metabolism	33.17	479.52	18.40	18.09	0.00	53.79
Lizard06Caudovirales_VIRSorter_scaffold_8602-gene_4|3566|4066|500|+|-|-|-|-|-|-|-|-(+).p1	boa:Bovatus_00155	169	3	166	73	240	2.38E-58	54.438	**K03800**	Metabolism	Metabolism of cofactors and vitamins	lplA, lplJ, lipL1	lipoate---protein ligase [EC:6.3.1.20]	ko00785	Lipoic acid metabolism	20.25	43.25	0.00	0.00	0.00	8.42
Lizard06Caudovirales_VIRSorter_scaffold_42143-gene_2|1096|1548|452|-|-|-|-|-|PEP-utilizers_C|155.9|2e-46(-).p1	mbf:BHV42_07155	148	2	149	746	892	1.59E-82	87.838	**K01006**	Metabolism	Carbohydrate metabolism	ppdK	pyruvate, orthophosphate dikinase [EC:2.7.9.1]	ko00620,ko00710,ko00720	Pyruvate metabolism|Carbon fixation in photosynthetic organisms|Carbon fixation pathways in prokaryotes	0.00	0.00	0.00	13.06	9.51	4.49
Lizard06Caudovirales_VIRSorter_scaffold_42143-gene_3|1674|3776|2102|-	mbf:BHV42_07155	699	1	699	2	700	0	87.124	**K01006**	Metabolism	Carbohydrate metabolism	ppdK	pyruvate, orthophosphate dikinase [EC:2.7.9.1]	ko00620,ko00710,ko00720	Pyruvate metabolism|Carbon fixation in photosynthetic organisms|Carbon fixation pathways in prokaryotes	0.00	0.89	2.66	44.91	62.29	44.38
Lizard06Caudovirales_VIRSorter_scaffold_78798-gene_2|1551|2903|1352|-|-|-|-|-|Na_H_antiport_1|396.8|1.6e-119(-).p1	pdi:BDI_0928	445	1	445	2	446	0	86.517	**K03313**	Brite Hierarchies	Protein families: signaling and cellular processes	nhaA	Na+:H+ antiporter, NhaA family	—	—	0.00	13.38	0.00	0.00	0.00	27.28
Lizard06Caudovirales_VIRSorter_scaffold_59558-gene_3|1277|2209|932|+|-|-|-|-|Pfam-B_2560|116.6|1.4e-34(+).p1	mbf:BHV42_07835	301	8	308	5	304	5.31E-139	63.455	**K07139**	Not Included in Pathway or Brite	Poorly characterized	K07139	uncharacterized protein	—	—	0.00	0.00	0.00	0.00	0.00	13.87
Lizard06Caudovirales_VIRSorter_scaffold_16612-gene_3|386|1294|908|-|Phage_cluster_653|280.2|1.4e-	pdt:Prede_1189	305	1	299	2	300	1.34E-72	45.246	**K03465**	Metabolism	Nucleotide metabolism	thyX, thy1	thymidylate synthase (FAD) [EC:2.1.1.148]	ko00240,ko00670	Pyrimidine metabolism|One carbon pool by folate	0.00	0.00	0.00	0.00	0.00	183.54
Lizard06Caudovirales_VIRSorter_scaffold_840-gene_5|1993|4322|2329|+|Phage_cluster_2718|512.2|3.4e-154|1|-|-|-	kphy:AOZ06_12055	122	118	229	235	356	5.46E-16	37.705	**K06217**	Not Included in Pathway or Brite	Unclassified: signaling and cellular processes	phoH, phoL	phosphate starvation-inducible protein PhoH and related proteins	—	—	0.00	0.00	0.00	0.00	0.00	527.54
Lizard06Caudovirales_VIRSorter_scaffold_28653-gene_1|723|1187|464|+|Phage_cluster_7048|161.1|2.3e-	parc:CI960_02720	153	1	153	1	153	1.70E-105	92.157	**K09457**	Metabolism	Metabolism of cofactors and vitamins	queF	7-cyano-7-deazaguanine reductase [EC:1.7.1.13]	ko00790	Folate biosynthesis	0.00	276.88	6.16	59.41	0.00	67.37
Lizard06Caudovirales_VIRSorter_scaffold_28653-gene_4|2659|4041|1382|+|-|-|-|-|OEP|205.7|9.2e-62(+).p1	bcel:BcellWH2_00662	434	26	459	42	475	0	76.498	**K12340**	Environmental Information Processing	Membrane transport	tolC	outer membrane protein	ko01501,ko01503,ko02020,ko03070,ko04626,ko05133	beta-Lactam resistance|Cationic antimicrobial peptide (CAMP) resistance|Two-component system|Bacterial secretion system|Plant-pathogen interaction|Pertussis	488.84	3288.55	343.18	487.33	176.46	765.64
Lizard06Caudovirales_VIRSorter_scaffold_7340-gene_1|36|1358|1322|+|Phage_cluster_3804|115.4|4.8e-	pdi:BDI_1779	440	1	440	1	440	0	82.727	**K03327**	Brite Hierarchies	Protein families: signaling and cellular processes	TC.MATE, SLC47A, norM, mdtK, dinF	multidrug resistance protein, MATE family	—	—	6017.72	7496.53	4451.27	0.00	0.00	929.31
Lizard06Caudovirales_VIRSorter_scaffold_7340-gene_2|1498|2622|1124|-|Phage_cluster_3071|83.2|3.2e-	pdi:BDI_1778	373	1	373	2	374	0	83.914	**K02341**	Genetic Information Processing	Replication and repair	holB	DNA polymerase III subunit delta' [EC:2.7.7.7]	ko03030,ko03430,ko03440	DNA replication|Mismatch repair|Homologous recombination	0.00	3.96	0.00	7.17	0.00	21.02
Lizard06Caudovirales_VIRSorter_scaffold_87230-gene_4|1555|2328|773|+|Phage_cluster_1089|103.4|2.3e-	sns:VC03_02740	227	25	243	10	235	3.24E-48	44.053	**K07741**	Not Included in Pathway or Brite	Unclassified: genetic information processing	antB	anti-repressor protein	—	—	0.00	8.58	0.00	0.00	1039.33	1334.54
Lizard06Caudovirales_VIRSorter_scaffold_77070-gene_3|1342|2052|710|-|Phage_cluster_415|126.3|2.4e-	bfb:VU15_02325	143	93	235	225	367	2.59E-93	94.406	**K04763**	Brite Hierarchies	Protein families: genetic information processing	xerD	integrase/recombinase XerD	—	—	0.00	1.10	0.00	0.00	0.00	23.83
Lizard06Caudovirales_VIRSorter_scaffold_77820-gene_4|1309|2232|923|-|Phage_cluster_396|315.3|6.5e-	bacq:DOE78_18900	308	2	306	6	302	1.62E-105	53.896	**K00558**	Metabolism	Amino acid metabolism	DNMT1, dcm	DNA (cytosine-5)-methyltransferase 1 [EC:2.1.1.37]	ko00270,ko05206	Cysteine and methionine metabolism|MicroRNAs in cancer	0.00	0.00	0.00	0.00	63.72	33.83
Lizard06Caudovirales_VIRSorter_scaffold_17636-gene_2|127|3150|3023|+|Phage_cluster_38|59.8|2.3e-17|1|Pfam-	sri:SELR_pSRC600110	1010	8	1005	5	997	0	60.396	**K01153**	Brite Hierarchies	Protein families: signaling and cellular processes	hsdR	type I restriction enzyme, R subunit [EC:3.1.21.3]	—	—	0.00	0.00	0.00	0.00	0.00	379.64
Lizard06Caudovirales_VIRSorter_scaffold_33694-gene_2|423|926|503|-|Phage_cluster_78|121.9|5e-	cbut:ATN24_04660	166	1	166	1	145	4.15E-26	37.349	**K01520**	Metabolism	Nucleotide metabolism	dut, DUT	dUTP pyrophosphatase [EC:3.6.1.23]	ko00240,ko00983	Pyrimidine metabolism|Drug metabolism - other enzymes	0.00	0.00	0.00	0.00	98.60	26.75
Lizard06Caudovirales_VIRSorter_scaffold_22655-gene_1|1|1104|1103|-|Phage_cluster_3763|549.2|7.6e-	can:Cyan10605_3209	387	1	367	2	373	4.27E-90	45.22	**K07496**	Not Included in Pathway or Brite	Unclassified: genetic information processing	K07496	putative transposase	—	—	0.00	0.00	0.00	0.00	0.00	51166.70
Lizard06Caudovirales_VIRSorter_scaffold_14015-gene_1|206|928|722|+|-|-|-|-|TIM|285.0|6.7e-86(+).p1	pdi:BDI_2695	240	1	240	12	251	3.46E-164	94.583	**K01803**	Metabolism	Carbohydrate metabolism	TPI, tpiA	triosephosphate isomerase (TIM) [EC:5.3.1.1]	ko00010,ko00051,ko00562,ko00710	Glycolysis / Gluconeogenesis|Fructose and mannose metabolism|Inositol phosphate metabolism|Carbon fixation in photosynthetic organisms	0.00	35.99	0.00	295.41	0.00	38.21
Lizard06Caudovirales_VIRSorter_scaffold_14015-gene_3|1609|2202|593|+|Phage_cluster_388|236.9|2.7e-	pdi:BDI_2693	194	4	197	3	196	6.72E-132	90.722	**K01495**	Metabolism	Metabolism of cofactors and vitamins	GCH1, folE	GTP cyclohydrolase IA [EC:3.5.4.16]	ko00790	Folate biosynthesis	0.00	9.22	0.00	13.35	0.00	3.36
Lizard06Caudovirales_VIRSorter_scaffold_92764-gene_2|316|720|404|+|Phage_cluster_9|60.9|2.4e-17|1|SSB|55.0|1.1e-	mbf:BHV42_06245	135	1	134	1	135	1.78E-39	50.37	**K03111**	Genetic Information Processing	Replication and repair	ssb	single-strand DNA-binding protein	ko03030,ko03430,ko03440	DNA replication|Mismatch repair|Homologous recombination	0.00	0.00	0.00	0.00	0.00	10.90
Lizard06Caudovirales_VIRSorter_scaffold_92764-gene_4|1422|3401|1979|+|Phage_cluster_1540|144.2|6.2e-	aba:Acid345_2524	520	42	559	12	489	3.13E-101	37.5	**K01950**	Metabolism	Metabolism of cofactors and vitamins	E6.3.5.1, NADSYN1, QNS1, nadE	NAD+ synthase (glutamine-hydrolysing) [EC:6.3.5.1]	ko00760	Nicotinate and nicotinamide metabolism	0.00	0.00	0.00	0.00	0.00	53.28
Lizard06Caudovirales_VIRSorter_scaffold_65898-gene_1|1|530|529|-|-|-|-|-|-|-|-|-(-).p1	ptc:phytr_8660	171	5	175	46	216	5.63E-59	54.386	**K04069**	Not Included in Pathway or Brite	Unclassified: metabolism	pflA, pflC, pflE	pyruvate formate lyase activating enzyme [EC:1.97.1.4]	—	—	0.00	0.00	0.00	0.00	0.00	7871.80
Lizard06Caudovirales_VIRSorter_scaffold_65898-gene_2|644|2575|1931|-|Phage_cluster_131|301.0|2.6e-	ptc:phytr_8650	619	23	638	80	695	0	73.021	**K21636**	Metabolism	Nucleotide metabolism	nrdD	ribonucleoside-triphosphate reductase (formate) [EC:1.1.98.6]	ko00230,ko00240	Purine metabolism|Pyrimidine metabolism	0.00	0.00	0.00	0.00	0.00	127.58
Lizard06Caudovirales_VIRSorter_scaffold_65898-gene_3|2765|3736|971|+|-|-|-|-|DNA_pol3_delta|93.0|2.8e-27(+).p1	mbf:BHV42_06605	322	2	321	3	316	2.80E-70	41.615	**K02340**	Genetic Information Processing	Replication and repair	holA	DNA polymerase III subunit delta [EC:2.7.7.7]	ko03030,ko03430,ko03440	DNA replication|Mismatch repair|Homologous recombination	0.00	0.00	0.00	0.00	0.00	6.94

<table>
<tr><th rowspan="2">Query ORF ID</th><th rowspan="2">Subject gene ID</th><th rowspan="2">Alignment length</th><th rowspan="2">Query start</th><th rowspan="2">Query end</th><th rowspan="2">Subject start</th><th rowspan="2">Subject end</th><th rowspan="2">e-value</th><th rowspan="2">Identity(%)</th><th rowspan="2">KO</th><th rowspan="2">Category</th><th rowspan="2">Subcategory</th><th rowspan="2">Gene name</th><th rowspan="2">Description</th><th rowspan="2">Pathway</th><th rowspan="2">Pathway definition</th><th colspan="6">Abundance (fpkm)</th></tr>
<tr><th>(1) PE-NMC</th><th>(2) PE-NQ1</th><th>(3) PE-NQ2</th><th>(4) PT-SZZ</th><th>(5) PT-ZB</th><th>(6) PT-RT</th></tr>
<tr><td>Lizard06Caudovirales_VIRSorter_scaffold_110975-gene_1|1|500|499|-|-|-|-|-|RecR|54.4|1.2e-15(-).p1</td><td>osp:Odosp_1715</td><td>163</td><td>3</td><td>165</td><td>2</td><td>164</td><td>7.46E-91</td><td>77.914</td><td>K06187</td><td>Genetic Information Processing</td><td>Replication and repair</td><td>recR</td><td>recombination protein RecR</td><td>ko03440</td><td>Homologous recombination</td><td>0.00</td><td>0.00</td><td>0.00</td><td>0.00</td><td>0.00</td><td>6887.82</td></tr>
<tr><td>Lizard06Caudovirales_VIRSorter_scaffold_110975-gene_2|510|2243|1733|-|Phage_cluster_3078|331.4|1.6e-</td><td>osp:Odosp_1714</td><td>573</td><td>1</td><td>573</td><td>2</td><td>574</td><td>0</td><td>90.576</td><td>K07462</td><td>Genetic Information Processing</td><td>Replication and repair</td><td>recJ</td><td>single-stranded-DNA-specific exonuclease [EC:3.1.-.-]</td><td>ko03410,ko03430,ko03440</td><td>Base excision repair|Mismatch repair|Homologous recombination</td><td>0.00</td><td>0.00</td><td>0.00</td><td>0.00</td><td>0.00</td><td>109.01</td></tr>
<tr><td>Lizard06Caudovirales_VIRSorter_scaffold_31483-gene_2|848|2695|1847|-|Phage_cluster_3071|257.3|3.4e-</td><td>bfr:BF3231</td><td>617</td><td>1</td><td>614</td><td>2</td><td>614</td><td>0</td><td>82.82</td><td>K02343</td><td>Genetic Information Processing</td><td>Replication and repair</td><td>dnaX</td><td>DNA polymerase III subunit gamma/tau [EC:2.7.7.7]</td><td>ko03030,ko03430,ko03440</td><td>DNA replication|Mismatch repair|Homologous recombination</td><td>255.47</td><td>972.23</td><td>73.49</td><td>37.99</td><td>49.18</td><td>158.97</td></tr>
<tr><td>Lizard06Caudovirales_VIRSorter_scaffold_17344-gene_1|1|642|641|-|-|-|-|-|-|-|-(-).p2</td><td>osp:Odosp_2647</td><td>213</td><td>1</td><td>213</td><td>189</td><td>401</td><td>7.96E-109</td><td>73.239</td><td>K03307</td><td>Not Included in Pathway or Brite</td><td>Unclassified: signaling and cellular processes</td><td>TC.SSS</td><td>solute:Na+ symporter, SSS family</td><td>—</td><td>—</td><td>0.00</td><td>0.00</td><td>0.00</td><td>633593.33</td><td>72988.13</td><td>497891.34</td></tr>
<tr><td>Lizard06Caudovirales_VIRSorter_scaffold_17344-gene_2|621|1013|392|-|-|-|-|-|-|-|-(-).p1</td><td>osp:Odosp_2647</td><td>115</td><td>1</td><td>115</td><td>2</td><td>116</td><td>5.30E-61</td><td>84.348</td><td>K03307</td><td>Not Included in Pathway or Brite</td><td>Unclassified: signaling and cellular processes</td><td>TC.SSS</td><td>solute:Na+ symporter, SSS family</td><td>—</td><td>—</td><td>0.00</td><td>0.00</td><td>0.00</td><td>201.75</td><td>33.58</td><td>197.27</td></tr>
<tr><td>Lizard06Caudovirales_VIRSorter_scaffold_17344-gene_3|1181|2314|1133|+|-|-|-|-|Pfam-B_9039|168.9|3.3e-50(+).p1</td><td>ssr:SALIVB_0236</td><td>301</td><td>3</td><td>287</td><td>4</td><td>304</td><td>4.36E-110</td><td>53.156</td><td>K01869</td><td>Genetic Information Processing</td><td>Translation</td><td>LARS, leuS</td><td>leucyl-tRNA synthetase [EC:6.1.1.4]</td><td>ko00970</td><td>Aminoacyl-tRNA biosynthesis</td><td>0.00</td><td>0.00</td><td>0.00</td><td>600.14</td><td>123.60</td><td>945.65</td></tr>
<tr><td>Lizard06Caudovirales_VIRSorter_scaffold_17344-gene_4|2262|3772|1510|+|-|-|-|-|Anticodon_1|51.3|1.9e-14(+).p1</td><td>osp:Odosp_2649</td><td>413</td><td>1</td><td>413</td><td>496</td><td>908</td><td>0</td><td>89.346</td><td>K01869</td><td>Genetic Information Processing</td><td>Translation</td><td>LARS, leuS</td><td>leucyl-tRNA synthetase [EC:6.1.1.4]</td><td>ko00970</td><td>Aminoacyl-tRNA biosynthesis</td><td>0.00</td><td>0.66</td><td>0.00</td><td>701.05</td><td>73.75</td><td>838.68</td></tr>
<tr><td>Lizard06Caudovirales_VIRSorter_scaffold_2323-gene_4|3202|3768|566|+|Phage_cluster_52|207.4|7.6e-</td><td>aeq:AEQU_1181</td><td>176</td><td>1</td><td>175</td><td>1</td><td>176</td><td>5.72E-72</td><td>61.364</td><td>K01358</td><td>Cellular Processes</td><td>Cell growth and death</td><td>clpP, CLPP</td><td>ATP-dependent Clp protease, protease subunit [EC:3.4.21.92]</td><td>ko04112,ko04212</td><td>Cell cycle - Caulobacter|Longevity regulating pathway - worm</td><td>0.00</td><td>0.00</td><td>0.00</td><td>0.00</td><td>0.00</td><td>0.61</td></tr>
<tr><td>Lizard06Caudovirales_VIRSorter_scaffold_95139-gene_2|588|1526|938|-|-|-|-|-|PdxA|259.8|5.6e-78(-).p1</td><td>mbf:BHV42_02865</td><td>332</td><td>3</td><td>308</td><td>3</td><td>327</td><td>2.47E-113</td><td>52.711</td><td>K22024</td><td>Not Included in Pathway or Brite</td><td>Unclassified: metabolism</td><td>pdxA2</td><td>4-phospho-D-threonate 3-dehydrogenase / 4-phospho-D-erythronate 3-dehydrogenase [EC:1.1.1.408 1.1.1.409]</td><td>—</td><td>—</td><td>0.00</td><td>0.00</td><td>0.00</td><td>0.00</td><td>0.00</td><td>11.71</td></tr>
<tr><td>Lizard06Caudovirales_VIRSorter_scaffold_95139-gene_3|1519|2823|1304|-|-|-|-|-|DUF1537|101.9|7.5e-30(-).p1</td><td>mbf:BHV42_02860</td><td>430</td><td>3</td><td>432</td><td>5</td><td>434</td><td>2.98E-176</td><td>56.744</td><td>K22129</td><td>Not Included in Pathway or Brite</td><td>Unclassified: metabolism</td><td>dtnK, denK</td><td>D-threonate/D-erythronate kinase [EC:2.7.1.219 2.7.1.220]</td><td>—</td><td>—</td><td>0.00</td><td>0.00</td><td>0.00</td><td>0.00</td><td>0.00</td><td>16.84</td></tr>
<tr><td>Lizard06Caudovirales_VIRSorter_scaffold_95139-gene_4|2845|3177|332|-|Phage_cluster_9|104.8|6.7e-31|1|SSB|95.3|3.3e-</td><td>mbf:BHV42_02855</td><td>110</td><td>1</td><td>109</td><td>1</td><td>110</td><td>1.98E-42</td><td>60</td><td>K03111</td><td>Genetic Information Processing</td><td>Replication and repair</td><td>ssb</td><td>single-strand DNA-binding protein</td><td>ko03030,ko03430,ko03440</td><td>DNA replication|Mismatch repair|Homologous recombination</td><td>0.00</td><td>0.00</td><td>0.00</td><td>0.00</td><td>0.00</td><td>1.73</td></tr>
<tr><td>Lizard06Caudovirales_VIRSorter_scaffold_11414-gene_5|3331|3731|400|+|-|-|-|-|-|-|-|-(+).p1</td><td>osp:Odosp_2583</td><td>133</td><td>1</td><td>133</td><td>1</td><td>133</td><td>2.03E-86</td><td>95.489</td><td>K01585</td><td>Metabolism</td><td>Amino acid metabolism</td><td>speA</td><td>arginine decarboxylase [EC:4.1.1.19]</td><td>ko00330</td><td>Arginine and proline metabolism</td><td>0.00</td><td>0.00</td><td>0.00</td><td>19.34</td><td>6.26</td><td>29.69</td></tr>
<tr><td>Lizard06Caudovirales_VIRSorter_scaffold_106651-gene_1|1|487|486|-|-|-|-|-|MFS_1|51.6|1.1e-14(-).p1</td><td>mbf:BHV42_01760</td><td>155</td><td>8</td><td>161</td><td>1</td><td>155</td><td>3.11E-44</td><td>50.323</td><td>K08223</td><td>Brite Hierarchies</td><td>Protein families: signaling and cellular processes</td><td>fsr</td><td>MFS transporter, FSR family, fosmidomycin resistance protein</td><td>—</td><td>—</td><td>0.00</td><td>0.00</td><td>0.00</td><td>0.00</td><td>0.00</td><td>6887.82</td></tr>
<tr><td>Lizard06Caudovirales_VIRSorter_scaffold_106651-gene_2|501|2036|1535|-|gi_61806062_ref_YP_214422.1_|256|6.60e-</td><td>len:LEP3755_13220</td><td>514</td><td>6</td><td>510</td><td>3</td><td>511</td><td>2.99E-161</td><td>47.276</td><td>K00602</td><td>Metabolism</td><td>Nucleotide metabolism</td><td>purH</td><td>phosphoribosylaminoimidazolecarboxamide formyltransferase / IMP cyclohydrolase [EC:2.1.2.3 3.5.4.10]</td><td>ko00230,ko00670,ko01523</td><td>Purine metabolism|One carbon pool by folate|Antifolate resistance</td><td>0.00</td><td>0.00</td><td>0.00</td><td>0.00</td><td>0.00</td><td>70.70</td></tr>
<tr><td>Lizard06Caudovirales_VIRSorter_scaffold_83693-gene_3|795|1667|872|-|Phage_cluster_3510|308.5|4.8e-</td><td>mbf:BHV42_06690</td><td>289</td><td>1</td><td>289</td><td>2</td><td>290</td><td>9.17E-169</td><td>78.547</td><td>K00973</td><td>Metabolism</td><td>Glycan biosynthesis and metabolism</td><td>rfbA, rmlA, rffH</td><td>glucose-1-phosphate thymidylyltransferase [EC:2.7.7.24]</td><td>ko00521,ko00523,ko00525</td><td>Streptomycin biosynthesis|Polyketide sugar unit biosynthesis|Acarbose and validamycin biosynthesis</td><td>0.00</td><td>0.00</td><td>0.00</td><td>0.00</td><td>0.00</td><td>21.04</td></tr>
<tr><td>Lizard06Caudovirales_VIRSorter_scaffold_83693-gene_4|1667|2503|836|-</td><td>mbf:BHV42_05410</td><td>274</td><td>4</td><td>277</td><td>1</td><td>272</td><td>1.08E-121</td><td>59.489</td><td>K00067</td><td>Metabolism</td><td>Glycan biosynthesis and metabolism</td><td>rfbD, rmlD</td><td>dTDP-4-dehydrorhamnose reductase [EC:1.1.1.133]</td><td>ko00521,ko00523</td><td>Streptomycin biosynthesis|Polyketide sugar unit biosynthesis</td><td>0.00</td><td>0.00</td><td>0.00</td><td>0.00</td><td>0.00</td><td>10.92</td></tr>
<tr><td>Lizard06Caudovirales_VIRSorter_scaffold_83693-gene_5|2503|3489|986|-|Phage_cluster_398|353.5|1.4e-</td><td>mbf:BHV42_05405</td><td>322</td><td>2</td><td>323</td><td>4</td><td>323</td><td>0</td><td>85.093</td><td>K01710</td><td>Metabolism</td><td>Glycan biosynthesis and metabolism</td><td>rfbB, rmlB, rffG</td><td>dTDP-glucose 4,6-dehydratase [EC:4.2.1.46]</td><td>ko00521,ko00523,ko00525,ko01055</td><td>Streptomycin biosynthesis|Polyketide sugar unit biosynthesis|Acarbose and validamycin biosynthesis|Biosynthesis of vancomycin group antibiotics</td><td>0.00</td><td>0.00</td><td>0.00</td><td>0.00</td><td>0.00</td><td>6.09</td></tr>
<tr><td>Lizard06Caudovirales_VIRSorter_scaffold_26929-gene_1|1|510|509|-|-|-|-|-|MreB_Mbl|219.6|7.6e-66(-).p1</td><td>toh:BCB71_01715</td><td>169</td><td>1</td><td>169</td><td>2</td><td>170</td><td>5.40E-105</td><td>87.574</td><td>K03569</td><td>Brite Hierarchies</td><td>Protein families: genetic information processing</td><td>mreB</td><td>rod shape-determining protein MreB and related proteins</td><td>—</td><td>—</td><td>0.00</td><td>0.00</td><td>0.00</td><td>0.00</td><td>62561.26</td><td>361610.81</td></tr>
<tr><td>Lizard06Caudovirales_VIRSorter_scaffold_26929-gene_2|597|1862|1265|-|gi_61806062_ref_YP_214422.1_|231|1.41e-</td><td>pdi:BDI_1885</td><td>409</td><td>12</td><td>420</td><td>99</td><td>507</td><td>0</td><td>88.264</td><td>K00602</td><td>Metabolism</td><td>Nucleotide metabolism</td><td>purH</td><td>phosphoribosylaminoimidazolecarboxamide formyltransferase / IMP cyclohydrolase [EC:2.1.2.3 3.5.4.10]</td><td>ko00230,ko00670,ko01523</td><td>Purine metabolism|One carbon pool by folate|Antifolate resistance</td><td>0.00</td><td>14.90</td><td>0.00</td><td>0.00</td><td>244.52</td><td>1623.48</td></tr>
<tr><td>Lizard06Caudovirales_VIRSorter_scaffold_74782-gene_2|134|619|485|+|-|-|-|-|-|-|-|-(+).p1</td><td>mbf:BHV42_00075</td><td>148</td><td>1</td><td>145</td><td>1</td><td>145</td><td>5.18E-23</td><td>37.838</td><td>K10947</td><td>Brite Hierarchies</td><td>Protein families: genetic information processing</td><td>padR</td><td>PadR family transcriptional regulator, regulatory protein PadR</td><td>—</td><td>—</td><td>0.00</td><td>0.00</td><td>0.00</td><td>0.00</td><td>0.00</td><td>22.03</td></tr>
<tr><td>Lizard06Caudovirales_VIRSorter_scaffold_74782-gene_4|2836|3651|815|-|Phage_cluster_1661|147.0|8.9e-</td><td>mbf:BHV42_02520</td><td>241</td><td>29</td><td>267</td><td>20</td><td>259</td><td>9.85E-63</td><td>46.473</td><td>K03090</td><td>Brite Hierarchies</td><td>Protein families: genetic information processing</td><td>sigB</td><td>RNA polymerase sigma-B factor</td><td>—</td><td>—</td><td>0.00</td><td>0.00</td><td>0.00</td><td>0.00</td><td>0.00</td><td>4.51</td></tr>
<tr><td>Lizard06Caudovirales_VIRSorter_scaffold_103893-gene_1|103|1314|1211|+|-|-|-|-|Glyco_transf_5|166.8|1.1e-49(+).p1</td><td>mbf:BHV42_00505</td><td>401</td><td>1</td><td>401</td><td>76</td><td>476</td><td>9.48E-165</td><td>58.354</td><td>K00703</td><td>Metabolism</td><td>Carbohydrate metabolism</td><td>glgA</td><td>starch synthase [EC:2.4.1.21]</td><td>ko00500,ko02026</td><td>Starch and sucrose metabolism|Biofilm formation - Escherichia coli</td><td>0.00</td><td>0.00</td><td>0.00</td><td>347.54</td><td>303.70</td><td>114.64</td></tr>
<tr><td>Lizard06Caudovirales_VIRSorter_scaffold_103893-gene_3|2224|3039|815|+|-|-|-|-|-|-|-|-(+).p1</td><td>tta:Theth_1677</td><td>271</td><td>4</td><td>270</td><td>3</td><td>249</td><td>1.34E-37</td><td>34.686</td><td>K02392</td><td>Cellular Processes</td><td>Cell motility</td><td>flgG</td><td>flagellar basal-body rod protein FlgG</td><td>ko02040</td><td>Flagellar assembly</td><td>0.00</td><td>0.00</td><td>0.00</td><td>11.27</td><td>14.07</td><td>4.42</td></tr>
<tr><td>Lizard06Caudovirales_VIRSorter_scaffold_103893-gene_4|3130|3650|520|+|-|-|-|-|Flg_bb_rod|47.8|1.5e-13(+).p1</td><td>tai:Taci_1147</td><td>173</td><td>1</td><td>173</td><td>1</td><td>173</td><td>1.27E-62</td><td>56.069</td><td>K02392</td><td>Cellular Processes</td><td>Cell motility</td><td>flgG</td><td>flagellar basal-body rod protein FlgG</td><td>ko02040</td><td>Flagellar assembly</td><td>0.00</td><td>0.00</td><td>0.00</td><td>11.44</td><td>19.99</td><td>1.26</td></tr>
<tr><td>Lizard06Caudovirales_VIRSorter_scaffold_89795-gene_1|1|317|316|-|-|-|-|-|-|-|-(-).p1</td><td>mbf:BHV42_07055</td><td>75</td><td>31</td><td>104</td><td>28</td><td>102</td><td>3.23E-09</td><td>45.333</td><td>K09807</td><td>Not Included in Pathway or Brite</td><td>Poorly characterized</td><td>K09807</td><td>uncharacterized protein</td><td>—</td><td>—</td><td>0.00</td><td>0.00</td><td>0.00</td><td>0.00</td><td>0.00</td><td>491.99</td></tr>
<tr><td>Lizard06Caudovirales_VIRSorter_scaffold_89795-gene_3|764|2686|1922|-|Phage_cluster_828|579.4|1.5e-</td><td>mbf:BHV42_07065</td><td>641</td><td>1</td><td>637</td><td>24</td><td>657</td><td>0</td><td>56.006</td><td>K01972</td><td>Genetic Information Processing</td><td>Replication and repair</td><td>E6.5.1.2, ligA, ligB</td><td>DNA ligase (NAD+) [EC:6.5.1.2]</td><td>ko03030,ko03410,ko03420,ko03430</td><td>DNA replication|Base excision repair|Nucleotide excision repair|Mismatch repair</td><td>0.00</td><td>0.00</td><td>0.00</td><td>0.00</td><td>0.00</td><td>45.72</td></tr>
<tr><td>Lizard06Caudovirales_VIRSorter_scaffold_89795-gene_4|2821|3649|828|+|-|-|-|-|OKR_DC_1|171.5|4.1e-51(+).p1</td><td>mbf:BHV42_05990</td><td>274</td><td>3</td><td>275</td><td>30</td><td>302</td><td>2.19E-62</td><td>38.321</td><td>K01585</td><td>Metabolism</td><td>Amino acid metabolism</td><td>speA</td><td>arginine decarboxylase [EC:4.1.1.19]</td><td>ko00330</td><td>Arginine and proline metabolism</td><td>0.00</td><td>0.00</td><td>0.00</td><td>0.00</td><td>0.00</td><td>1.92</td></tr>
<tr><td>Lizard06Caudovirales_VIRSorter_scaffold_106486-gene_1|63|815|752|+|-|-|-|-|SpoU_methylase|130.4|8.5e-39(+).p1</td><td>parc:CI960_07490</td><td>247</td><td>1</td><td>247</td><td>1</td><td>247</td><td>1.08E-163</td><td>88.664</td><td>K03218</td><td>Brite Hierarchies</td><td>Protein families: genetic information processing</td><td>rlmB</td><td>23S rRNA (guanosine2251-2'-O)-methyltransferase [EC:2.1.1.185]</td><td>—</td><td>—</td><td>0.00</td><td>23.54</td><td>0.00</td><td>0.00</td><td>0.00</td><td>70.28</td></tr>
<tr><td>Lizard06Caudovirales_VIRSorter_scaffold_106486-gene_2|851|1231|380|+|-|-|-|-|Ribonuc_L-PSP|152.8|6e-46(+).p1</td><td>pdi:BDI_0709</td><td>125</td><td>1</td><td>125</td><td>1</td><td>125</td><td>5.21E-74</td><td>87.2</td><td>K09022</td><td>Not Included in Pathway or Brite</td><td>Unclassified: metabolism</td><td>ridA, tdcF, RIDA</td><td>2-iminobutanoate/2-iminopropanoate deaminase [EC:3.5.99.10]</td><td>—</td><td>—</td><td>0.00</td><td>0.00</td><td>0.00</td><td>0.00</td><td>0.00</td><td>2.89</td></tr>
<tr><td>Lizard06Caudovirales_VIRSorter_scaffold_66761-gene_1|1|462|461|-|-|-|-|-|Peptidase_M50|103.4|1.4e-30(-).p1</td><td>osp:Odosp_1644</td><td>153</td><td>1</td><td>153</td><td>29</td><td>181</td><td>8.36E-101</td><td>93.464</td><td>K11749</td><td>Cellular Processes</td><td>Cell growth and death</td><td>rseP</td><td>regulator of sigma E protease [EC:3.4.24.-]</td><td>ko04112</td><td>Cell cycle - Caulobacter</td><td>0.00</td><td>0.00</td><td>0.00</td><td>0.00</td><td>0.00</td><td>2951.92</td></tr>
<tr><td>Lizard06Caudovirales_VIRSorter_scaffold_66761-gene_2|476|1633|1157|-|-|-|-|-|DXP_reductoisom|155.6|1.8e-46(-).p1</td><td>osp:Odosp_1645</td><td>381</td><td>1</td><td>381</td><td>2</td><td>382</td><td>0</td><td>82.677</td><td>K00099</td><td>Metabolism</td><td>Metabolism of terpenoids and polyketides</td><td>dxr</td><td>1-deoxy-D-xylulose-5-phosphate reductoisomerase [EC:1.1.1.267]</td><td>ko00900</td><td>Terpenoid backbone biosynthesis</td><td>0.00</td><td>0.00</td><td>0.00</td><td>22.56</td><td>0.00</td><td>53.75</td></tr>
<tr><td>Lizard06Caudovirales_VIRSorter_scaffold_66761-gene_3|1698|2558|860|-|Phage_cluster_1627|61.4|7.6e-</td><td>osp:Odosp_1646</td><td>285</td><td>1</td><td>285</td><td>2</td><td>286</td><td>1.88E-168</td><td>81.754</td><td>K22719</td><td>Not Included in Pathway or Brite</td><td>Unclassified: signaling and cellular processes</td><td>envC</td><td>murein hydrolase activator</td><td>—</td><td>—</td><td>0.00</td><td>0.00</td><td>0.00</td><td>8.43</td><td>0.00</td><td>8.69</td></tr>
<tr><td>Lizard06Caudovirales_VIRSorter_scaffold_66761-gene_4|2647|3072|425|-|-|-|-|-|-|-|-(-).p1</td><td>osp:Odosp_1647</td><td>133</td><td>1</td><td>133</td><td>266</td><td>398</td><td>2.06E-78</td><td>83.459</td><td>K16053</td><td>Brite Hierarchies</td><td>Protein families: signaling and cellular processes</td><td>ybdG, mscM</td><td>miniconductance mechanosensitive channel</td><td>—</td><td>—</td><td>0.00</td><td>0.00</td><td>0.00</td><td>0.00</td><td>0.00</td><td>0.56</td></tr>
<tr><td>Lizard06Caudovirales_VIRSorter_scaffold_66761-gene_5|3086|3598|512|-|-|-|-|-|MS_channel|112.1|4.1e-33(-).p1</td><td>osp:Odosp_1647</td><td>169</td><td>1</td><td>169</td><td>107</td><td>275</td><td>1.60E-99</td><td>93.491</td><td>K16053</td><td>Brite Hierarchies</td><td>Protein families: signaling and cellular processes</td><td>ybdG, mscM</td><td>miniconductance mechanosensitive channel</td><td>—</td><td>—</td><td>0.00</td><td>0.00</td><td>0.00</td><td>4.64</td><td>0.00</td><td>0.64</td></tr>
<tr><td>Lizard06Caudovirales_VIRSorter_scaffold_7994-gene_4|1934|3100|1166|+|Phage_cluster_2527|58.7|7.9e-17|1|-|-|-(+).p1</td><td>parc:CI960_16940</td><td>388</td><td>1</td><td>388</td><td>1</td><td>388</td><td>0</td><td>94.072</td><td>K01933</td><td>Metabolism</td><td>Nucleotide metabolism</td><td>purM</td><td>phosphoribosylformylglycinamidine cyclo-ligase [EC:6.3.3.1]</td><td>ko00230</td><td>Purine metabolism</td><td>0.00</td><td>654.78</td><td>9.21</td><td>3.70</td><td>0.00</td><td>94.89</td></tr>
<tr><td>Lizard06Caudovirales_VIRSorter_scaffold_7994-gene_5|3109|3596|487|+|Phage_cluster_5428|112.9|2.3e-</td><td>pdi:BDI_2102</td><td>150</td><td>1</td><td>150</td><td>1</td><td>150</td><td>2.39E-86</td><td>83.333</td><td>K02835</td><td>Brite Hierarchies</td><td>Protein families: genetic information processing</td><td>prfA, MTRF1, MRF1</td><td>peptide chain release factor 1</td><td>—</td><td>—</td><td>11.61</td><td>93.48</td><td>1.43</td><td>0.00</td><td>10.06</td><td>16.46</td></tr>
<tr><td>Lizard06Caudovirales_VIRSorter_scaffold_11199-gene_1|111|464|353|+|Phage_cluster_57|84.5|1.1e-24|0|-|-|-(+).p1</td><td>pdc:CDIF630_03195</td><td>109</td><td>1</td><td>109</td><td>316</td><td>410</td><td>8.46E-35</td><td>58.716</td><td>K06909</td><td>Not Included in Pathway or Brite</td><td>Unclassified: genetic information processing</td><td>xtmB</td><td>phage terminase large subunit</td><td>—</td><td>—</td><td>0.00</td><td>66.79</td><td>0.00</td><td>0.00</td><td>0.00</td><td>22.16</td></tr>
<tr><td>Lizard06Caudovirales_VIRSorter_scaffold_11199-gene_2|534|1898|1364|+|Phage_cluster_767|231.8|2.1e-</td><td>smw:SMWW4_v1c19340</td><td>429</td><td>39</td><td>452</td><td>27</td><td>443</td><td>9.03E-33</td><td>27.972</td><td>K09961</td><td>Not Included in Pathway or Brite</td><td>Poorly characterized</td><td>K09961</td><td>uncharacterized protein</td><td>—</td><td>—</td><td>0.00</td><td>61.09</td><td>8.34</td><td>0.00</td><td>0.00</td><td>48.83</td></tr>
<tr><td>Lizard06Caudovirales_VIRSorter_scaffold_16051-gene_2|294|1664|1370|+|Phage_cluster_863|54.9|8.5e-</td><td>pdi:BDI_1729</td><td>427</td><td>1</td><td>427</td><td>1</td><td>427</td><td>2.21E-178</td><td>64.637</td><td>K03546</td><td>Brite Hierarchies</td><td>Protein families: genetic information processing</td><td>sbcC, rad50</td><td>DNA repair protein SbcC/Rad50</td><td>—</td><td>—</td><td>736.86</td><td>8891.94</td><td>60.56</td><td>24.35</td><td>212.79</td><td>16635.54</td></tr>
<tr><td>Lizard06Caudovirales_VIRSorter_scaffold_16051-gene_4|1985|3055|1070|+|Phage_cluster_863|57.8|1.1e-</td><td>parc:CI960_05655</td><td>356</td><td>1</td><td>356</td><td>638</td><td>993</td><td>2.18E-145</td><td>69.101</td><td>K03546</td><td>Brite Hierarchies</td><td>Protein families: genetic information processing</td><td>sbcC, rad50</td><td>DNA repair protein SbcC/Rad50</td><td>—</td><td>—</td><td>0.00</td><td>825.45</td><td>4.48</td><td>9.02</td><td>57.78</td><td>1945.40</td></tr>
<tr><td>Lizard06Caudovirales_VIRSorter_scaffold_16051-gene_5|3099|3421|322|+|-|-|-|-|-|-|-|-(+).p1</td><td>boa:Bovatus_04724</td><td>107</td><td>1</td><td>107</td><td>1</td><td>103</td><td>5.99E-26</td><td>51.402</td><td>K07076</td><td>Not Included in Pathway or Brite</td><td>Poorly characterized</td><td>K07076</td><td>uncharacterized protein</td><td>—</td><td>—</td><td>0.00</td><td>19.14</td><td>0.00</td><td>1.16</td><td>0.00</td><td>79.85</td></tr>
<tr><td>Lizard06Caudovirales_VIRSorter_scaffold_96353-gene_1|32|793|761|+|-|-|-|-|SIMPL|108.1|1e-31(+).p1</td><td>mbf:BHV42_07055</td><td>255</td><td>1</td><td>253</td><td>1</td><td>245</td><td>7.48E-87</td><td>54.51</td><td>K09807</td><td>Not Included in Pathway or Brite</td><td>Poorly characterized</td><td>K09807</td><td>uncharacterized protein</td><td>—</td><td>—</td><td>0.00</td><td>0.00</td><td>0.00</td><td>0.00</td><td>0.00</td><td>169.12</td></tr>
<tr><td>Lizard06Caudovirales_VIRSorter_scaffold_96353-gene_2|1540|3078|1538|-|-|-|-|-|Prismane|526.6|1e-158(-).p1</td><td>mbf:BHV42_03185</td><td>507</td><td>3</td><td>508</td><td>3</td><td>509</td><td>0</td><td>69.428</td><td>K05601</td><td>Metabolism</td><td>Energy metabolism</td><td>hcp</td><td>hydroxylamine reductase [EC:1.7.99.1]</td><td>ko00910</td><td>Nitrogen metabolism</td><td>0.00</td><td>0.00</td><td>0.00</td><td>0.00</td><td>0.00</td><td>11.18</td></tr>
<tr><td>Lizard06Caudovirales_VIRSorter_scaffold_58545-gene_1|361|1278|917|+|-|-|-|-|HTS|456.3|5.8e-138(+).p1</td><td>bvu:BVU_2042</td><td>305</td><td>1</td><td>305</td><td>1</td><td>305</td><td>0</td><td>94.098</td><td>K00651</td><td>Metabolism</td><td>Energy metabolism</td><td>metA</td><td>homoserine O-succinyltransferase/O-acetyltransferase [EC:2.3.1.46 2.3.1.31]</td><td>ko00270,ko00920</td><td>Cysteine and methionine metabolism|Sulfur metabolism</td><td>900.16</td><td>2279.70</td><td>36.99</td><td>0.00</td><td>0.00</td><td>27.26</td></tr>
<tr><td>Lizard06Caudovirales_VIRSorter_scaffold_58545-gene_2|1275|3101|1826|+|-|-|-|-|Peptidase_U32|238.9|6.4e-72(+).p1</td><td>bdo:EL88_03170</td><td>607</td><td>1</td><td>607</td><td>1</td><td>607</td><td>0</td><td>84.514</td><td>K08303</td><td>Human Diseases</td><td>Infectious disease: bacterial</td><td>K08303</td><td>putative protease [EC:3.4.-.-]</td><td>ko05120</td><td>Epithelial cell signaling in Helicobacter pylori infection</td><td>84.96</td><td>1920.12</td><td>20.95</td><td>16.85</td><td>0.00</td><td>29.71</td></tr>
<tr><td>Lizard06Caudovirales_VIRSorter_scaffold_55954-gene_2|571|1353|782|-|-|-|-|-|Bac_export_1|158.3|4e-47(-).p1</td><td>mbf:BHV42_06945</td><td>258</td><td>1</td><td>258</td><td>1</td><td>258</td><td>7.92E-106</td><td>60.078</td><td>K02421</td><td>Cellular Processes</td><td>Cell motility</td><td>fliR</td><td>flagellar biosynthetic protein FliR</td><td>ko02040</td><td>Flagellar assembly</td><td>0.00</td><td>0.00</td><td>0.00</td><td>0.00</td><td>0.00</td><td>18.09</td></tr>
<tr><td>Lizard06Caudovirales_VIRSorter_scaffold_55954-gene_4|1729|2223|494|-|-|-|-|-|Bac_export_3|73.6|1.5e-21(-).p1</td><td>mbf:BHV42_06940</td><td>164</td><td>1</td><td>163</td><td>2</td><td>162</td><td>1.55E-58</td><td>54.878</td><td>K02420</td><td>Cellular Processes</td><td>Cell motility</td><td>fliQ</td><td>flagellar biosynthetic protein FliQ</td><td>ko02040</td><td>Flagellar assembly</td><td>0.00</td><td>0.00</td><td>0.00</td><td>0.00</td><td>0.00</td><td>1.99</td></tr>
<tr><td>Lizard06Caudovirales_VIRSorter_scaffold_55954-gene_5|2248|2889|641|-|-|-|-|-|FliP|239.8|3.8e-72(-).p1</td><td>mbf:BHV42_06935</td><td>212</td><td>1</td><td>212</td><td>2</td><td>213</td><td>7.17E-117</td><td>75.472</td><td>K02419</td><td>Cellular Processes</td><td>Cell motility</td><td>fliP</td><td>flagellar biosynthetic protein FliP</td><td>ko02040</td><td>Flagellar assembly</td><td>0.00</td><td>0.00</td><td>0.00</td><td>0.00</td><td>0.00</td><td>4.60</td></tr>
<tr><td>Lizard06Caudovirales_VIRSorter_scaffold_93450-gene_3|773|3049|2276|+|Phage_cluster_2193|92.6|3.5e-</td><td>mbf:BHV42_02360</td><td>723</td><td>35</td><td>756</td><td>6</td><td>722</td><td>0</td><td>66.113</td><td>K01139</td><td>Metabolism</td><td>Nucleotide metabolism</td><td>spoT</td><td>GTP diphosphokinase / guanosine-3',5'-bis(diphosphate) 3'-diphosphatase [EC:2.7.6.5 3.1.7.2]</td><td>ko00230</td><td>Purine metabolism</td><td>0.00</td><td>0.00</td><td>0.00</td><td>0.00</td><td>0.00</td><td>77.01</td></tr>
<tr><td>Lizard06Caudovirales_VIRSorter_scaffold_27180-gene_2|269|1306|1037|+|-|-|-|-|Pfam-B_2744|65.6|8.2e-19(+).p1</td><td>ccun:CCUN_1883</td><td>338</td><td>7</td><td>344</td><td>37</td><td>353</td><td>3.75E-175</td><td>71.302</td><td>K06889</td><td>Not Included in Pathway or Brite</td><td>Poorly characterized</td><td>K06889</td><td>uncharacterized protein</td><td>—</td><td>—</td><td>0.00</td><td>0.00</td><td>0.00</td><td>0.00</td><td>38.76</td><td>100.59</td></tr>
<tr><td>Lizard06Caudovirales_VIRSorter_scaffold_27180-gene_4|2130|2858|728|+|Phage_cluster_3510|161.1|5e-</td><td>psyy:DLE54_04960</td><td>238</td><td>1</td><td>238</td><td>52</td><td>289</td><td>5.04E-75</td><td>44.958</td><td>K00973</td><td>Metabolism</td><td>Glycan biosynthesis and metabolism</td><td>rfbA, rmlA, rffH</td><td>glucose-1-phosphate thymidylyltransferase [EC:2.7.7.24]</td><td>ko00521,ko00523,ko00525</td><td>Streptomycin biosynthesis|Polyketide sugar unit biosynthesis|Acarbose and validamycin biosynthesis</td><td>0.00</td><td>0.00</td><td>0.00</td><td>0.00</td><td>0.00</td><td>1.85</td></tr>
<tr><td>Lizard06Caudovirales_VIRSorter_scaffold_68342-gene_2|570|2387|1817|-|Phage_cluster_3071|259.2|9.1e-</td><td>parc:CI960_16865</td><td>607</td><td>1</td><td>604</td><td>2</td><td>586</td><td>0</td><td>77.43</td><td>K02343</td><td>Genetic Information Processing</td><td>Replication and repair</td><td>dnaX</td><td>DNA polymerase III subunit gamma/tau [EC:2.7.7.7]</td><td>ko03030,ko03430,ko03440</td><td>DNA replication|Mismatch repair|Homologous recombination</td><td>0.00</td><td>1261.71</td><td>31.24</td><td>6.28</td><td>0.00</td><td>126.88</td></tr>
<tr><td>Lizard06Caudovirales_VIRSorter_scaffold_75485-gene_1|1|1689|1688|+|gi_66392035_ref_YP_238960.1_|182|1.58e-</td><td>bth:BT_0355</td><td>562</td><td>1</td><td>562</td><td>3</td><td>564</td><td>0</td><td>94.662</td><td>K03307</td><td>Not Included in Pathway or Brite</td><td>Unclassified: signaling and cellular processes</td><td>TC.SSS</td><td>solute:Na+ symporter, SSS family</td><td>—</td><td>—</td><td>0.00</td><td>198699.26</td><td>26707.62</td><td>14318.49</td><td>20853.75</td><td>620396.23</td></tr>
<tr><td>Lizard06Caudovirales_VIRSorter_scaffold_75485-gene_2|1714|2391|677|+|-|-|-|-|Pfam-B_19239|52.4|8.7e-15(+).p1</td><td>bcac:CGC64_10340</td><td>225</td><td>1</td><td>225</td><td>1</td><td>225</td><td>1.23E-142</td><td>83.556</td><td>K03574</td><td>Brite Hierarchies</td><td>Protein families: genetic information processing</td><td>mutT, NUDT15, MTH2</td><td>8-oxo-dGTP diphosphatase [EC:3.6.1.55]</td><td>—</td><td>—</td><td>0.00</td><td>2.60</td><td>0.00</td><td>0.00</td><td>0.00</td><td>2.58</td></tr>
<tr><td>Lizard06Caudovirales_VIRSorter_scaffold_75485-gene_3|2397|3080|683|+|-|-|-|-|Aldolase_II|168.1|2.8e-50(+).p1</td><td>bcae:A4V03_06530</td><td>227</td><td>1</td><td>227</td><td>1</td><td>227</td><td>6.82E-167</td><td>99.119</td><td>K03077</td><td>Metabolism</td><td>Carbohydrate metabolism</td><td>araD, ulaF, sgaE, sgbE</td><td>L-ribulose-5-phosphate 4-epimerase [EC:5.1.3.4]</td><td>ko00040,ko00053</td><td>Pentose and glucuronate interconversions|Ascorbate and aldarate metabolism</td><td>0.00</td><td>24.13</td><td>3.71</td><td>0.00</td><td>0.00</td><td>84.97</td></tr>
<tr><td>Lizard06Caudovirales_VIRSorter_scaffold_4373-gene_1|1|912|911|+|-|-|-|-|Peptidase_M16_C|61.9|1.2e-17(+).p1</td><td>osp:Odosp_1470</td><td>310</td><td>1</td><td>300</td><td>616</td><td>919</td><td>9.39E-55</td><td>37.742</td><td>K07263</td><td>Brite Hierarchies</td><td>Protein families: metabolism</td><td>pqqL</td><td>zinc protease [EC:3.4.24.-]</td><td>—</td><td>—</td><td>72212.59</td><td>5931.32</td><td>0.00</td><td>35796.23</td><td>0.00</td><td>29519.25</td></tr>
<tr><td>Lizard06Caudovirales_VIRSorter_scaffold_50003-gene_4|2236|3087|851|-|Phage_cluster_126|249.0|1.2e-</td><td>ccel:CCDG5_1548</td><td>254</td><td>1</td><td>252</td><td>170</td><td>423</td><td>6.08E-92</td><td>53.937</td><td>K06909</td><td>Not Included in Pathway or Brite</td><td>Unclassified: genetic information processing</td><td>xtmB</td><td>phage terminase large subunit</td><td>—</td><td>—</td><td>0.00</td><td>0.00</td><td>0.00</td><td>22.41</td><td>0.00</td><td>7.70</td></tr>
<tr><td>Lizard06Caudovirales_VIRSorter_scaffold_52496-gene_2|746|1417|671|-|-|-|-|-|MgtC|137.8|3.6e-41(-).p1</td><td>boa:Bovatus_04700</td><td>222</td><td>1</td><td>222</td><td>1</td><td>222</td><td>1.76E-127</td><td>79.73</td><td>K07507</td><td>Brite Hierarchies</td><td>Protein families: signaling and cellular processes</td><td>mgtC</td><td>putative Mg2+ transporter-C (MgtC) family protein</td><td>—</td><td>—</td><td>0.00</td><td>0.00</td><td>0.00</td><td>0.00</td><td>0.00</td><td>2.64</td></tr>
</table>

<table>
<tr><th rowspan="2">Query ORF ID</th><th rowspan="2">Subject gene ID</th><th rowspan="2">Alignment length</th><th rowspan="2">Query start</th><th rowspan="2">Query end</th><th rowspan="2">Subject start</th><th rowspan="2">Subject end</th><th rowspan="2">e-value</th><th rowspan="2">Identity(%)</th><th rowspan="2">KO</th><th rowspan="2">Category</th><th rowspan="2">Subcategory</th><th rowspan="2">Gene name</th><th rowspan="2">Description</th><th rowspan="2">Pathway</th><th rowspan="2">Pathway definition</th><th colspan="6">Abundance (fpkm)</th></tr>
<tr><th>(1) PE-NMC</th><th>(2) PE-NQ1</th><th>(3) PE-NQ2</th><th>(4) PT-SZZ</th><th>(5) PT-ZB</th><th>(6) PT-RT</th></tr>
<tr><td>Lizard06Caudovirales_VIRSorter_scaffold_90091-gene_2|1073|1414|341|+|-|-|-|-|-|-|-|-(+).p1</td><td>slr:L21SP2_1918</td><td>89</td><td>2</td><td>88</td><td>36</td><td>123</td><td>4.04E-12</td><td>41.573</td><td>K09005</td><td>Not Included in Pathway or Brite</td><td>Poorly characterized</td><td>K09005</td><td>uncharacterized protein</td><td>—</td><td>—</td><td>0.00</td><td>0.00</td><td>0.00</td><td>0.00</td><td>0.00</td><td>0.92</td></tr>
<tr><td>Lizard06Caudovirales_VIRSorter_scaffold_32223-gene_1|341|1045|704|-|-|-|-|-|Glucosamine_iso|159.5|1.6e-47(-).p1</td><td>parc:CI960_07190</td><td>228</td><td>2</td><td>229</td><td>3</td><td>229</td><td>7.54E-117</td><td>69.298</td><td>K01057</td><td>Metabolism</td><td>Carbohydrate metabolism</td><td>PGLS, pgl, devB</td><td>6-phosphogluconolactonase [EC:3.1.1.31]</td><td>ko00030</td><td>Pentose phosphate pathway</td><td>211.77</td><td>56.53</td><td>0.00</td><td>0.00</td><td>0.00</td><td>25.97</td></tr>
<tr><td>Lizard06Caudovirales_VIRSorter_scaffold_32223-gene_2|1061|2602|1541|-|Phage_cluster_2040|373.0|3.6e-</td><td>parc:CI960_07185</td><td>486</td><td>23</td><td>508</td><td>2</td><td>486</td><td>0</td><td>80.658</td><td>K00036</td><td>Metabolism</td><td>Carbohydrate metabolism</td><td>G6PD, zwf</td><td>glucose-6-phosphate 1-dehydrogenase [EC:1.1.1.49 1.1.1.363]</td><td>ko00030,ko00480,ko05230</td><td>Pentose phosphate pathway|Glutathione metabolism|Central carbon metabolism in cancer</td><td>0.00</td><td>9.78</td><td>8.39</td><td>0.00</td><td>19.65</td><td>17.62</td></tr>
<tr><td>Lizard06Caudovirales_VIRSorter_scaffold_32223-gene_3|2595|2984|389|-|-|-|-|-|6PGD|150.0|1.6e-44(-).p2</td><td>parc:CI960_07180</td><td>128</td><td>1</td><td>128</td><td>359</td><td>486</td><td>1.36E-72</td><td>83.594</td><td>K00033</td><td>Metabolism</td><td>Carbohydrate metabolism</td><td>PGD, gnd, gntZ</td><td>6-phosphogluconate dehydrogenase [EC:1.1.1.44 1.1.1.343]</td><td>ko00030,ko00480</td><td>Pentose phosphate pathway|Glutathione metabolism</td><td>13.91</td><td>1.71</td><td>0.00</td><td>0.00</td><td>0.00</td><td>0.19</td></tr>
<tr><td>Lizard06Caudovirales_VIRSorter_scaffold_106069-gene_3|2627|2966|339|+|-|-|-|-|-|-|-|-(+).p1</td><td>bxy:BXY_15530</td><td>112</td><td>2</td><td>113</td><td>1</td><td>112</td><td>2.96E-65</td><td>89.286</td><td>K01599</td><td>Metabolism</td><td>Metabolism of cofactors and vitamins</td><td>hemE, UROD</td><td>uroporphyrinogen decarboxylase [EC:4.1.1.37]</td><td>ko00860</td><td>Porphyrin and chlorophyll metabolism</td><td>0.00</td><td>2.82</td><td>0.00</td><td>0.00</td><td>0.00</td><td>5.43</td></tr>
<tr><td>Lizard06Caudovirales_VIRSorter_scaffold_16125-gene_1|29|706|677|-|-|-|-|-|Pfam-B_19239|43.2|5.6e-12(-).p1</td><td>bcac:CGC64_10340</td><td>224</td><td>1</td><td>224</td><td>2</td><td>225</td><td>1.58E-156</td><td>92.411</td><td>K03574</td><td>Brite Hierarchies</td><td>Protein families: genetic information processing</td><td>mutT, NUDT15, MTH2</td><td>8-oxo-dGTP diphosphatase [EC:3.6.1.55]</td><td>—</td><td>—</td><td>13695.49</td><td>43257.74</td><td>9823.49</td><td>0.00</td><td>2157.28</td><td>139537.83</td></tr>
<tr><td>Lizard06Caudovirales_VIRSorter_scaffold_16125-gene_2|732|1742|1010|-|gi_66392035_ref_YP_238960.1_|109|5.01e-</td><td>bxy:BXY_15630</td><td>335</td><td>1</td><td>335</td><td>188</td><td>522</td><td>0</td><td>97.313</td><td>K03307</td><td>Not Included in Pathway or Brite</td><td>Unclassified: signaling and cellular processes</td><td>TC.SSS</td><td>solute:Na+ symporter, SSS family</td><td>—</td><td>—</td><td>1430.44</td><td>5469.46</td><td>1009.44</td><td>9.78</td><td>370.35</td><td>15433.08</td></tr>
<tr><td>Lizard06Caudovirales_VIRSorter_scaffold_16125-gene_3|1739|2257|518|-|gi_66392035_ref_YP_238960.1_|84.7|5.06e-</td><td>bcae:A4V03_06540</td><td>169</td><td>1</td><td>169</td><td>2</td><td>170</td><td>8.42E-107</td><td>95.858</td><td>K03307</td><td>Not Included in Pathway or Brite</td><td>Unclassified: signaling and cellular processes</td><td>TC.SSS</td><td>solute:Na+ symporter, SSS family</td><td>—</td><td>—</td><td>311.44</td><td>573.86</td><td>76.79</td><td>0.00</td><td>53.96</td><td>1629.02</td></tr>
<tr><td>Lizard06Caudovirales_VIRSorter_scaffold_16125-gene_4|2283|2930|647|-|-|-|-|-|Aldose_epim|192.1|2.2e-57(-).p1</td><td>boa:Bovatus_00212</td><td>214</td><td>1</td><td>214</td><td>166</td><td>379</td><td>2.56E-148</td><td>94.393</td><td>K01785</td><td>Metabolism</td><td>Carbohydrate metabolism</td><td>galM, GALM</td><td>aldose 1-epimerase [EC:5.1.3.3]</td><td>ko00010,ko00052</td><td>Glycolysis / Gluconeogenesis|Galactose metabolism</td><td>205.60</td><td>502.07</td><td>50.69</td><td>0.00</td><td>54.81</td><td>1487.17</td></tr>
<tr><td>Lizard06Caudovirales_VIRSorter_scaffold_4077-gene_1|600|2348|1748|-|-|-|-|-|PGM_PMM_I|139.7|7.9e-42(-).p1</td><td>parc:CI960_16915</td><td>581</td><td>1</td><td>581</td><td>2</td><td>582</td><td>0</td><td>95.697</td><td>K01835</td><td>Metabolism</td><td>Carbohydrate metabolism</td><td>pgm</td><td>phosphoglucomutase [EC:5.4.2.2]</td><td>ko00010,ko00030,ko00052,ko00230,ko00500,ko00520,ko00521</td><td>Glycolysis / Gluconeogenesis|Pentose phosphate pathway|Galactose metabolism|Purine metabolism|Starch and sucrose metabolism|Amino sugar and nucleotide sugar metabolism|Streptomycin biosynthesis</td><td>0.00</td><td>12.36</td><td>0.00</td><td>0.00</td><td>0.00</td><td>43.46</td></tr>
<tr><td>Lizard06Caudovirales_VIRSorter_scaffold_39886-gene_1|39|2375|2336|+|Phage_cluster_1607|588.1|3.6e-</td><td>pdi:BDI_0939</td><td>778</td><td>1</td><td>778</td><td>3</td><td>779</td><td>0</td><td>80.463</td><td>K03657</td><td>Genetic Information Processing</td><td>Replication and repair</td><td>uvrD, pcrA</td><td>DNA helicase II / ATP-dependent DNA helicase PcrA [EC:3.6.4.12]</td><td>ko03420,ko03430</td><td>Nucleotide excision repair|Mismatch repair</td><td>0.00</td><td>646.36</td><td>684.81</td><td>0.00</td><td>534.71</td><td>681.21</td></tr>
<tr><td>Lizard06Caudovirales_VIRSorter_scaffold_105975-gene_2|470|913|443|-|-|-|-|-|-|-|-|-(-).p1</td><td>mbf:BHV42_08025</td><td>146</td><td>1</td><td>146</td><td>2</td><td>147</td><td>1.50E-81</td><td>76.027</td><td>K02388</td><td>Cellular Processes</td><td>Cell motility</td><td>flgC</td><td>flagellar basal-body rod protein FlgC</td><td>ko02040</td><td>Flagellar assembly</td><td>0.00</td><td>0.00</td><td>0.00</td><td>7.62</td><td>66.55</td><td>3.14</td></tr>
<tr><td>Lizard06Caudovirales_VIRSorter_scaffold_105975-gene_3|931|1275|344|-|-|-|-|-|-|-|-|-(-).p1</td><td>mbf:BHV42_08020</td><td>114</td><td>1</td><td>113</td><td>2</td><td>115</td><td>2.79E-45</td><td>64.035</td><td>K02387</td><td>Cellular Processes</td><td>Cell motility</td><td>flgB</td><td>flagellar basal-body rod protein FlgB</td><td>ko02040</td><td>Flagellar assembly</td><td>0.00</td><td>0.00</td><td>0.00</td><td>0.00</td><td>0.00</td><td>0.00</td></tr>
<tr><td>Lizard06Caudovirales_VIRSorter_scaffold_105975-gene_4|1339|2052|713|-|-|-|-|-|-|-|-|-(-).p1</td><td>mbf:BHV42_08015</td><td>236</td><td>1</td><td>236</td><td>2</td><td>237</td><td>8.25E-76</td><td>47.034</td><td>K02392</td><td>Cellular Processes</td><td>Cell motility</td><td>flgG</td><td>flagellar basal-body rod protein FlgG</td><td>ko02040</td><td>Flagellar assembly</td><td>0.00</td><td>0.00</td><td>0.00</td><td>5.35</td><td>31.15</td><td>9.92</td></tr>
<tr><td>Lizard06Caudovirales_VIRSorter_scaffold_105975-gene_5|2064|2834|770|-|-|-|-|-|-|-|-|-(-).p1</td><td>mbf:BHV42_08010</td><td>229</td><td>25</td><td>253</td><td>1</td><td>229</td><td>1.04E-100</td><td>60.262</td><td>K02392</td><td>Cellular Processes</td><td>Cell motility</td><td>flgG</td><td>flagellar basal-body rod protein FlgG</td><td>ko02040</td><td>Flagellar assembly</td><td>0.00</td><td>0.00</td><td>0.00</td><td>8.67</td><td>20.21</td><td>2.62</td></tr>
<tr><td>Lizard06Caudovirales_VIRSorter_scaffold_70655-gene_1|20|634|614|+|Phage_cluster_2087|117.8|7.1e-</td><td>pet:PEIBARAKI_5326</td><td>185</td><td>6</td><td>190</td><td>5</td><td>161</td><td>5.81E-28</td><td>35.135</td><td>K07474</td><td>Not Included in Pathway or Brite</td><td>Unclassified: genetic information processing</td><td>xtmA</td><td>phage terminase small subunit</td><td>—</td><td>—</td><td>0.00</td><td>0.00</td><td>0.00</td><td>0.00</td><td>0.00</td><td>147.60</td></tr>
<tr><td>Lizard06Caudovirales_VIRSorter_scaffold_81985-gene_1|1|984|983|-|Phage_cluster_371|138.1|7.7e-41|2|Glyco_hydro_25|89.7|3.8e-26(-).p2</td><td>cls:CXIVA_07150</td><td>352</td><td>5</td><td>327</td><td>6</td><td>345</td><td>1.25E-58</td><td>39.205</td><td>K07273</td><td>Not Included in Pathway or Brite</td><td>Unclassified: signaling and cellular processes</td><td>acm</td><td>lysozyme</td><td>—</td><td>—</td><td>0.00</td><td>0.00</td><td>0.00</td><td>0.00</td><td>0.00</td><td>8855.77</td></tr>
<tr><td>Lizard06Caudovirales_VIRSorter_scaffold_84399-gene_1|266|1465|1199|+|-|-|-|-|AAA_14|93.7|1.5e-27(+).p1</td><td>bth:BT_2405</td><td>399</td><td>1</td><td>399</td><td>1</td><td>399</td><td>0</td><td>83.96</td><td>K07133</td><td>Not Included in Pathway or Brite</td><td>Poorly characterized</td><td>K07133</td><td>uncharacterized protein</td><td>—</td><td>—</td><td>0.00</td><td>128.21</td><td>33.47</td><td>0.00</td><td>0.00</td><td>44.39</td></tr>
<tr><td>Lizard06Caudovirales_VIRSorter_scaffold_11010-gene_2|331|702|371|+|-|-|-|-|Chromate_transp|97.9|8.8e-29(+).p1</td><td>carg:RSJ17_14980</td><td>122</td><td>1</td><td>122</td><td>34</td><td>155</td><td>2.71E-32</td><td>49.18</td><td>K07240</td><td>Brite Hierarchies</td><td>Protein families: signaling and cellular processes</td><td>chrA</td><td>chromate transporter</td><td>—</td><td>—</td><td>0.00</td><td>0.00</td><td>0.00</td><td>32.44</td><td>0.00</td><td>1.49</td></tr>
<tr><td>Lizard06Caudovirales_VIRSorter_scaffold_11010-gene_3|803|1363|560|+|-|-|-|-|Chromate_transp|147.0|7.5e-44(+).p1</td><td>carg:RSJ17_14985</td><td>186</td><td>1</td><td>186</td><td>1</td><td>185</td><td>1.74E-57</td><td>48.925</td><td>K07240</td><td>Brite Hierarchies</td><td>Protein families: signaling and cellular processes</td><td>chrA</td><td>chromate transporter</td><td>—</td><td>—</td><td>0.00</td><td>0.00</td><td>0.00</td><td>0.00</td><td>0.00</td><td>4.29</td></tr>
<tr><td>Lizard06Caudovirales_VIRSorter_scaffold_11010-gene_4|1484|2743|1259|-|Phage_cluster_244|181.3|4.6e-</td><td>mbf:BHV42_07935</td><td>418</td><td>1</td><td>418</td><td>374</td><td>790</td><td>0</td><td>70.813</td><td>K02470</td><td>Brite Hierarchies</td><td>Protein families: genetic information processing</td><td>gyrB</td><td>DNA gyrase subunit B [EC:5.6.2.2]</td><td>—</td><td>—</td><td>0.00</td><td>0.00</td><td>0.00</td><td>9.65</td><td>14.05</td><td>7.63</td></tr>
<tr><td>Lizard06Caudovirales_VIRSorter_scaffold_78849-gene_2|483|1187|704|+|Phage_cluster_197|263.9|4.2e-79|1|Pfam-</td><td>dru:Desru_1077</td><td>234</td><td>1</td><td>234</td><td>1</td><td>234</td><td>4.30E-90</td><td>59.402</td><td>K02334</td><td>Not Included in Pathway or Brite</td><td>Unclassified: metabolism</td><td>dpo</td><td>DNA polymerase bacteriophage-type [EC:2.7.7.7]</td><td>—</td><td>—</td><td>0.00</td><td>0.00</td><td>0.00</td><td>0.00</td><td>0.00</td><td>14.26</td></tr>
<tr><td>Lizard06Caudovirales_VIRSorter_scaffold_78849-gene_3|1209|2432|1223|+|Phage_cluster_197|530.7|7.6e-160|1|Pfam-</td><td>drm:Dred_1191</td><td>407</td><td>2</td><td>407</td><td>244</td><td>650</td><td>3.78E-180</td><td>62.899</td><td>K02334</td><td>Not Included in Pathway or Brite</td><td>Unclassified: metabolism</td><td>dpo</td><td>DNA polymerase bacteriophage-type [EC:2.7.7.7]</td><td>—</td><td>—</td><td>0.00</td><td>0.00</td><td>0.00</td><td>0.00</td><td>0.00</td><td>6.10</td></tr>
<tr><td>Lizard06Caudovirales_VIRSorter_scaffold_12303-gene_1|2|316|314|+|-|-|-|-|Peptidase_S49|58.9|9.4e-17(+).p1</td><td>parc:CI960_02975</td><td>96</td><td>9</td><td>104</td><td>172</td><td>267</td><td>2.78E-39</td><td>67.708</td><td>K04773</td><td>Brite Hierarchies</td><td>Protein families: metabolism</td><td>sppA</td><td>protease IV [EC:3.4.21.-]</td><td>—</td><td>—</td><td>0.00</td><td>0.00</td><td>0.00</td><td>0.00</td><td>0.00</td><td>983.97</td></tr>
<tr><td>Lizard06Caudovirales_VIRSorter_scaffold_12303-gene_2|356|1261|905|+|Phage_cluster_714|205.0|3.5e-</td><td>parc:CI960_02975</td><td>301</td><td>1</td><td>301</td><td>282</td><td>582</td><td>9.93E-162</td><td>73.422</td><td>K04773</td><td>Brite Hierarchies</td><td>Protein families: metabolism</td><td>sppA</td><td>protease IV [EC:3.4.21.-]</td><td>—</td><td>—</td><td>0.00</td><td>16.66</td><td>0.00</td><td>0.00</td><td>0.00</td><td>23.49</td></tr>
<tr><td>Lizard06Caudovirales_VIRSorter_scaffold_12303-gene_3|1508|2404|896|+|-|-|-|-|LpxK|191.5|3.5e-57(+).p1</td><td>parc:CI960_02970</td><td>293</td><td>2</td><td>294</td><td>72</td><td>364</td><td>1.22E-131</td><td>63.481</td><td>K00912</td><td>Metabolism</td><td>Glycan biosynthesis and metabolism</td><td>lpxK</td><td>tetraacyldisaccharide 4'-kinase [EC:2.7.1.130]</td><td>ko00540</td><td>Lipopolysaccharide biosynthesis</td><td>0.00</td><td>0.00</td><td>0.00</td><td>0.00</td><td>13.83</td><td>4.24</td></tr>
<tr><td>Lizard06Caudovirales_VIRSorter_scaffold_105497-gene_1|66|1049|983|+|-|-|-|-|BATS|74.8|6.9e-22(+).p1</td><td>pdi:BDI_1913</td><td>326</td><td>2</td><td>327</td><td>3</td><td>327</td><td>1.33E-169</td><td>69.632</td><td>K01012</td><td>Metabolism</td><td>Metabolism of cofactors and vitamins</td><td>bioB</td><td>biotin synthase [EC:2.8.1.6]</td><td>ko00780</td><td>Biotin metabolism</td><td>0.00</td><td>112.34</td><td>0.00</td><td>0.00</td><td>0.00</td><td>186.36</td></tr>
<tr><td>Lizard06Caudovirales_VIRSorter_scaffold_105497-gene_2|1046|2320|1274|+|Phage_cluster_15111|66.5|3.2e-</td><td>pdi:BDI_1914</td><td>420</td><td>1</td><td>420</td><td>1</td><td>420</td><td>0</td><td>77.857</td><td>K00833</td><td>Metabolism</td><td>Metabolism of cofactors and vitamins</td><td>bioA</td><td>adenosylmethionine---8-amino-7-oxononanoate aminotransferase [EC:2.6.1.62]</td><td>ko00780</td><td>Biotin metabolism</td><td>0.00</td><td>15.59</td><td>0.00</td><td>0.00</td><td>0.00</td><td>8.94</td></tr>
<tr><td>Lizard06Caudovirales_VIRSorter_scaffold_98682-gene_1|53|430|377|-|-|-|-|-|-|-|-|-(-).p1</td><td>mbf:BHV42_03035</td><td>124</td><td>1</td><td>124</td><td>2</td><td>125</td><td>4.52E-32</td><td>44.355</td><td>K07015</td><td>Not Included in Pathway or Brite</td><td>Poorly characterized</td><td>K07015</td><td>uncharacterized protein</td><td>—</td><td>—</td><td>0.00</td><td>0.00</td><td>0.00</td><td>0.00</td><td>0.00</td><td>46.41</td></tr>
<tr><td>Lizard06Caudovirales_VIRSorter_scaffold_98682-gene_2|434|1702|1268|-|-|-|-|-|Peptidase_U32|301.0|6.7e-91(-).p1</td><td>mbf:BHV42_03040</td><td>417</td><td>2</td><td>418</td><td>2</td><td>414</td><td>0</td><td>59.952</td><td>K08303</td><td>Human Diseases</td><td>Infectious disease: bacterial</td><td>K08303</td><td>putative protease [EC:3.4.-.-]</td><td>ko05120</td><td>Epithelial cell signaling in Helicobacter pylori infection</td><td>0.00</td><td>0.00</td><td>0.00</td><td>0.00</td><td>0.00</td><td>34.01</td></tr>
<tr><td>Lizard06Caudovirales_VIRSorter_scaffold_84876-gene_2|235|1143|908|+|-|-|-|-|HlyD_2|82.8|4.7e-24(+).p1</td><td>bfs:BF9343_3560</td><td>296</td><td>2</td><td>295</td><td>1</td><td>295</td><td>2.68E-146</td><td>69.932</td><td>K15727</td><td>Brite Hierarchies</td><td>Protein families: signaling and cellular processes</td><td>czcB, cusB, cnrB</td><td>membrane fusion protein, heavy metal efflux system</td><td>—</td><td>—</td><td>0.00</td><td>18.93</td><td>0.00</td><td>0.00</td><td>44.37</td><td>50.25</td></tr>
<tr><td>Lizard06Caudovirales_VIRSorter_scaffold_84330-gene_1|183|929|746|+|Phage_cluster_3079|223.7|2.7e-</td><td>mbf:BHV42_00615</td><td>240</td><td>9</td><td>248</td><td>4</td><td>243</td><td>1.78E-154</td><td>88.333</td><td>K11329</td><td>Environmental Information Processing</td><td>Signal transduction</td><td>rpaB</td><td>two-component system, OmpR family, response regulator RpaB</td><td>ko02020</td><td>Two-component system</td><td>0.00</td><td>0.00</td><td>0.00</td><td>19.56</td><td>113.95</td><td>45.70</td></tr>
<tr><td>Lizard06Caudovirales_VIRSorter_scaffold_58311-gene_1|151|1017|866|+|Phage_cluster_3510|290.9|1.1e-</td><td>bcac:CGC64_17965</td><td>288</td><td>1</td><td>288</td><td>1</td><td>288</td><td>0</td><td>87.847</td><td>K00973</td><td>Metabolism</td><td>Glycan biosynthesis and metabolism</td><td>rfbA, rmlA, rffH</td><td>glucose-1-phosphate thymidylyltransferase [EC:2.7.7.24]</td><td>ko00521,ko00523,ko00525</td><td>Streptomycin biosynthesis|Polyketide sugar unit biosynthesis|Acarbose and validamycin biosynthesis</td><td>0.00</td><td>206.22</td><td>0.00</td><td>71.12</td><td>0.00</td><td>94.49</td></tr>
<tr><td>Lizard06Caudovirales_VIRSorter_scaffold_58311-gene_2|1035|2174|1139|+|Phage_cluster_398|308.7|5.8e-</td><td>bfs:BF9343_3411</td><td>379</td><td>1</td><td>379</td><td>1</td><td>379</td><td>0</td><td>96.042</td><td>K01710</td><td>Metabolism</td><td>Glycan biosynthesis and metabolism</td><td>rfbB, rmlB, rffG</td><td>dTDP-glucose 4,6-dehydratase [EC:4.2.1.46]</td><td>ko00521,ko00523,ko00525,ko01055</td><td>Streptomycin biosynthesis|Polyketide sugar unit biosynthesis|Acarbose and validamycin biosynthesis|Biosynthesis of vancomycin group antibiotics</td><td>0.00</td><td>68.77</td><td>0.00</td><td>0.00</td><td>0.00</td><td>23.77</td></tr>
<tr><td>Lizard06Caudovirales_VIRSorter_scaffold_58311-gene_3|2164|2499|335|-|-|-|-|-|-|-|-|-(-).p1</td><td>bfs:BF9343_3410</td><td>110</td><td>1</td><td>110</td><td>189</td><td>298</td><td>2.76E-57</td><td>76.364</td><td>K02548</td><td>Metabolism</td><td>Metabolism of cofactors and vitamins</td><td>menA</td><td>1,4-dihydroxy-2-naphthoate polyprenyltransferase [EC:2.5.1.74]</td><td>ko00130</td><td>Ubiquinone and other terpenoid-quinone biosynthesis</td><td>0.00</td><td>4.11</td><td>0.00</td><td>0.00</td><td>0.00</td><td>1.14</td></tr>
<tr><td>Lizard06Caudovirales_VIRSorter_scaffold_8327-gene_4|1167|2491|1324|+|Phage_cluster_38|231.1|3.1e-</td><td>bdh:GV66_19085</td><td>450</td><td>1</td><td>441</td><td>2</td><td>422</td><td>1.43E-89</td><td>38.889</td><td>K19789</td><td>Brite Hierarchies</td><td>Protein families: genetic information processing</td><td>radD</td><td>DNA repair protein RadD</td><td>—</td><td>—</td><td>0.00</td><td>0.00</td><td>0.00</td><td>6.13</td><td>214.43</td><td>62.82</td></tr>
<tr><td>Lizard06Caudovirales_VIRSorter_scaffold_15855-gene_1|72|1022|950|+|Phage_cluster_2703|391.6|6.3e-</td><td>mbf:BHV42_06695</td><td>312</td><td>1</td><td>312</td><td>101</td><td>412</td><td>0</td><td>78.205</td><td>K00600</td><td>Metabolism</td><td>Carbohydrate metabolism</td><td>glyA, SHMT</td><td>glycine hydroxymethyltransferase [EC:2.1.2.1]</td><td>ko00260,ko00460,ko00630,ko00670,ko00680,ko01523</td><td>Glycine, serine and threonine metabolism|Cyanoamino acid metabolism|Glyoxylate and dicarboxylate metabolism|One carbon pool by</td><td>0.00</td><td>0.00</td><td>0.00</td><td>0.00</td><td>0.00</td><td>211.83</td></tr>
<tr><td>Lizard06Caudovirales_VIRSorter_scaffold_15855-gene_2|1022|2065|1043|+|-|-|-|-|Glycos_transf_4|143.8|7.2e-43(+).p1</td><td>mbf:BHV42_06700</td><td>346</td><td>4</td><td>347</td><td>3</td><td>343</td><td>1.05E-154</td><td>65.607</td><td>K02851</td><td>Metabolism</td><td>Glycan biosynthesis and metabolism</td><td>wecA, tagO, rfe</td><td>UDP-GlcNAc:undecaprenyl-phosphate/decaprenyl-phosphate GlcNAc-1-phosphate transferase [EC:2.7.8.33 2.7.8.35]</td><td>ko00572</td><td>Arabinogalactan biosynthesis - Mycobacterium</td><td>0.00</td><td>0.00</td><td>0.00</td><td>0.00</td><td>0.00</td><td>12.52</td></tr>
<tr><td>Lizard06Caudovirales_VIRSorter_scaffold_15029-gene_2|600|1496|896|+|Phage_cluster_298|172.0|3.7e-</td><td>sclo:SCLO_1023290</td><td>267</td><td>36</td><td>297</td><td>39</td><td>304</td><td>4.30E-74</td><td>46.067</td><td>K06400</td><td>Not Included in Pathway or Brite</td><td>Unclassified: signaling and cellular processes</td><td>spoIVCA</td><td>site-specific DNA recombinase</td><td>—</td><td>—</td><td>0.00</td><td>0.00</td><td>0.00</td><td>5.97</td><td>34.76</td><td>45.10</td></tr>
<tr><td>Lizard06Caudovirales_VIRSorter_scaffold_16163-gene_3|1077|1997|920|+|Phage_cluster_415|269.5|5.8e-</td><td>cld:CLSPO_c24690</td><td>297</td><td>12</td><td>304</td><td>36</td><td>330</td><td>2.24E-81</td><td>43.771</td><td>K04763</td><td>Brite Hierarchies</td><td>Protein families: genetic information processing</td><td>xerD</td><td>integrase/recombinase XerD</td><td>—</td><td>—</td><td>0.00</td><td>9.64</td><td>0.00</td><td>249.28</td><td>19.36</td><td>26.50</td></tr>
<tr><td>Lizard06Caudovirales_VIRSorter_scaffold_56611-gene_3|1170|2390|1220|+|Phage_cluster_7824|40.2|2.7e-</td><td>bhf:C3V43_00500</td><td>407</td><td>1</td><td>406</td><td>36</td><td>437</td><td>0</td><td>83.047</td><td>K08307</td><td>Brite Hierarchies</td><td>Protein families: metabolism</td><td>mltD, dniR</td><td>membrane-bound lytic murein transglycosylase D [EC:4.2.2.-]</td><td>—</td><td>—</td><td>0.00</td><td>32.95</td><td>0.00</td><td>21.42</td><td>8.91</td><td>10.51</td></tr>
<tr><td>Lizard06Caudovirales_VIRSorter_scaffold_10943-gene_1|44|616|572|-|gi_448260370_ref_YP_007348468.1_|92.0|2.79e-</td><td>bhl:Bache_3093</td><td>186</td><td>1</td><td>186</td><td>98</td><td>283</td><td>2.48E-117</td><td>83.871</td><td>K00067</td><td>Metabolism</td><td>Glycan biosynthesis and metabolism</td><td>rfbD, rmlD</td><td>dTDP-4-dehydrorhamnose reductase [EC:1.1.1.133]</td><td>ko00521,ko00523</td><td>Streptomycin biosynthesis|Polyketide sugar unit biosynthesis</td><td>0.00</td><td>0.00</td><td>0.00</td><td>325.42</td><td>0.00</td><td>134.18</td></tr>
<tr><td>Lizard06Caudovirales_VIRSorter_scaffold_67187-gene_4|1694|2092|398|-|Phage_cluster_20|175.7|1.4e-</td><td>bvu:BVU_2811</td><td>131</td><td>1</td><td>131</td><td>2</td><td>132</td><td>5.71E-81</td><td>84.733</td><td>K08640</td><td>Brite Hierarchies</td><td>Protein families: metabolism</td><td>E3.4.17.14</td><td>zinc D-Ala-D-Ala carboxypeptidase [EC:3.4.17.14]</td><td>—</td><td>—</td><td>0.00</td><td>2.63</td><td>2.63</td><td>2.11</td><td>0.00</td><td>2.90</td></tr>
<tr><td>Lizard06Caudovirales_VIRSorter_scaffold_63232-gene_1|253|714|461|+|gi_61806062_ref_YP_214422.1_|126|1.10e-</td><td>pdi:BDI_1885</td><td>153</td><td>1</td><td>153</td><td>355</td><td>507</td><td>1.31E-96</td><td>91.503</td><td>K00602</td><td>Metabolism</td><td>Nucleotide metabolism</td><td>purH</td><td>phosphoribosylaminoimidazolecarboxamide formyltransferase / IMP cyclohydrolase [EC:2.1.2.3 3.5.4.10]</td><td>ko00230,ko00670,ko01523</td><td>Purine metabolism|One carbon pool by folate|Antifolate resistance</td><td>0.00</td><td>5.86</td><td>0.00</td><td>0.00</td><td>0.00</td><td>11.67</td></tr>
<tr><td>Lizard06Caudovirales_VIRSorter_scaffold_63232-gene_2|778|1797|1019|+|-|-|-|-|MreB_Mbl|403.6|7.8e-122(+).p1</td><td>tfo:BFO_3351</td><td>339</td><td>1</td><td>339</td><td>1</td><td>339</td><td>0</td><td>87.611</td><td>K03569</td><td>Brite Hierarchies</td><td>Protein families: genetic information processing</td><td>mreB</td><td>rod shape-determining protein MreB and related proteins</td><td>—</td><td>—</td><td>232.05</td><td>11.44</td><td>0.00</td><td>0.00</td><td>13.40</td><td>27.19</td></tr>
<tr><td>Lizard06Caudovirales_VIRSorter_scaffold_63232-gene_3|1967|2341|374|+|-|-|-|-|-|-|-|-(+).p1</td><td>pdi:BDI_1883</td><td>124</td><td>1</td><td>124</td><td>26</td><td>149</td><td>1.74E-68</td><td>81.452</td><td>K03570</td><td>Brite Hierarchies</td><td>Protein families: genetic information processing</td><td>mreC</td><td>rod shape-determining protein MreC</td><td>—</td><td>—</td><td>18.36</td><td>2.26</td><td>0.00</td><td>0.00</td><td>0.00</td><td>3.00</td></tr>
<tr><td>Lizard06Caudovirales_VIRSorter_scaffold_97319-gene_2|260|568|308|-|Phage_cluster_749|60.5|2.6e-</td><td>bcel:BcellWH2_01523</td><td>101</td><td>1</td><td>101</td><td>62</td><td>162</td><td>2.68E-53</td><td>78.218</td><td>K00287</td><td>Metabolism</td><td>Metabolism of cofactors and vitamins</td><td>DHFR, folA</td><td>dihydrofolate reductase [EC:1.5.1.3]</td><td>ko00670,ko00790,ko01523</td><td>One carbon pool by folate|Folate biosynthesis|Antifolate resistance</td><td>0.00</td><td>79.84</td><td>0.00</td><td>0.00</td><td>0.00</td><td>5.68</td></tr>
<tr><td>Lizard06Caudovirales_VIRSorter_scaffold_16551-gene_4|1179|2150|971|-|Phage_cluster_6731|301.3|1.5e-90|2|-|-|-(-).p1</td><td>mef:MSWH1_2818</td><td>317</td><td>1</td><td>308</td><td>6</td><td>292</td><td>7.75E-26</td><td>28.076</td><td>K07097</td><td>Not Included in Pathway or Brite</td><td>Poorly characterized</td><td>K07097</td><td>uncharacterized protein</td><td>—</td><td>—</td><td>0.00</td><td>0.00</td><td>0.00</td><td>0.00</td><td>291.85</td><td>50.49</td></tr>
<tr><td>Lizard06Caudovirales_VIRSorter_scaffold_73398-gene_3|724|1491|767|+|Phage_cluster_579|135.6|3.1e-</td><td>cle:Clole_0990</td><td>171</td><td>1</td><td>171</td><td>1</td><td>164</td><td>6.29E-62</td><td>56.725</td><td>K01447</td><td>Not Included in Pathway or Brite</td><td>Unclassified: metabolism</td><td>xlyAB</td><td>N-acetylmuramoyl-L-alanine amidase [EC:3.5.1.28]</td><td>—</td><td>—</td><td>0.00</td><td>0.00</td><td>0.00</td><td>0.00</td><td>0.00</td><td>10.19</td></tr>
<tr><td>Lizard06Caudovirales_VIRSorter_scaffold_63301-gene_2|446|1591|1145|-|Phage_cluster_3700|62.7|5.1e-</td><td>pdt:Prede_2381</td><td>379</td><td>1</td><td>379</td><td>2</td><td>380</td><td>2.38E-169</td><td>58.839</td><td>K07486</td><td>Not Included in Pathway or Brite</td><td>Unclassified: genetic information processing</td><td>K07486</td><td>transposase</td><td>—</td><td>—</td><td>890.51</td><td>236.06</td><td>139.73</td><td>1067.47</td><td>210.41</td><td>205.18</td></tr>
<tr><td>Lizard06Caudovirales_VIRSorter_scaffold_94157-gene_3|661|1890|1229|+|gi_61806062_ref_YP_214422.1_|239|6.55e-</td><td>mbf:BHV42_03670</td><td>413</td><td>1</td><td>409</td><td>98</td><td>501</td><td>2.32E-138</td><td>50.605</td><td>K00602</td><td>Metabolism</td><td>Nucleotide metabolism</td><td>purH</td><td>phosphoribosylaminoimidazolecarboxamide formyltransferase / IMP cyclohydrolase [EC:2.1.2.3 3.5.4.10]</td><td>ko00230,ko00670,ko01523</td><td>Purine metabolism|One carbon pool by folate|Antifolate resistance</td><td>0.00</td><td>0.00</td><td>0.00</td><td>27.08</td><td>31.55</td><td>10.42</td></tr>
<tr><td>Lizard06Caudovirales_VIRSorter_scaffold_62698-gene_1|1|1039|1038|-|Phage_cluster_10704|88.1|8.5e-26|2|Pfam-</td><td>bcm:Bcenmc03_1893</td><td>233</td><td>125</td><td>345</td><td>224</td><td>444</td><td>2.61E-39</td><td>39.914</td><td>K02314</td><td>Genetic Information Processing</td><td>Replication and repair</td><td>dnaB</td><td>replicative DNA helicase [EC:3.6.4.12]</td><td>ko03030,ko04112</td><td>DNA replication|Cell cycle - Caulobacter</td><td>0.00</td><td>0.00</td><td>0.00</td><td>0.00</td><td>0.00</td><td>7379.81</td></tr>
<tr><td>Lizard06Caudovirales_VIRSorter_scaffold_79290-gene_4|778|1509|731|-|Phage_cluster_164|159.0|3.1e-</td><td>rus:RBI_II00521</td><td>223</td><td>2</td><td>223</td><td>6</td><td>221</td><td>8.55E-48</td><td>39.462</td><td>K07273</td><td>Not Included in Pathway or Brite</td><td>Unclassified: signaling and cellular processes</td><td>acm</td><td>lysozyme</td><td>—</td><td>—</td><td>0.00</td><td>0.00</td><td>0.00</td><td>0.00</td><td>0.00</td><td>18.97</td></tr>
<tr><td>Lizard06Caudovirales_VIRSorter_scaffold_7988-gene_2|255|1370|1115|-</td><td>bhl:Bache_0801</td><td>369</td><td>1</td><td>369</td><td>2</td><td>370</td><td>0</td><td>79.675</td><td>K04096</td><td>Not Included in Pathway or Brite</td><td>Unclassified: genetic information processing</td><td>smf</td><td>DNA processing protein</td><td>—</td><td>—</td><td>283.19</td><td>610.58</td><td>174.56</td><td>112.30</td><td>81.78</td><td>50.16</td></tr>
<tr><td>Lizard06Caudovirales_VIRSorter_scaffold_7988-gene_3|1363|1770|407|-|-|-|-|-|-|-|-|-(-).p1</td><td>bcac:CGC64_03185</td><td>132</td><td>1</td><td>132</td><td>2</td><td>133</td><td>4.63E-88</td><td>93.182</td><td>K07107</td><td>Not Included in Pathway or Brite</td><td>Unclassified: metabolism</td><td>ybgC</td><td>acyl-CoA thioester hydrolase [EC:3.1.2.-]</td><td>—</td><td>—</td><td>0.00</td><td>10.88</td><td>0.00</td><td>0.00</td><td>0.00</td><td>1.44</td></tr>
<tr><td>Lizard06Caudovirales_VIRSorter_scaffold_58544-gene_2|137|553|416|+|Phage_cluster_405|92.5|3.9e-</td><td>mbf:BHV42_03625</td><td>138</td><td>1</td><td>138</td><td>1</td><td>138</td><td>6.27E-63</td><td>71.014</td><td>K10112</td><td>Environmental Information Processing</td><td>Membrane transport</td><td>msmX, msmK, malK, sugC, ggtA, msiK</td><td>multiple sugar transport system ATP-binding protein</td><td>ko02010</td><td>ABC transporters</td><td>0.00</td><td>0.00</td><td>0.00</td><td>0.00</td><td>0.00</td><td>7.18</td></tr>
<tr><td>Lizard06Caudovirales_VIRSorter_scaffold_58544-gene_3|728|1192|464|+|-|-|-|-|-|-|-|-(+).p1</td><td>mbf:BHV42_03625</td><td>154</td><td>1</td><td>154</td><td>198</td><td>331</td><td>2.96E-34</td><td>46.753</td><td>K10112</td><td>Environmental Information Processing</td><td>Membrane transport</td><td>msmX, msmK, malK, sugC, ggtA, msiK</td><td>multiple sugar transport system ATP-binding protein</td><td>ko02010</td><td>ABC transporters</td><td>0.00</td><td>0.00</td><td>0.00</td><td>0.00</td><td>0.00</td><td>0.68</td></tr>
<tr><td>Lizard06Caudovirales_VIRSorter_scaffold_58544-gene_4|1257|2102|845|+|-|-|-|-|tRNA_Me_trans|314.6|1.1e-94(+).p1</td><td>mbf:BHV42_00535</td><td>274</td><td>1</td><td>273</td><td>1</td><td>274</td><td>2.12E-102</td><td>55.474</td><td>K00566</td><td>Genetic Information Processing</td><td>Folding, sorting and degradation</td><td>mnmA, trmU</td><td>tRNA-uridine 2-sulfurtransferase [EC:2.8.1.13]</td><td>ko04122</td><td>Sulfur relay system</td><td>57.45</td><td>0.00</td><td>14.16</td><td>0.00</td><td>0.00</td><td>2.74</td></tr>
</table>

Query ORF ID	Subject gene ID	Alignment length	Query start	Query end	Subject start	Subject end	e-value	Identity(%)	KO	Category	Subcategory	Gene name	Description	Pathway	Pathway definition	Abundance (fpkm)					
																(1) PE-NMC	(2) PE-NQ1	(3) PE-NQ2	(4) PT-SZZ	(5) PT-ZB	(6) PT-RT
Lizard06Caudovirales_VIRSorter_scaffold_36371-gene_1|33|980|947|-|-|-|-|PfkB|107.5|1.3e-31(-).p1	bhl:Bache_0616	304	1	304	2	305	0	85.197	**K00847**	Metabolism	Carbohydrate metabolism	E2.7.1.4, scrK	fructokinase [EC:2.7.1.4]	ko00051,ko00500,ko00520	Fructose and mannose metabolism|Starch and sucrose metabolism|Amino sugar and nucleotide sugar metabolism	2188.26	21298.84	2427.97	1084.73	631.93	432.35
Lizard06Caudovirales_VIRSorter_scaffold_36371-gene_2|968|1573|605|-|Phage_cluster_5905|114.1|9.6e-	bhl:Bache_0615	200	1	200	2	201	3.34E-130	95.5	**K06041**	Metabolism	Glycan biosynthesis and metabolism	kdsD, kpsF	arabinose-5-phosphate isomerase [EC:5.3.1.13]	ko00540	Lipopolysaccharide biosynthesis	74.60	349.26	18.39	40.68	0.00	8.13
Lizard06Caudovirales_VIRSorter_scaffold_34098-gene_2|871|1509|638|-|-|-|-|-|Cauli_VI|67.3|1.6e-19(-).p1	bcac:CGC64_04550	211	1	211	2	209	1.32E-125	81.991	**K03469**	Genetic Information Processing	Replication and repair	rnhA, RNASEH1	ribonuclease HI [EC:3.1.26.4]	ko03030	DNA replication	41.45	314.95	0.00	0.00	0.00	32.20
Lizard06Caudovirales_VIRSorter_scaffold_34098-gene_3|1627|1928|301|+|-|-|-|-|-|-|-|-(+).p1	bfs:BF9343_0189	100	1	100	1	100	4.98E-55	80	**K07078**	Not Included in Pathway or Brite	Poorly characterized	K07078	uncharacterized protein	—	—	0.00	16.41	0.00	0.00	0.00	4.84
Lizard06Caudovirales_VIRSorter_scaffold_53018-gene_3|641|1606|965|-|Phage_cluster_3700|98.1|9.3e-	ori:Q785_11155	316	2	317	3	318	0	76.266	**K07486**	Not Included in Pathway or Brite	Unclassified: genetic information processing	K07486	transposase	—	—	0.00	23.13	0.00	33.51	16.27	28.40
Lizard06Caudovirales_VIRSorter_scaffold_22335-gene_1|3|875|872|+|-|-|-|-|PFL|307.3|4e-92(+).p1	pdi:BDI_0786	267	1	267	195	461	0	92.135	**K23121**	Metabolism	Amino acid metabolism	pflD	trans-4-hydroxy-L-proline dehydratase [EC:4.2.1.172]	ko00330	Arginine and proline metabolism	0.00	447320.48	14837.57	8352.45	10426.88	116109.05
Lizard06Caudovirales_VIRSorter_scaffold_22335-gene_2|826|1536|710|+|Phage_cluster_1262|50.5|3.9e-	pdi:BDI_0786	236	1	236	575	810	2.70E-151	89.407	**K23121**	Metabolism	Amino acid metabolism	pflD	trans-4-hydroxy-L-proline dehydratase [EC:4.2.1.172]	ko00330	Arginine and proline metabolism	131.14	2418.13	75.45	60.67	63.12	458.63
Lizard06Caudovirales_VIRSorter_scaffold_22335-gene_3|1523|1923|400|+|-|-|-|-|Fer4_10|43.2|4.8e-12(+).p1	parc:CI960_07855	124	10	133	2	125	2.70E-64	75	**K04069**	Not Included in Pathway or Brite	Unclassified: metabolism	pflA, pflC, pflE	pyruvate formate lyase activating enzyme [EC:1.97.1.4]	—	—	118.54	675.70	20.46	14.10	27.39	98.20
Lizard06Caudovirales_VIRSorter_scaffold_67483-gene_2|911|1303|392|-|Phage_cluster_11858|52.0|1.1e-14|1|-|-|-(-).p1	lhk:LHK_01635	115	9	121	5	119	4.97E-13	27.826	**K06903**	Not Included in Pathway or Brite	Poorly characterized	K06903	uncharacterized protein	—	—	0.00	0.00	0.00	0.00	0.00	1.08
Lizard06Caudovirales_VIRSorter_scaffold_104467-gene_2|684|1367|683|+|Phage_cluster_289|247.6|3.8e-	mev:Metev_1166	227	2	227	73	299	8.81E-76	50.661	**K07496**	Not Included in Pathway or Brite	Unclassified: genetic information processing	K07496	putative transposase	—	—	0.00	0.00	0.00	0.00	259.15	622.90
Lizard06Caudovirales_VIRSorter_scaffold_78013-gene_3|555|1763|1208|+|Phage_cluster_289|428.0|6.3e-	smon:AWR27_24320	369	1	369	1	367	1.74E-131	50.678	**K07496**	Not Included in Pathway or Brite	Unclassified: genetic information processing	K07496	putative transposase	—	—	0.00	0.00	0.00	0.00	432.10	145.38
Lizard06Caudovirales_VIRSorter_scaffold_69999-gene_4|1389|1832|443|+|Phage_cluster_740|116.8|1.8e-34|2|-|-|-(+).p1	pbd:PBOR_32655	140	4	143	5	143	1.01E-26	42.857	**K06907**	Not Included in Pathway or Brite	Poorly characterized	K06907	uncharacterized protein	—	—	0.00	0.00	0.00	0.00	0.00	2.48
Lizard06Caudovirales_VIRSorter_scaffold_66821-gene_2|721|1329|608|+|gi_34419448_ref_NP_899461.1_|78.2|2.63e-	osp:Odosp_1082	193	9	201	2	194	1.56E-92	70.984	**K03811**	Brite Hierarchies	Protein families: signaling and cellular processes	pnuC	nicotinamide mononucleotide transporter	—	—	0.00	0.00	0.00	0.00	0.00	10.24
Lizard06Caudovirales_VIRSorter_scaffold_66821-gene_3|1313|1696|383|+|-|-|-|-|TPK_catalytic|81.1|9.2e-24(+).p1	osp:Odosp_1081	125	1	125	1	125	1.25E-47	62.4	**K00949**	Metabolism	Metabolism of cofactors and vitamins	thiN, TPK1, THI80	thiamine pyrophosphokinase [EC:2.7.6.2]	ko00730	Thiamine metabolism	0.00	0.00	0.00	0.00	0.00	1.50
Lizard06Caudovirales_VIRSorter_scaffold_17139-gene_1|77|481|404|+|-|-|-|-|ACT_4|74.0|1.8e-21(+).p1	bfs:BF9343_0658	134	1	134	614	747	7.63E-82	91.791	**K01139**	Metabolism	Nucleotide metabolism	spoT	GTP diphosphokinase / guanosine-3',5'-bis(diphosphate) 3'-diphosphatase [EC:2.7.6.5 3.1.7.2]	ko00230	Purine metabolism	0.00	154.06	0.00	0.00	0.00	12.78
Lizard06Caudovirales_VIRSorter_scaffold_74707-gene_1|282|1073|791|+|-|-|-|-|AhpC-TSA|111.3|4.3e-33(+).p1	mby:MSBRM_3049	219	27	244	11	229	2.54E-100	61.644	**K03386**	Cellular Processes	Cell growth and death	PRDX2_4, ahpC	peroxiredoxin (alkyl hydroperoxide reductase subunit C) [EC:1.11.1.15]	ko04214	Apoptosis - fly	0.00	0.00	0.00	0.00	0.00	62.81
Lizard06Caudovirales_VIRSorter_scaffold_74707-gene_2|1090|1419|329|+|Phage_cluster_3529|83.5|1.3e-	hsc:HVS_11425	105	2	106	4	108	1.12E-33	56.19	**K03671**	Organismal Systems	Immune system	trxA	thioredoxin 1	ko04621,ko05132,ko05418	NOD-like receptor signaling pathway|Salmonella infection|Fluid shear stress and atherosclerosis	0.00	0.00	0.00	0.00	0.00	0.90
Lizard06Caudovirales_VIRSorter_scaffold_103742-gene_2|872|1510|638|-	pdi:BDI_3691	210	1	210	2	211	1.13E-139	91.905	**K00762**	Metabolism	Nucleotide metabolism	pyrE	orotate phosphoribosyltransferase [EC:2.4.2.10]	ko00240	Pyrimidine metabolism	0.00	27.21	0.00	4.11	0.00	8.46
Lizard06Caudovirales_VIRSorter_scaffold_52398-gene_1|85|786|701|+|-|-|-|-|SpoIID|111.9|2.5e-33(+).p1	mbf:BHV42_07105	229	1	229	48	274	8.57E-98	62.445	**K06381**	Not Included in Pathway or Brite	Unclassified: signaling and cellular processes	spoIID	stage II sporulation protein D	—	—	0.00	0.00	0.00	0.00	0.00	98.40
Lizard06Caudovirales_VIRSorter_scaffold_52398-gene_3|1294|1758|464|-|Phage_cluster_1022|117.8|3.5e-	mbf:BHV42_07475	134	21	153	15	148	1.09E-73	79.104	**K01611**	Metabolism	Amino acid metabolism	speD, AMD1	S-adenosylmethionine decarboxylase [EC:4.1.1.50]	ko00270,ko00330	Cysteine and methionine metabolism|Arginine and proline metabolism	0.00	0.00	0.00	0.00	0.00	2.66
Lizard06Caudovirales_VIRSorter_scaffold_5330-gene_1|314|904|590|+|Phage_cluster_3140|175.0|3.1e-	bfr:BF2527	194	2	195	12	205	3.58E-132	90.722	**K04564**	Environmental Information Processing	Signal transduction	SOD2	superoxide dismutase, Fe-Mn family [EC:1.15.1.1]	ko04013,ko04068,ko04146,ko04211,ko04212,ko04213,ko05016	MAPK signaling pathway - fly|FoxO signaling pathway|Peroxisome|Longevity regulating pathway|Longevity regulating pathway - worm|Longevity regulating pathway - multiple species|Huntington disease	114.99	750.86	0.00	22.80	66.41	335.30
Lizard06Caudovirales_VIRSorter_scaffold_5330-gene_3|1395|1726|331|+|-|-|-|-|TMP-TENI|98.9|3.3e-29(+).p1	boa:Bovatus_00985	110	1	110	1	110	4.31E-67	88.182	**K00788**	Metabolism	Metabolism of cofactors and vitamins	thiE	thiamine-phosphate pyrophosphorylase [EC:2.5.1.3]	ko00730	Thiamine metabolism	25.88	36.14	3.19	2.57	0.00	13.40
Lizard06Caudovirales_VIRSorter_scaffold_55602-gene_1|1|468|467|-|Phage_cluster_2180|113.9|1.2e-33|1|-|-|-(-).p1	psor:RSJ16_07690	157	1	155	2	158	1.72E-33	44.586	**K21449**	Brite Hierarchies	Protein families: signaling and cellular processes	ata, sadA, emaA	trimeric autotransporter adhesin	—	—	0.00	0.00	0.00	0.00	0.00	1475.96
Lizard06Caudovirales_VIRSorter_scaffold_86412-gene_1|2|580|578|+|Phage_cluster_205|222.2|2e-	mbf:BHV42_00455	191	1	191	763	953	1.51E-78	64.398	**K02335**	Genetic Information Processing	Replication and repair	polA	DNA polymerase I [EC:2.7.7.7]	ko03030,ko03410,ko03420,ko03440	DNA replication|Base excision repair|Nucleotide excision repair|Homologous recombination	0.00	0.00	0.00	0.00	0.00	3443.91
Lizard06Caudovirales_VIRSorter_scaffold_80340-gene_4|632|1597|965|-|Phage_cluster_415|256.6|5e-	cbo:CBO2364	279	41	318	55	330	1.97E-77	44.086	**K04763**	Brite Hierarchies	Protein families: genetic information processing	xerD	integrase/recombinase XerD	—	—	0.00	0.00	0.00	0.00	0.00	7.01
Lizard06Caudovirales_VIRSorter_scaffold_10518-gene_1|12|875|863|-|-|-|-|-|Lyase_1|124.5|9.4e-37(-).p1	pdi:BDI_3689	284	1	284	2	283	0	89.789	**K01755**	Metabolism	Amino acid metabolism	argH, ASL	argininosuccinate lyase [EC:4.3.2.1]	ko00220,ko00250	Arginine biosynthesis|Alanine, aspartate and glutamate metabolism	3008.86	0.00	370.94	0.00	0.00	573.99
Lizard06Caudovirales_VIRSorter_scaffold_10518-gene_3|1323|1682|359|-	pdi:BDI_3691	116	2	117	96	211	2.78E-71	88.793	**K00762**	Metabolism	Nucleotide metabolism	pyrE	orotate phosphoribosyltransferase [EC:2.4.2.10]	ko00240	Pyrimidine metabolism	0.00	0.00	0.00	0.00	0.00	1.49
Lizard06Caudovirales_VIRSorter_scaffold_14237-gene_3|972|1340|368|-|Phage_cluster_6491|63.9|2.3e-	mcak:MCCS_14420	85	21	101	6	89	4.18E-09	31.765	**K06940**	Not Included in Pathway or Brite	Poorly characterized	K06940	uncharacterized protein	—	—	111.44	0.00	4.58	0.00	0.00	3.04
Lizard06Caudovirales_VIRSorter_scaffold_38109-gene_2|263|640|377|+|-|-|-|-|SecE|56.6|2.6e-16(+).p1	capr:EQM14_05160	70	31	100	2	67	2.92E-13	47.143	**K03073**	Genetic Information Processing	Folding, sorting and degradation	secE	preprotein translocase subunit SecE	ko02024,ko03060,ko03070	Quorum sensing|Protein export|Bacterial secretion system	0.00	0.00	0.00	27.22	0.00	3.74
Lizard06Caudovirales_VIRSorter_scaffold_38109-gene_3|657|1199|542|+|gi_333798264_ref_YP_004508537.1_|73.6|4.55	capr:EQM14_05165	179	1	179	1	173	7.33E-83	66.48	**K02601**	Brite Hierarchies	Protein families: genetic information processing	nusG	transcription termination/antitermination protein NusG	—	—	0.00	0.00	0.00	10.90	0.00	3.74
Lizard06Caudovirales_VIRSorter_scaffold_38109-gene_4|1354|1673|319|+|-|-|-|-|Ribosomal_L11_N|106.3|7.2e-32(+).p1	esu:EUS_08900	106	1	106	1	106	2.64E-64	91.509	**K02867**	Genetic Information Processing	Translation	RP-L11, MRPL11, rplK	large subunit ribosomal protein L11	ko03010	Ribosome	0.00	0.00	0.00	0.00	0.00	0.73
Lizard06Caudovirales_VIRSorter_scaffold_81580-gene_4|1005|1406|401|+|Phage_cluster_20|153.6|8.2e-	bcac:CGC64_11030	135	1	133	1	135	8.13E-49	59.259	**K08640**	Brite Hierarchies	Protein families: metabolism	E3.4.17.14	zinc D-Ala-D-Ala carboxypeptidase [EC:3.4.17.14]	—	—	0.00	7.38	0.00	0.00	570.63	485.13
Lizard06Caudovirales_VIRSorter_scaffold_5074-gene_1|107|790|683|-|Phage_cluster_4904|143.7|9e-43|4|DUF165|144.9|3.6e-43(-).p1	pdi:BDI_1643	226	1	226	2	226	1.30E-148	92.92	**K09125**	Brite Hierarchies	Protein families: signaling and cellular processes	K09125	uncharacterized protein	—	—	0.00	12957.44	166.40	66.91	0.00	685.10
Lizard06Caudovirales_VIRSorter_scaffold_36023-gene_2|249|1574|1325|-|Phage_cluster_1607|116.6|1.8e-	elm:ELI_0943	435	12	437	267	635	1.66E-19	23.678	**K03657**	Genetic Information Processing	Replication and repair	uvrD, pcrA	DNA helicase II / ATP-dependent DNA helicase PcrA [EC:3.6.4.12]	ko03420,ko03430	Nucleotide excision repair|Mismatch repair	0.00	0.00	0.00	43.13	41.88	35.57
Lizard06Caudovirales_VIRSorter_scaffold_3287-gene_2|752|1096|344|-	tfo:BFO_2693	113	1	113	703	814	1.97E-57	85.841	**K03466**	Brite Hierarchies	Protein families: genetic information processing	ftsK, spoIIIE	DNA segregation ATPase FtsK/SpoIIIE, S-DNA-T family	—	—	336.10	678.32	76.95	76.16	69.33	212.63
Lizard06Caudovirales_VIRSorter_scaffold_3287-gene_3|1024|1623|599|-|Phage_cluster_852|193.4|8.8e-	tfo:BFO_2693	175	1	175	483	657	1.96E-105	88.571	**K03466**	Brite Hierarchies	Protein families: genetic information processing	ftsK, spoIIIE	DNA segregation ATPase FtsK/SpoIIIE, S-DNA-T family	—	—	282.08	1101.99	130.41	108.37	152.74	377.64
Lizard06Caudovirales_VIRSorter_scaffold_72109-gene_2|287|775|488|+|Phage_cluster_2087|74.1|2.1e-	amih:CO731_05106	123	5	125	1	113	2.55E-10	34.959	**K07474**	Not Included in Pathway or Brite	Unclassified: genetic information processing	xtmA	phage terminase small subunit	—	—	0.00	0.00	0.00	0.00	0.00	10.29
Lizard06Caudovirales_VIRSorter_scaffold_72109-gene_3|747|1472|725|+|Phage_cluster_338|142.8|2.2e-	eso:O3O_17975	210	25	230	15	222	2.05E-28	31.905	**K06909**	Not Included in Pathway or Brite	Unclassified: genetic information processing	xtmB	phage terminase large subunit	—	—	0.00	0.00	0.00	0.00	0.00	2.63
Lizard06Caudovirales_VIRSorter_scaffold_19790-gene_2|379|1503|1124|+|Phage_cluster_6140|294.2|2.1e-	blq:L21SP5_03347	257	16	269	180	422	1.84E-08	24.514	**K02314**	Genetic Information Processing	Replication and repair	dnaB	replicative DNA helicase [EC:3.6.4.12]	ko03030,ko04112	DNA replication|Cell cycle - Caulobacter	0.00	0.00	0.00	0.00	1045.44	249.24
Lizard06Caudovirales_VIRSorter_scaffold_107332-gene_2|372|1145|773|+|Phage_cluster_12|132.0|5.3e-	bamb:BAPNAU_2982	256	5	243	32	278	3.38E-45	39.062	**K07741**	Not Included in Pathway or Brite	Unclassified: genetic information processing	antB	anti-repressor protein	—	—	0.00	0.00	0.00	28.87	56.06	15.87
Lizard06Caudovirales_VIRSorter_scaffold_34873-gene_5|937|1578|641|-|Phage_cluster_143|94.1|1.4e-	apv:Apar_0551	209	2	210	5	208	1.40E-44	36.364	**K01356**	Brite Hierarchies	Protein families: metabolism	lexA	repressor LexA [EC:3.4.21.88]	—	—	0.00	0.00	0.00	0.00	0.00	2.63
Lizard06Caudovirales_VIRSorter_scaffold_90297-gene_3|511|1014|503|-|Phage_cluster_1305|112.2|3.8e-33|1|-|-|-(-).p1	apb:SAR116_2051	166	1	162	61	219	8.47E-17	33.735	**K03648**	Genetic Information Processing	Replication and repair	UNG, UDG	uracil-DNA glycosylase [EC:3.2.2.27]	ko03410,ko05340	Base excision repair|Primary immunodeficiency	0.00	0.00	0.00	0.00	0.00	5.78
Lizard06Caudovirales_VIRSorter_scaffold_2744-gene_1|24|647|623|-|Phage_cluster_164|126.6|1.9e-37|1|Glyco_hydro_25|79.4|5.6e-23(-).p1	gvh:HMPREF9231_0512	204	1	202	15	205	5.65E-36	39.216	**K07273**	Not Included in Pathway or Brite	Unclassified: signaling and cellular processes	acm	lysozyme	—	—	0.00	0.00	0.00	0.00	2172.27	225.49
Lizard06Caudovirales_VIRSorter_scaffold_86368-gene_1|2|736|734|+|Phage_cluster_244|165.9|2e-49|1|Toprim|73.3|2.4e-	bhl:Bache_0820	244	1	244	322	565	1.38E-166	93.852	**K02622**	Brite Hierarchies	Protein families: genetic information processing	parE	topoisomerase IV subunit B [EC:5.6.2.2]	—	—	0.00	5931.32	0.00	1789.81	10426.88	5165.87
Lizard06Caudovirales_VIRSorter_scaffold_86368-gene_3|913|1365|452|+|-|-|-|-|Pfam-B_14649|65.6|5.5e-19(+).p1	bhf:C3V43_08845	150	1	150	1	150	9.30E-97	89.333	**K00954**	Metabolism	Metabolism of cofactors and vitamins	E2.7.7.3A, coaD, kdtB	pantetheine-phosphate adenylyltransferase [EC:2.7.7.3]	ko00770	Pantothenate and CoA biosynthesis	0.00	0.00	0.00	7.84	0.00	4.31
Lizard06Caudovirales_VIRSorter_scaffold_54370-gene_1|79|789|710|-|-|-|-|-|LysR_substrate|138.5|2.9e-41(-).p1	bhl:Bache_1704	234	1	234	65	298	6.10E-141	84.615	**K21900**	Brite Hierarchies	Protein families: genetic information processing	cysL	LysR family transcriptional regulator, transcriptional activator of the cysJI operon	—	—	914.08	37.54	0.00	135.94	527.94	56.05
Lizard06Caudovirales_VIRSorter_scaffold_81112-gene_3|408|719|311|-|Phage_cluster_598|76.1|4e-22|1|-|-|-(-).p1	fpla:A4U99_14385	98	1	94	346	442	6.19E-23	58.163	**K01448**	Human Diseases	Drug resistance: antimicrobial	amiABC	N-acetylmuramoyl-L-alanine amidase [EC:3.5.1.28]	ko01503	Cationic antimicrobial peptide (CAMP) resistance	0.00	0.00	0.00	0.00	0.00	2.41
Lizard06Caudovirales_VIRSorter_scaffold_81112-gene_4|753|1247|494|-|Phage_cluster_629|148.8|2.9e-	fpla:A4U99_14385	172	1	163	163	333	5.66E-59	56.395	**K01448**	Human Diseases	Drug resistance: antimicrobial	amiABC	N-acetylmuramoyl-L-alanine amidase [EC:3.5.1.28]	ko01503	Cationic antimicrobial peptide (CAMP) resistance	0.00	0.00	0.00	0.00	13.85	1.31
Lizard06Caudovirales_VIRSorter_scaffold_70301-gene_1|46|495|449|-|Phage_cluster_88|110.2|2e-32|1|dCMP_cyt_deam_1|70.4|1.6e-20(-).p1	mbf:BHV42_03665	144	1	144	3	146	1.02E-81	79.167	**K01493**	Metabolism	Nucleotide metabolism	comEB	dCMP deaminase [EC:3.5.4.12]	ko00240	Pyrimidine metabolism	0.00	0.00	0.00	0.00	0.00	139.04
Lizard06Caudovirales_VIRSorter_scaffold_26375-gene_2|318|1133|815|-	mbf:BHV42_03110	262	2	263	97	355	9.53E-109	58.397	**K04096**	Not Included in Pathway or Brite	Unclassified: genetic information processing	smf	DNA processing protein	—	—	0.00	0.00	0.00	0.00	0.00	75.81
Lizard06Caudovirales_VIRSorter_scaffold_55808-gene_1|2|388|386|+|Phage_cluster_1122|96.8|3e-	cbo:CBO2325	76	3	78	104	179	1.45E-19	53.947	**K01448**	Human Diseases	Drug resistance: antimicrobial	amiABC	N-acetylmuramoyl-L-alanine amidase [EC:3.5.1.28]	ko01503	Cationic antimicrobial peptide (CAMP) resistance	0.00	0.00	0.00	0.00	0.00	245.99
Lizard06Caudovirales_VIRSorter_scaffold_79306-gene_3|564|1127|563|+|Phage_cluster_289|175.7|2.7e-	metm:MSMTP_1453	186	2	187	65	250	1.24E-54	51.075	**K07496**	Not Included in Pathway or Brite	Unclassified: genetic information processing	K07496	putative transposase	—	—	0.00	0.00	0.00	0.00	92.44	362.88
Lizard06Caudovirales_VIRSorter_scaffold_46133-gene_2|197|769|572|-|Phage_cluster_2578|66.6|3.6e-	bfr:BF0614	189	1	189	2	190	1.89E-118	88.889	**K03811**	Brite Hierarchies	Protein families: signaling and cellular processes	pnuC	nicotinamide mononucleotide transporter	—	—	0.00	188.18	0.00	18.17	0.00	17.48
Lizard06Caudovirales_VIRSorter_scaffold_50787-gene_2|666|980|314|-|-|-|-|-|Na_H_antiport_1|91.1|1.1e-26(-).p2	bfs:BF9343_2498	103	1	103	335	437	9.58E-51	79.612	**K03313**	Brite Hierarchies	Protein families: signaling and cellular processes	nhaA	Na+:H+ antiporter, NhaA family	—	—	0.00	0.00	0.00	0.00	0.00	0.00
Lizard06Caudovirales_VIRSorter_scaffold_47417-gene_3|601|1015|414|+|Phage_cluster_57|70.9|1.4e-	byl:A4V09_00730	111	26	136	166	271	4.76E-08	33.333	**K06909**	Not Included in Pathway or Brite	Unclassified: genetic information processing	xtmB	phage terminase large subunit	—	—	0.00	0.00	0.00	0.00	0.00	1.64
Lizard06Caudovirales_VIRSorter_scaffold_4598-gene_3|712|1013|301|+|Phage_cluster_78|71.1|1.4e-20|1|-|-|-(+).p1	stan:STA3757_24330	99	3	100	1	88	3.89E-18	46.465	**K01520**	Metabolism	Nucleotide metabolism	dut, DUT	dUTP pyrophosphatase [EC:3.6.1.23]	ko00240,ko00983	Pyrimidine metabolism|Drug metabolism - other enzymes	0.00	0.00	6.25	0.00	14.64	17.97
Lizard06Caudovirales_VIRSorter_scaffold_37557-gene_1|23|457|434|-|Phage_cluster_6303|94.4|1.1e-27|1|ParBc|77.2|1.5e-22(-).p2	parc:CI960_18985	142	1	142	20	161	2.07E-56	80.986	**K03497**	Brite Hierarchies	Protein families: genetic information processing	parB, spo0J	ParB family transcriptional regulator, chromosome partitioning protein	—	—	0.00	193.41	0.00	0.00	0.00	64.17
Lizard06Caudovirales_VIRSorter_scaffold_37557-gene_2|608|910|302|-|Phage_cluster_438|67.8|2.2e-19|1|-|-|-(-).p1	pdi:BDI_2559	99	1	99	156	254	1.43E-61	92.929	**K03496**	Brite Hierarchies	Protein families: genetic information processing	parA, soj	chromosome partitioning protein	—	—	0.00	0.00	0.00	0.00	0.00	0.81

<table>
<tr><th rowspan="2">Query ORF ID</th><th rowspan="2">Subject gene ID</th><th rowspan="2">Alignment length</th><th rowspan="2">Query start</th><th rowspan="2">Query end</th><th rowspan="2">Subject start</th><th rowspan="2">Subject end</th><th rowspan="2">e-value</th><th rowspan="2">Identity(%)</th><th rowspan="2">KO</th><th rowspan="2">Category</th><th rowspan="2">Subcategory</th><th rowspan="2">Gene name</th><th rowspan="2">Description</th><th rowspan="2">Pathway</th><th rowspan="2">Pathway definition</th><th colspan="6">Abundance (fpkm)</th></tr>
<tr><th>(1) PE-NMC</th><th>(2) PE-NQ1</th><th>(3) PE-NQ2</th><th>(4) PT-SZZ</th><th>(5) PT-ZB</th><th>(6) PT-RT</th></tr>
<tr><td>Lizard06Caudovirales_VIRSorter_scaffold_65678-gene_2|298|840|542|+|Phage_cluster_2492|159.1|1.6e-</td><td>bacy:QF06_20630</td><td>155</td><td>1</td><td>150</td><td>1</td><td>154</td><td>1.46E-17</td><td>32.903</td><td>K00942</td><td>Metabolism</td><td>Nucleotide metabolism</td><td>E2.7.4.8, gmk</td><td>guanylate kinase [EC:2.7.4.8]</td><td>ko00230</td><td>Purine metabolism</td><td>0.00</td><td>0.00</td><td>0.00</td><td>0.00</td><td>0.00</td><td>4.95</td></tr>
<tr><td>Lizard06Caudovirales_VIRSorter_scaffold_96719-gene_3|562|897|335|-|Phage_cluster_9|101.4|7.5e-30|1|SSB|56.7|3.3e-</td><td>cbol:CGC65_24530</td><td>100</td><td>11</td><td>110</td><td>50</td><td>147</td><td>2.09E-40</td><td>64</td><td>K03111</td><td>Genetic Information Processing</td><td>Replication and repair</td><td>ssb</td><td>single-strand DNA-binding protein</td><td>ko03030,ko03430,ko03440</td><td>DNA replication|Mismatch repair|Homologous recombination</td><td>0.00</td><td>0.00</td><td>0.00</td><td>0.00</td><td>0.00</td><td>0.88</td></tr>
<tr><td>Lizard06unclassified_bacterial_viruses_VIRSorter_scaffold_14976-gene_5|1316|2161|845|-|Phage_cluster_551|153.3|2e-</td><td>ckr:CKR_1755</td><td>246</td><td>29</td><td>265</td><td>10</td><td>250</td><td>4.33E-37</td><td>34.146</td><td>K07455</td><td>Brite Hierarchies</td><td>Protein families: genetic information processing</td><td>recT</td><td>recombination protein RecT</td><td>—</td><td>—</td><td>0.00</td><td>0.00</td><td>0.00</td><td>0.00</td><td>0.00</td><td>20.56</td></tr>
<tr><td>Lizard06unclassified_bacterial_viruses_VIRSorter_scaffold_14976-gene_10|3981|5063|1082|-|Phage_cluster_1277|315.9|2e-</td><td>rhl:LPU83_1989</td><td>303</td><td>56</td><td>357</td><td>38</td><td>318</td><td>2.53E-62</td><td>47.525</td><td>K15352</td><td>Human Diseases</td><td>Infectious disease: bacterial</td><td>pipB2</td><td>secreted effector protein PipB2</td><td>ko05132</td><td>Salmonella infection</td><td>0.00</td><td>0.00</td><td>0.00</td><td>0.00</td><td>0.00</td><td>12.73</td></tr>
<tr><td>Lizard06unclassified_bacterial_viruses_VIRSorter_scaffold_14976-gene_40|28136|28567|431|-|-|-|-|-|-|-|-|-(-).p1</td><td>ctak:4412677_01892</td><td>95</td><td>9</td><td>103</td><td>22</td><td>116</td><td>4.13E-12</td><td>32.632</td><td>K04047</td><td>Brite Hierarchies</td><td>Protein families: genetic information processing</td><td>dps</td><td>starvation-inducible DNA-binding protein</td><td>—</td><td>—</td><td>0.00</td><td>0.00</td><td>0.00</td><td>0.00</td><td>0.00</td><td>0.35</td></tr>
<tr><td>Lizard06unclassified_bacterial_viruses_VIRSorter_scaffold_19149-gene_5|1554|2174|620|+|Phage_cluster_6058|147.0|1.7e-</td><td>ere:EUBREC_2136</td><td>126</td><td>81</td><td>206</td><td>69</td><td>188</td><td>2.45E-28</td><td>47.619</td><td>K01449</td><td>Not Included in Pathway or Brite</td><td>Unclassified: metabolism</td><td>cwlJ, sleB</td><td>N-acetylmuramoyl-L-alanine amidase [EC:3.5.1.28]</td><td>—</td><td>—</td><td>0.00</td><td>0.00</td><td>0.00</td><td>0.00</td><td>0.00</td><td>3.48</td></tr>
<tr><td>Lizard06unclassified_bacterial_viruses_VIRSorter_scaffold_19149-gene_10|4196|5503|1307|+|Phage_cluster_57|76.8|3.7e-</td><td>bamf:U722_06610</td><td>439</td><td>3</td><td>427</td><td>2</td><td>413</td><td>7.85E-35</td><td>25.968</td><td>K06909</td><td>Not Included in Pathway or Brite</td><td>Unclassified: genetic information processing</td><td>xtmB</td><td>phage terminase large subunit</td><td>—</td><td>—</td><td>0.00</td><td>0.00</td><td>0.00</td><td>0.00</td><td>9.94</td><td>9.97</td></tr>
<tr><td>Lizard06unclassified_bacterial_viruses_VIRSorter_scaffold_19149-gene_11|5527|6915|1388|+|Phage_cluster_57|361.4|2e-</td><td>ctyk:CTK_C10820</td><td>455</td><td>1</td><td>455</td><td>1</td><td>421</td><td>6.89E-173</td><td>56.264</td><td>K06909</td><td>Not Included in Pathway or Brite</td><td>Unclassified: genetic information processing</td><td>xtmB</td><td>phage terminase large subunit</td><td>—</td><td>—</td><td>0.00</td><td>0.00</td><td>0.00</td><td>0.00</td><td>3.77</td><td>21.36</td></tr>
<tr><td>Lizard06unclassified_bacterial_viruses_VIRSorter_scaffold_19149-gene_14|7587|9002|1415|+|Phage_cluster_371|487.1|1.8e-147|2|Cpl-</td><td>det:DET1070</td><td>223</td><td>162</td><td>383</td><td>223</td><td>445</td><td>2.27E-89</td><td>60.09</td><td>K07273</td><td>Not Included in Pathway or Brite</td><td>Unclassified: signaling and cellular processes</td><td>acm</td><td>lysozyme</td><td>—</td><td>—</td><td>0.00</td><td>0.00</td><td>0.00</td><td>0.00</td><td>6.87</td><td>14.40</td></tr>
<tr><td>Lizard06unclassified_bacterial_viruses_VIRSorter_scaffold_4255-gene_3|2091|3314|1223|+|Phage_cluster_457|415.5|7.2e-</td><td>tto:Thethe_02918</td><td>398</td><td>3</td><td>399</td><td>10</td><td>396</td><td>2.25E-116</td><td>43.216</td><td>K14415</td><td>Brite Hierarchies</td><td>Protein families: genetic information processing</td><td>RTCB, rtcB</td><td>tRNA-splicing ligase RtcB (3'-phosphate/5'-hydroxy nucleic acid ligase) [EC:6.5.1.8]</td><td>—</td><td>—</td><td>0.00</td><td>0.00</td><td>0.00</td><td>0.00</td><td>0.00</td><td>12.00</td></tr>
<tr><td>Lizard06unclassified_bacterial_viruses_VIRSorter_scaffold_4255-gene_21|11924|14917|2993|+|Phage_cluster_10114|302.3|1.6e-91|0|-|-|-</td><td>mem:Memar_1247</td><td>380</td><td>580</td><td>913</td><td>415</td><td>772</td><td>6.80E-25</td><td>28.158</td><td>K10726</td><td>Genetic Information Processing</td><td>Replication and repair</td><td>mcm, cdc21</td><td>replicative DNA helicase Mcm [EC:3.6.4.-]</td><td>ko03030</td><td>DNA replication</td><td>0.00</td><td>0.00</td><td>0.00</td><td>0.00</td><td>0.00</td><td>4.33</td></tr>
<tr><td>Lizard06unclassified_bacterial_viruses_VIRSorter_scaffold_4255-gene_23|15418|15765|347|-|-|-|-|-|DUF192|40.2|6.6e-12(-).p1</td><td>vbl:L21SP4_01272</td><td>96</td><td>10</td><td>100</td><td>43</td><td>137</td><td>4.79E-07</td><td>35.417</td><td>K09005</td><td>Not Included in Pathway or Brite</td><td>Poorly characterized</td><td>K09005</td><td>uncharacterized protein</td><td>—</td><td>—</td><td>0.00</td><td>0.00</td><td>0.00</td><td>0.00</td><td>0.00</td><td>0.16</td></tr>
<tr><td>Lizard06unclassified_bacterial_viruses_VIRSorter_scaffold_78317-gene_4|1694|2761|1067|-|-|-|-|-|AIRS|73.5|3.7e-22(-).p1</td><td>osp:Odosp_3268</td><td>353</td><td>1</td><td>351</td><td>2</td><td>354</td><td>0</td><td>87.252</td><td>K00946</td><td>Metabolism</td><td>Metabolism of cofactors and vitamins</td><td>thiL</td><td>thiamine-monophosphate kinase [EC:2.7.4.16]</td><td>ko00730</td><td>Thiamine metabolism</td><td>0.00</td><td>0.00</td><td>0.00</td><td>8.45</td><td>98.48</td><td>114.72</td></tr>
<tr><td>Lizard06unclassified_bacterial_viruses_VIRSorter_scaffold_78317-gene_5|2977|4419|1442|+|-|-|-|-|UPF0051|217.0|6.9e-66(+).p1</td><td>osp:Odosp_3269</td><td>477</td><td>4</td><td>480</td><td>1</td><td>477</td><td>0</td><td>94.13</td><td>K09014</td><td>Not Included in Pathway or Brite</td><td>Poorly characterized</td><td>sufB</td><td>Fe-S cluster assembly protein SufB</td><td>—</td><td>—</td><td>0.00</td><td>0.00</td><td>0.00</td><td>32.47</td><td>31.52</td><td>141.13</td></tr>
<tr><td>Lizard06unclassified_bacterial_viruses_VIRSorter_scaffold_78317-gene_6|4427|5212|785|+|Phage_cluster_405|94.6|1.5e-</td><td>osp:Odosp_3270</td><td>249</td><td>9</td><td>257</td><td>1</td><td>249</td><td>4.74E-174</td><td>94.378</td><td>K09013</td><td>Brite Hierarchies</td><td>Protein families: signaling and cellular processes</td><td>sufC</td><td>Fe-S cluster assembly ATP-binding protein</td><td>—</td><td>—</td><td>0.00</td><td>0.00</td><td>0.00</td><td>2.43</td><td>42.40</td><td>63.57</td></tr>
<tr><td>Lizard06unclassified_bacterial_viruses_VIRSorter_scaffold_78317-gene_7|5226|6590|1364|+|-|-|-|-|UPF0051|255.1|1.6e-77(+).p1</td><td>osp:Odosp_3271</td><td>452</td><td>1</td><td>452</td><td>1</td><td>452</td><td>0</td><td>84.956</td><td>K09015</td><td>Not Included in Pathway or Brite</td><td>Poorly characterized</td><td>sufD</td><td>Fe-S cluster assembly protein SufD</td><td>—</td><td>—</td><td>0.00</td><td>0.00</td><td>0.00</td><td>8.90</td><td>67.84</td><td>127.00</td></tr>
<tr><td>Lizard06unclassified_bacterial_viruses_VIRSorter_scaffold_78317-gene_8|6604|7824|1220|+|Phage_cluster_1231|169.8|2.1e-</td><td>osp:Odosp_3272</td><td>404</td><td>1</td><td>404</td><td>1</td><td>404</td><td>0</td><td>82.426</td><td>K11717</td><td>Metabolism</td><td>Metabolism of other amino acids</td><td>sufS</td><td>cysteine desulfurase / selenocysteine lyase [EC:2.8.1.7 4.4.1.16]</td><td>ko00450</td><td>Selenocompound metabolism</td><td>0.00</td><td>0.00</td><td>0.00</td><td>11.38</td><td>61.58</td><td>133.28</td></tr>
<tr><td>Lizard06unclassified_bacterial_viruses_VIRSorter_scaffold_78317-gene_9|7834|8292|458|+|Phage_cluster_5579|81.5|1.3e-</td><td>osp:Odosp_3273</td><td>141</td><td>12</td><td>152</td><td>1</td><td>141</td><td>2.77E-84</td><td>82.27</td><td>K02426</td><td>Not Included in Pathway or Brite</td><td>Poorly characterized</td><td>sufE</td><td>cysteine desulfuration protein SufE</td><td>—</td><td>—</td><td>0.00</td><td>0.00</td><td>0.00</td><td>9.14</td><td>17.30</td><td>34.04</td></tr>
<tr><td>Lizard06unclassified_bacterial_viruses_VIRSorter_scaffold_78317-gene_11|8617|9519|902|+|-|-|-|-|DUF1730|60.9|2.1e-18(+).p1</td><td>buy:D8S85_08410</td><td>298</td><td>4</td><td>299</td><td>2</td><td>298</td><td>1.06E-116</td><td>56.04</td><td>K18979</td><td>Brite Hierarchies</td><td>Protein families: genetic information processing</td><td>queG</td><td>epoxyqueuosine reductase [EC:1.17.99.6]</td><td>—</td><td>—</td><td>0.00</td><td>0.00</td><td>0.00</td><td>10.39</td><td>77.44</td><td>76.16</td></tr>
<tr><td>Lizard06unclassified_bacterial_viruses_VIRSorter_scaffold_78317-gene_12|9603|10933|1330|+|-|-|-|-|BATS|65.0|1.2e-19(+).p1</td><td>osp:Odosp_3276</td><td>443</td><td>1</td><td>443</td><td>1</td><td>443</td><td>0</td><td>94.131</td><td>K03150</td><td>Metabolism</td><td>Metabolism of cofactors and vitamins</td><td>thiH</td><td>2-iminoacetate synthase [EC:4.1.99.19]</td><td>ko00730</td><td>Thiamine metabolism</td><td>0.00</td><td>0.00</td><td>0.00</td><td>31.31</td><td>141.15</td><td>156.98</td></tr>
<tr><td>Lizard06unclassified_bacterial_viruses_VIRSorter_scaffold_8636-gene_15|9691|10029|338|+|Phage_cluster_2089|131.2|1.2e-</td><td>pary:A4V02_06420</td><td>105</td><td>1</td><td>105</td><td>270</td><td>374</td><td>1.67E-28</td><td>52.381</td><td>K04763</td><td>Brite Hierarchies</td><td>Protein families: genetic information processing</td><td>xerD</td><td>integrase/recombinase XerD</td><td>—</td><td>—</td><td>0.00</td><td>0.00</td><td>0.00</td><td>0.00</td><td>0.00</td><td>1.02</td></tr>
<tr><td>Lizard06unclassified_bacterial_viruses_VIRSorter_scaffold_8636-gene_16|10109|10615|506|-|Phage_cluster_6439|47.7|3.4e-</td><td>cfm:BJL90_16990</td><td>75</td><td>8</td><td>82</td><td>21</td><td>95</td><td>1.36E-07</td><td>37.333</td><td>K15773</td><td>Brite Hierarchies</td><td>Protein families: genetic information processing</td><td>hipB</td><td>HTH-type transcriptional regulator / antitoxin HipB</td><td>—</td><td>—</td><td>0.00</td><td>0.00</td><td>0.00</td><td>0.00</td><td>0.00</td><td>0.92</td></tr>
<tr><td>Lizard06unclassified_bacterial_viruses_VIRSorter_scaffold_2340-gene_12|4174|5043|869|-|Phage_cluster_2094|218.9|2e-</td><td>lagl:BEN83_03325</td><td>216</td><td>3</td><td>217</td><td>7</td><td>221</td><td>1.06E-49</td><td>41.667</td><td>K07455</td><td>Brite Hierarchies</td><td>Protein families: genetic information processing</td><td>recT</td><td>recombination protein RecT</td><td>—</td><td>—</td><td>0.00</td><td>14.21</td><td>4.27</td><td>20.58</td><td>244.81</td><td>303.99</td></tr>
<tr><td>Lizard06unclassified_bacterial_viruses_VIRSorter_scaffold_2340-gene_17|6740|8491|1751|-|Phage_cluster_503|237.4|7e-72|1|Pfam-</td><td>mpz:Marpi_0300</td><td>604</td><td>10</td><td>578</td><td>79</td><td>669</td><td>9.55E-32</td><td>26.325</td><td>K03546</td><td>Brite Hierarchies</td><td>Protein families: genetic information processing</td><td>sbcC, rad50</td><td>DNA repair protein SbcC/Rad50</td><td>—</td><td>—</td><td>0.00</td><td>8.36</td><td>1.32</td><td>18.06</td><td>527.53</td><td>609.51</td></tr>
<tr><td>Lizard06unclassified_bacterial_viruses_VIRSorter_scaffold_16340-gene_1|1|615|614|+|gi_333798264_ref_YP_004508537.1_|74.7|3.69e-</td><td>mbf:BHV42_07770</td><td>184</td><td>21</td><td>204</td><td>18</td><td>201</td><td>2.13E-107</td><td>80.435</td><td>K02601</td><td>Brite Hierarchies</td><td>Protein families: genetic information processing</td><td>nusG</td><td>transcription termination/antitermination protein NusG</td><td>—</td><td>—</td><td>0.00</td><td>0.00</td><td>0.00</td><td>0.00</td><td>0.00</td><td>4919.87</td></tr>
<tr><td>Lizard06unclassified_bacterial_viruses_VIRSorter_scaffold_16340-gene_2|788|1213|425|+|-|-|-|-|Ribosomal_L11_N|106.5|1e-32(+).p1</td><td>mbf:BHV42_07775</td><td>141</td><td>1</td><td>141</td><td>1</td><td>141</td><td>2.68E-87</td><td>88.652</td><td>K02867</td><td>Genetic Information Processing</td><td>Translation</td><td>RP-L11, MRPL11, rplK</td><td>large subunit ribosomal protein L11</td><td>ko03010</td><td>Ribosome</td><td>0.00</td><td>0.00</td><td>0.00</td><td>0.00</td><td>0.00</td><td>4.37</td></tr>
<tr><td>Lizard06unclassified_bacterial_viruses_VIRSorter_scaffold_16340-gene_3|1369|2091|722|+|-|-|-|-|Ribosomal_L1|161.7|4.8e-49(+).p1</td><td>mbf:BHV42_07780</td><td>238</td><td>1</td><td>238</td><td>1</td><td>238</td><td>4.05E-137</td><td>78.992</td><td>K02863</td><td>Genetic Information Processing</td><td>Translation</td><td>RP-L1, MRPL1, rplA</td><td>large subunit ribosomal protein L1</td><td>ko03010</td><td>Ribosome</td><td>0.00</td><td>0.00</td><td>0.00</td><td>0.00</td><td>0.00</td><td>6.83</td></tr>
<tr><td>Lizard06unclassified_bacterial_viruses_VIRSorter_scaffold_16340-gene_5|2827|5268|2441|-|-|-|-|-|H_PPase|577.1|1.4e-174(-).p1</td><td>mbf:BHV42_09050</td><td>792</td><td>18</td><td>807</td><td>16</td><td>807</td><td>0</td><td>81.439</td><td>K15987</td><td>Brite Hierarchies</td><td>Protein families: signaling and cellular processes</td><td>hppA</td><td>K(+)-stimulated pyrophosphate-energized sodium pump</td><td>—</td><td>—</td><td>0.00</td><td>0.00</td><td>0.00</td><td>0.00</td><td>0.00</td><td>33.94</td></tr>
<tr><td>Lizard06unclassified_bacterial_viruses_VIRSorter_scaffold_16340-gene_7|6635|7984|1349|-|Phage_cluster_71|548.6|2.7e-</td><td>mbf:BHV42_06090</td><td>445</td><td>13</td><td>448</td><td>4</td><td>447</td><td>0</td><td>68.315</td><td>K02314</td><td>Genetic Information Processing</td><td>Replication and repair</td><td>dnaB</td><td>replicative DNA helicase [EC:3.6.4.12]</td><td>ko03030,ko04112</td><td>DNA replication|Cell cycle - Caulobacter</td><td>0.00</td><td>0.00</td><td>0.00</td><td>0.00</td><td>0.00</td><td>3.41</td></tr>
<tr><td>Lizard06unclassified_bacterial_viruses_VIRSorter_scaffold_4593-gene_3|1859|2608|749|+|-|-|-|-|ATP_bind_3|59.3|1e-17(+).p1</td><td>bfs:BF9343_1088</td><td>249</td><td>1</td><td>249</td><td>1</td><td>249</td><td>6.34E-168</td><td>87.149</td><td>K14058</td><td>Brite Hierarchies</td><td>Protein families: genetic information processing</td><td>ttcA</td><td>tRNA 2-thiocytidine biosynthesis protein TtcA</td><td>—</td><td>—</td><td>0.00</td><td>143.58</td><td>0.00</td><td>9.63</td><td>39.26</td><td>53.72</td></tr>
<tr><td>Lizard06unclassified_bacterial_viruses_VIRSorter_scaffold_4593-gene_4|2686|3006|320|+|-|-|-|-|DUF486|102.6|3.4e-31(+).p1</td><td>bfs:BF9343_1089</td><td>106</td><td>1</td><td>106</td><td>17</td><td>122</td><td>2.56E-68</td><td>89.623</td><td>K09922</td><td>Not Included in Pathway or Brite</td><td>Poorly characterized</td><td>K09922</td><td>uncharacterized protein</td><td>—</td><td>—</td><td>0.00</td><td>17.67</td><td>0.00</td><td>0.00</td><td>0.00</td><td>4.58</td></tr>
<tr><td>Lizard06unclassified_bacterial_viruses_VIRSorter_scaffold_4593-gene_5|3179|4195|1016|-|-|-|-|-|ApbE|268.7|1.3e-81(-).p1</td><td>bfr:BF1182</td><td>335</td><td>1</td><td>335</td><td>2</td><td>336</td><td>0</td><td>85.97</td><td>K03734</td><td>Not Included in Pathway or Brite</td><td>Unclassified: metabolism</td><td>apbE</td><td>FAD:protein FMN transferase [EC:2.7.1.180]</td><td>—</td><td>—</td><td>0.00</td><td>143.67</td><td>2.80</td><td>1.13</td><td>6.56</td><td>44.42</td></tr>
<tr><td>Lizard06unclassified_bacterial_viruses_VIRSorter_scaffold_4593-gene_6|4202|4783|581|-|-|-|-|-|DUF204|95.0|6.7e-29(-).p1</td><td>bfs:BF9343_1091</td><td>192</td><td>1</td><td>192</td><td>2</td><td>193</td><td>1.44E-101</td><td>77.604</td><td>K23242</td><td>Brite Hierarchies</td><td>Protein families: signaling and cellular processes</td><td>mntP</td><td>manganese efflux pump family protein</td><td>—</td><td>—</td><td>0.00</td><td>42.35</td><td>0.00</td><td>2.56</td><td>0.00</td><td>7.96</td></tr>
<tr><td>Lizard06unclassified_bacterial_viruses_VIRSorter_scaffold_16595-gene_3|3025|5313|2288|-|Phage_cluster_2193|95.0|9.9e-</td><td>pdi:BDI_2550</td><td>761</td><td>1</td><td>761</td><td>14</td><td>773</td><td>0</td><td>88.305</td><td>K01139</td><td>Metabolism</td><td>Nucleotide metabolism</td><td>spoT</td><td>GTP diphosphokinase / guanosine-3',5'-bis(diphosphate) 3'-diphosphatase [EC:2.7.6.5 3.1.7.2]</td><td>ko00230</td><td>Purine metabolism</td><td>0.00</td><td>3.92</td><td>0.00</td><td>0.00</td><td>6.89</td><td>32.53</td></tr>
<tr><td>Lizard06unclassified_bacterial_viruses_VIRSorter_scaffold_16595-gene_4|5407|6933|1526|-|-|-|-|-|LysM|88.4|6.1e-27(-).p1</td><td>parc:CI960_18975</td><td>521</td><td>1</td><td>507</td><td>3</td><td>518</td><td>0</td><td>71.401</td><td>K08307</td><td>Brite Hierarchies</td><td>Protein families: metabolism</td><td>mltD, dniR</td><td>membrane-bound lytic murein transglycosylase D [EC:4.2.2.-]</td><td>—</td><td>—</td><td>0.00</td><td>0.82</td><td>0.82</td><td>0.00</td><td>0.00</td><td>4.64</td></tr>
<tr><td>Lizard06unclassified_bacterial_viruses_VIRSorter_scaffold_16595-gene_6|7683|7997|314|-|-|-|-|-|-|-|-|-(-).p2</td><td>parc:CI960_18985</td><td>105</td><td>1</td><td>103</td><td>192</td><td>295</td><td>8.36E-47</td><td>78.095</td><td>K03497</td><td>Brite Hierarchies</td><td>Protein families: genetic information processing</td><td>parB, spo0J</td><td>ParB family transcriptional regulator, chromosome partitioning protein</td><td>—</td><td>—</td><td>0.00</td><td>0.19</td><td>0.00</td><td>0.00</td><td>0.00</td><td>0.38</td></tr>
<tr><td>Lizard06unclassified_bacterial_viruses_VIRSorter_scaffold_7453-gene_4|4415|6397|1982|-|Phage_cluster_197|710.4|4.9e-215|1|Pfam-</td><td>ova:OBV_43170</td><td>662</td><td>2</td><td>659</td><td>17</td><td>672</td><td>0</td><td>63.293</td><td>K02334</td><td>Not Included in Pathway or Brite</td><td>Unclassified: metabolism</td><td>dpo</td><td>DNA polymerase bacteriophage-type [EC:2.7.7.7]</td><td>—</td><td>—</td><td>0.00</td><td>0.67</td><td>2.02</td><td>22.70</td><td>18.89</td><td>11.48</td></tr>
<tr><td>Lizard06unclassified_bacterial_viruses_VIRSorter_scaffold_64769-gene_5|2174|3388|1214|+|Phage_cluster_1231|138.3|7.7e-</td><td>parc:CI960_00830</td><td>404</td><td>1</td><td>404</td><td>1</td><td>404</td><td>0</td><td>89.851</td><td>K11717</td><td>Metabolism</td><td>Metabolism of other amino acids</td><td>sufS</td><td>cysteine desulfurase / selenocysteine lyase [EC:2.8.1.7 4.4.1.16]</td><td>ko00450</td><td>Selenocompound metabolism</td><td>0.00</td><td>0.00</td><td>0.00</td><td>19.76</td><td>71.94</td><td>62.69</td></tr>
<tr><td>Lizard06unclassified_bacterial_viruses_VIRSorter_scaffold_64769-gene_9|4908|5507|599|+|-|-|-|-|RNase_HII|160.1|1.5e-48(+).p1</td><td>pdi:BDI_3545</td><td>199</td><td>1</td><td>199</td><td>1</td><td>199</td><td>1.12E-127</td><td>86.935</td><td>K03470</td><td>Genetic Information Processing</td><td>Replication and repair</td><td>rnhB</td><td>ribonuclease HII [EC:3.1.26.4]</td><td>ko03030</td><td>DNA replication</td><td>0.00</td><td>0.30</td><td>3.63</td><td>0.73</td><td>0.00</td><td>4.81</td></tr>
<tr><td>Lizard06unclassified_bacterial_viruses_VIRSorter_scaffold_36814-gene_1|269|1012|743|+|-|-|-|-|Peptidase_M17_N|75.3|1e-22(+).p1</td><td>mox:DAMO_0754</td><td>249</td><td>4</td><td>247</td><td>2</td><td>246</td><td>2.08E-46</td><td>40.964</td><td>K01255</td><td>Metabolism</td><td>Metabolism of other amino acids</td><td>CARP, pepA</td><td>leucyl aminopeptidase [EC:3.4.11.1]</td><td>ko00480</td><td>Glutathione metabolism</td><td>0.00</td><td>0.00</td><td>0.00</td><td>0.00</td><td>0.00</td><td>29.26</td></tr>
<tr><td>Lizard06unclassified_bacterial_viruses_VIRSorter_scaffold_36814-gene_2|1076|1717|641|+|-|-|-|-|Peptidase_M17|287.4|3.1e-87(+).p1</td><td>mbf:BHV42_03335</td><td>211</td><td>1</td><td>211</td><td>253</td><td>463</td><td>2.62E-106</td><td>72.512</td><td>K01255</td><td>Metabolism</td><td>Metabolism of other amino acids</td><td>CARP, pepA</td><td>leucyl aminopeptidase [EC:3.4.11.1]</td><td>ko00480</td><td>Glutathione metabolism</td><td>0.00</td><td>0.00</td><td>0.00</td><td>0.00</td><td>0.00</td><td>18.75</td></tr>
<tr><td>Lizard06unclassified_bacterial_viruses_VIRSorter_scaffold_36814-gene_5|3752|5269|1517|-|-|-|-|-|Malic_M|218.3|3.9e-66(-).p1</td><td>tjr:TherJR_1506</td><td>304</td><td>183</td><td>486</td><td>65</td><td>368</td><td>2.44E-114</td><td>54.605</td><td>K00027</td><td>Metabolism</td><td>Carbohydrate metabolism</td><td>ME2, sfcA, maeA</td><td>malate dehydrogenase (oxaloacetate-decarboxylating) [EC:1.1.1.38]</td><td>ko00620,ko02020</td><td>Pyruvate metabolism|Two-component system</td><td>0.00</td><td>0.00</td><td>0.00</td><td>0.00</td><td>0.00</td><td>10.75</td></tr>
<tr><td>Lizard06unclassified_bacterial_viruses_VIRSorter_scaffold_77593-gene_2|770|2185|1415|-|Phage_cluster_1765|159.9|1.9e-</td><td>mbf:BHV42_01075</td><td>464</td><td>1</td><td>463</td><td>5</td><td>462</td><td>2.16E-142</td><td>46.552</td><td>K00970</td><td>Genetic Information Processing</td><td>Folding, sorting and degradation</td><td>pcnB</td><td>poly(A) polymerase [EC:2.7.7.19]</td><td>ko03018</td><td>RNA degradation</td><td>0.00</td><td>0.00</td><td>0.00</td><td>0.00</td><td>0.00</td><td>17.25</td></tr>
<tr><td>Lizard06unclassified_bacterial_viruses_VIRSorter_scaffold_77593-gene_3|2178|2918|740|-|-|-|-|-|Methyltrans_RNA|200.7|5.1e-61(-).p1</td><td>mbf:BHV42_01080</td><td>245</td><td>1</td><td>244</td><td>2</td><td>245</td><td>4.24E-69</td><td>46.939</td><td>K09761</td><td>Brite Hierarchies</td><td>Protein families: genetic information processing</td><td>rsmE</td><td>16S rRNA (uracil1498-N3)-methyltransferase [EC:2.1.1.193]</td><td>—</td><td>—</td><td>0.00</td><td>0.00</td><td>0.00</td><td>0.00</td><td>0.00</td><td>2.94</td></tr>
<tr><td>Lizard06unclassified_bacterial_viruses_VIRSorter_scaffold_77593-gene_4|3122|4084|962|+|-|-|-|-|Flagellin_N|99.3|4.6e-30(+).p1</td><td>sti:Sthe_0558</td><td>318</td><td>3</td><td>318</td><td>1</td><td>291</td><td>5.63E-59</td><td>39.937</td><td>K02406</td><td>Environmental Information Processing</td><td>Signal transduction</td><td>fliC</td><td>flagellin</td><td>ko02020,ko02040,ko04621,ko04626,ko05131,ko05132,ko05134</td><td>Two-component system|Flagellar assembly|NOD-like receptor signaling pathway|Plant-pathogen interaction|Shigellosis|Salmonella infection|Legionellosis</td><td>0.00</td><td>0.00</td><td>0.00</td><td>3.44</td><td>13.36</td><td>14.66</td></tr>
<tr><td>Lizard06unclassified_bacterial_viruses_VIRSorter_scaffold_77593-gene_5|4207|4938|731|-|-|-|-|-|GGDEF|161.2|4.6e-49(-).p1</td><td>mbf:BHV42_02220</td><td>237</td><td>3</td><td>238</td><td>207</td><td>442</td><td>8.22E-71</td><td>49.789</td><td>K02488</td><td>Environmental Information Processing</td><td>Signal transduction</td><td>pleD</td><td>two-component system, cell cycle response regulator [EC:2.7.7.65]</td><td>ko02020,ko04112</td><td>Two-component system|Cell cycle - Caulobacter</td><td>0.00</td><td>0.00</td><td>0.00</td><td>0.00</td><td>0.00</td><td>2.22</td></tr>
<tr><td>Lizard06unclassified_bacterial_viruses_VIRSorter_scaffold_46117-gene_10|4050|4592|542|+|Phage_cluster_197|201.9|3.9e-61|1|Pfam-</td><td>cpas:Clopa_0561</td><td>178</td><td>1</td><td>178</td><td>1</td><td>177</td><td>2.54E-79</td><td>66.292</td><td>K02334</td><td>Not Included in Pathway or Brite</td><td>Unclassified: metabolism</td><td>dpo</td><td>DNA polymerase bacteriophage-type [EC:2.7.7.7]</td><td>—</td><td>—</td><td>0.00</td><td>0.00</td><td>0.00</td><td>0.00</td><td>0.00</td><td>0.49</td></tr>
<tr><td>Lizard06unclassified_bacterial_viruses_VIRSorter_scaffold_20046-gene_2|134|1348|1214|+|-|-|-|-|SpoIID|86.6|3.2e-26(+).p1</td><td>mbf:BHV42_04615</td><td>393</td><td>1</td><td>393</td><td>43</td><td>421</td><td>6.39E-104</td><td>45.547</td><td>K06381</td><td>Not Included in Pathway or Brite</td><td>Unclassified: signaling and cellular processes</td><td>spoIID</td><td>stage II sporulation protein D</td><td>—</td><td>—</td><td>0.00</td><td>0.00</td><td>0.00</td><td>0.00</td><td>0.00</td><td>381.84</td></tr>
<tr><td>Lizard06unclassified_bacterial_viruses_VIRSorter_scaffold_20046-gene_4|2892|3230|338|+|-|-|-|-|-|-|-|-(+).p1</td><td>mbf:BHV42_04620</td><td>106</td><td>1</td><td>104</td><td>427</td><td>531</td><td>3.24E-10</td><td>42.453</td><td>K03980</td><td>Brite Hierarchies</td><td>Protein families: metabolism</td><td>murJ, mviN</td><td>putative peptidoglycan lipid II flippase</td><td>—</td><td>—</td><td>0.00</td><td>0.00</td><td>0.00</td><td>0.00</td><td>0.00</td><td>1.53</td></tr>
<tr><td>Lizard06unclassified_bacterial_viruses_VIRSorter_scaffold_20046-gene_5|3240|3833|593|+|-|-|-|-|Sua5_yciO_yrdC|145.7|2.6e-44(+).p1</td><td>mbf:BHV42_04625</td><td>197</td><td>1</td><td>197</td><td>1</td><td>194</td><td>4.79E-66</td><td>53.299</td><td>K07566</td><td>Brite Hierarchies</td><td>Protein families: genetic information processing</td><td>tsaC, rimN, SUA5</td><td>L-threonylcarbamoyladenylate synthase [EC:2.7.7.87]</td><td>—</td><td>—</td><td>0.00</td><td>0.00</td><td>0.00</td><td>0.00</td><td>0.00</td><td>1.67</td></tr>
<tr><td>Lizard06unclassified_bacterial_viruses_VIRSorter_scaffold_3512-gene_2|1676|2041|365|+|Phage_cluster_11477|72.7|5.7e-</td><td>mbf:BHV42_08455</td><td>119</td><td>1</td><td>119</td><td>1</td><td>119</td><td>6.72E-52</td><td>62.185</td><td>K01737</td><td>Metabolism</td><td>Metabolism of cofactors and vitamins</td><td>queD, ptpS, PTS</td><td>6-pyruvoyltetrahydropterin/6-carboxytetrahydropterin synthase [EC:4.2.3.12 4.1.2.50]</td><td>ko00790</td><td>Folate biosynthesis</td><td>0.00</td><td>0.00</td><td>0.00</td><td>0.00</td><td>0.00</td><td>0.59</td></tr>
<tr><td>Lizard06unclassified_bacterial_viruses_VIRSorter_scaffold_52258-gene_1|1|1368|1367|+|-|-|-|-|Glyco_hydro_43|127.0|2.2e-38(+).p1</td><td>bcae:A4V03_06575</td><td>455</td><td>1</td><td>455</td><td>59</td><td>513</td><td>0</td><td>89.011</td><td>K06113</td><td>Not Included in Pathway or Brite</td><td>Unclassified: metabolism</td><td>abnA</td><td>arabinan endo-1,5-alpha-L-arabinosidase [EC:3.2.1.99]</td><td>—</td><td>—</td><td>0.00</td><td>34105.10</td><td>4451.27</td><td>0.00</td><td>20853.75</td><td>2762509.77</td></tr>
<tr><td>Lizard06unclassified_bacterial_viruses_VIRSorter_scaffold_52258-gene_2|1444|3366|1922|+|-|-|-|-|Pfam-B_9232|425.2|1.5e-129(+).p1</td><td>bxy:BXY_15710</td><td>636</td><td>1</td><td>636</td><td>17</td><td>652</td><td>0</td><td>92.925</td><td>K01209</td><td>Metabolism</td><td>Carbohydrate metabolism</td><td>abfA</td><td>alpha-L-arabinofuranosidase [EC:3.2.1.55]</td><td>ko00520</td><td>Amino sugar and nucleotide sugar metabolism</td><td>525.09</td><td>1268.21</td><td>218.86</td><td>0.00</td><td>64.99</td><td>2791.11</td></tr>
<tr><td>Lizard06unclassified_bacterial_viruses_VIRSorter_scaffold_71053-gene_3|1273|1704|431|-|Phage_cluster_9|155.9|1.8e-</td><td>fpr:FP2_05080</td><td>109</td><td>1</td><td>109</td><td>2</td><td>110</td><td>2.04E-49</td><td>65.138</td><td>K03111</td><td>Genetic Information Processing</td><td>Replication and repair</td><td>ssb</td><td>single-strand DNA-binding protein</td><td>ko03030,ko03430,ko03440</td><td>DNA replication|Mismatch repair|Homologous recombination</td><td>0.00</td><td>0.00</td><td>0.00</td><td>0.00</td><td>0.00</td><td>2.71</td></tr>
<tr><td>Lizard06unclassified_bacterial_viruses_VIRSorter_scaffold_30432-gene_1|1|1149|1148|+|Phage_cluster_405|101.4|1.3e-</td><td>pdi:BDI_1648</td><td>382</td><td>1</td><td>382</td><td>81</td><td>462</td><td>0</td><td>90.052</td><td>K11072</td><td>Environmental Information Processing</td><td>Membrane transport</td><td>potA</td><td>spermidine/putrescine transport system ATP-binding protein [EC:7.6.2.11]</td><td>ko02010</td><td>ABC transporters</td><td>0.00</td><td>0.00</td><td>0.00</td><td>0.00</td><td>0.00</td><td>11315.71</td></tr>
<tr><td>Lizard06unclassified_bacterial_viruses_VIRSorter_scaffold_30432-gene_2|1162|1959|797|+|-|-|-|-|BPD_transp_1|50.8|4.2e-15(+).p1</td><td>bcel:BcellWH2_05263</td><td>262</td><td>2</td><td>263</td><td>2</td><td>263</td><td>1.12E-158</td><td>83.206</td><td>K11071</td><td>Environmental Information Processing</td><td>Membrane transport</td><td>potB</td><td>spermidine/putrescine transport system permease protein</td><td>ko02010</td><td>ABC transporters</td><td>0.00</td><td>1.28</td><td>0.00</td><td>0.00</td><td>0.00</td><td>4.23</td></tr>
<tr><td>Lizard06unclassified_bacterial_viruses_VIRSorter_scaffold_30432-gene_3|1956|2756|800|+|-|-|-|-|BPD_transp_1|67.1|4.1e-20(+).p1</td><td>parc:CI960_06415</td><td>261</td><td>1</td><td>261</td><td>1</td><td>261</td><td>1.92E-166</td><td>90.805</td><td>K11070</td><td>Environmental Information Processing</td><td>Membrane transport</td><td>potC</td><td>spermidine/putrescine transport system permease protein</td><td>ko02010</td><td>ABC transporters</td><td>0.00</td><td>3.03</td><td>0.00</td><td>0.00</td><td>15.99</td><td>4.28</td></tr>
<tr><td>Lizard06unclassified_bacterial_viruses_VIRSorter_scaffold_30432-gene_4|2753|3284|531|+|-|-|-|-|-|-|-|-(+).p1</td><td>pdi:BDI_1645</td><td>162</td><td>16</td><td>177</td><td>17</td><td>178</td><td>1.77E-98</td><td>84.568</td><td>K11069</td><td>Environmental Information Processing</td><td>Membrane transport</td><td>potD</td><td>spermidine/putrescine transport system substrate-binding protein</td><td>ko02010</td><td>ABC transporters</td><td>0.00</td><td>0.00</td><td>0.00</td><td>0.00</td><td>0.00</td><td>1.07</td></tr>
</table>

Query ORF ID	Subject gene ID	Alignment length	Query start	Query end	Subject start	Subject end	e-value	Identity(%)	KO	Category	Subcategory	Gene name	Description	Pathway	Pathway definition	Abundance (fpkm)					
																(1) PE-NMC	(2) PE-NQ1	(3) PE-NQ2	(4) PT-SZZ	(5) PT-ZB	(6) PT-RT
Lizard06unclassified_bacterial_viruses_VIRSorter_scaffold_68291-gene_2|232|846|614|-|-|-|-|-|RNase_HII|158.0|7e-48(-).p2	pdi:BDI_3545	198	1	198	2	199	4.69E-114	79.798	**K03470**	Genetic Information Processing	Replication and repair	rnhB	ribonuclease HII [EC:3.1.26.4]	ko03030	DNA replication	0.00	0.00	0.00	0.00	0.00	171.77
Lizard06unclassified_bacterial_viruses_VIRSorter_scaffold_68291-gene_3|859|2073|1214|-|Phage_cluster_1231|161.1|9e-	parc:CI960_00830	403	1	403	2	404	0	78.908	**K11717**	Metabolism	Metabolism of other amino acids	sufS	cysteine desulfurase / selenocysteine lyase [EC:2.8.1.7 4.4.1.16]	ko00450	Selenocompound metabolism	0.00	0.00	0.00	0.00	0.00	77.32
Lizard06unclassified_bacterial_viruses_VIRSorter_scaffold_68291-gene_5|2473|2985|512|-|-|-|-|-|-|-|-|-(-).p1	hsw:Hsw_1619	179	1	158	1262	1435	5.84E-06	29.05	**K21449**	Brite Hierarchies	Protein families: signaling and cellular processes	ata, sadA, emaA	trimeric autotransporter adhesin	—	—	0.00	0.00	0.00	0.00	0.00	5.57
Lizard06unclassified_bacterial_viruses_VIRSorter_scaffold_69756-gene_2|1498|2481|983|-|Phage_cluster_8516|109.2|5.4e-	bcae:A4V03_08590	326	1	326	2	327	1.44E-162	65.644	**K07485**	Not Included in Pathway or Brite	Unclassified: genetic information processing	K07485	transposase	—	—	0.00	49.49	0.00	23.90	76.57	65.69
Lizard06unclassified_bacterial_viruses_VIRSorter_scaffold_69210-gene_1|222|1430|1208|+|gi_481019673_ref_YP_007878024.1_|52.0|1.8	mbf:BHV42_08100	401	1	401	1	401	0	89.027	**K02358**	Organismal Systems	Environmental adaptation	tuf, TUFM	elongation factor Tu	ko04626	Plant-pathogen interaction	162.64	0.00	120.30	129.00	328.78	252.64
Lizard06unclassified_bacterial_viruses_VIRSorter_scaffold_69210-gene_2|1529|2623|1094|-|-|-|-|-|Peptidase_M17|403.2|1.7e-122(-).p1	mbf:BHV42_03335	358	4	361	115	463	3.54E-163	63.128	**K01255**	Metabolism	Metabolism of other amino acids	CARP, pepA	leucyl aminopeptidase [EC:3.4.11.1]	ko00480	Glutathione metabolism	0.00	0.00	0.00	32.78	6.82	12.23
Lizard06unclassified_bacterial_viruses_VIRSorter_scaffold_13787-gene_2|297|674|377|-|-|-|-|-|-|-|-|-(-).p1	ddl:Desdi_3152	85	34	118	614	698	8.57E-18	52.941	**K21449**	Brite Hierarchies	Protein families: signaling and cellular processes	ata, sadA, emaA	trimeric autotransporter adhesin	—	—	0.00	0.00	0.00	0.00	70.21	4.97
Lizard06unclassified_bacterial_viruses_VIRSorter_scaffold_74353-gene_1|1|687|686|-|Phage_cluster_4482|128.4|5.5e-39|1|-|-|-(-).p2	pce:PECL_830	120	1	120	172	291	5.42E-18	25	**K21471**	Brite Hierarchies	Protein families: metabolism	cwlO	peptidoglycan DL-endopeptidase CwlO [EC:3.4.-.-]	—	—	0.00	0.00	0.00	0.00	62561.26	90525.70
Lizard06unclassified_bacterial_viruses_VIRSorter_scaffold_85218-gene_3|605|1288|683|+|Phage_cluster_932|182.6|1.8e-	mbf:BHV42_08185	224	4	226	3	219	2.60E-78	55.357	**K06920**	Metabolism	Metabolism of cofactors and vitamins	queC	7-cyano-7-deazaguanine synthase [EC:6.3.4.20]	ko00790	Folate biosynthesis	0.00	0.00	0.00	0.00	0.00	14.64
Lizard06unclassified_bacterial_viruses_VIRSorter_scaffold_85218-gene_4|1310|1885|575|+|Phage_cluster_9355|41.3|3e-	mbf:BHV42_08180	191	1	191	1	184	6.29E-73	61.78	**K09139**	Not Included in Pathway or Brite	Poorly characterized	K09139	uncharacterized protein	—	—	0.00	0.00	0.00	0.00	0.00	4.51
Lizard06unclassified_bacterial_viruses_VIRSorter_scaffold_8036-gene_3|484|1062|578|+|Phage_cluster_388|157.1|1.1e-	cls:CXIVA_24410	178	14	185	14	184	2.78E-55	49.438	**K01495**	Metabolism	Metabolism of cofactors and vitamins	GCH1, folE	GTP cyclohydrolase IA [EC:3.5.4.16]	ko00790	Folate biosynthesis	0.00	0.00	0.00	0.00	0.00	15.25
Lizard06unclassified_bacterial_viruses_VIRSorter_scaffold_12219-gene_1|323|1420|1097|+|Phage_cluster_152|274.0|5.2e-	bkw:BkAM31D_02480	360	3	361	2	355	9.76E-73	38.611	**K00986**	Not Included in Pathway or Brite	Unclassified: metabolism	ltrA	RNA-directed DNA polymerase [EC:2.7.7.49]	—	—	0.00	27.54	13.78	0.00	0.00	42.65
Lizard06unclassified_bacterial_viruses_VIRSorter_scaffold_49264-gene_1|64|771|707|-|Phage_cluster_157|175.8|3.7e-53|0|-|-|-(-).p1	ppo:PPM_2769	207	6	212	12	218	7.68E-107	70.048	**K06909**	Not Included in Pathway or Brite	Unclassified: genetic information processing	xtmB	phage terminase large subunit	—	—	564.16	926.77	139.10	0.00	814.60	322.87
Lizard06unclassified_bacterial_viruses_VIRSorter_scaffold_13973-gene_2|884|1525|641|+|Phage_cluster_176|96.5|4.6e-	osp:Odosp_2085	212	1	212	1	212	2.28E-88	59.434	**K07482**	Not Included in Pathway or Brite	Unclassified: genetic information processing	K07482	transposase, IS30 family	—	—	122.53	11.74	0.00	0.00	0.00	9.46
Lizard06unclassified_bacterial_viruses_VIRSorter_scaffold_37963-gene_1|118|429|311|+|Phage_cluster_400|96.2|2.5e-	aza:AZKH_1363	91	1	91	1	91	2.59E-18	42.857	**K04764**	Brite Hierarchies	Protein families: genetic information processing	ihfA, himA	integration host factor subunit alpha	—	—	0.00	0.00	0.00	0.00	0.00	4.17
Lizard06unclassified_bacterial_viruses_VIRSorter_scaffold_89405-gene_4|1005|1430|425|+|Phage_cluster_24|215.0|4.6e-	crz:D1345_00365	141	1	140	1	141	2.47E-47	58.156	**K01185**	Not Included in Pathway or Brite	Unclassified: metabolism	E3.2.1.17	lysozyme [EC:3.2.1.17]	—	—	0.00	0.00	0.00	0.00	0.00	0.49
Lizard06unclassified_bacterial_viruses_VIRSorter_scaffold_113913-gene_2|909|1226|317|-|Phage_cluster_400|78.2|1e-	banc:PU02_1111	92	2	93	1	91	1.09E-14	34.783	**K03530**	Brite Hierarchies	Protein families: genetic information processing	hupB	DNA-binding protein HU-beta	—	—	0.00	0.00	0.00	0.00	0.00	0.54
Lizard06unclassified_bacterial_viruses_VIRSorter_scaffold_93692-gene_2|219|542|323|-|Phage_cluster_142|92.7|8.8e-28|2|-|-|-(-).p1	rto:RTO_01470	89	2	90	250	338	6.13E-33	61.798	**K04763**	Brite Hierarchies	Protein families: genetic information processing	xerD	integrase/recombinase XerD	—	—	0.00	0.00	0.00	0.00	0.00	2.25
Lizard06unclassified_dsDNA_phages_VIRSorter_scaffold_29337-gene_2|2861|4063|1202|+|-|-|-|-|PALP|150.8|1.3e-46(+).p1	parc:CI960_06900	392	1	392	1	392	0	92.092	**K01696**	Metabolism	Amino acid metabolism	trpB	tryptophan synthase beta chain [EC:4.2.1.20]	ko00260,ko00400	Glycine, serine and threonine metabolism|Phenylalanine, tyrosine and tryptophan biosynthesis	0.00	39.91	0.00	0.00	0.00	321.23
Lizard06unclassified_dsDNA_phages_VIRSorter_scaffold_29337-gene_3|4105|5508|1403|+|-|-|-|-|Chorismate_bind|256.5|6.3e-79(+).p1	pdi:BDI_0591	467	1	467	1	467	0	86.938	**K01657**	Metabolism	Amino acid metabolism	trpE	anthranilate synthase component I [EC:4.1.3.27]	ko00400,ko00405,ko02024,ko02025	Phenylalanine, tyrosine and tryptophan biosynthesis|Phenazine biosynthesis|Quorum sensing|Biofilm formation - Pseudomonas aeruginosa	0.00	41.90	0.00	0.00	0.00	484.68
Lizard06unclassified_dsDNA_phages_VIRSorter_scaffold_29337-gene_4|5557|6123|566|+|-|-|-|-|GATase|164.3|5.7e-51(+).p1	pdi:BDI_0590	187	1	187	1	187	5.52E-122	89.305	**K01658**	Metabolism	Amino acid metabolism	trpG	anthranilate synthase component II [EC:4.1.3.27]	ko00400,ko00405,ko02024,ko02025	Phenylalanine, tyrosine and tryptophan biosynthesis|Phenazine biosynthesis|Quorum sensing|Biofilm formation - Pseudomonas aeruginosa	0.00	5.34	0.00	0.64	0.00	119.26
Lizard06unclassified_dsDNA_phages_VIRSorter_scaffold_29337-gene_5|6136|7131|995|+|-|-|-|-|Glycos_transf_3|216.3|1.2e-66(+).p1	parc:CI960_06885	330	1	330	1	330	0	89.697	**K00766**	Metabolism	Amino acid metabolism	trpD	anthranilate phosphoribosyltransferase [EC:2.4.2.18]	ko00400	Phenylalanine, tyrosine and tryptophan biosynthesis	0.00	26.58	0.00	0.58	0.00	283.20
Lizard06unclassified_dsDNA_phages_VIRSorter_scaffold_29337-gene_6|7140|7949|809|+|-|-|-|-|IGPS|241.0|2.7e-74(+).p1	pdi:BDI_0588	264	2	265	3	260	5.49E-136	71.97	**K01609**	Metabolism	Amino acid metabolism	trpC	indole-3-glycerol phosphate synthase [EC:4.1.1.48]	ko00400	Phenylalanine, tyrosine and tryptophan biosynthesis	0.00	15.37	0.00	0.00	0.00	152.76
Lizard06unclassified_dsDNA_phages_VIRSorter_scaffold_29337-gene_7|7946|8560|614|+|-|-|-|-|PRAI|123.0|2.9e-38(+).p1	pdi:BDI_0587	206	1	202	1	206	2.50E-84	60.194	**K01817**	Metabolism	Amino acid metabolism	trpF	phosphoribosylanthranilate isomerase [EC:5.3.1.24]	ko00400	Phenylalanine, tyrosine and tryptophan biosynthesis	0.00	7.84	0.00	0.00	1.31	85.44
Lizard06unclassified_dsDNA_phages_VIRSorter_scaffold_29337-gene_8|8585|9394|809|+|-|-|-|-|Trp_syntA|257.2|2.2e-79(+).p1	pdi:BDI_0586	257	1	257	1	257	3.17E-157	80.934	**K01695**	Metabolism	Amino acid metabolism	trpA	tryptophan synthase alpha chain [EC:4.2.1.20]	ko00260,ko00400	Glycine, serine and threonine metabolism|Phenylalanine, tyrosine and tryptophan biosynthesis	0.00	12.61	0.00	0.00	0.00	162.52
Lizard06unclassified_dsDNA_phages_VIRSorter_scaffold_29337-gene_9|9478|10842|1364|-|-|-|-|-|OEP|165.0|3.6e-51(-).p1	bdo:EL88_19915	455	1	453	3	457	0	74.725	**K18139**	Cellular Processes	Cellular community - prokaryotes	oprM, emhC, ttgC, cusC, adeK, smeF, mtrE, cmeC,	outer membrane protein, multidrug efflux system	ko01501,ko02024	beta-Lactam resistance|Quorum sensing	0.00	35.36	0.00	0.00	7.70	389.11
Lizard06unclassified_dsDNA_phages_VIRSorter_scaffold_29337-gene_11|14056|15042|986|-|-|-|-|-|HlyD|106.4|4.3e-33(-).p1	bcel:BcellWH2_03475	322	1	322	47	368	0	79.503	**K03585**	Human Diseases	Drug resistance: antimicrobial	acrA, mexA, adeI, smeD, mtrC, cmeA	membrane fusion protein, multidrug efflux system	ko01501,ko01503	beta-Lactam resistance|Cationic antimicrobial peptide (CAMP) resistance	0.00	12.24	0.00	0.00	3.71	204.38
Lizard06unclassified_dsDNA_phages_VIRSorter_scaffold_29337-gene_12|16697|17221|524|-|-|-|-|-|SusD|108.8|8.5e-34(-).p2	bfs:BF9343_0495	173	1	173	483	655	1.77E-110	90.751	**K21572**	Brite Hierarchies	Protein families: signaling and cellular processes	susD	starch-binding outer membrane protein, SusD/RagB family	—	—	0.00	7.02	0.00	0.00	0.62	104.81
Lizard06unclassified_dsDNA_phages_VIRSorter_scaffold_29337-gene_13|17250|18230|980|-|-|-|-|-|SusD-like_3|48.3|3.1e-15(-).p2	bfs:BF9343_0495	321	1	321	17	337	0	91.277	**K21572**	Brite Hierarchies	Protein families: signaling and cellular processes	susD	starch-binding outer membrane protein, SusD/RagB family	—	—	0.00	17.62	0.52	0.21	3.02	208.17
Lizard06unclassified_dsDNA_phages_VIRSorter_scaffold_9745-gene_2|286|3333|3047|-|-|-|-|-|ACR_tran|808.7|1.4e-245(-).p1	osp:Odosp_2536	1050	1	1014	2	1051	0	89.524	**K03296**	Not Included in Pathway or Brite	Unclassified: signaling and cellular processes	TC.HAE1	hydrophobic/amphiphilic exporter-1 (mainly G- bacteria), HAE1 family	—	—	631.23	114.06	31.13	1814.84	4630.12	14902.40
Lizard06unclassified_dsDNA_phages_VIRSorter_scaffold_9745-gene_3|3425|4462|1037|-|-|-|-|-|HlyD_2|96.0|6.3e-30(-).p1	osp:Odosp_2535	344	1	344	2	345	0	90.407	**K03585**	Human Diseases	Drug resistance: antimicrobial	acrA, mexA, adeI, smeD, mtrC, cmeA	membrane fusion protein, multidrug efflux system	ko01501,ko01503	beta-Lactam resistance|Cationic antimicrobial peptide (CAMP) resistance	10.54	0.87	0.00	0.00	6.09	91.50
Lizard06unclassified_dsDNA_phages_VIRSorter_scaffold_9745-gene_5|5922|6563|641|-|-|-|-|-|TetR_N|44.1|3.1e-14(-).p1	osp:Odosp_2533	203	1	203	1	203	9.86E-114	75.862	**K22108**	Brite Hierarchies	Protein families: genetic information processing	kstR2	TetR/AcrR family transcriptional regulator, cholesterol catabolism regulator	—	—	0.00	0.00	0.00	0.60	0.00	5.73
Lizard06unclassified_dsDNA_phages_VIRSorter_scaffold_9745-gene_10|9913|11463|1550|-|-|-|-|-|SusD|94.5|2e-29(-).p1	est:DN752_15030	527	3	515	11	512	1.79E-88	35.863	**K21572**	Brite Hierarchies	Protein families: signaling and cellular processes	susD	starch-binding outer membrane protein, SusD/RagB family	—	—	0.00	0.00	0.00	0.00	2.10	5.76
Lizard06unclassified_dsDNA_phages_VIRSorter_scaffold_9745-gene_11|11480|14806|3326|-|-|-|-|-|Plug|69.9|5.2e-22(-).p1	est:DN752_15035	1056	72	1107	21	1064	0	43.277	**K21573**	Brite Hierarchies	Protein families: signaling and cellular processes	susC	TonB-dependent starch-binding outer membrane protein SusC	—	—	0.00	0.00	0.00	0.62	3.63	5.10
Lizard06unclassified_dsDNA_phages_VIRSorter_scaffold_5163-gene_1|127|477|350|-|-|-|-|-|ACR_tran|121.0|7.8e-38(-).p1	parc:CI960_04535	115	1	115	911	1025	1.98E-61	89.565	**K15726**	Brite Hierarchies	Protein families: signaling and cellular processes	czcA, cusA, cnrA	heavy metal efflux system protein	—	—	0.00	0.00	35.05	0.00	0.00	27.12
Lizard06unclassified_dsDNA_phages_VIRSorter_scaffold_5163-gene_2|699|1319|620|+|-|-|-|-|-|-|-|-|-(+).p1	parc:CI960_04520	127	75	201	1	127	2.90E-74	84.252	**K01302**	Brite Hierarchies	Protein families: metabolism	CPQ	carboxypeptidase Q [EC:3.4.17.-]	—	—	51.65	4.24	0.00	0.00	0.00	5.63
Lizard06unclassified_dsDNA_phages_VIRSorter_scaffold_5163-gene_3|1321|2037|716|+|-|-|-|-|Peptidase_M28|61.5|2.2e-19(+).p1	pdi:BDI_0222	238	1	238	199	436	8.22E-159	89.496	**K01302**	Brite Hierarchies	Protein families: metabolism	CPQ	carboxypeptidase Q [EC:3.4.17.-]	—	—	0.00	3.37	0.00	0.00	0.00	3.35

Table S4. The results of ARGs annotation of phage-encoded genes based on CARD database.

Query ORF ID	Subject ARG ID	Alignment length	query start	query end	subject start	subject end	e-value	Identity(%)	ARO Accession	CVTERM ID	Model ID	ARO Name	Protein Accession	DNA Accession	AMR Gene Family	Drug Class	Resistance Mechanism	Abundance (ppm)					
																		(1) PE-NMC	(2) PE-NQ1	(3) PE-NQ2	(4) PT-SZZ	(5) PT-ZB	(6) PT-RT
Lizard01unclassified_dsDNA_phages_VIRSorter_scaffold_3158-gene_4|5339|6358|1019|+|-|-|-|-|HlyD|95.1|1.5e-30(+).p1	gb|ABS43901.1|ARO:3000783|cmeA	327	14	336	45	362	1.25E-42	31.193	ARO:3000783	37163	1181	cmeA	ABS43901.1	CP000768	resistance-nodulation-cell division (RND) antibiotic efflux pump	cephalosporin;fluoroquinolone antibiotic;fusidic acid;macrolide antibiotic	antibiotic efflux	5.55	273.53	25.95	14.61	11.07	215.57
Lizard01unclassified_dsDNA_phages_VIRSorter_scaffold_3158-gene_5|6371|9355|2984|+|-|-|-|-|ACR_tran|942.7|6.1e-287(+).p1	gb|ABS43151.1|ARO:3000784|cmeB	1002	4	994	4	991	0	38.922	ARO:3000784	37164	748	cmeB	ABS43151.1	CP000768	resistance-nodulation-cell division (RND) antibiotic efflux pump	cephalosporin;fluoroquinolone antibiotic;fusidic acid;macrolide antibiotic	antibiotic efflux	12.99	595.84	52.78	24.94	29.07	588.77
Lizard02Bicaudaviridae_VIRSorter_scaffold_3586-gene_2|765|1502|737|+|Phage_cluster_8323|46.8|1.7e-	gb|AAC75314.1|ARO:3003578|pmrF	215	6	216	12	211	3.18E-15	27.907	ARO:3003578	40188	2014	PmrF	AAC75314.1	U00096	pmr phosphoethanolamine transferase	peptide antibiotic	antibiotic target alteration	0.00	1.82	0.34	1.24	0.30	24.95
Lizard02unclassified_dsDNA_phages_VIRSorter_scaffold_22892-gene_4|4967|5365|398|-|Phage_cluster_3079|90.1|5.7e-	gb|ADM92605.1|ARO:3000553|adeR	101	14	114	16	115	8.28E-18	38.614	ARO:3000553	36692	456	adeR	ADM92605.1	HM440348	resistance-nodulation-cell division (RND) antibiotic efflux pump	glycylcycline;tetracycline antibiotic	antibiotic efflux	0.05	2.26	0.14	0.08	0.00	2.89
Lizard02unclassified_dsDNA_phages_VIRSorter_scaffold_22892-gene_6|7316|10462|3146|-|-|-|-|-|ACR_tran|889.6|1.1e-269(-).p1	gb|CAH14033.1|ARO:3004100|LpeB	1044	3	1038	2	998	1.97E-85	25.575	ARO:3004100	41209	2759	LpeB	CAH14033.1	CR628336.1	resistance-nodulation-cell division (RND) antibiotic efflux pump	macrolide antibiotic	antibiotic efflux	1.02	117.47	3.66	1.94	6.20	55.84
Lizard02unclassified_dsDNA_phages_VIRSorter_scaffold_22892-gene_7|10554|11552|998|-|-|-|-|-|HlyD_2|90.2|7.1e-28(-).p1	gb|AAG07762.1|ARO:3003030|mexV	324	15	317	53	358	5.49E-16	26.235	ARO:3003030	39464	2207	MexV	AAG07762.1	AE004091.2	resistance-nodulation-cell division (RND) antibiotic efflux pump	acridine dye;fluoroquinolone antibiotic;macrolide antibiotic;phenicol antibiotic;tetracycline antibiotic	antibiotic efflux	0.18	17.53	1.63	0.00	0.15	8.34
Lizard02unclassified_dsDNA_phages_VIRSorter_scaffold_22892-gene_19|26016|27263|1247|-|Phage_cluster_3804|274.5|6.6e-	gb|NP_207975.1|ARO:3003965|hp1184	398	2	395	38	432	9.41E-43	29.146	ARO:3003965	40736	2431	hp1184	NP_207975.1	NC_000915.1	multidrug and toxic compound extrusion (MATE) transporter	fluoroquinolone antibiotic;macrolide antibiotic;nitroimidazole antibiotic;tetracycline antibiotic	antibiotic efflux	0.00	5.51	0.68	0.54	1.18	8.58
Lizard02unclassified_dsDNA_phages_VIRSorter_scaffold_49327-gene_3|6878|7864|986|+|-|-|-|-|HlyD|106.4|8.8e-33(+).p1	gb|ABS43901.1|ARO:3000783|cmeA	323	2	321	48	362	3.17E-43	30.65	ARO:3000783	37163	1181	cmeA	ABS43901.1	CP000768	resistance-nodulation-cell division (RND) antibiotic efflux pump	cephalosporin;fluoroquinolone antibiotic;fusidic acid;macrolide antibiotic	antibiotic efflux	0.00	6.39	0.00	0.00	0.74	468.60
Lizard02unclassified_dsDNA_phages_VIRSorter_scaffold_49327-gene_4|7884|11081|3197|+|-|-|-|-|ACR_tran|981.5|1.9e-297(+).p1	gb|ABS43151.1|ARO:3000784|cmeB	1039	4	1032	4	1029	0	37.921	ARO:3000784	37164	748	cmeB	ABS43151.1	CP000768	resistance-nodulation-cell division (RND) antibiotic efflux pump	cephalosporin;fluoroquinolone antibiotic;fusidic acid;macrolide antibiotic	antibiotic efflux	0.00	40.96	0.14	0.08	7.38	2054.57
Lizard02unclassified_dsDNA_phages_VIRSorter_scaffold_49327-gene_5|11078|12442|1364|+|-|-|-|-|OEP|165.0|7.4e-51(+).p1	gb|NP_249118.1|ARO:3000379|OprM	470	1	452	1	466	2.32E-48	28.936	ARO:3000379	36518	1305	OprM	AAG03816.1	AE004091.2	resistance-nodulation-cell division (RND) antibiotic efflux pump	acridinedye;aminocoumarin;aminoglycoside;carbapenem;cephalosporin;cephamycin;diaminopyrimidinec;fluoroquinolone;macrolide;monobactam;penam;penem;peptide;phenicol;sulfonamide;tetracycl	antibiotic efflux	0.00	12.46	0.00	0.00	1.03	601.29
Lizard02unclassified_dsDNA_phages_VIRSorter_scaffold_7706-gene_2|1223|4504|3281|-|-|-|-|-|ACR_tran|358.6|2.7e-109(-).p1	gb|EGP45231.1|ARO:3004144|AxyY	1077	13	1050	17	1018	1.48E-32	19.406	ARO:3004144	41273	2799	AxyY	EGP45231.1	AFRQ01000061.1	resistance-nodulation-cell division (RND) antibiotic efflux pump	aminoglycoside antibiotic;cephalosporin;fluoroquinolone antibiotic;macrolide antibiotic	antibiotic efflux	0.09	13.01	0.14	2.18	0.74	4.65
Lizard02unclassified_dsDNA_phages_VIRSorter_scaffold_7706-gene_4|5971|9039|3068|-|-|-|-|-|ACR_tran|576.4|4.4e-175(-).p1	gb|YP_490697.1|ARO:3000491|acrD	1080	2	1018	3	1028	2.33E-70	23.704	ARO:3000491	36630	1427	acrD	BAA16344.1	AP009048.1	resistance-nodulation-cell division (RND) antibiotic efflux pump	aminoglycoside antibiotic	antibiotic efflux	0.18	14.44	0.20	2.25	0.59	3.77
Lizard02unclassified_dsDNA_phages_VIRSorter_scaffold_7706-gene_5|9043|10122|1079|-|-|-|-|-|HlyD_2|138.9|1.1e-42(-).p1	gb|NP_252895.1|ARO:3000807|mexH	224	144	358	138	355	3.08E-10	24.554	ARO:3000807	37187	1344	MexH	AAG07593.1	AE004091.2	resistance-nodulation-cell division (RND) antibiotic efflux pump	acridine dye;fluoroquinolone antibiotic;tetracycline antibiotic	antibiotic efflux	0.18	4.41	0.07	1.09	0.00	1.28
Lizard02unclassified_dsDNA_phages_VIRSorter_scaffold_9540-gene_1|50|1423|1373|-|-|-|-|-|OEP|174.3|1e-53(-).p1	gb|CAA64891.1|ARO:3000812|mtrE	456	6	453	9	458	1.78E-51	30.044	ARO:3000812	37192	453	mtrE	CAA64891.1	X95635	resistance-nodulation-cell division (RND) antibiotic efflux pump	macrolide antibiotic;penam	antibiotic efflux	1.25	166.36	2.37	13.67	1.77	135.26
Lizard02unclassified_dsDNA_phages_VIRSorter_scaffold_9540-gene_2|1413|2882|1469|-|-|-|-|-|ACR_tran|378.9|2e-115(-).p1	gb|EGP45231.1|ARO:3004144|AxyY	409	32	440	614	1021	7.52E-87	37.408	ARO:3004144	41273	2799	AxyY	EGP45231.1	AFRQ01000061.1	resistance-nodulation-cell division (RND) antibiotic efflux pump	aminoglycoside antibiotic;cephalosporin;fluoroquinolone antibiotic;macrolide antibiotic	antibiotic efflux	0.79	175.52	1.69	12.74	4.87	167.75
Lizard02unclassified_dsDNA_phages_VIRSorter_scaffold_9540-gene_3|2864|4414|1550|-|-|-|-|-|ACR_tran|500.7|3.1e-152(-).p1	gb|ABS43151.1|ARO:3000784|cmeB	515	1	506	29	535	7.06E-131	42.524	ARO:3000784	37164	748	cmeB	ABS43151.1	CP000768	resistance-nodulation-cell division (RND) antibiotic efflux pump	cephalosporin;fluoroquinolone antibiotic;fusidic acid;macrolide antibiotic	antibiotic efflux	0.18	128.77	1.15	7.69	2.80	127.96
Lizard02unclassified_dsDNA_phages_VIRSorter_scaffold_9540-gene_4|4411|5280|869|-|-|-|-|-|HlyD|104.0|4.8e-32(-).p1	gb|AAD51344.1|ARO:3003051|smeA	246	33	276	65	310	1.96E-33	32.927	ARO:3003051	39485	1507	smeA	AAD51344.1	AF173226	resistance-nodulation-cell division (RND) antibiotic efflux pump	aminoglycoside antibiotic;cephalosporin;cephamycin;penam	antibiotic efflux	0.00	32.58	0.41	0.31	0.30	32.65
Lizard02unclassified_dsDNA_phages_VIRSorter_scaffold_3622-gene_6|5420|8320|2900|-|-|-|-|-|ACR_tran|771.7|4.3e-234(-).p1	gb|BAE06006.1|ARO:3003705|mexN	964	3	932	95	1014	1.24E-117	28.942	ARO:3003705	40359	2217	mexN	BAE06006.1	AB219523.1	resistance-nodulation-cell division (RND) antibiotic efflux pump	phenicol antibiotic	antibiotic efflux	0.09	62.84	0.27	8.16	0.59	3.45
Lizard02unclassified_dsDNA_phages_VIRSorter_scaffold_3622-gene_7|8598|9317|719|-|-|-|-|-|HlyD_2|85.2|2.4e-26(-).p1	gb|AAG07762.1|ARO:3003030|mexV	231	25	236	136	366	3.25E-22	30.736	ARO:3003030	39464	2207	MexV	AAG07762.1	AE004091.2	resistance-nodulation-cell division (RND) antibiotic efflux pump	acridine dye;fluoroquinolone antibiotic;macrolide antibiotic;phenicol antibiotic;tetracycline antibiotic	antibiotic efflux	0.09	6.28	0.00	0.70	0.00	0.32
Lizard02unclassified_dsDNA_phages_VIRSorter_scaffold_3622-gene_8|9347|10234|887|-|-|-|-|-|OEP|145.9|5.4e-45(-).p1	gb|NP_253661.1|ARO:3003682|OpmH	134	160	291	304	437	2.18E-08	26.866	ARO:3003682	40317	2195	OpmH	AAG08359.1	AE004091.2	resistance-nodulation-cell division (RND) antibiotic efflux pump	triclosan	antibiotic efflux	0.14	14.11	0.14	2.95	0.00	1.28
Lizard02unclassified_dsDNA_phages_VIRSorter_scaffold_3622-gene_10|10492|11148|656|-|-|-|-|-|TetR_N|49.0|1.7e-15(-).p1	gb|NP_252368.1|ARO:3003710|mexL	90	14	102	19	103	5.07E-07	32.222	ARO:3003710	40364	2219	MexL	AAG07066.1	AE004091.2	resistance-nodulation-cell division (RND) antibiotic efflux pump	macrolide antibiotic;tetracycline antibiotic;triclosan	antibiotic efflux	0.09	4.96	0.14	0.54	0.00	0.72
Lizard02unclassified_dsDNA_phages_VIRSorter_scaffold_22609-gene_5|3574|6594|3020|-|-|-|-|-|ACR_tran|822.9|1.5e-249(-).p1	gb|AAC75137.1|ARO:3000794|mdtC	1012	1	1002	37	1021	9.91E-124	27.668	ARO:3000794	37174	1315	mdtC	AAC75137.1	U00096	resistance-nodulation-cell division (RND) antibiotic efflux pump	aminocoumarin antibiotic	antibiotic efflux	0.00	190.45	0.00	132.07	18.44	27.84
Lizard02unclassified_dsDNA_phages_VIRSorter_scaffold_22609-gene_6|6712|7722|1010|-|-|-|-|-|HlyD_2|119.8|7e-37(-).p1	gb|AAG07762.1|ARO:3003030|mexV	279	78	333	88	366	3.32E-30	31.541	ARO:3003030	39464	2207	MexV	AAG07762.1	AE004091.2	resistance-nodulation-cell division (RND) antibiotic efflux pump	acridine dye;fluoroquinolone antibiotic;macrolide antibiotic;phenicol antibiotic;tetracycline antibiotic	antibiotic efflux	0.00	18.74	0.20	11.11	0.15	2.73
Lizard02unclassified_dsDNA_phages_VIRSorter_scaffold_22609-gene_7|7780|9069|1289|-|-|-|-|-|OEP|212.1|2.6e-65(-).p1	gb|BAO79432.1|ARO:3000785|cmeC	449	2	424	64	466	8.93E-13	23.608	ARO:3000785	37165	798	cmeC	BAO79432.1	AB894099	resistance-nodulation-cell division (RND) antibiotic efflux pump	cephalosporin;fluoroquinolone antibiotic;fusidic acid;macrolide antibiotic	antibiotic efflux	0.00	3.97	0.00	2.10	1.48	1.20
Lizard02unclassified_dsDNA_phages_VIRSorter_scaffold_7484-gene_1|1|2841|2840|-|-|-|-|-|ACR_tran|478.9|1.2e-145(-).p1	gb|AAC75137.1|ARO:3000794|mdtC	979	3	943	6	935	5.60E-60	23.493	ARO:3000794	37174	1315	mdtC	AAC75137.1	U00096	resistance-nodulation-cell division (RND) antibiotic efflux pump	aminocoumarin antibiotic	antibiotic efflux	0.00	6.78	0.00	0.16	0.30	1.44
Lizard02unclassified_dsDNA_phages_VIRSorter_scaffold_7484-gene_2|2838|3872|1034|-|-|-|-|-|HlyD_2|129.1|1.1e-39(-).p1	gb|NP_252367.1|ARO:3003692|mexJ	293	48	328	51	341	6.18E-14	25.939	ARO:3003692	40340	2205	MexJ	AAG07065.1	AE004091.2	resistance-nodulation-cell division (RND) antibiotic efflux pump	macrolide antibiotic;tetracycline antibiotic;triclosan	antibiotic efflux	0.09	2.20	0.00	0.16	0.30	1.20
Lizard02unclassified_dsDNA_phages_VIRSorter_scaffold_53624-gene_1|32|1948|1916|-|-|-|-|-|ACR_tran|551.6|1.3e-167(-).p1	gb|AAG07763.1|ARO:3003031|mexW	633	1	626	376	1003	2.97E-138	37.915	ARO:3003031	39465	2208	MexW	AAG07763.1	NC_002516.2	resistance-nodulation-cell division (RND) antibiotic efflux pump	acridine dye;fluoroquinolone antibiotic;macrolide antibiotic;phenicol antibiotic;tetracycline antibiotic	antibiotic efflux	0.09	33.52	0.61	1.55	0.59	25.67
Lizard02unclassified_dsDNA_phages_VIRSorter_scaffold_53624-gene_2|1963|2964|1001|-|-|-|-|-|ACR_tran|324.0|7.4e-99(-).p2	gb|AAG07763.1|ARO:3003031|mexW	325	7	331	8	332	1.01E-56	28.923	ARO:3003031	39465	2208	MexW	AAG07763.1	NC_002516.2	resistance-nodulation-cell division (RND) antibiotic efflux pump	acridine dye;fluoroquinolone antibiotic;macrolide antibiotic;phenicol antibiotic;tetracycline antibiotic	antibiotic efflux	0.23	19.46	0.00	0.93	0.30	21.90
Lizard02unclassified_dsDNA_phages_VIRSorter_scaffold_53624-gene_3|2980|3642|662|-|-|-|-|-|HlyD_2|85.6|1.8e-26(-).p2	gb|AAG07762.1|ARO:3003030|mexV	242	1	215	134	366	1.95E-27	29.752	ARO:3003030	39464	2207	MexV	AAG07762.1	AE004091.2	resistance-nodulation-cell division (RND) antibiotic efflux pump	acridine dye;fluoroquinolone antibiotic;macrolide antibiotic;phenicol antibiotic;tetracycline antibiotic	antibiotic efflux	0.05	10.97	0.00	0.85	0.00	12.27
Lizard02UnclassifiedPhage_VIRSorter_scaffold_597-gene_5|3345|4661|1316|-|Phage_cluster_2466|59.9|3.3e-	gb|WP_011461302.1|ARO:3003726|vanSI	153	291	437	239	381	1.31E-12	29.412	ARO:3003726	40383	2244	vanSI	WP_011461302.1	NC_007907.1	glycopeptide resistance gene cluster;vanS	glycopeptide antibiotic	antibiotic target alteration	4.76	279.81	22.70	64.87	38.22	155.56
Lizard02UnclassifiedPhage_VIRSorter_scaffold_597-gene_9|7301|8356|1055|+|-|-|-|-|HlyD_2|65.7|8.7e-20(+).p1	gb|BAE78084.1|ARO:3003548|mdtN	220	51	265	49	265	8.42E-08	25.455	ARO:3003548	40150	1442	mdtN	BAE78084.1	AP009048.1	major facilitator superfamily (MFS) antibiotic efflux pump	acridine dye;nucleoside antibiotic	antibiotic efflux	5.73	596.83	59.21	184.05	58.28	184.92
Lizard02UnclassifiedPhage_VIRSorter_scaffold_1367-gene_4|2449|3831|1382|-|Phage_cluster_405|181.7|5e-	gb|AAV85982.1|ARO:3000535|macB	202	5	196	2	202	5.97E-29	32.178	ARO:3000535	36674	487	macB	AAV85982.1	AY768532	ATP-binding cassette (ABC) antibiotic efflux pump	macrolide antibiotic	antibiotic efflux	0.00	6.01	0.00	1.01	0.00	5.94
Lizard02UnclassifiedPhage_VIRSorter_scaffold_60490-gene_3|1055|2185|1130|-|Phage_cluster_405|163.6|1.4e-	gb|APB03214.1|ARO:3003980|tetA(48)	211	14	223	26	235	2.40E-26	30.332	ARO:3003980	40861	2517	tetA(58)	APB03214.1	KX531044.1	major facilitator superfamily (MFS) antibiotic efflux pump	tetracycline antibiotic	antibiotic efflux	0.00	1.21	0.00	0.00	0.00	0.00
Lizard02UnclassifiedPhage_VIRSorter_scaffold_70498-gene_2|331|1224|893|+|-|-|-|-|LysR_substrate|126.2|2e-38(+).p1	gb|ALH22601.1|ARO:3000620|adeL	288	5	287	1	288	7.38E-38	27.778	ARO:3000620	36962	2654	adeL	ALH22601.1	KR297239.1	resistance-nodulation-cell division (RND) antibiotic efflux pump	fluoroquinolone antibiotic;tetracycline antibiotic	antibiotic efflux	0.00	0.66	0.00	0.00	0.00	0.00
Lizard04Caudovirales_VIRSorter_scaffold_11156-gene_7|7852|8946|1094|+|Phage_cluster_398|134.6|2.3e-	gb|NP_252244|ARO:3002985|arnA	379	17	353	299	649	4.79E-14	25.33	ARO:3002985	39419	1643	arnA	AAG06942.1	AE004091.2	pmr phosphoethanolamine transferase	peptide antibiotic	antibiotic target alteration	0.00	0.00	0.00	23.54	3.98	53.83
Lizard04Caudovirales_VIRSorter_scaffold_11156-gene_8|8940|10199|1259|+|Phage_cluster_3799|141.9|1.3e-	gb|AAC75089.1|ARO:3003577|pmrE	370	1	366	1	342	5.43E-32	28.108	ARO:3003577	40187	842	ugd	AAC75089.1	U00096	pmr phosphoethanolamine transferase	peptide antibiotic	antibiotic target alteration	0.00	0.00	0.00	76.37	10.62	165.26
Lizard04Caudovirales_VIRSorter_scaffold_21291-gene_16|14288|17056|2768|+|-|-|-|-|tRNA-synt_1_2|216.8|9.9e-66(+).p1	gb|YP_003971446|ARO:3003730|Bifidobacteria	163	4	143	27	188	1.38E-06	26.994	ARO:3003730	40419	2278	ileS	ADP36409.1	CP001840.1	antibiotic-resistant isoleucyl-tRNA synthetase (ileS)	mupirocin	antibiotic target alteration	0.00	0.06	0.00	89.19	8.26	798.72
Lizard04Caudovirales_VIRSorter_scaffold_8420-gene_4|6303|7160|857|+|-|-|-|-|Pterin_bind|226.2|1.9e-68(+).p1	gb|ACJ63260.1|ARO:3000413|sul3	263	22	275	3	258	2.37E-34	32.319	ARO:3000413	36552	396	sul3	ACJ63260.1	FJ196385	sulfonamide resistant sul	sulfonamide antibiotic;sulfone antibiotic	antibiotic target replacement	1.53	86.60	3.32	15.46	2.95	323.55
Lizard04Caudovirales_VIRSorter_scaffold_2072-gene_3|2659|3366|707|-|-|-|-|-|FtsX|50.6|1e-14(-).p1	gb|AAV85982.1|ARO:3000535|macB	120	1	119	522	637	6.41E-08	27.5	ARO:3000535	36674	487	macB	AAV85982.1	AY768532	ATP-binding cassette (ABC) antibiotic efflux pump	macrolide antibiotic	antibiotic efflux	0.00	0.06	0.00	1.24	0.15	0.00
Lizard04Caudovirales_VIRSorter_scaffold_2307-gene_4|2439|3506|1067|+|-|-|-|-|HlyD|104.9|3.5e-31(+).p1	gb|BAA11236.1|ARO:3000206|emrK	301	49	346	9	296	1.18E-36	31.561	ARO:3000206	36345	609	emrK	BAA11236.1	D78168	major facilitator superfamily (MFS) antibiotic efflux pump	tetracycline antibiotic	antibiotic efflux	0.00	0.11	0.00	2.72	0.30	1.93
Lizard04Caudovirales_VIRSorter_scaffold_467-gene_10|5958|7853|1895|-|-|-|-|-|ACR_tran|273.9|1.4e-82(-).p1	gb|BAE06006.1|ARO:3003705|mexN	275	9	275	748	1019	1.52E-29	30.909	ARO:3003705	40359	2217	mexN	BAE06006.1	AB219523.1	resistance-nodulation-cell division (RND) antibiotic efflux pump	phenicol antibiotic	antibiotic efflux	10.22	753.71	125.13	295.46	153.01	583.00
Lizard04Caudovirales_VIRSorter_scaffold_467-gene_12|8659|9018|359|+|-|-|-|-|-|-|-|-(+).p3	gb|NP_252896.1|ARO:3000808|mexI	108	14	119	305	412	5.29E-17	41.667	ARO:3000808	37188	1647	MexI	AAG07594.1	AE004091.2	resistance-nodulation-cell division (RND) antibiotic efflux pump	acridine dye;fluoroquinolone antibiotic;tetracycline antibiotic	antibiotic efflux	1.62	132.19	19.99	43.20	22.57	88.33
Lizard04Caudovirales_VIRSorter_scaffold_12929-gene_4|2887|3771|884|-|-|-|-|-|LysR_substrate|135.0|1.3e-40(-).p1	gb|ALH22601.1|ARO:3000620|adeL	140	4	142	1	140	4.33E-08	25	ARO:3000620	36962	2654	adeL	ALH22601.1	KR297239.1	resistance-nodulation-cell division (RND) antibiotic efflux pump	fluoroquinolone antibiotic;tetracycline antibiotic	antibiotic efflux	0.46	115.43	1.22	7.15	0.00	29.60
Lizard04Caudovirales_VIRSorter_scaffold_6119-gene_2|2463|4730|2267|-|Phage_cluster_2466|159.9|4.3e-	gb|AAC75429.1|ARO:3000833|evgS	258	515	754	686	941	6.11E-32	32.946	ARO:3000833	37213	1318	evgS	AAC75429.1	U00096	major facilitator superfamily (MFS) antibiotic efflux pump;resistance-	fluoroquinolone antibiotic;macrolide antibiotic;penam;tetracycline antibiotic	antibiotic efflux	0.00	0.00	0.00	6.84	0.59	4.65
Lizard04Caudovirales_VIRSorter_scaffold_10848-gene_5|2463|3845|1382|+|-|-|-|-|OEP|205.9|2.9e-62(+).p1	gb|NP_249118.1|ARO:3000379|OprM	412	40	436	66	446	6.92E-16	22.33	ARO:3000379	36518	1305	OprM	AAG03816.1	AE004091.2	resistance-nodulation-cell division (RND) antibiotic efflux pump	acridinedye;aminocoumarin;aminoglycoside;carbapenem;cephalosporin;cephamycin;diaminopyrimidine;fluoroquinolone;macrolide;monobactam;penam;penem;peptide;phenicol;sulfonamide;tetracycli	antibiotic efflux	1.66	324.79	13.89	28.90	6.64	328.52
Lizard04Caudovirales_VIRSorter_scaffold_10848-gene_6|4012|5220|1208|+|-|-|-|-|HlyD_2|117.5|4.7e-35(+).p1	gb|AAV85981.1|ARO:3000533|macA	384	35	396	35	390	1.81E-29	29.948	ARO:3000533	36672	1680	macA	AAV85981.1	AY768531	ATP-binding cassette (ABC) antibiotic efflux pump	macrolide antibiotic	antibiotic efflux	2.73	309.41	13.89	30.69	8.26	359.73
Lizard04Caudovirales_VIRSorter_scaffold_10848-gene_7|5266|6009|743|+|Phage_cluster_405|145.1|1.8e-	gb|AAV85982.1|ARO:3000535|macB	229	4	231	3	230	2.52E-62	45.852	ARO:3000535	36674	487	macB	AAV85982.1	AY768532	ATP-binding cassette (ABC) antibiotic efflux pump	macrolide antibiotic	antibiotic efflux	1.57	176.78	7.32	22.69	4.43	193.58
Lizard04Caudovirales_VIRSorter_scaffold_10848-gene_8|6108|7328|1220|+|Phage_cluster_6774|45.5|2.3e-	gb|AAV85982.1|ARO:3000535|macB	398	7	403	255	641	1.36E-90	39.447	ARO:3000535	36674	487	macB	AAV85982.1	AY768532	ATP-binding cassette (ABC) antibiotic efflux pump	macrolide antibiotic	antibiotic efflux	6.71	589.33	28.86	45.99	19.33	638.11
Lizard04Caudovirales_VIRSorter_scaffold_3559-gene_6|5289|5873|584|+|Phage_cluster_3242|52.3|3.5e-	gb|NP_460903.1|ARO:3000826|sdiA	61	127	187	180	240	2.50E-06	37.705	ARO:3000826	37206	228	sdiA	AAL20862.1	AE006468.2	resistance-nodulation-cell division (RND) antibiotic efflux pump	cephalosporin;fluoroquinolone antibiotic;glycylcycline;penam;phenicol antibiotic;rifamycin antibiotic;tetracycline antibiotic;triclosan	antibiotic efflux	0.09	96.19	1.08	10.72	3.84	24.23
Lizard04Caudovirales_VIRSorter_scaffold_7434-gene_2|200|709|509|+|Phage_cluster_1277|124.2|2.9e-	gb|CCP46182.1|ARO:3003035|mfpA	57	47	101	98	154	1.77E-06	45.614	ARO:3003035	39469	801	mfpA	CCP46182.1	AL123456	quinolone resistance protein (qnr)	fluoroquinolone antibiotic	antibiotic target protection	0.00	0.00	0.00	2.25	0.00	0.00
Lizard04unclassified_bacterial_viruses_VIRSorter_scaffold_5626-gene_1|3|482|479|+|-|-|-|-|Pfam-B_8079|54.9|3.6e-17(+).p1	gb|ABF66011.1|ARO:3002881|lmrC	103	40	141	459	556	8.88E-08	33.981	ARO:3002881	39315	974	lmrC	ABX00624.1	EU124663.1	ABC-F ATP-binding cassette ribosomal protection protein	lincosamide antibiotic;macrolide antibiotic;oxazolidinone antibiotic;phenicol antibiotic;pleuromutilin antibiotic;streptogramin antibiotic;tetracycline antibiotic	antibiotic target protection	0.00	0.00	0.00	0.16	0.59	13.40
Lizard04unclassified_dsDNA_phages_VIRSorter_scaffold_6680-gene_5|4955|5974|1019|+|-|-|-|-|HlyD|95.1|5.3e-30(+).p1	gb|ABS43901.1|ARO:3000783|cmeA	327	14	336	45	362	1.25E-42	31.193	ARO:3000783	37163	1181	cmeA	ABS43901.1	CP000768	resistance-nodulation-cell division (RND) antibiotic efflux pump	cephalosporin;fluoroquinolone antibiotic;fusidic acid;macrolide antibiotic	antibiotic efflux	5.32	273.08	25.88	14.92	11.07	215.89
Lizard04unclassified_dsDNA_phages_VIRSorter_scaffold_6680-gene_6|5987|9166|3179|+|-|-|-|-|ACR_tran|977.4|7.2e-297(+).p1	gb|ABS43151.1|ARO:3000784|cmeB	1048	4	1040	4	1037	0	38.836	ARO:3000784	37164	748	cmeB	ABS43151.1	CP000768	resistance-nodulation-cell division (RND) antibiotic efflux pump	cephalosporin;fluoroquinolone antibiotic;fusidic acid;macrolide antibiotic	antibiotic efflux	12.58	619.76	53.59	25.79	29.80	612.84
Lizard04unclassified_dsDNA_phages_VIRSorter_scaffold_6680-gene_7|9212|10564|1352|+|-|-|-|-|OEP|172.8|6.8e-54(+).p1	gb|CAA64891.1|ARO:3000812|mtrE	442	1	438	18	458	7.46E-51	28.507	ARO:3000812	37192	453	mtrE	CAA64891.1	X95635	resistance-nodulation-cell division (RND) antibiotic efflux pump	macrolide antibiotic;penam	antibiotic efflux	2.77	165.54	11.04	4.12	10.03	167.27

Query ORF ID	Subject ARG ID	Alignment length	query start	query end	subject start	subject end	e-value	Identity(%)	ARO Accession	CVTERM ID	Model ID	ARO Name	Protein Accession	DNA Accession	AMR Gene Family	Drug Class	Resistance Mechanism	Abundance (ppm)					
																		(1) PE-NMC	(2) PE-NQ1	(3) PE-NQ2	(4) PT-SZZ	(5) PT-ZB	(6) PT-RT
Lizard04unclassified_dsDNA_phages_VIRSorter_scaffold_1413-gene_2|1905|3278|1373|-|-|-|-|-|OEP|173.5|3.9e-54(-).p1	gb|CAA64891.1|ARO:3000812|mtrE	456	6	453	9	458	1.40E-50	29.825	ARO:3000812	37192	453	mtrE	CAA64891.1	X95635	resistance-nodulation-cell division (RND) antibiotic efflux pump	macrolide antibiotic;penam	antibiotic efflux	1.25	162.23	2.37	13.83	1.77	135.42
Lizard04unclassified_dsDNA_phages_VIRSorter_scaffold_1413-gene_3|3268|6495|3227|-|-|-|-|-|ACR_tran|973.9|7.9e-296(-).p1	gb|ABS43151.1|ARO:3000784|cmeB	1038	3	1032	4	1029	0	38.15	ARO:3000784	37164	748	cmeB	ABS43151.1	CP000768	resistance-nodulation-cell division (RND) antibiotic efflux pump	cephalosporin;fluoroquinolone antibiotic;fusidic acid;macrolide antibiotic	antibiotic efflux	1.02	350.87	2.98	24.47	8.41	359.89
Lizard04unclassified_dsDNA_phages_VIRSorter_scaffold_1413-gene_4|6508|7182|674|-|-|-|-|-|HlyD_3|41.2|1.9e-13(-).p1	gb|YP_002320475.1|ARO:3000780|adeI	211	15	222	172	381	5.49E-26	32.701	ARO:3000780	37160	1471	adeI	ACJ41739.1	CP001182.2	resistance-nodulation-cell division (RND) antibiotic efflux pump	carbapenem;cephalosporin;diaminopyrimidine antibiotic;fluoroquinolone antibiotic;lincosamide antibiotic;macrolide antibiotic;penem;phenicol antibiotic;rifamycin antibiotic;tetracycline antibiotic	antibiotic efflux	0.00	29.77	0.47	0.70	0.15	28.32
Lizard04unclassified_dsDNA_phages_VIRSorter_scaffold_244-gene_1|1|1044|1043|+|-|-|-|-|OEP|182.5|6.9e-57(+).p1	gb|BAO79432.1|ARO:3000785|cmeC	366	3	343	126	466	4.44E-12	24.59	ARO:3000785	37165	798	cmeC	BAO79432.1	AB894099	resistance-nodulation-cell division (RND) antibiotic efflux pump	cephalosporin;fluoroquinolone antibiotic;fusidic acid;macrolide antibiotic	antibiotic efflux	0.00	3.80	0.00	2.10	1.48	0.80
Lizard04unclassified_dsDNA_phages_VIRSorter_scaffold_244-gene_2|1102|1917|815|+|-|-|-|-|HlyD_2|67.2|1.6e-21(+).p1	gb|AAG07762.1|ARO:3003030|mexV	145	79	216	88	232	1.57E-13	30.345	ARO:3003030	39464	2207	MexV	AAG07762.1	AE004091.2	resistance-nodulation-cell division (RND) antibiotic efflux pump	acridine dye;fluoroquinolone antibiotic;macrolide antibiotic;phenicol antibiotic;tetracycline antibiotic	antibiotic efflux	0.00	10.03	0.07	6.06	0.15	1.52
Lizard04unclassified_dsDNA_phages_VIRSorter_scaffold_244-gene_3|1930|5055|3125|+|-|-|-|-|ACR_tran|853.5|2e-259(+).p1	gb|AAC75137.1|ARO:3000794|mdtC	1048	1	1038	1	1021	1.29E-129	27.672	ARO:3000794	37174	1315	mdtC	AAC75137.1	U00096	resistance-nodulation-cell division (RND) antibiotic efflux pump	aminocoumarin antibiotic	antibiotic efflux	0.00	193.15	0.00	135.49	18.44	28.40
Lizard05Caudovirales_VIRSorter_scaffold_6289-gene_1|3|1070|1067|+|Phage_cluster_398|134.9|6.6e-	gb|NP_252244|ARO:3002985|arnA	379	8	344	299	649	3.26E-14	25.33	ARO:3002985	39419	1643	arnA	AAG06942.1	AE004091.2	pmr phosphoethanolamine transferase	peptide antibiotic	antibiotic target alteration	0.00	0.00	0.00	23.15	3.98	53.27
Lizard05Caudovirales_VIRSorter_scaffold_6289-gene_2|1064|2323|1259|+|Phage_cluster_3799|141.9|4.6e-	gb|AAC75089.1|ARO:3003577|pmrE	370	1	366	1	342	5.43E-32	28.108	ARO:3003577	40187	842	ugd	AAC75089.1	U00096	pmr phosphoethanolamine transferase	peptide antibiotic	antibiotic target alteration	0.00	0.00	0.00	76.37	10.62	165.26
Lizard05Caudovirales_VIRSorter_scaffold_475-gene_5|2785|3783|998|+|Phage_cluster_559|247.2|2.9e-	gb|AAD01867.1|ARO:3002875|dfrE	109	210	317	4	104	1.39E-08	27.523	ARO:3002875	39309	1544	dfrE	EOD99669.1	AIIS01000002.1	trimethoprim resistant dihydrofolate reductase dfr	diaminopyrimidine antibiotic	antibiotic target replacement	0.00	0.00	0.00	113.04	5.46	0.00
Lizard05Caudovirales_VIRSorter_scaffold_17667-gene_4|1619|2575|956|+|Phage_cluster_54|177.8|6.2e-	gb|AAD01867.1|ARO:3002875|dfrE	154	165	318	4	153	2.85E-23	33.117	ARO:3002875	39309	1544	dfrE	EOD99669.1	AIIS01000002.1	trimethoprim resistant dihydrofolate reductase dfr	diaminopyrimidine antibiotic	antibiotic target replacement	0.00	0.00	0.00	0.00	3.98	5.94
Lizard05Caudovirales_VIRSorter_scaffold_155-gene_7|4649|5071|422|-|-|-|-|-|HlyD_3|56.7|6.3e-17(-).p3	gb|AAG07762.1|ARO:3003030|mexV	106	31	136	168	273	2.68E-11	25.472	ARO:3003030	39464	2207	MexV	AAG07762.1	AE004091.2	resistance-nodulation-cell division (RND) antibiotic efflux pump	acridine dye;fluoroquinolone antibiotic;macrolide antibiotic;phenicol antibiotic;tetracycline antibiotic	antibiotic efflux	1.11	110.41	20.66	46.38	23.61	67.95
Lizard05Caudovirales_VIRSorter_scaffold_1211-gene_5|776|2163|1387|+|Phage_cluster_38|230.7|5.3e-	gb|NP_415632.1|ARO:3003844|mfd	200	213	393	762	954	2.09E-06	24	ARO:3003844	40538	2332	mfd	NP_415632.1	NC_000913.3	quinolone resistance protein (qnr)	fluoroquinolone antibiotic	antibiotic target protection	0.00	0.00	0.00	0.16	3.69	12.60
Lizard05unclassified_dsDNA_phages_VIRSorter_scaffold_5475-gene_5|3681|4658|977|-|-|-|-|-|ACR_tran|286.1|2e-88(-).p1	gb|AAC75137.1|ARO:3000794|mdtC	333	6	324	649	980	7.02E-41	27.027	ARO:3000794	37174	1315	mdtC	AAC75137.1	U00096	resistance-nodulation-cell division (RND) antibiotic efflux pump	aminocoumarin antibiotic	antibiotic efflux	0.14	0.28	0.07	4.04	6.34	263.06
Lizard05unclassified_dsDNA_phages_VIRSorter_scaffold_6921-gene_3|390|836|446|-|-|-|-|-|ACR_tran|97.3|2.2e-31(-).p1	gb|AAG07763.1|ARO:3003031|mexW	136	16	146	817	951	5.23E-18	33.824	ARO:3003031	39465	2208	MexW	AAG07763.1	NC_002516.2	resistance-nodulation-cell division (RND) antibiotic efflux pump	acridine dye;fluoroquinolone antibiotic;macrolide antibiotic;phenicol antibiotic;tetracycline antibiotic	antibiotic efflux	3.33	270.99	28.05	15.77	7.08	31.21
Lizard06Bicaudaviridae_VIRSorter_scaffold_1876-gene_3|1037|1789|752|-|Phage_cluster_3811|43.0|6.2e-	gb|AAC75314.1|ARO:3003578|pmrF	215	7	217	12	211	1.71E-16	29.302	ARO:3003578	40188	2014	PmrF	AAC75314.1	U00096	pmr phosphoethanolamine transferase	peptide antibiotic	antibiotic target alteration	0.00	14.77	0.00	0.39	0.30	4.97
Lizard06Bicaudaviridae_VIRSorter_scaffold_1876-gene_4|1905|2605|700|+|-|-|-|-|Pfam-B_9784|84.9|1.1e-26(+).p1	gb|NP_415632.1|ARO:3003844|mfd	198	34	229	20	213	6.27E-23	28.788	ARO:3003844	40538	2332	mfd	NP_415632.1	NC_000913.3	quinolone resistance protein (qnr)	fluoroquinolone antibiotic	antibiotic target protection	0.09	17.09	0.00	0.54	0.00	6.66
Lizard06Caudovirales_VIRSorter_scaffold_40011-gene_28|23936|24988|1052|-|Phage_cluster_8947|297.0|2.2e-	gb|AJP77057|ARO:3003718|MSI-1	182	11	156	93	263	5.44E-06	27.473	ARO:3003718	40372	2233	MSI-1	AJP77057	KP109676	MSI beta-lactamase	carbapenem	antibiotic inactivation	0.00	0.06	0.47	0.00	0.00	3.13
Lizard06Caudovirales_VIRSorter_scaffold_20281-gene_2|1192|2694|1502|-|-|-|-|-|GTP_EFTU|134.4|6e-40(-).p2	gb|CAD55718.1|ARO:3000197|tet36	442	1	440	20	422	1.48E-38	29.186	ARO:3000197	36336	1708	tet36	CAD55718.1	AJ514254	tetracycline-resistant ribosomal protection protein	tetracycline antibiotic	antibiotic target protection	0.00	0.28	0.00	132.38	28.33	152.27
Lizard06Caudovirales_VIRSorter_scaffold_5080-gene_11|13944|14666|722|-|Phage_cluster_405|128.5|5.1e-	gb|APB03214.1|ARO:3003980|tetA(48)	220	1	219	18	235	6.81E-37	35.455	ARO:3003980	40861	2517	tetA(58)	APB03214.1	KX531044.1	major facilitator superfamily (MFS) antibiotic efflux pump	tetracycline antibiotic	antibiotic efflux	0.00	0.11	0.00	0.00	0.89	31.29
Lizard06Caudovirales_VIRSorter_scaffold_5080-gene_13|16605|18113|1508|-|-|-|-|-|OEP|118.0|6.9e-35(-).p1	gb|BAO79432.1|ARO:3000785|cmeC	342	151	482	157	470	1.27E-06	23.977	ARO:3000785	37165	798	cmeC	BAO79432.1	AB894099	resistance-nodulation-cell division (RND) antibiotic efflux pump	cephalosporin;fluoroquinolone antibiotic;fusidic acid;macrolide antibiotic	antibiotic efflux	0.00	0.33	0.00	0.00	0.00	12.11
Lizard06Caudovirales_VIRSorter_scaffold_107610-gene_13|14067|15293|1226|-|Phage_cluster_8323|62.4|7.9e-	gb|AAC75314.1|ARO:3003578|pmrF	124	33	150	10	131	1.36E-09	29.032	ARO:3003578	40188	2014	PmrF	AAC75314.1	U00096	pmr phosphoethanolamine transferase	peptide antibiotic	antibiotic target alteration	0.00	0.00	0.00	0.00	0.00	5.38
Lizard06Caudovirales_VIRSorter_scaffold_48869-gene_6|4335|5483|1148|+|Phage_cluster_559|269.2|4.2e-	gb|AAD01867.1|ARO:3002875|dfrE	82	288	369	34	110	1.20E-07	26.829	ARO:3002875	39309	1544	dfrE	EOD99669.1	AIIS01000002.1	trimethoprim resistant dihydrofolate reductase dfr	diaminopyrimidine antibiotic	antibiotic target replacement	0.00	0.00	0.00	0.00	0.00	4.81
Lizard06Caudovirales_VIRSorter_scaffold_15095-gene_8|8269|9363|1094|+|Phage_cluster_398|135.1|4.2e-	gb|NP_252244|ARO:3002985|arnA	379	17	353	299	649	3.38E-13	25.066	ARO:3002985	39419	1643	arnA	AAG06942.1	AE004091.2	pmr phosphoethanolamine transferase	peptide antibiotic	antibiotic target alteration	0.00	0.00	0.00	23.54	3.98	54.31
Lizard06Caudovirales_VIRSorter_scaffold_15095-gene_9|9357|10616|1259|+|Phage_cluster_3799|139.7|1.7e-	gb|AAC75089.1|ARO:3003577|pmrE	393	1	387	1	365	7.95E-34	28.244	ARO:3003577	40187	842	ugd	AAC75089.1	U00096	pmr phosphoethanolamine transferase	peptide antibiotic	antibiotic target alteration	0.00	0.00	0.00	75.05	10.62	168.07
Lizard06Caudovirales_VIRSorter_scaffold_23275-gene_6|8520|11837|3317|-|Phage_cluster_7519|63.3|1.9e-	gb|BAD59497.1|ARO:3000501|Nocardia	1108	22	1079	40	1142	0	55.596	ARO:3000501	36640	2831	rpoB2	BAD59497.1	AP006618.1	rifamycin-resistant beta-subunit of RNA polymerase (rpoB)	peptide antibiotic;rifamycin antibiotic	antibiotic target alteration;antibiotic	0.14	0.00	0.88	0.08	0.00	20.46
Lizard06Caudovirales_VIRSorter_scaffold_33331-gene_1|54|740|686|+|Phage_cluster_405|175.7|2.6e-	gb|AAV85982.1|ARO:3000535|macB	228	1	228	3	230	1.15E-56	39.035	ARO:3000535	36674	487	macB	AAV85982.1	AY768532	ATP-binding cassette (ABC) antibiotic efflux pump	macrolide antibiotic	antibiotic efflux	0.00	0.11	0.00	0.00	0.44	101.32
Lizard06Caudovirales_VIRSorter_scaffold_33331-gene_3|3471|5789|2318|+|-|-|-|-|FtsX|95.7|2.9e-28(+).p1	gb|AAV85982.1|ARO:3000535|macB	274	97	358	341	601	3.24E-06	20.073	ARO:3000535	36674	487	macB	AAV85982.1	AY768532	ATP-binding cassette (ABC) antibiotic efflux pump	macrolide antibiotic	antibiotic efflux	0.00	1.76	0.61	0.62	1.62	833.30
Lizard06Caudovirales_VIRSorter_scaffold_33331-gene_6|7514|8860|1346|-|Phage_cluster_3804|329.0|7.2e-	gb|CAE00499.1|ARO:3003835|cdeA	422	15	423	14	421	9.78E-53	30.806	ARO:3003835	40526	2321	cdeA	CAE00499.1	AJ574887.1	multidrug and toxic compound extrusion (MATE) transporter	acridine dye;fluoroquinolone antibiotic	antibiotic efflux	0.00	0.55	0.07	0.16	1.33	217.49
Lizard06Caudovirales_VIRSorter_scaffold_16643-gene_6|5068|6312|1244|-|-|-|-|-|OEP|187.2|4.3e-56(-).p1	gb|BAO79432.1|ARO:3000785|cmeC	277	151	411	204	468	1.21E-11	24.188	ARO:3000785	37165	798	cmeC	BAO79432.1	AB894099	resistance-nodulation-cell division (RND) antibiotic efflux pump	cephalosporin;fluoroquinolone antibiotic;fusidic acid;macrolide antibiotic	antibiotic efflux	0.00	0.00	0.00	0.00	0.00	27.60
Lizard06Caudovirales_VIRSorter_scaffold_16643-gene_7|6371|9436|3065|-|-|-|-|-|ACR_tran|523.5|1.5e-157(-).p1	gb|AAG07064.1|ARO:3003693|mexK	1020	1	1009	4	1019	4.53E-157	29.51	ARO:3003693	40341	2206	MexK	AAG07064.1	AE004091.2	resistance-nodulation-cell division (RND) antibiotic efflux pump	macrolide antibiotic;tetracycline antibiotic;triclosan	antibiotic efflux	0.00	0.00	0.00	1.09	0.00	60.97
Lizard06Caudovirales_VIRSorter_scaffold_16643-gene_8|9449|10462|1013|-|-|-|-|-|HlyD_2|95.9|5e-28(-).p2	gb|NP_252367.1|ARO:3003692|mexJ	328	21	331	36	351	6.63E-29	26.829	ARO:3003692	40340	2205	MexJ	AAG07065.1	AE004091.2	resistance-nodulation-cell division (RND) antibiotic efflux pump	macrolide antibiotic;tetracycline antibiotic;triclosan	antibiotic efflux	0.00	0.00	0.00	0.23	0.00	9.47
Lizard06Caudovirales_VIRSorter_scaffold_102358-gene_2|453|1409|956|+|Phage_cluster_7121|63.2|2.9e-	gb|AAC75315.1|ARO:3002985|arnA	295	1	292	1	279	1.89E-25	27.797	ARO:3002985	39419	1643	arnA	AAG06942.1	AE004091.2	pmr phosphoethanolamine transferase	peptide antibiotic	antibiotic target alteration	0.00	0.00	0.00	0.00	0.00	2.25
Lizard06Caudovirales_VIRSorter_scaffold_102358-gene_5|5817|6470|653|-|-|-|-|-|Lactamase_B|89.1|5.4e-26(-).p1	gb|BAL14456.1|ARO:3000854|SMB-1	153	2	149	41	179	8.17E-09	25.49	ARO:3000854	37234	753	SMB-1	BAL14456.1	AB636283	SMB beta-lactamase	carbapenem;cephalosporin;penam	antibiotic inactivation	0.00	0.00	0.00	0.00	0.00	0.64
Lizard06Caudovirales_VIRSorter_scaffold_102358-gene_9|9622|11214|1592|+|Phage_cluster_2466|83.7|1.6e-	gb|YP_499945.1|ARO:3000839|arlS	419	119	524	105	451	1.62E-14	24.821	ARO:3000839	37219	182	arlS	ABD30512.1	CP000253.1	major facilitator superfamily (MFS) antibiotic efflux pump	acridine dye;fluoroquinolone antibiotic	antibiotic efflux	0.00	0.00	0.00	0.00	0.00	8.74
Lizard06Caudovirales_VIRSorter_scaffold_102358-gene_10|11261|12268|1007|+|Phage_cluster_3079|60.0|3.5e-	gb|ACL82957.1|ARO:3002928|vanRM	122	1	119	1	118	7.31E-06	29.508	ARO:3002928	39362	752	vanRM	ACL82957.1	FJ349556	glycopeptide resistance gene cluster;vanR	glycopeptide antibiotic	antibiotic target alteration	0.00	0.00	0.00	0.00	0.00	3.93
Lizard06Caudovirales_VIRSorter_scaffold_86473-gene_9|8431|11256|2825|-|-|-|-|-|tRNA-synt_1|666.8|4e-201(-).p1	gb|YP_003971446|ARO:3003730|Bifidobacteria	887	40	845	47	890	3.11E-71	26.607	ARO:3003730	40419	2278	ileS	ADP36409.1	CP001840.1	antibiotic-resistant isoleucyl-tRNA synthetase (ileS)	mupirocin	antibiotic target alteration	0.00	0.00	0.00	0.00	0.00	16.29
Lizard06Caudovirales_VIRSorter_scaffold_39861-gene_7|8883|9842|959|-|Phage_cluster_405|123.5|1.7e-	gb|AAV85982.1|ARO:3000535|macB	248	3	248	3	228	8.53E-25	31.452	ARO:3000535	36674	487	macB	AAV85982.1	AY768532	ATP-binding cassette (ABC) antibiotic efflux pump	macrolide antibiotic	antibiotic efflux	0.00	0.00	0.00	0.00	0.00	4.25
Lizard06Caudovirales_VIRSorter_scaffold_30160-gene_1|1|1143|1142|-|-|-|-|-|ABC_membrane|105.0|8.9e-31(-).p1	gb|CDO61513.1|ARO:3003948|efrA	189	62	250	1	189	1.08E-24	30.688	ARO:3003948	40703	2421	efrA	WP_104671188.1	NZ_PTWF01000025.1	ATP-binding cassette (ABC) antibiotic efflux pump	fluoroquinolone antibiotic;macrolide antibiotic;rifamycin antibiotic	antibiotic efflux	0.00	42.94	0.00	6.22	0.30	1.93
Lizard06Caudovirales_VIRSorter_scaffold_31230-gene_2|623|1642|1019|+|-|-|-|-|HlyD_2|100.1|2.5e-29(+).p1	gb|NP_248847.1|ARO:3003680|TriB	340	1	324	14	349	4.67E-26	27.647	ARO:3003680	40315	2193	TriB	AAG03547.1	AE004091.2	resistance-nodulation-cell division (RND) antibiotic efflux pump	triclosan	antibiotic efflux	0.00	0.00	0.00	0.00	0.00	2.73
Lizard06Caudovirales_VIRSorter_scaffold_31230-gene_3|1679|4852|3173|+|-|-|-|-|ACR_tran|505.2|5.2e-152(+).p1	gb|AAG07064.1|ARO:3003693|mexK	1055	1	1042	3	1022	1.54E-153	29.668	ARO:3003693	40341	2206	MexK	AAG07064.1	AE004091.2	resistance-nodulation-cell division (RND) antibiotic efflux pump	macrolide antibiotic;tetracycline antibiotic;triclosan	antibiotic efflux	0.00	0.00	0.00	0.00	0.89	27.52
Lizard06Caudovirales_VIRSorter_scaffold_31230-gene_5|6073|7398|1325|+|-|-|-|-|OEP|209.9|4.6e-63(+).p1	gb|BAO79432.1|ARO:3000785|cmeC	352	109	438	133	466	1.22E-12	22.443	ARO:3000785	37165	798	cmeC	BAO79432.1	AB894099	resistance-nodulation-cell division (RND) antibiotic efflux pump	cephalosporin;fluoroquinolone antibiotic;fusidic acid;macrolide antibiotic	antibiotic efflux	0.00	0.00	0.00	0.00	0.00	27.76
Lizard06Caudovirales_VIRSorter_scaffold_71252-gene_7|6573|8186|1613|+|Phage_cluster_11678|241.9|8.9e-	gb|APB03219.1|ARO:3003986|TaeA	521	1	513	3	516	3.98E-71	29.175	ARO:3003986	40868	2522	TaeA	APB03219.1	KX531049.1	ATP-binding cassette (ABC) antibiotic efflux pump	pleuromutilin antibiotic	antibiotic efflux	0.00	0.00	0.00	0.70	0.00	8.34
Lizard06Caudovirales_VIRSorter_scaffold_50099-gene_8|5476|6780|1304|+|Phage_cluster_15111|58.9|7e-	gb|NP_417544.5|ARO:3000024|patA	352	5	338	26	355	8.81E-41	33.523	ARO:3000024	36033	2081	patA	AAK76137.1	AE005672.3	ATP-binding cassette (ABC) antibiotic efflux pump	fluoroquinolone antibiotic	antibiotic efflux	0.00	0.00	0.00	1.09	0.59	3.13
Lizard06Caudovirales_VIRSorter_scaffold_30650-gene_2|1313|2221|908|-|Phage_cluster_8887|64.5|1.4e-	gb|YP_491919.1|ARO:3000508|gadX	81	212	292	163	242	8.69E-06	24.691	ARO:3000508	36647	91	gadX	BAE77778.1	AP009048.1	resistance-nodulation-cell division (RND) antibiotic efflux pump	fluoroquinolone antibiotic;macrolide antibiotic;penam	antibiotic efflux	0.00	0.22	0.00	0.85	0.00	2.49
Lizard06Caudovirales_VIRSorter_scaffold_922-gene_2|661|2424|1763|-|Phage_cluster_2466|162.3|2.2e-	gb|BAA15934.1|ARO:3000829|baeS	236	348	581	228	457	1.64E-25	26.695	ARO:3000829	37209	986	baeS	BAA15934.1	AP009048	resistance-nodulation-cell division (RND) antibiotic efflux pump	aminocoumarin antibiotic;aminoglycoside antibiotic	antibiotic efflux	0.00	0.00	0.00	0.00	0.89	275.57
Lizard06Caudovirales_VIRSorter_scaffold_922-gene_4|3189|3959|770|-|Phage_cluster_405|164.9|5.1e-	gb|AAA99504.1|ARO:3002987|bcrA	239	21	254	17	232	1.74E-25	31.799	ARO:3002987	39421	345	bcrA	AAA99504.1	L20573	ATP-binding cassette (ABC) antibiotic efflux pump	peptide antibiotic	antibiotic efflux	0.00	0.00	0.00	0.00	0.00	44.53
Lizard06Caudovirales_VIRSorter_scaffold_100206-gene_5|3218|3691|473|-|Phage_cluster_3079|59.4|5.1e-	gb|ADM92605.1|ARO:3000553|adeR	107	39	142	15	118	3.62E-07	28.037	ARO:3000553	36692	456	adeR	ADM92605.1	HM440348	resistance-nodulation-cell division (RND) antibiotic efflux pump	glycylcycline;tetracycline antibiotic	antibiotic efflux	0.00	0.00	0.00	0.00	0.00	1.60
Lizard06Caudovirales_VIRSorter_scaffold_43393-gene_6|4256|5413|1157|+|-|-|-|-|Ala_racemase_N|198.4|2e-59(+).p1	gb|ABA71733.1|ARO:3002972|vanTG	346	24	355	345	682	2.53E-55	34.682	ARO:3002972	39406	1561	vanTG	ABA71733.1	DQ212986	glycopeptide resistance gene cluster;vanT	glycopeptide antibiotic	antibiotic target alteration	0.00	0.00	0.00	0.00	0.00	5.13
Lizard06Caudovirales_VIRSorter_scaffold_62201-gene_4|4284|5417|1133|-|-|-|-|-|EPSP_synthase|265.2|1.4e-79(-).p1	gb|CCE36834|ARO:3003784|Mycobacterium	356	2	349	64	415	1.23E-103	47.191	ARO:3003784	40467	2290	murA	CCE36834	HE608151	antibiotic-resistant murA transferase	fosfomycin	antibiotic target alteration	0.00	0.00	0.00	0.00	0.00	7.06
Lizard06Caudovirales_VIRSorter_scaffold_21507-gene_1|382|1296|914|-|Phage_cluster_8887|45.0|1.2e-	gb|CAE51745.1|ARO:3000168|tet(D)	78	221	298	50	127	7.86E-08	37.179	ARO:3000168	36307	1138	tet(D)	AAL75563.1	AF467077.1	major facilitator superfamily (MFS) antibiotic efflux pump	tetracycline antibiotic	antibiotic efflux	0.00	0.50	0.00	0.00	0.00	3.61
Lizard06Caudovirales_VIRSorter_scaffold_21507-gene_3|2696|3322|626|+|-|-|-|-|Hexapep_2|65.1|5.7e-19(+).p1	gb|ACX92987.1|ARO:3002845|vatH	117	72	185	58	170	6.05E-17	41.026	ARO:3002845	39279	837	vatH	ACX92987.1	GQ205627	streptogramin vat acetyltransferase	streptogramin antibiotic	antibiotic inactivation	0.00	0.99	0.00	0.00	0.00	3.05
Lizard06Caudovirales_VIRSorter_scaffold_43851-gene_4|1826|2773|947|-|Phage_cluster_3811|128.8|3.8e-	gb|AAC75314.1|ARO:3003578|pmrF	317	2	314	10	314	1.35E-52	34.069	ARO:3003578	40188	2014	PmrF	AAC75314.1	U00096	pmr phosphoethanolamine transferase	peptide antibiotic	antibiotic target alteration	0.00	0.00	0.00	0.00	0.00	1.12
Lizard06Caudovirales_VIRSorter_scaffold_35676-gene_1|191|1792|1601|+|Phage_cluster_2466|126.6|1.5e-	gb|BAA15934.1|ARO:3000829|baeS	199	357	514	244	441	1.93E-18	31.156	ARO:3000829	37209	986	baeS	BAA15934.1	AP009048	resistance-nodulation-cell division (RND) antibiotic efflux pump	aminocoumarin antibiotic;aminoglycoside antibiotic	antibiotic efflux	0.00	17.42	0.00	1.09	0.00	53.75
Lizard06Caudovirales_VIRSorter_scaffold_16333-gene_3|954|1910|956|-|Phage_cluster_54|175.7|1.9e-	gb|AAD01867.1|ARO:3002875|dfrE	154	164	317	4	153	2.52E-23	33.117	ARO:3002875	39309	1544	dfrE	EOD99669.1	AIIS01000002.1	trimethoprim resistant dihydrofolate reductase dfr	diaminopyrimidine antibiotic	antibiotic target replacement	0.00	0.00	0.00	0.00	3.98	6.34
Lizard06Caudovirales_VIRSorter_scaffold_62577-gene_2|495|1445|950|+|Phage_cluster_3811|130.4|1.2e-	gb|AAC75314.1|ARO:3003578|pmrF	318	3	316	10	314	2.08E-46	32.075	ARO:3003578	40188	2014	PmrF	AAC75314.1	U00096	pmr phosphoethanolamine transferase	peptide antibiotic	antibiotic target alteration	0.00	0.00	0.00	0.16	0.00	2.89

Query ORF ID	Subject ARG ID	Alignment length	query start	query end	subject start	subject end	e-value	Identity(%)	ARO Accession	CVTERM ID	Model ID	ARO Name	Protein Accession	DNA Accession	AMR Gene Family	Drug Class	Resistance Mechanism	Abundance (ppm)					
																		(1) PE-NMC	(2) PE-NQ1	(3) PE-NQ2	(4) PT-SZZ	(5) PT-ZB	(6) PT-RT
Lizard06Caudovirales_VIRSorter_scaffold_28653-gene_4|2659|4041|1382|+|-|-|-|-|OEP|205.7|9.2e-62(+).p1	gb|NP_249118.1|ARO:3000379|OprM	413	40	436	66	446	4.59E-16	22.76	ARO:3000379	36518	1305	OprM	AAG03816.1	AE004091.2	resistance-nodulation-cell division (RND) antibiotic efflux pump	acridinedye;aminocoumarin;aminoglycoside;carbapenem;cephalosporin;cephamycin;diaminopyrimidine;fluoroquinolone;macrolide;monobactam;penam;penem;peptide;phenicol;sulfonamide;tetracycli	antibiotic efflux	1.66	325.07	13.89	28.12	6.64	331.97
Lizard06Caudovirales_VIRSorter_scaffold_7340-gene_1|36|1358|1322|+|Phage_cluster_3804|115.4|4.8e-	gb|WP_014550864.1|ARO:3003953|hmrM	303	6	306	14	315	1.37E-24	24.422	ARO:3003953	40723	2425	hmrM	ADO96486.1	CP002276.1	multidrug and toxic compound extrusion (MATE) transporter	acridine dye;fluoroquinolone antibiotic	antibiotic efflux	0.28	10.03	2.44	0.00	0.00	5.46
Lizard06Caudovirales_VIRSorter_scaffold_17344-gene_3|1181|2314|1133|+|-|-|-|-|Pfam-B_9039|168.9|3.3e-50(+).p1	gb|YP_003971446|ARO:3003730|Bifidobacteria	163	4	143	27	188	3.51E-07	26.38	ARO:3003730	40419	2278	ileS	ADP36409.1	CP001840.1	antibiotic-resistant isoleucyl-tRNA synthetase (ileS)	mupirocin	antibiotic target alteration	0.00	0.00	0.00	15.38	2.07	182.11
Lizard06Caudovirales_VIRSorter_scaffold_106651-gene_1|1|487|486|-|-|-|-|-|MFS_1|51.6|1.1e-14(-).p1	gb|YP_489321.1|ARO:3001329|mdtG	107	9	109	3	109	3.74E-07	27.103	ARO:3001329	37728	603	mdtG	ABV18113.1	CP000800.1	major facilitator superfamily (MFS) antibiotic efflux pump	fosfomycin	antibiotic efflux	0.00	0.00	0.00	0.00	0.00	1.12
Lizard06Caudovirales_VIRSorter_scaffold_83693-gene_5|2503|3489|986|-|Phage_cluster_398|353.5|1.4e-	gb|AAC75315.1|ARO:3002985|arnA	347	1	314	317	654	6.34E-11	24.207	ARO:3002985	39419	1643	arnA	AAG06942.1	AE004091.2	pmr phosphoethanolamine transferase	peptide antibiotic	antibiotic target alteration	0.00	0.00	0.00	0.00	0.00	2.49
Lizard06Caudovirales_VIRSorter_scaffold_106486-gene_1|63|815|752|+|-|-|-|-|SpoU_methylase|130.4|8.5e-39(+).p1	gb|CCP44409.1|ARO:3003060|tsnr	163	82	241	99	255	2.11E-11	26.994	ARO:3003060	39494	49	tsnR	AAA99931.1	L39157.1	non-erm 23S ribosomal RNA methyltransferase (A1067)	peptide antibiotic	antibiotic target alteration	0.00	0.06	0.00	0.00	0.00	0.72
Lizard06Caudovirales_VIRSorter_scaffold_11010-gene_4|1484|2743|1259|-|Phage_cluster_244|181.3|4.6e-	gb|AAO47226.2|ARO:3003318|Streptomyces	178	1	172	453	627	1.88E-47	48.876	ARO:3003318	39902	2292	parY	AAO47226.2	AF235050.4	aminocoumarin resistant parY	aminocoumarin antibiotic	antibiotic target alteration	0.00	0.00	0.00	0.31	0.30	1.85
Lizard06Caudovirales_VIRSorter_scaffold_105497-gene_2|1046|2320|1274|+|Phage_cluster_15111|66.5|3.2e-	gb|NP_417544.5|ARO:3000024|patA	299	37	334	75	355	4.23E-46	33.779	ARO:3000024	36033	2081	patA	AAK76137.1	AE005672.3	ATP-binding cassette (ABC) antibiotic efflux pump	fluoroquinolone antibiotic	antibiotic efflux	0.00	0.61	0.00	0.00	0.00	1.52
Lizard06Caudovirales_VIRSorter_scaffold_84876-gene_2|235|1143|908|+|-|-|-|-|HlyD_2|82.8|4.7e-24(+).p1	gb|AAG07762.1|ARO:3003030|mexV	265	30	294	44	274	6.35E-11	23.019	ARO:3003030	39464	2207	MexV	AAG07762.1	AE004091.2	resistance-nodulation-cell division (RND) antibiotic efflux pump	acridine dye;fluoroquinolone antibiotic;macrolide antibiotic;phenicol antibiotic;tetracycline antibiotic	antibiotic efflux	0.00	0.17	0.00	0.00	0.15	1.93
Lizard06Caudovirales_VIRSorter_scaffold_84330-gene_1|183|929|746|+|Phage_cluster_3079|223.7|2.7e-	gb|CCP46065.1|ARO:3000816|mtrA	230	13	242	5	223	8.76E-57	43.478	ARO:3000816	37196	1559	mtrA	CCP46065.1	AL123456.3	resistance-nodulation-cell division (RND) antibiotic efflux pump	macrolide antibiotic;penam	antibiotic efflux	0.00	0.00	0.00	0.08	0.30	1.36
Lizard06Caudovirales_VIRSorter_scaffold_97319-gene_2|260|568|308|-|Phage_cluster_749|60.5|2.6e-	gb|AAA25550.1|ARO:3003105|dfrA3	101	1	101	62	161	1.04E-15	38.614	ARO:3003105	39679	511	dfrA3	AAA25550.1	J03306	trimethoprim resistant dihydrofolate reductase dfr	diaminopyrimidine antibiotic	antibiotic target replacement	0.00	0.77	0.00	0.00	0.00	0.24
Lizard06Caudovirales_VIRSorter_scaffold_58544-gene_2|137|553|416|+|Phage_cluster_405|92.5|3.9e-	gb|AAV85982.1|ARO:3000535|macB	148	1	138	1	148	4.24E-13	28.378	ARO:3000535	36674	487	macB	AAV85982.1	AY768532	ATP-binding cassette (ABC) antibiotic efflux pump	macrolide antibiotic	antibiotic efflux	0.00	0.00	0.00	0.00	0.00	0.16
Lizard06Caudovirales_VIRSorter_scaffold_86368-gene_1|2|736|734|+|Phage_cluster_244|165.9|2e-49|1|Toprim|73.3|2.4e-	gb|AAO47226.2|ARO:3003318|Streptomyces	184	76	244	464	639	2.29E-21	32.065	ARO:3003318	39902	2292	parY	AAO47226.2	AF235050.4	aminocoumarin resistant parY	aminocoumarin antibiotic	antibiotic target alteration	0.00	0.44	0.00	0.08	0.30	1.68
Lizard06Caudovirales_VIRSorter_scaffold_101559-gene_2|316|1008|692|-|Phage_cluster_3811|57.5|1.9e-	gb|AAC75314.1|ARO:3003578|pmrF	218	1	208	8	211	2.35E-20	30.734	ARO:3003578	40188	2014	PmrF	AAC75314.1	U00096	pmr phosphoethanolamine transferase	peptide antibiotic	antibiotic target alteration	0.00	0.00	0.00	0.00	0.00	1.85
Lizard06Caudovirales_VIRSorter_scaffold_50787-gene_1|149|514|365|-|-|-|-|-|Penicillinase_R|109.0|2.5e-32(-).p1	gb|ABU39978.1|ARO:3000160|blaI	120	3	119	8	125	1.37E-10	28.333	ARO:3000160	36299	1374	blaI	ABU39978.1	EF540343.1	blaZ beta-lactamase	penam	antibiotic inactivation	0.00	0.17	0.00	0.00	0.00	0.32
Lizard06unclassified_bacterial_viruses_VIRSorter_scaffold_14976-gene_10|3981|5063|1082|-|Phage_cluster_1277|315.9|2e-	gb|ABO93588.1|ARO:3002715|QnrB2	178	27	198	29	204	4.43E-16	30.337	ARO:3002715	39149	353	QnrB2	ABO93588.1	EF488762	quinolone resistance protein (qnr)	fluoroquinolone antibiotic	antibiotic target protection	0.00	0.00	0.00	0.00	0.00	8.26
Lizard06unclassified_bacterial_viruses_VIRSorter_scaffold_78317-gene_6|4427|5212|785|+|Phage_cluster_405|94.6|1.5e-	gb|AET10445.1|ARO:3004033|tetB(46)	233	20	243	350	563	1.08E-12	27.039	ARO:3004033	41066	2644	tetB(46)	AET10445.1	HQ652506.1	ATP-binding cassette (ABC) antibiotic efflux pump	tetracycline antibiotic	antibiotic efflux	0.00	0.00	0.00	0.23	2.66	45.89
Lizard06unclassified_bacterial_viruses_VIRSorter_scaffold_4593-gene_9|6084|7013|929|-|Phage_cluster_3811|127.8|1.2e-	gb|AAC75314.1|ARO:3003578|pmrF	294	1	293	17	297	2.09E-45	30.612	ARO:3003578	40188	2014	PmrF	AAC75314.1	U00096	pmr phosphoethanolamine transferase	peptide antibiotic	antibiotic target alteration	0.09	10.36	0.00	0.00	0.44	7.06
Lizard06unclassified_bacterial_viruses_VIRSorter_scaffold_64769-gene_2|280|1113|833|+|gi_418489409_ref_YP_007003226.1_|87.0|1.3	gb|BAD95494.1|ARO:3002895|SAT-1	87	162	248	61	146	1.72E-07	28.736	ARO:3002895	39329	1285	SAT-2	BAD95494.1	AB211124	streptothricin acetyltransferase (SAT)	nucleoside antibiotic	antibiotic inactivation	0.00	0.00	0.00	0.08	0.44	10.99
Lizard06unclassified_bacterial_viruses_VIRSorter_scaffold_30432-gene_1|1|1149|1148|+|Phage_cluster_405|101.4|1.3e-	gb|AAV85982.1|ARO:3000535|macB	114	6	119	90	202	4.88E-16	35.088	ARO:3000535	36674	487	macB	AAV85982.1	AY768532	ATP-binding cassette (ABC) antibiotic efflux pump	macrolide antibiotic	antibiotic efflux	0.00	0.00	0.00	0.00	0.00	1.85
Lizard06unclassified_bacterial_viruses_VIRSorter_scaffold_69210-gene_1|222|1430|1208|+|gi_481019673_ref_YP_007878024.1_|52.0|1.	gb|CAA67349.1|ARO:3003359|Streptomyces	405	1	400	1	395	0	67.16	ARO:3003359	39943	2310	EF-Tu	CAA67349.1	X98831.1	elfamycin resistant EF-Tu	elfamycin antibiotic	antibiotic target alteration	0.05	0.00	0.41	0.62	1.03	9.15
Lizard06unclassified_bacterial_viruses_VIRSorter_scaffold_22158-gene_1|1|550|549|-|-|-|-|-|MerR|88.2|5.5e-27(-).p1	gb|NP_459349.1|ARO:3000504|golS	126	9	124	7	123	1.00E-08	35.714	ARO:3000504	36643	44	golS	AAL19308.1	AE006468.2	resistance-nodulation-cell division (RND) antibiotic efflux pump	carbapenem;cephalosporin;cephamycin;monobactam;penam;penem;phenicol antibiotic	antibiotic efflux	0.00	0.00	0.00	0.00	0.00	0.56
Lizard06unclassified_dsDNA_phages_VIRSorter_scaffold_29337-gene_9|9478|10842|1364|-|-|-|-|-|OEP|165.0|3.6e-51(-).p1	gb|NP_249118.1|ARO:3000379|OprM	468	1	451	3	466	2.20E-48	28.632	ARO:3000379	36518	1305	OprM	AAG03816.1	AE004091.2	resistance-nodulation-cell division (RND) antibiotic efflux pump	acridinedye;aminocoumarin;aminoglycoside;carbapenem;cephalosporin;cephamycin;diaminopyrimidine;fluoroquinolone;macrolide;monobactam;penam;penem;peptide;phenicol;sulfonamide;tetracycli	antibiotic efflux	0.00	12.46	0.00	0.00	1.03	601.37
Lizard06unclassified_dsDNA_phages_VIRSorter_scaffold_29337-gene_10|10839|14003|3164|-|-|-|-|-|ACR_tran|965.4|6.8e-293(-).p1	gb|ABS43151.1|ARO:3000784|cmeB	1030	1	1020	13	1029	0	37.573	ARO:3000784	37164	748	cmeB	ABS43151.1	CP000768	resistance-nodulation-cell division (RND) antibiotic efflux pump	cephalosporin;fluoroquinolone antibiotic;fusidic acid;macrolide antibiotic	antibiotic efflux	0.00	40.57	0.14	0.08	7.23	2037.72
Lizard06unclassified_dsDNA_phages_VIRSorter_scaffold_29337-gene_11|14056|15042|986|-|-|-|-|-|HlyD|106.4|4.3e-33(-).p1	gb|ABS43901.1|ARO:3000783|cmeA	323	1	320	48	362	3.91E-43	30.65	ARO:3000783	37163	1181	cmeA	ABS43901.1	CP000768	resistance-nodulation-cell division (RND) antibiotic efflux pump	cephalosporin;fluoroquinolone antibiotic;fusidic acid;macrolide antibiotic	antibiotic efflux	0.00	6.39	0.00	0.00	0.74	468.44
Lizard06unclassified_dsDNA_phages_VIRSorter_scaffold_9745-gene_2|286|3333|3047|-|-|-|-|-|ACR_tran|808.7|1.4e-245(-).p1	gb|AAC75137.1|ARO:3000794|mdtC	1031	7	993	8	1018	1.20E-116	26.382	ARO:3000794	37174	1315	mdtC	AAC75137.1	U00096	resistance-nodulation-cell division (RND) antibiotic efflux pump	aminocoumarin antibiotic	antibiotic efflux	0.23	1.21	0.14	11.27	18.74	694.99
Lizard06unclassified_dsDNA_phages_VIRSorter_scaffold_9745-gene_3|3425|4462|1037|-|-|-|-|-|HlyD_2|96.0|6.3e-30(-).p1	gb|NP_252895.1|ARO:3000807|mexH	356	4	341	13	354	1.18E-26	29.775	ARO:3000807	37187	1344	MexH	AAG07593.1	AE004091.2	resistance-nodulation-cell division (RND) antibiotic efflux pump	acridine dye;fluoroquinolone antibiotic;tetracycline antibiotic	antibiotic efflux	0.05	0.11	0.00	0.00	0.30	51.10
Lizard06unclassified_dsDNA_phages_VIRSorter_scaffold_9745-gene_4|4513|5814|1301|-|-|-|-|-|OEP|155.3|3.5e-48(-).p1	gb|NP_249118.1|ARO:3000379|OprM	426	25	426	62	461	1.16E-07	23.239	ARO:3000379	36518	1305	OprM	AAG03816.1	AE004091.2	resistance-nodulation-cell division (RND) antibiotic efflux pump	acridinedye;aminocoumarin;aminoglycoside;carbapenem;cephalosporin;cephamycin;diaminopyrimidine;fluoroquinolone;macrolide;monobactam;penam;penem;peptide;phenicol;sulfonamide;tetracycli	antibiotic efflux	0.00	0.11	0.00	0.23	0.15	24.07
Lizard06unclassified_dsDNA_phages_VIRSorter_scaffold_9745-gene_5|5922|6563|641|-|-|-|-|-|TetR_N|44.1|3.1e-14(-).p1	gb|AAC76296.1|ARO:3000656|acrS	219	3	206	6	216	7.04E-09	21.005	ARO:3000656	37000	520	AcrS	AAC76296.1	U00096	resistance-nodulation-cell division (RND) antibiotic efflux pump	cephalosporin;cephamycin;fluoroquinolone antibiotic;glycylcycline;penam;phenicol antibiotic;rifamycin antibiotic;tetracycline antibiotic;triclosan	antibiotic efflux	0.00	0.00	0.00	0.08	0.00	5.54
Lizard06unclassified_dsDNA_phages_VIRSorter_scaffold_23413-gene_1|1|1140|1139|+|-|-|-|-|HlyD_2|92.5|6.9e-29(+).p1	gb|NP_274719.1|ARO:3000810|mtrC	283	102	359	91	368	3.05E-13	25.442	ARO:3000810	37190	1340	mtrC	AAF42063.1	AE002098.2	resistance-nodulation-cell division (RND) antibiotic efflux pump	macrolide antibiotic;penam	antibiotic efflux	0.00	0.00	0.00	0.85	0.30	2.57
Lizard06unclassified_dsDNA_phages_VIRSorter_scaffold_23413-gene_2|1200|2720|1520|+|-|-|-|-|ACR_tran|465.5|6.8e-142(+).p1	gb|AAG07763.1|ARO:3003031|mexW	497	1	489	13	493	3.64E-54	28.169	ARO:3003031	39465	2208	MexW	AAG07763.1	NC_002516.2	resistance-nodulation-cell division (RND) antibiotic efflux pump	acridine dye;fluoroquinolone antibiotic;macrolide antibiotic;phenicol antibiotic;tetracycline antibiotic	antibiotic efflux	0.00	0.00	0.00	4.58	0.44	4.81
Lizard06unclassified_dsDNA_phages_VIRSorter_scaffold_23413-gene_3|2717|4231|1514|+|-|-|-|-|ACR_tran|408.4|1.2e-124(+).p1	gb|AAC75137.1|ARO:3000794|mdtC	514	1	497	519	1010	2.28E-34	25.097	ARO:3000794	37174	1315	mdtC	AAC75137.1	U00096	resistance-nodulation-cell division (RND) antibiotic efflux pump	aminocoumarin antibiotic	antibiotic efflux	0.00	0.00	0.00	4.89	0.89	9.31
Lizard06unclassified_dsDNA_phages_VIRSorter_scaffold_15738-gene_1|1|3016|3015|-|-|-|-|-|ACR_tran|863.8|3.2e-262(-).p1	gb|CAH14033.1|ARO:3004100|LpeB	1012	1	1004	23	987	1.17E-79	25.791	ARO:3004100	41209	2759	LpeB	CAH14033.1	CR628336.1	resistance-nodulation-cell division (RND) antibiotic efflux pump	macrolide antibiotic	antibiotic efflux	0.88	109.53	3.59	1.79	6.05	54.47
Lizard06unclassified_dsDNA_phages_VIRSorter_scaffold_15738-gene_2|3177|4448|1271|-|-|-|-|-|HlyD_2|86.3|5.6e-27(-).p1	gb|AAG07762.1|ARO:3003030|mexV	324	106	408	53	358	7.75E-15	26.235	ARO:3003030	39464	2207	MexV	AAG07762.1	AE004091.2	resistance-nodulation-cell division (RND) antibiotic efflux pump	acridine dye;fluoroquinolone antibiotic;macrolide antibiotic;phenicol antibiotic;tetracycline antibiotic	antibiotic efflux	0.18	26.51	1.69	0.16	0.59	12.84
Lizard06unclassified_dsDNA_phages_VIRSorter_scaffold_3935-gene_1|1|1373|1372|-|-|-|-|-|ACR_tran|277.7|3.6e-85(-).p1	gb|AAC75137.1|ARO:3000794|mdtC	462	1	456	20	446	1.40E-38	25.974	ARO:3000794	37174	1315	mdtC	AAC75137.1	U00096	resistance-nodulation-cell division (RND) antibiotic efflux pump	aminocoumarin antibiotic	antibiotic efflux	0.00	20.12	0.00	0.62	0.59	12.68
Lizard06unclassified_dsDNA_phages_VIRSorter_scaffold_3935-gene_2|1518|2618|1100|-|-|-|-|-|HlyD_2|116.0|5.2e-36(-).p1	gb|NP_252895.1|ARO:3000807|mexH	201	171	363	165	358	3.48E-11	31.343	ARO:3000807	37187	1344	MexH	AAG07593.1	AE004091.2	resistance-nodulation-cell division (RND) antibiotic efflux pump	acridine dye;fluoroquinolone antibiotic;tetracycline antibiotic	antibiotic efflux	0.00	9.81	0.14	0.00	0.30	5.78
Lizard06unclassified_dsDNA_phages_VIRSorter_scaffold_5163-gene_1|127|477|350|-|-|-|-|-|ACR_tran|121.0|7.8e-38(-).p1	gb|NP_274718.1|ARO:3000811|mtrD	106	8	113	937	1042	1.81E-19	31.132	ARO:3000811	37191	1938	mtrD	AAF42062.1	AE002098.2	resistance-nodulation-cell division (RND) antibiotic efflux pump	macrolide antibiotic;penam	antibiotic efflux	0.00	0.00	0.07	0.00	0.00	0.56
